# Total Synthesis of Homoseongomycin Enantiomers and Evaluation of Their Optical Rotation

**DOI:** 10.1021/acsomega.4c04249

**Published:** 2024-06-28

**Authors:** Greg Petruncio, Zachary Shellnutt, Lauren L. Young, Michael Girgis, Wendy K. Strangman, R. Thomas Williamson, Kylene Kehn-Hall, Mikell Paige

**Affiliations:** † Department of Chemistry & Biochemistry, George Mason University, 10920 George Mason Circle, Manassas, Virginia 20110, United States; ‡ Center for Molecular Engineering, George Mason University, 10920 George Mason Circle, Manassas, Virginia 20110, United States; § Department of Bioengineering, George Mason University, 10920 George Mason Circle, Manassas, Virginia 20110, United States; ∥ Department of Chemistry and Biochemistry, Center for Marine Science, University of North Carolina Wilmington, Wilmington, North Carolina 28409, United States; ⊥ Department of Biomedical Sciences and Pathobiology, Virginia−Maryland College of Veterinary Medicine, Virginia Polytechnic Institute and State University, Blacksburg, Virginia, 24061, United States; # Center for Emerging, Zoonotic, and Arthropod-Borne Pathogens, Virginia Polytechnic Institute and State University, Blacksburg, Virginia 24061, United States

## Abstract

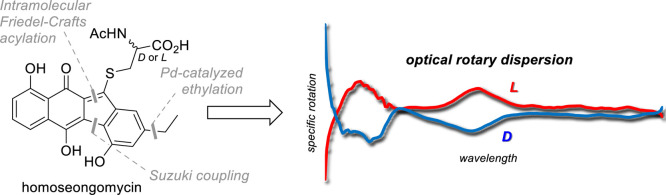

A total synthesis of each homoseongomycin enantiomer was accomplished in 17 total steps (longest linear sequence = 12 steps) and 10 chromatographic purifications. Several schemes were attempted to forge the key 5-membered ring, but only a Suzuki coupling-intramolecular Friedel–Crafts acylation sequence proved viable. Challenges encountered during the optical rotation characterization of the natural product left us with two important takeaways. First, highly colored compounds like homoseongomycin that absorb near/at the sodium *d*-line may require optical rotation measurements at other wavelengths. Second, high dilution of such compounds to obtain measurement at the sodium *d*-line could result in artificially large and incorrectly assigned specific rotations. To verify the optical rotation, electronic circular dichroism spectra were acquired for both homoseongomycin enantiomers and were transformed into optical rotary dispersions via the Kramers–Kronig transform. We note the wavelength dependency on rotation, and at the sodium d-line 589 nm, we reassign the optical rotation of *L*-homoseongomycin from (−) to (+).

## Introduction

**Figure 1:**
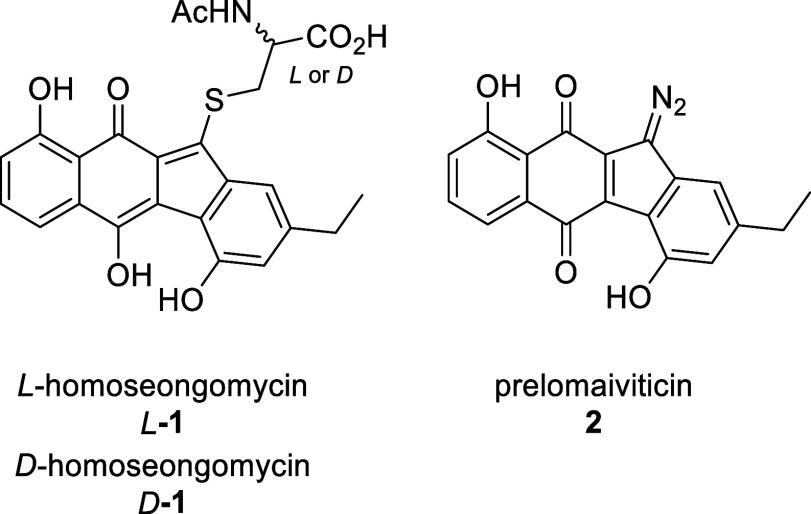
Structures of the natural products.

*L*-Homoseongomycin (*L***-1**) was isolated by the Herzon group and shown through isotope labeling to be derived from diazofluorene prelomaiviticin (**2**) (Figure 1).^1^ The isotopically labeled *L*-**1** was achieved by total synthesis, which featured fluoride-mediated fragment coupling and subsequent Heck cyclization to establish the tetracyclic scaffold.^1,2^ Diazo transfer furnished **2**, which was then subjected to substitution by *N*-acetyl-*l*-cysteine to yield *L*-**1**.^1^

The precursor natural product **2** can be regarded as a homologue of the more well-known natural product prekinamycin, which serves as a precursor to cytotoxic kinamycins. This structural similarity led to the hypothesis and later confirmation that **2** acts as a biosynthetic precursor to the cytotoxic lomaiviticins.^3^

The absolute configuration of homoseongomycin was assumed to be the *L*-configuration based on comparison to its homologue seongomycin, whose absolute configuration was determined through cleavage of the *N*-acetyl cysteine side chain and analysis by Marfey’s method.^1,4^ The reported specific rotation of *L*-**1** was a notably large rotation of [α]_*D*_^20^ = −525° (*c* = 0.001, MeOH).^1^ Other compounds with comparable specific rotations include certain bicyclic ketones,^5^ paracyclophane derivatives,^6^ and natural products such as dihydroobionin B,^7^ dibrevianamide F,^8^ and falcarindiol,^9^ to name a few. Assuming that the optical rotation of *L*-**1** was measured in a 1 dm cell, back calculation of the reported specific rotation affords a small optical rotation of −0.00525°.

## Results and Discussion

**Scheme 1:**
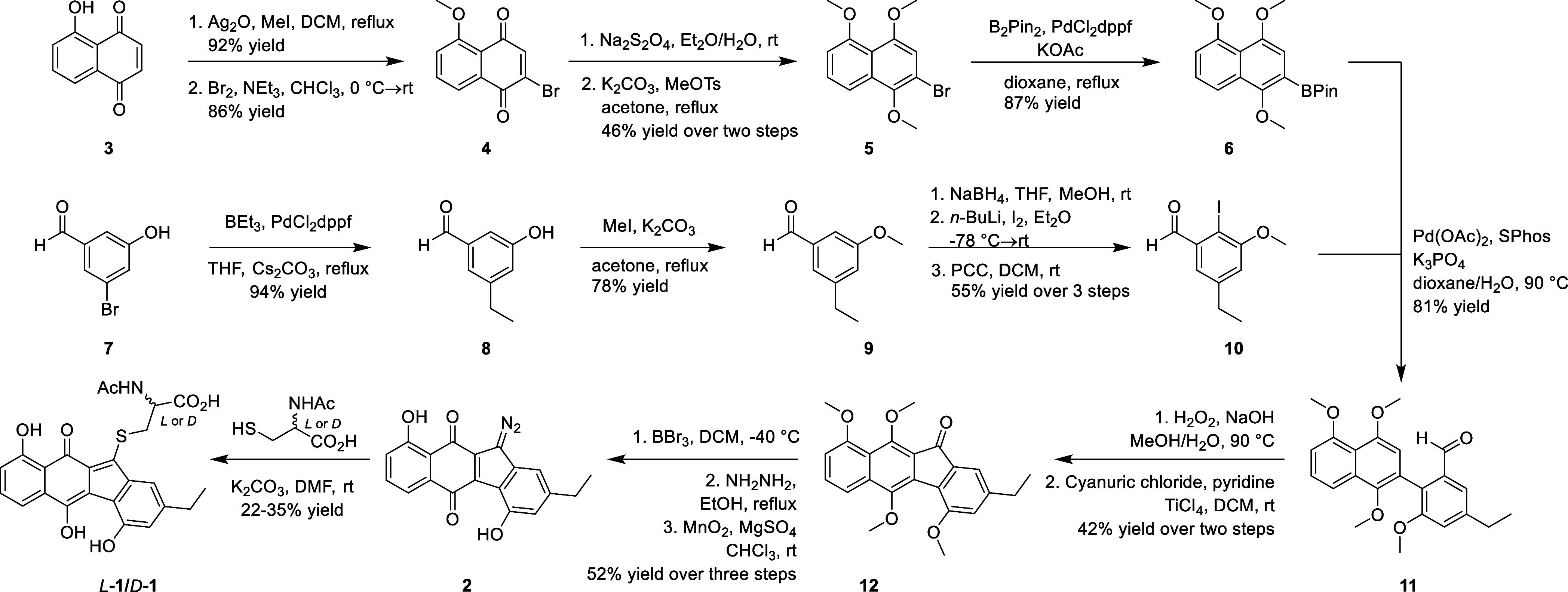
Total Synthesis of *L*- and *D*-Homoseongomycin

We previously demonstrated *L*-**1** as an inhibitor of the Venezuelan equine encephalitis virus (VEEV) with low cytotoxicity toward healthy cells.^10^ To facilitate a mechanistic study of *L*-**1** on VEEV, we sought to synthesize the material starting from juglone (**3**), which was methylated in the presence of silver(I) oxide, to give methylated juglone in almost quantitative yield without the need for chromatography (Scheme 1). Treatment with ∼1 equiv of bromine (Br_2_) followed by triethylamine-promoted elimination gave brominated juglone **4** in 86% yield as the sole product. Of note, attempted monobromination of **3** before phenol methylation resulted in low conversion and a mixture of products. Sodium dithionite reduction of **4** in a biphasic solvent system followed by methylation with methyl *p*-toluenesulfonate (MeOTs) afforded masked quinone **5** in 46% yield over two steps. Finally, Suzuki–Miyaura borylation gave pinacol boronate **6** in 87% yield. Synthesis of the other fragment commenced with Pd-catalyzed ethylation of aldehyde **7**, which installed the ethyl side chain of **1** in 94% yield.^11^ Aldehyde **8** was then protected as methyl ether to give **9** in 78% yield. Interestingly, the Pd-catalyzed ethylation reaction proceeded with a much higher yield in the presence of an unprotected phenol than that in the presence of a methyl ether.

We then attempted various aldehyde *ortho*-functionalization reactions to convert aldehyde **9** into a cross-coupling handle for coupling with either **5** or **6**. One such method that was used in various total syntheses on similar substrates^12,13^ was the Comins protocol.^14^ The procedure “protects” aldehydes as α-amino alkoxides in situ, which act as directing groups for *ortho*-lithiation reactions. We subjected **9** to the Comins protocol and trapped the lithiated species with triethyl borate (B(OEt)_3_). Transesterification with *N*-methyliminodiacetic acid (MIDA) in DMF afforded a MIDA boronate-functionalized aldehyde in 42% yield over 2 steps at ∼50 mg scale.^15^ Unfortunately, moderate scaling-up of the reaction resulted in a significant decrease in yield, and the transesterification step proved difficult to reproduce from trial-to-trial [see Supporting Information for details]. Trapping the lithiated species with other electrophiles such as iodine (I_2_) or 2-isopropoxy-4,4,5,5-tetramethyl-1,3,2-dioxaborolane (*i*PrOBPin) afforded low yields and impure products, which led us to abandon the Comins protocol.

We then found that the reduction of aldehyde **9** provided a benzyl alcohol directing group that allowed for reproducible *ortho*-lithiation and trapping by I_2_ [trapping with B(OEt)_3_ under these conditions resulted in no reaction].^16,17^ Oxidation with PCC afforded iodinated aldehyde **10** in 55% yield over 3 steps, needing only one chromatographic purification of the final product. The 3-step sequence proved reproducible over multiple trials and was scalable. The location of the iodide in the key intermediate **10** was determined by a 2D NMR ^13^C–^13^C correlation (INADEQUATE) experiment.

With **6** and **10** in hand, Suzuki coupling using the SPhos ligand to join the sterically hindered pieces was accomplished in 81% yield.^18^ Biaryl aldehyde **11** was then oxidized to the corresponding carboxylic acid in a refluxing aqueous sodium hydroxide-hydrogen peroxide (NaOH–H_2_O_2_) system. Intramolecular Friedel–Crafts (IMFC) acylation was carried out with cyanuric chloride,^19^ followed by addition of Lewis acid titanium(IV) chloride (TiCl_4_)^12^ in one-pot at room temperature to afford ketone **12** in 42% yield over 2 steps.

The final sequence begins with global deprotection of the methyl ethers in **12** by boron tribromide (BBr_3_). Treatment of the resultant crude product with anhydrous hydrazine under refluxing conditions yielded a crude hydrazone, which upon exposure to manganese dioxide gave diazo **2** in 52% yield over 3 steps. Treatment of precursor **2** with either *N*-acetyl-*l*-cysteine or *N*-acetyl-*d*-cysteine followed by reverse phase purification afforded enantiomers *L*-**1** and *D*-**1** in 22–35% yield as purple solids.^1^ Overall, the total synthesis was accomplished in 17 total steps with the longest linear sequence of 12 steps and only 10 chromatographic purifications.

**Figure 2:**
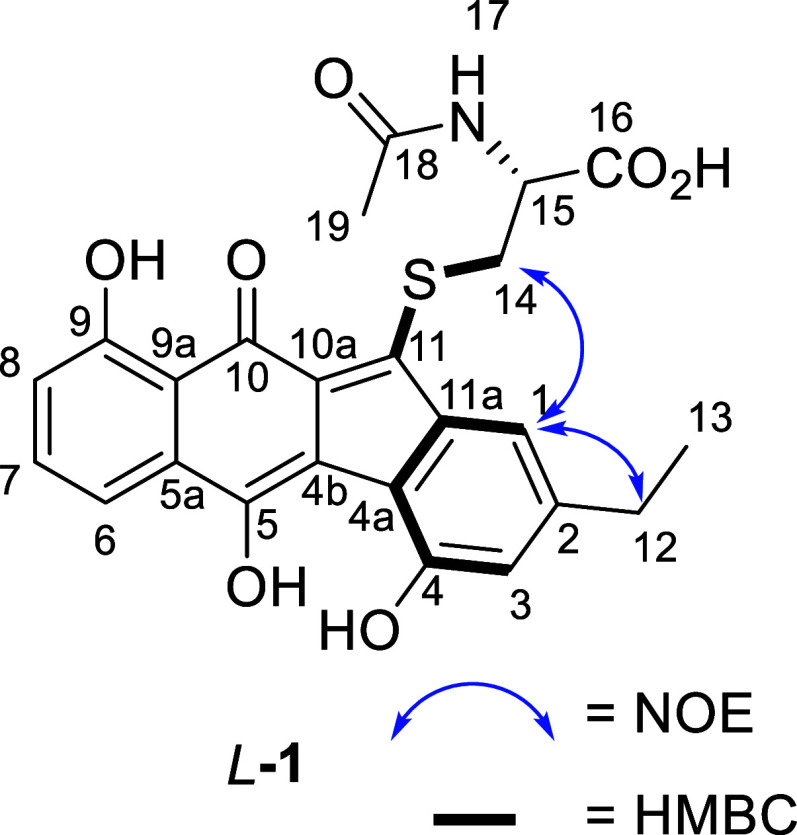
HMBC and NOE correlations of 1.

^1^H NMR data of synthetic *L*-**1** were compared to several literature ^1^H NMR data of natural *L*-**1** and were found to match *after* the addition of TFA to the DMSO-*d*_6_ solvent.^1,10^ However, this caused ^13^C signals from vinylogous enol carbons C5 and C11 to resonate outside experimental error (Δδ > 3 ppm). The C5 and C11 chemical shifts were in good agreement when synthetic *L*-**1** was compared to seongomycin isolated by Carney [see Supporting Information for details].^4^ HMBC and NOE correlations supported correct placement of the cysteine side chain (Figure 2) and matching HPLC retention times, and HRMS gave further confidence of the successful synthesis of *L*-**1** and *D*-**1**.

The last data point needed for full characterization was the optical rotation of each enantiomer. To our surprise, we were unable to obtain any signal at concentrations (*c*) greater than 0.01 g/100 mL due to the strong absorbance of the sodium-*D* wavelength, which was supported by UV–vis spectroscopy of *L*-**1**. Only at *c* = 0.002 g/100 mL was the sample dilute enough for light to pass through the cell, giving an observed rotation of α = +0.004° to +0.005° for *L*-**1**. This observed rotation was then converted to a specific rotation of +200° to +250°, which was the opposite sign of Herzon’s isolate material.^1^ Under identical conditions, *D*-**1** gave no observed rotation, leading us to suspect that the observed rotation for *L*-**1** was below the detection limit of the instrument and simply an artifact of instrument noise.

**Figure 3:**
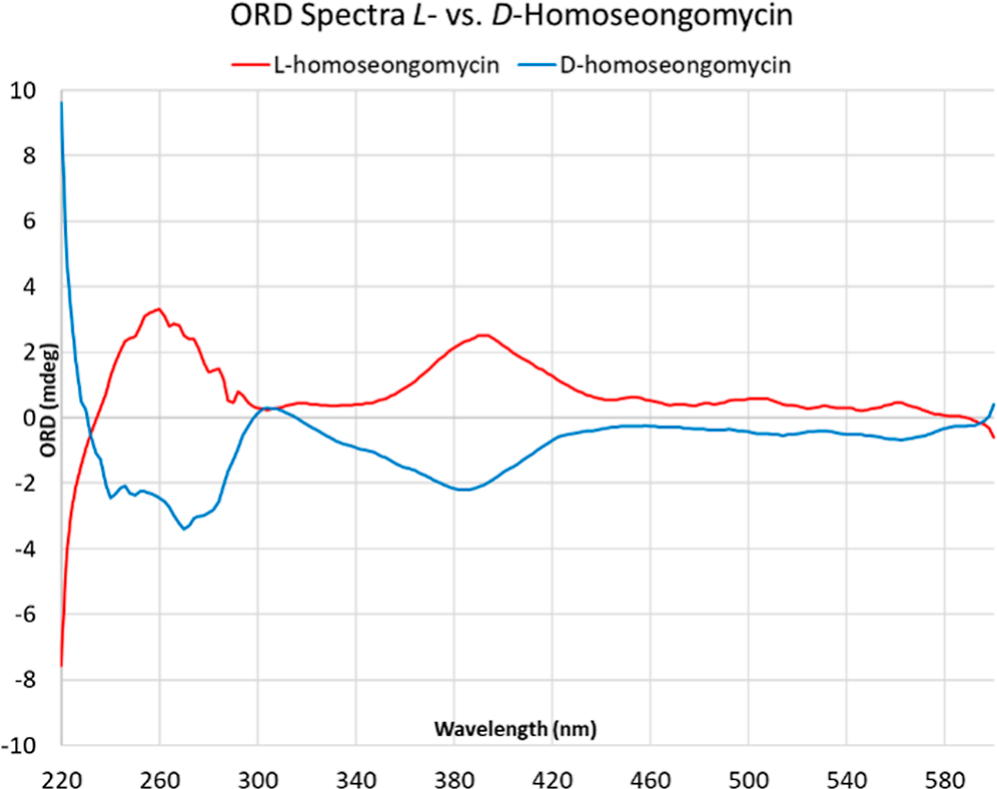
ORD spectra for *L*-1 and *D*-1.

An underexploited application of electronic circular dichroism (ECD) is its conversion to optical rotary dispersion (ORD) via the Kramers–Kronig (KK) transform.^20^ An experimental ECD spectrum can be used to calculate the optical rotation of a sample at wavelengths where it is highly absorbing. After acquiring ECD spectra of both *L*-**1** and *D*-**1**, we then carried out both a software-assisted and manual KK transform using the Ohta–Ishida method as recommended by Polavarapu.^20^ The resultant ORD spectra from both KK transform methods were in decent agreement with each other and showed that *L*-**1** exhibits (+) rotation at standard wavelengths, such as 365, 405, 436, and 546 nm. The only discrepancy between the two methods was at 589 nm with a calculated [α]_*D*_^25^ = +2.18° for one method and −37.2° for the other. By extension, *D*-**1** exhibits (−) rotation across the same wavelengths, as well as at 589 nm, with a calculated [α]_*D*_^25^ = −36.4° and −24.6° from the two methods (Figure 3).

On the basis of these findings, we reassign *L*-**1** as (+)-homoseongomycin and *D*-**1** as (−)-homoseongomycin at 589 nm, which would be valid for most wavelengths we measured. This optical rotation study left us with an important takeaway: compounds that absorb strongly near the sodium-*D* line like homoseongomycin may not permit experimental optical rotation at that particular wavelength, and dilution of such compounds to low enough concentrations may lead to artificially large specific rotations and potential misassignment. In these cases, an experimental ECD spectrum’s conversion to an ORD via the KK transform appears to be a viable alternative. With this in mind, we include an Excel KK transform spreadsheet that uses the Ohta–Ishida method as recommended by Polavarapu to automatically generate an ORD when provided with ECD data [see Supporting Information for details].^20^ Our experience highlights the importance of measuring optical rotation measurements at several standard wavelengths (365, 405, 436, 546, 589, and 633 nm) instead of a single measurement at the sodium-*D* line, which is a standard practice for assessing batch-to-batch purity in the saccharide industry.^21^ In addition, recording experimental optical rotation at two or more wavelengths has been noted to improve the accuracy of absolute configuration determination via computational ORD predictions.^22^ Unfortunately, many academic laboratories only possess a single-wavelength polarimeter. We also note the importance of including the observed rotation α (or cell length and concentration) so other researchers can note the raw data used to generate the reported specific rotation [α].

## Conclusions

A total synthesis of both homoseongomycin enantiomers was accomplished. The synthesis features a Suzuki coupling-IMFC acylation sequence to generate the key tetracyclic scaffold. A challenge encountered during the synthesis was the *ortho*-functionalization of an aryl aldehyde fragment. On the characterization side, the intense dark purple color of homoseongomycin prevented the acquisition of experimental optical rotation, which led us to use the KK transform to calculate the optical rotation. With this technique, we were able to provide the wavelength dependency of optical rotation for both enantiomers of homoseongomycin.

## Supporting Information Available

Synthetic schemes, procedures, characterization data, spectra, raw ECD data, and manual KK transform (PDF)
KK transform spreadsheet (XLSX)

## Abbreviations

VEEV: venezuelan equine encephalitis virus
KK: Kramers–Kronig
ECD: electronic circular dichroism
LLS: longest linear sequence
ORD: optical rotary dispersion
IMFC: intramolecular Friedel–Crafts acylation
