# Supplementary material for: Total Synthesis of Homoseongomycin Enantiomers and Evaluation of Their Optical Rotation
Source: ACS Omega. 2024 Jun 28;9(28):30993–7. doi: 10.1021/acsomega.4c04249 (PMC11256097; doi:10.1021/acsomega.4c04249)
Supplement: Supplementary file 1 — ao4c04249_si_001.pdf [file ao4c04249_si_001.pdf]

Total Synthesis of Homoseongomycin Enantiomers and Evaluation of their Optical Rotation

Greg Petruncio,[†§] Zachary Shellnutt,[†Δ] Lauren L. Young,[†] Michael Girgis,[¶§] Wendy K. Strangman,[‖] R. Thomas Williamson,[‖] Kylene Kehn-Hall,[‡] Mikell Paige,[†§*]

† Department of Chemistry & Biochemistry, George Mason University, 10920 George Mason Circle, Manassas, Virginia 20110, United States

§ Center for Molecular Engineering, George Mason University, 10920 George Mason Circle, Manassas, Virginia 20110, United States

¶ Department of Bioengineering, George Mason University, 10920 George Mason Circle, Manassas, Virginia 20110, United States

‖ Department of Chemistry and Biochemistry, Center for Marine Science, University of North Carolina Wilmington, Wilmington, North Carolina 28409, United States

‡ Department of Biomedical Sciences and Pathobiology, Virginia−Maryland College of Veterinary Medicine, Virginia Polytechnic Institute and State University, Blacksburg, Virginia 24061, United States; Center for Emerging, Zoonotic, and Arthropod-Borne Pathogens, Virginia Polytechnic Institute and State University, Blacksburg, Virginia 24061, United States

[Δ] Current affiliation: Department of Chemistry, Chevron Science Center, University of Pittsburgh, Pennsylvania 15260, United States

Supporting Information

Contents

List of Abbreviations S3

Schemes, Synthetic Procedures, and Characterization Data S4

- Unsuccessful Schemes for Synthesis of Model Natural Product Dideoxy Deethyl Prelomaivicitin S5
  - Scheme S1. Radical Functionalization-Heck Cyclization Strategy S5
  - Scheme S2. Oxidative Dearomatization-Heck Cyclization Strategy S7
  - Scheme S3. Carbonyl Addition-Cyclization Strategy S9
- Successful Synthesis of Model Natural Product Dideoxy Deethyl Prelomaiviticin S11
  - Scheme S4. Suzuki Coupling-Intramolecular Friedel-Crafts (IMFC) Acylation Strategy S11
- Successful Total Synthesis of Homoseongomycin S28
  - Scheme S5. Suzuki Coupling-Intramolecular Friedel-Crafts (IMFC) Acylation Strategy S28

Synthetic Homoseongomycin NMR Data Comparison S57

Spectra S63

- Compound 1 ((L)-Homoseongomycin) S63
- Compound 1 ((D)-Homoseongomycin) S82
- Compound 2 (prelomaiviticin) S83

Compound 4 .... S89
Compound 5 .... S92
Compound 6 .... S96
Compound 8 .... S101
Compound 9 .... S105
Compound 10 .... S109
Compound 11 .... S115
Compound 12 .... S120
Compound 16 .... S127
Compound 22 .... S133
Compound 24 .... S137
Compound 25 .... S142
Compound 27 .... S145
Compound 28 .... S147
Compound 31 .... S150
ECD Raw Data and KK Transform S156
References S182

List of Abbreviations

Atmospheric pressure chemical ionization	APCI
Electronic circular dichroism	ECD
Decomposition	decomp.
2,4-Dinitrophenylhydrazine	DNP
Dichloromethane	DCM
Electrospray ionization	ESI
Equivalent	equiv. or eq.
Ethyl acetate	EtOAc
Hexanes	Hex
High-performance liquid chromatography	HPLC
Infrared	IR
(High-resolution) mass spectrometry	(HR)MS
Kramers-Kronig	KK
Methanol	MeOH
Millidegree	mdeg
No reaction	NR
Not determined	n.d.
Nuclear magnetic resonance	NMR
Optical rotary dispersion	ORD
Preparatory liquid chromatography	Prep LC
Reaction	rxn
Retention factor	R_f
Room temperature	rt
Rotary evaporation	rotavap
Round-bottom flask	rbf
Solution	soln.
Starting material	SM
Tetrahydrofuran	THF
Thin layer chromatography	TLC
Ultraviolet-visible	UV-Vis
Ultra-performance liquid chromatography	UPLC

Schemes, Synthetic Procedures, and Characterization Data

General Special instructions regarding reagents are noted in the appropriate procedures where they are employed. "Drying an organic layer" refers to treatment with Na_2SO_4 or $MgSO_4$ after aqueous workup and then decanting. "Flame-drying" refers to heating glassware with a propane torch for one minute immediately prior to use. "Flushing/purging a reaction vessel" refers to delivering an inert gas through a septum for several minutes. A Biotage Initiator, Biotage microwave vials, and Biotage aluminum caps were used for microwave reactions. Reaction progress was monitored on SiliCycle F^{254} TLC plates. "TLCMS" refers to MS analysis of spots on a TLC plate. Reactions were concentrated using Biotage V10 Touch or various Buchi rotary evaporators with water bath temperatures between 40-50 °C. Products were purified using SiliCycle SiliaFlash F60 silica gel (40-63 µm, 230-400 mesh) with solvent mixtures specified in the corresponding experiment. Tables are provided for specific reactions where optimization was required.

Materials Trimethylsilylmethylmagnesium chloride ($TMSCH_2MgCl$) 1 M ether solution was purchased from TCI America. Chloromethyl methyl ether (MOMCl) was prepared according to the procedure of Wang *et al*.[1] Tetrabutylammonium fluoride (TBAF) 1 M THF solution was purchased from Thermo Scientific Chemicals. Anhydrous TBAF was prepared according to the procedure of Sun *et al*.[2] Tetrabutylammonium fluoride tri(1-adamantanol) [TBAF(Ada-OH)$_3$] was prepared according to the procedure of Engle *et al*.[3] Tris(dimethylamino)sulfonium difluorotrimethylsilicate (TASF(Me)) technical grade was purchased from Sigma-Aldrich. Lithium diisopropylamide (LDA) 2M solution in THF/n-heptane/ethylbenzene was purchased from Thermo Scientific Chemicals. Potassium bis(trimethylsilyl)amide (KHMDS) 1 M THF solution was purchased from Sigma-Aldrich. Triphenylphosphine, polymer-bound, on styrene-divinylbenzene copolymer (20% cross-linked) (PS-PPh_3) was purchased from Strem Chemicals. *n*-Butyllithium (*n*-BuLi) 2.5M solution in hexanes and *sec*-Butyllithium (*sec*-BuLi) 1.4 M solution in cyclohexane were purchased from Sigma-Aldrich. Triethylborane (BEt_3) 1 M solution in THF was purchased from Thermo Scientific Chemicals.

Instrumentation APCI MS was performed on an Advion Expression CMS. TLCMS was performed on Advion Expression CMS Plate Express attachment, which allows for direct APCI MS analysis of spots on a TLC plate. ^1H and ^{13}C NMR spectra were obtained on a Bruker 400 MHz spectrometer equipped with a PA BBO probe or a 500 MHz spectrometer equipped with a cryoprobe and spectra were calibrated using the solvent residual peak. HRESIMS data were collected on a Waters G2-XS QToF coupled to a Waters I-Class UPLC. Reverse phase chromatography (i.e. prep-LC) was performed on Waters 2545 Binary Gradient Module equipped with a Waters 2489 UV/Vis Detector. IR spectra were obtained on an Agilent Cary-FTIR. Polarimetry was performed on Rudolph Autopol IV. LCMS was run on a SciEx QTRAP 4100. ECD was performed on Jasco J-1500 CD Spectrometer. UV-Vis was conducted on an Agilent Cary 60 UV-Vis spectrometer. Fluorescence spectroscopy was run on Jasco FP-8300 fluorometer.

Unsuccessful Schemes for Synthesis of Model Natural Product Dideoxy Deethyl Prelomaiviticin

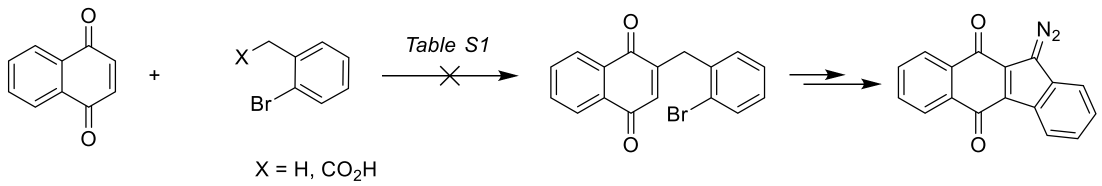

13 14 15 16

Scheme S1. Radical Functionalization-Heck Cyclization Strategy

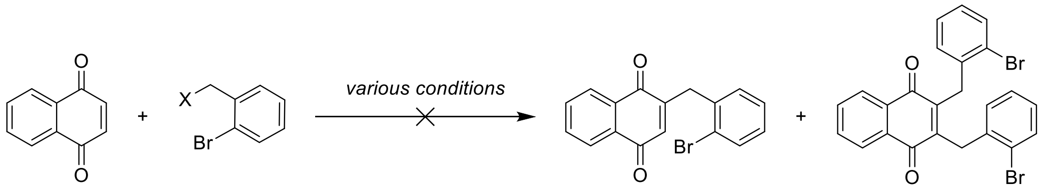

13 **14** **15** **17**

Table S1. Radical functionalization trials.

Trial	Scale	SM **13**:SM **14**	X	Conditions	Observations	Yield	Ref.
1	158 mg	1:8.3	H	DTBP (2 equiv.), 120 °C	TLCMS confirmed undesired product **17** only	-	[4]
2	158 mg	1:2	CO_2H	$(NH_4)_2S_2O_8$ (1.3 equiv.), $AgNO_3$ (10 mol %), $MeCN/H_2O$, 90 °C	TLCMS indicated undesired **17** at 495 and 497 m/z, which crystallized out of rxn	41%	[5]
3	158 mg	1:1	CO_2H	$(NH_4)_2S_2O_8$ (1.3 equiv.), $AgNO_3$ (13 mol %), $MeCN/H_2O$, 90 °C	NMR showed 1.2:1 ratio of impurity:**15** that could not be chromatographically separated	-	[5]
4	40 mg	1:10	CO_2H	$(NH_4)_2S_2O_8$ (2 equiv.), $DMSO/H_2O$, 40 °C	Crude NMR showed 1:1 ratio of **15**:**17**	-	[6]
5	40 mg	1:2	CO_2H	Selectfluor (2 equiv.), $AgNO_3$ (20 mol %), DCE/H_2O, rt	Crude NMR showed 1.2:1 ratio of impurity:**15**	-	[7]
6	40 mg	1:5	CO_2H	$(NH_4)_2S_2O_8$ (2 equiv.), $DMSO/H_2O$, 40 °C	Crude NMR showed 1.5:1 ratio of **15**:**17**	-	[6]
7	40 mg	1:5	CO_2H	Selectfluor (5 equiv.), $AgNO_3$ (20 mol %), DCE/H_2O, rt	Crude NMR showed 1.6:1 ratio of impurity:**15**	-	[7]
8	40 mg	1:2	CO_2H	$(NH_4)_2S_2O_8$ (2 equiv.), $DMSO/H_2O$, 40 °C	*Same as trial 6*	-	[6]

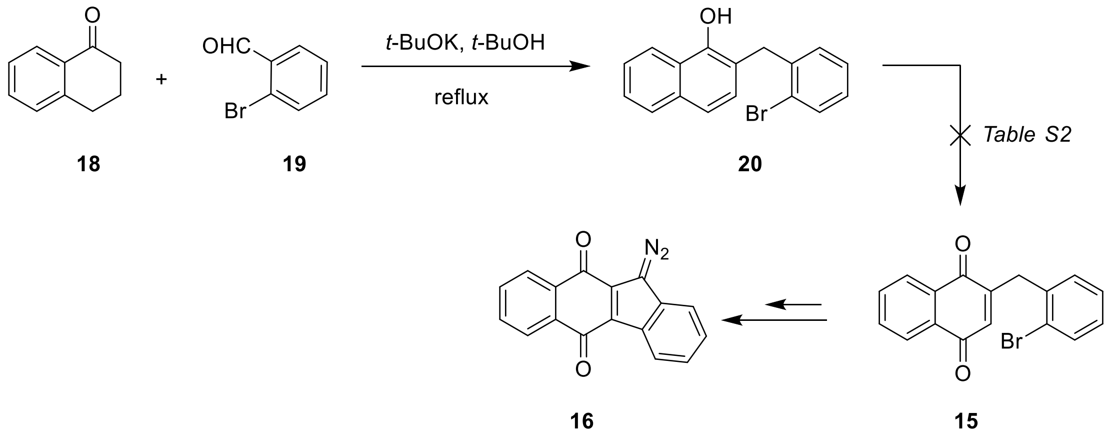

Scheme S2. Oxidative Dearomatization-Heck Cyclization Strategy

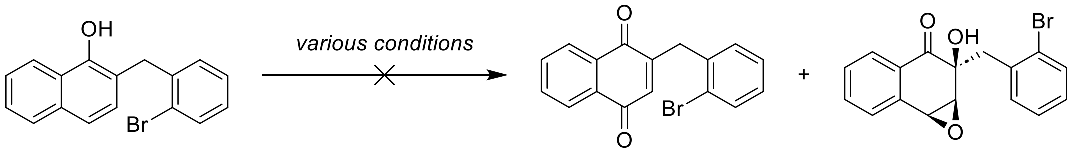

20 15 21

Table S2. Oxidative dearomatization trials.

Trial	Scale	Conditions	Observations	Yield	Ref.
1	94 mg	CAN (2 equiv.), MeCN/H_2O, rt	NR	-	-
2	60 mg	H_2O_2 (15 equiv.), I_2 (2 equiv.), H_2SO_4 (13 equiv.), MeOH, rt	NR	-	[8]
3	60 mg	*m*CPBA (2 equiv.), MeCN, rt	Obtained **21**	40%	[9]
4	60 mg	$NaNO_2$ (2 equiv.), 50% H_2SO_4 (25 equiv.), Dioxane, 80 °C	SM not consumed plus many new spots	-	[10]
5	116 mg	Fremy's Salt (2.5 equiv.), MeOH/H_2O, 0 °C	**15** coeluted with unknown impurity	45%	[11]

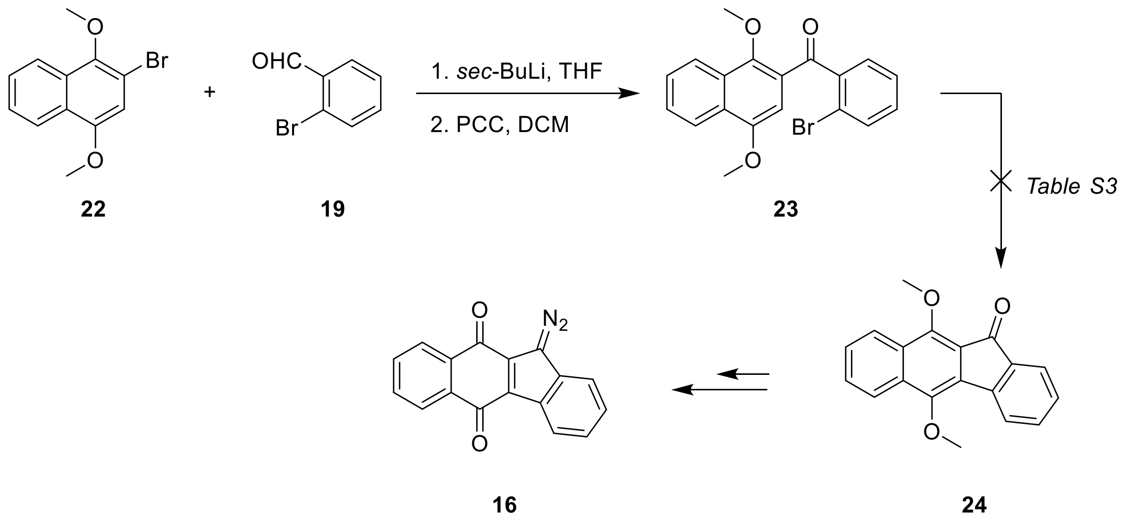

Scheme S3. Carbonyl Addition-Cyclization Strategy

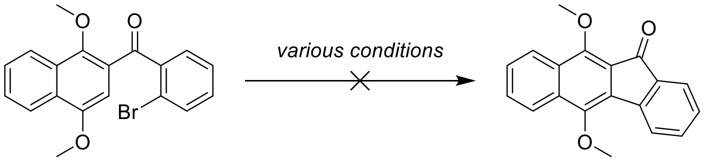

23 24

Table S3. Heck-type cyclization trials.

Trial	Scale	Conditions	Observations	Yield	Ref.
1	27 mg	$Pd(OAc)_2$ (10 mol %), PS-PPh_3 (20 mol%) Ag_2CO_3 (2 equiv.), DMA, 130 °C	NR	-	-
2	23 mg	$Pd(PPh_3)_4$ (6 mol %), DavePhos (10 mol %), KOAc (2 equiv.), K_2CO_3 (2 equiv.), DMA, 110 °C	Only protodehalogenation product was obtained	39%	-

Successful Synthesis of Model Natural Product Dideoxy Deethyl Prelomaivicitin

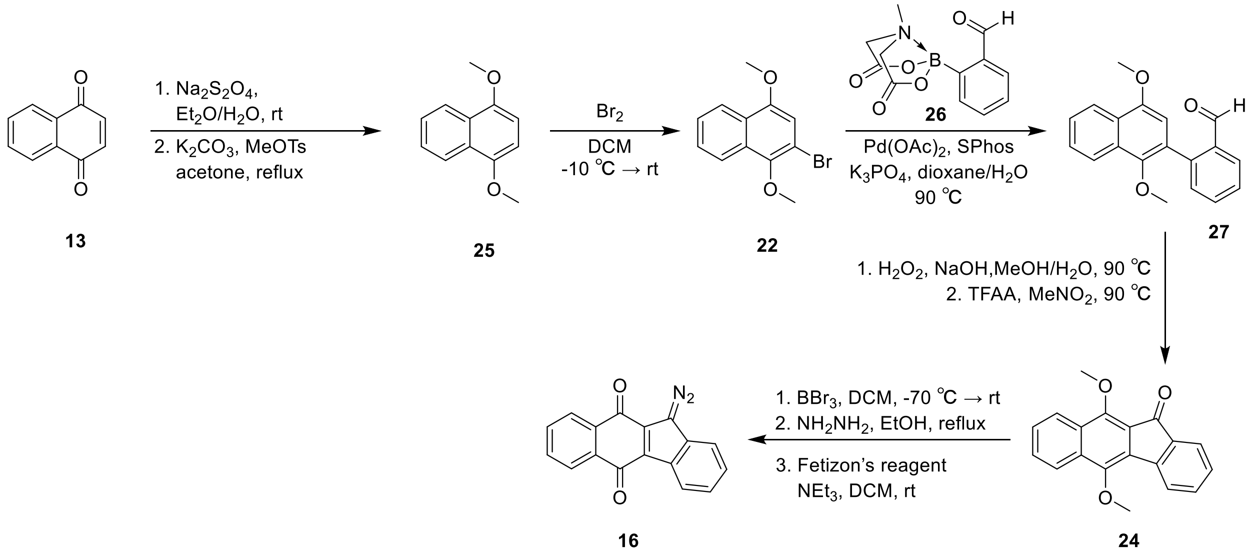

Scheme S4. Suzuki Coupling-Intramolecular Friedel-Crafts (IMFC) Acylation Strategy

Scheme S4 Step 1 & 2: Quinone Reduction-Methylation Sequence

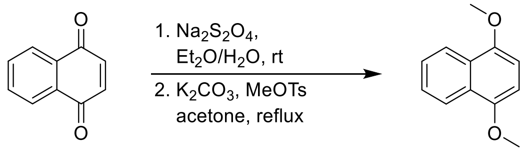

13 **25**

Comments Occasionally, in Step 1 the SM can still be seen by TLC despite additional reducing agent and rxn time being added. However, high yields can still be achieved despite this.

Procedure To rbf was added SM **13** (500 mg, 3.16 mmol, 1 equiv.) and Et_2O (40 mL). Added sodium hydrosulfite (4407 mg, 25 mmol, 8 equiv.) and then water (30 mL) and stirred the layers vigorously at rt overnight. TLC indicated new more polar UV active spot (Fig. S1a). Poured rxn into separatory funnel, separated layers, and washed aqueous layer with one more portion of ether. Combined organic layers, dried, and concentrated to afford brown solid which was dissolved in acetone (30 mL). To this was added K_2CO_3 (2620 mg, 19 mmol, 6 equiv.) followed by MeOTs (3 mL, 19 mmol, 6 equiv.). Refluxed the rxn for 12 h. TLC indicated new less polar spot that was fluorescent (Fig. S1b). TLCMS confirmed product **25** at 189.1 m/z. The rxn was diluted with water and the product was extracted with EtOAc twice. The organic layers were combined and washed with brine. The organic layer was dried, concentrated, and dry loaded onto silica gel column and purified with 1:19 EtOAc-Hex to yield **25** as 566 white solid (80% yield over two steps).

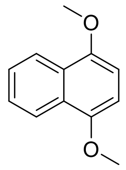

25 1,4-dimethoxynaphthalene (**25**)

^{1}H NMR (400 MHz, $CDCl_3$) δ 8.22-8.19 (m, 2H), 7.49-7.47 (m, 2H), 6.64 (s, 2H), 3.91 (s, 6H). The spectral data matched the literature.[12]

APCI-MS $[M+1]^+$ = 189.1

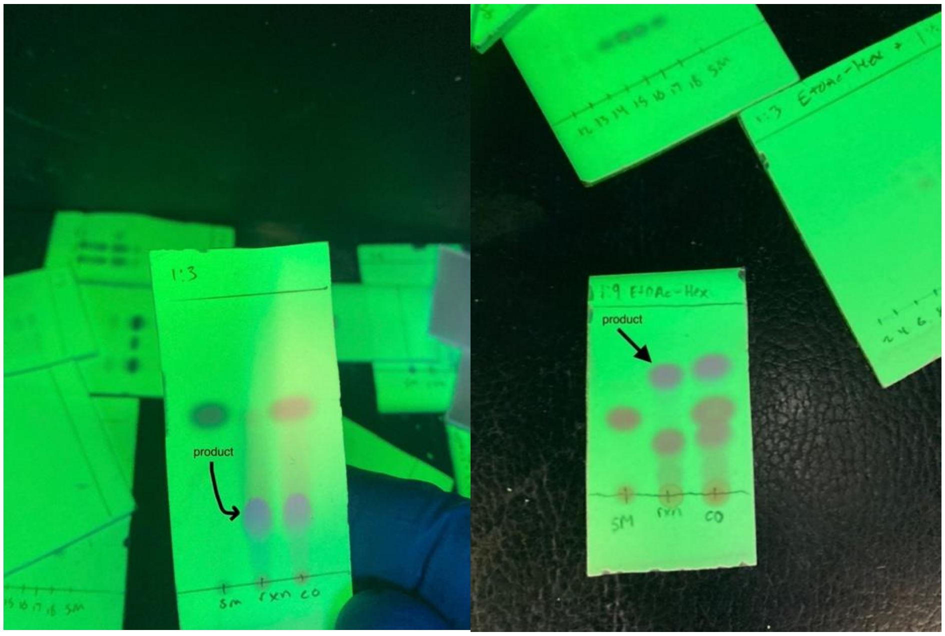

Figure S1. (a) TLC (UV 254 nm) quinone reduction rxn in 1:3 EtOAc-Hex eluent. Going from left: 1st lane: SM 13, 2nd lane: rxn, 3rd lane: cospot. The hydroquinone intermediate is marked with an arrow. (b) TLC (UV 254 nm) methylation rxn in 1:9 EtOAc-Hex eluent. Going from left: 1st lane: SM 13, 2nd lane: rxn, 3rd lane: cospot. The product 25 is marked with an arrow.

Scheme S4 Step 3: Bromination

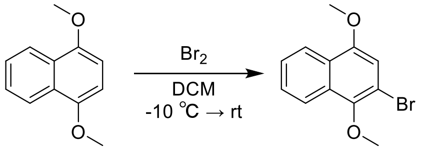

25 22

Comments *none*

Procedure To a screwcap vial containing SM **25** (486 mg, 2.58 mmol, 1 equiv.) was added sieve-dry DCM (6 mL). Cooled rxn to -10 °C using an ice-MeOH bath and added Br_2 (146 µL, 2.84 mmol, 1.1 equiv.) dropwise. After addition was complete, let the rxn slowly warm to rt over 1 h. TLC (Fig. S2) indicated rxn completion and appearance of new less polar UV active spot. Quenched rxn with water. Separated the layers and washed aqueous layer one more time with DCM. Combined organic fractions, dried, concentrated, and dry loaded onto silica gel column and eluted first with 100% Hex and then 2.5 % EtOAc in Hex to obtain **22** as 576 mg amber oil (83% yield).

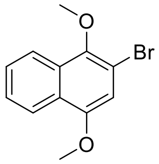

22 2-bromo-1,4-dimethoxynaphthalene (**22**)

^{1}H NMR (400 MHz, $CDCl_3$) δ 8.21-8.17 (m, 1H), 8.05-8.03 (dd, J = 7.6 Hz, 0.7 Hz, 1H), 7.55-7.45 (m, 2H), 6.86 (s, 1H), 3.93 (s, 3H), 3.92 (s, 3H). The spectral data matched the literature.[13]

APCI-MS $[M]^+$ = 266.0/268.0

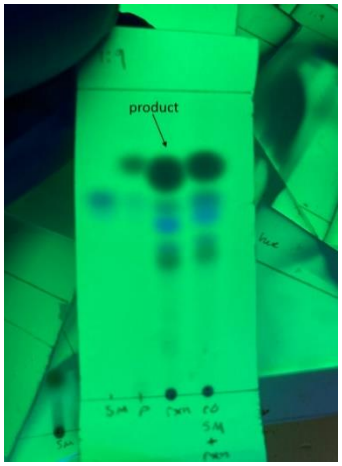

Figure S2. TLC (UV 254 nm) bromination rxn in 1:9 EtOAc-Hex eluent. Going from left: 1st lane: SM 25, 2nd lane: product 22 standard, 3rd lane: rxn, 4th lane: cospot. The product 22 is marked with an arrow.

Scheme S4 Step 4: Cross-Coupling

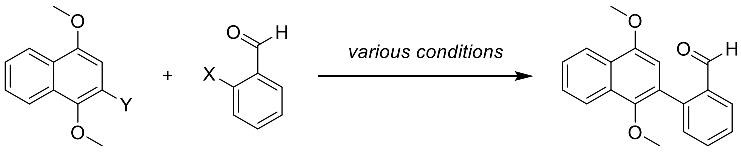

Table S4. Cross-coupling reaction optimization

Trial	Scale	Y	X	Conditions	Observations	Yield	Ref.
1	50 mg	Br	$SnBu_3$	$Pd(PPh_3)_4$ (10 mol%), CuI (20 mol%), THF, 70 °C	-	7%	-
2	65 mg	Br	$SnBu_3$	$Pd(OAc)_2$ (3 mol%), XPhos (6 mol%), CsF (2.2 eq.), DME, 80 °C	-	10%	14
3	50 mg	$SnBu_3$	Br	$Pd(OAc)_2$ (3 mol%), XPhos (6 mol%), CsF (2.2 eq.), THF, 80 °C	-	8%	14
4	69 mg	$SnBu_3$	I	$Pd(PPh_3)_4$ (15 mol%), CuI (10 mol%), CsF (2 eq.), DMF, 55 °C	-	30%	15
5	25 mg	$B(OH)_2$	Br	$Pd(OAc)_2$ (4 mol%), SPhos (6 mol%), K_3PO_4 (2 eq.), Toluene/H_2O, 100 °C	-	48%	16
6	57 mg	$B(OH)_2$	Br	$Pd(OAc)_2$ (4 mol%), SPhos (6 mol%), K_3PO_4 (2 eq.), Toluene/H_2O, 100 °C	-	57%	16
7	70 mg	Br	BMIDA	$Pd(OAc)_2$ (5 mol%), SPhos (10 mol%), Dioxane/3 M aq. K_3PO_4, 90 °C	-	90%	17
8	569 mg	Br	BMIDA	$Pd(OAc)_2$ (5 mol%), SPhos (10 mol%), Dioxane/3 M aq. K_3PO_4, 90 °C	-	65%	17
9	1591 mg	Br	BMIDA	$Pd(OAc)_2$ (5 mol%), SPhos (10 mol%), Dioxane/3 M aq. K_3PO_4, 90 °C	-	86%	17

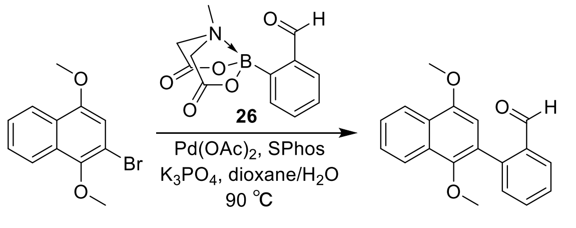

Comments Dioxane was freshly distilled from Na benzophenone prior to use in the rxn. DI water was degassed by bubbling nitrogen gas through it for 20 min. Flame drying the rxn vessel is not necessary as the rxn requires water.

Procedure To a microwave vial was added $Pd(OAc)_2$ (3.2 mg, 0.0145 mmol, 5 mol%), SPhos (12 mg, 0.029 mmol, 10 mol%) and 2-formylphenylboronic acid MIDA ester **26** (91 mg, 0.49 mmol, 1.7 eq.). The microwave vial was sealed with aluminum cap with septa and then flushed with N_2 gas. To degassed DI H_2O was added enough K_3PO_4 to prepare a 3 M solution. 2-bromo-1,4-dimethoxynaphthalene **22** (78 mg, 0.29 mmol, 1 eq.) was dissolved in distilled dioxane (4 mL) and soln. was added to the microwave vial containing the catalysts and **26**. Then injected the aq. 3M K_3PO_4 solution (0.8 mL, ~7 eq.) via syringe, which formed a biphasic 5:1 dioxane/H_2O rxn mixture. The rxn was flushed with N_2 again and the rxn was then heated in a 90 °C oil bath overnight for 19 h. TLC indicated rxn completion (Fig. S3) and TLCMS indicated product at 293 m/z. The rxn was diluted with brine and ether and stirred vigorously for several minutes. The layers were separated and the aqueous layer was washed two more times with ether. The organic layers were combined, dried, and concentrated. The crude was dry loaded onto a silica gel column and purified with 1:19 EtOAc-Hex to yield **27** as 76 mg yellow solid (90% yield).

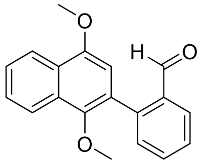

27 2-(1,4-dimethoxynaphthalen-2-yl)benzaldehyde (**27**)

^{1}H NMR (400 MHz, $CDCl_3$) δ 9.90 (s, 1H), 8.31-8.29 (dd, *J* = 7.8 Hz, 1.2 Hz, 1H), 8.15-8.13 (dd, *J* = 7.4 Hz, 1.1 Hz, 1H), 8.10-8.07 (dd, *J* = 8.3 Hz, 1.6 Hz, 1H), 7.73-7.69 (td, *J* = 7.6 Hz, 1.5 Hz, 1H), 7.62-7.53 (m, 4H), 6.74 (s, 1H), 4.01 (s, 3H), 3.40 (s, 3H). The spectral data matched the literature.[18]

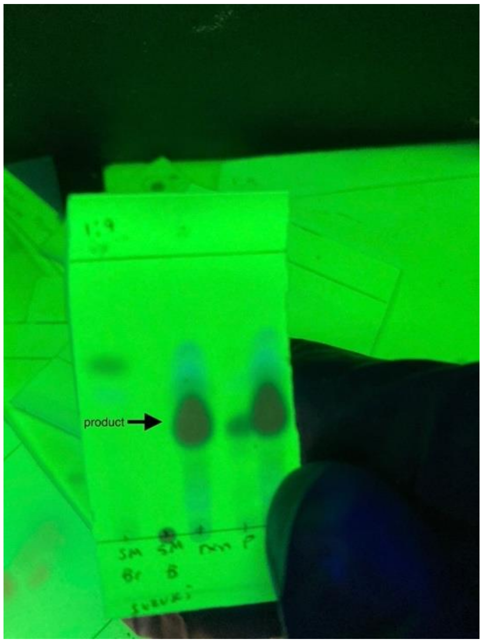

Figure S3. TLC (UV 254 nm) cross-coupling rxn in 1:9 EtOAc-Hex eluent. Going from left: 1st lane: SM 22, 2nd lane: SM 26, 3rd lane: rxn, 4th lane: product 27 standard, 5th: cospot (SM 22 and rxn).The product 27 is marked with an arrow.

Scheme S4 Step 5 & 6: Oxidation-Intramolecular Friedel-Crafts (IMFC) Acylation Sequence

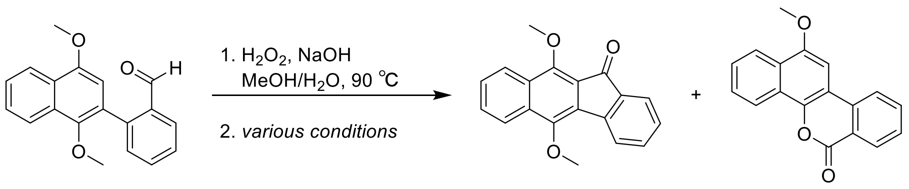

27 24 28

Table S5. Oxidation-IMFC acylation sequence reaction optimization.

Trial	Scale	Conditions	Observations	**24**:**28**	Yield[c]	Ref.
1	10 mg	1. NaOH (50 eq.), H_2O_2 (75 eq.), MeOH, 90 °C, µW<br>2. TFAA (5 eq.), molecular sieves, $MeNO_2$, 90 °C	Reactants were added together at rt and then placed in preheated oil bath	1:12[a]	-	19
2	52 mg	1. *same as Trial 1*<br>2. TFAA (5 eq.), molecular sieves, $EtNO_2$, 100 °C	SM and solvent were heated together in microwave to the desired temp, the vial was removed, TFAA was added, and the vial was placed back in the microwave	3:1[a]	-	19
3	10 mg	1. *same as Trial 1*<br>2. $(COCl)_2$ (7 eq.), DMF (cat.), $Fe(CO_2CF_3)_3$ (2 eq.), DCM, rt	By TLC, carboxylic acid did not appear to get converted to acyl chloride there appeared to be larger amount of **28** than **24**	-		20
4	10 mg	1. *same as Trial 1*<br>2. $(COCl)_2$ (7 eq.), DMF (cat.), $Fe(CO_2CF_3)_3$ (2 eq.), MeCN, 80 °C	TLC indicated carboxylic acid SM was consumed and that **24** and **28** formed before addition of Lewis acid	1:1[b]		20
5	10 mg	1. *same as Trial 1*<br>2. TFAA (10 eq.), molecular sieves, $EtNO_2$, 130 °C	*Decomp.*	-	-	19
6	4 mg	1. *same as Trial 1*<br>2. H_3PO_4 (excess), H_2O, 110 °C	Poor solubility of SM and no product detected by TLC	-	-	21
7	6 mg	1. *same as Trial 1*<br>2. P_2O_5/Al_2O_3 (1:1), DCE, 110 °C	-	2:1[b]	-	22

8	76 mg	1. *same as Trial 1*<br>2. TFAA (5 eq.), molecular sieves, $EtNO_2$, 105 °C	SM and solvent were placed in preheated oil bath and then TFAA was added to hot rxn mixture	-	48% (13%) (over two steps)	19
9	100 mg	1. *same as Trial 1*<br>2. TFAA (5 eq.), molecular sieves, $MeNO_2$, 90 °C	-	-	32% (n.d.) (over two steps)	19
10	1182 mg	1. *same as Trial 1*<br>2. TFAA (5 eq.), molecular sieves, $MeNO_2$:$EtNO_2$ (1:1), 90 °C	-	-	13% (n.d.) (over two steps)	19

[a] Ratio determined by NMR of crude.
[b] Ratio estimated by TLCMS.
[c] Yield outside parenthesis is for product **24**, yield within parenthesis is for product **28**.

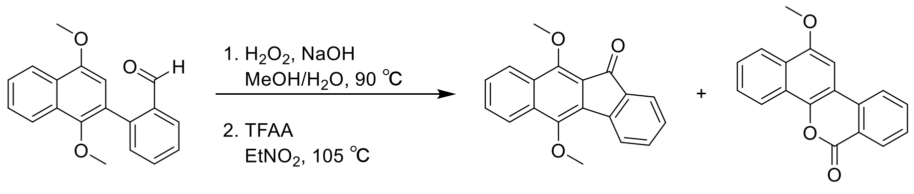

27 **24** **28**

Comments H_2O_2 oxidation of **27** generates extremely high pressures, which is exacerbated at higher scales. Biotage microwave vials with Biotage aluminum caps can withstand pressures of 15-20 bar before the vial shatters or the cap pops off and causes the rxn contents to be lost. IMFC yields tend to be higher with $EtNO_2$ solvent than $MeNO_2$, likely due to the higher boiling point of the former. However, the former solvent is a regulated substance and is not readily available from commercial suppliers. Therefore, the solvent can be substituted for the much more available $MeNO_2$ solvent, with the caveat of lower yields. Over the course of many trials, yields were observed to progressively drop but this issue was solved through distillation of TFAA or purchase of new TFAA. Our observations, along with a literature procedure for a similar reaction[19] show that mixing the SM with TFAA at room temp. and then immediately placing in a preheated oil bath leads to formation of higher amounts of undesired lactone **28**. Therefore, it is necessary to add TFAA to a hot rxn mixture in order to form the desired ketone **24**.

Procedure SM **27** (76 mg, 0.26 mmol, 1 eq.) was dissolved/suspended in MeOH (6 mL). Note, the SM does not readily dissolve in MeOH and sometimes requires heating and sonication to fully go into soln. More MeOH may be added or the undissolved SM slurry can be subjected to the rxn where it will likely dissolve at the refluxing temp. The SM soln./suspension is then transferred to a microwave vial and to it was added aqueous NaOH (15% m/v, 3.5 mL, 13 mmol, 50 eq.) followed by aqueous H_2O_2 (30% w/w, 2.2 mL, 19.5 mmol, 75 eq.). The vial was sealed, placed in Biotage microwave, and heated to 90 °C for 2 h. **CAUTION! PRESSURE EXPONENTIALLY INCREASES IN THE FIRST COUPLE MINUTES OF THE RXN. FOR SAFETY, WHEN THE PRESSURE OF THE RXN REACHED 10 BAR, A BLEED NEEDLE WAS INSERTED INTO THE RXN AND WAS CONTINUSOULY DONE SO**

UNTIL THE PRESSURE FLATLINED AT ~9-10 BAR. Note, if rxn is not homogenous after reaching 90 °C, more MeOH should be added or else not all the SM will be consumed. TLC indicated rxn completion and appearance of more polar spot (Fig. S4). TLCMS indicated carboxylic acid intermediate at 307 m/z. The rxn was acidified to pH 3-4 using 3 M HCl, causing a precipitate to form. $CHCl_3$ was added, forming two layers, and the biphasic rxn was stirred for several minutes (cloudiness disappeared). Separated the layers and washed aqueous layer two more times with $CHCl_3$. Combined the organic layers, dried, and concentrated down to 66 mg orange solid, which was subjected to the next rxn without further purification. To vial that contained carboxylic acid intermediate (66 mg, 0.21 mmol, 1 eq.) was added powdered molecular sieves (100 mg) and $EtNO_2$ (10 mL), bringing SM to ~0.02 M concentration. The rxn was placed in a preheated 105 °C oil bath and stirred for 5 min. before adding TFAA (147 µL, 1 mmol, 5 eq.) all at once (a small violent rxn occurred). The rxn was stirred at 105 °C for 30 min and TLC indicated rxn completion through appearance of two new less polar fluorescent spots (one yellow and one blue) (Fig. S5). TLCMS indicated the yellow fluorescent spot was **24** at 291.2 m/z and the blue fluorescent spot was **28** at 277.1 m/z. The rxn was diluted with water and washed with two portions of EtOAc. Combined the EtOAc layers and washed with brine twice. Dried the organic layer, concentrated, and dry loaded onto a silica gel column and purified with 100% hexanes, then 1:19 EtOAc-Hex, and then 1:9 EtOAc-Hex (**24** came out with 1:19 and **28** came out with 1:9). Obtained 36 mg of **24** as yellow needles (48% yield over two steps) and 9 mg of **28** as an orange solid (13% yield).

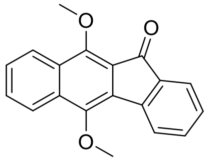

24 5,10-dimethoxy-11H-benzo[b]fluoren-11-one (**24**)

^{1}H NMR (400 MHz, $CDCl_3$) δ 8.32-8.30 (d, *J* = 8.2 Hz, 1H), 8.06-8.04 (d, *J* = 8.2 Hz, 1H), 8.02-8.00 (d, *J* = 7.6 Hz, 1H), 7.76-7.74 (d, *J* = 7.4 Hz, 1H), 7.64-7.55 (m, 2H), 7.52-7.48 (td, *J* = 8.0 Hz, 0.9 Hz, 1H), 7.36-7.32 (t, *J* = 7.4 Hz, 1H), 4.30 (s, 3H), 4.01 (s, 3H). ^{13}C NMR (100 MHz, $CDCl_3$) δ 190.33, 153.93, 146.98, 142.62, 136.17, 134.79, 133.65, 131.07, 129.63, 128.63, 127.71, 127.00, 125.67, 124.40, 124.05, 122.60, 119.60, 63.21, 61.31.

APCI-MS $[M+1]^+$ = 291.2

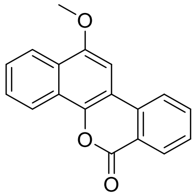

28 17-methoxy-9-oxatetracyclo[8.8.0.0^{2,7}.0^{11,16}]octadeca-1(18),2,4,6,10,12,14,16-octaen-8-one (**28**)

^{1}H NMR (400 MHz, $CDCl_3$) δ 8.55-8.53 (d, *J* = 8.5 Hz, 1H), 8.48-8.46 (dd, *J* = 7.7 Hz, 1.0 Hz, 1H), 8.28-8.26 (d, *J* = 8.5 Hz, 1H), 8.14-8.12 (d, *J* = 8.4 Hz, 1H), 7.89-7.84 (td, *J* = 7.2 Hz, 1.4 Hz, 1H), 7.68-7.57 (m, 3H), 7.25 (s, 1H), 4.11 (s, 3H). The spectral data matched the literature.[18]

APCI-MS $[M+1]^+ = 277.1$

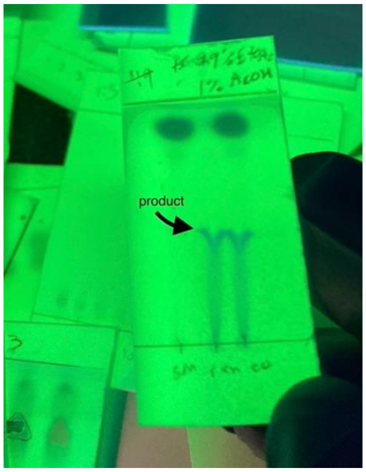

Figure S4. TLC (UV 254 nm) aldehyde oxidation rxn in 99:1 EtOAc-AcOH eluent. Going from left: 1st lane: SM 27, 2nd lane: rxn, 3rd lane: cospot. The carboxylic acid intermediate is marked with an arrow.

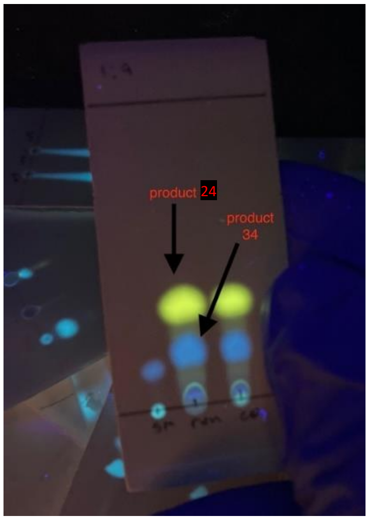

Figure S5. TLC (UV 365 nm) IMFC rxn in 1:9 EtOAc-Hex eluent. Going from left: 1st lane: SM (carboxylic acid intermediate), 2nd lane: rxn, 3rd lane: cospot. Product 24 and 28 are marked with arrows.

Scheme S4 Step 7-9: Final Sequence: Demethylation, Hydrazine Condensation, Fetizon Oxidation

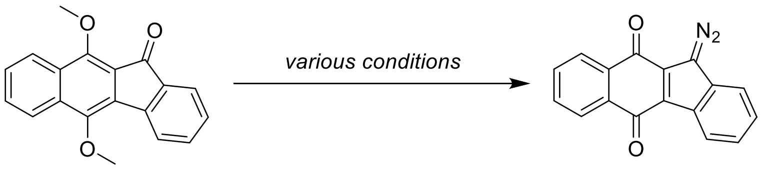

Table S6. Final sequence optimization

Trial	Scale	Conditions	Observations	Yield	Ref.
1	36 mg	1. TsNHNH$_2$ (1.7 eq.), HCl (cat.), EtOH, 90 °C, μW<br>2. CAN (3 eq.), PBS buffer, MeCN, 0 °C	Step 1 was difficult to push to completion, Step 2 reached completion but TLCMS did not confirm product and new spots shared similar mass to condensation product (not shown)	14%[a]	[19]
2	34 mg	1. TsNHNH$_2$ (10 eq.), H_2SO_4 (aq.) (2 eq.), *i*-PrOH, rt<br>2. DMP (5 eq.), DCM, rt	Step 1 was successful, albeit in low yield, but Step 2 reformed **24** as shown by TLC and TLCMS	15%[a]	[23]
3	20 mg	1. $NH_2NH_2 \cdot H_2O$ (10 eq.), AcOH/EtOH (1:10), 95 °C<br>2. DMP (5 eq.), DCM, rt	Step 1 was messy by TLC so did not attempt Step 2	-	-
4	32 mg	1. TsNHNH$_2$ (2.5 eq.), H_2SO_4, molecular sieves, EtOH, rt<br>2. 50% w/w NaOH aq., dioxane, 65 °C<br>3. CAN (3 eq.), PBS buffer, MeCN, -10 °C	Step 1 was successful, Step 2 appeared to be successful by TLCMS but workup seemed to indicate decomp. by TLC, Step 3 appeared to result in decomp. or its possible product decomposed after Step 2 workup	46%[a]	[23]
5	32 mg	CAN (6 eq.), PBS buffer, MeCN, 0 °C	SM was consumed and one new spot formed but TLCMS did not indicate product and crude NMR did not indicate product	-	-
6	38 mg	1. BBr_3 (6 eq.), DCM, -40 °C<br>2. TsNHNH$_2$ (2.5 eq.), H_2SO_4 (2.5 eq.), molecular sieves, EtOH, rt to 90 °C<br>3. Fetizon's reagent (2.5 eq.), NEt_3 (15 eq.), acetone	Step 1 worked by TLCMS, Step 2 was not confirmed by TLCMS, Step 3 seemed to indicate desired product **16** by TLCMS	-	[24]

7	25 mg	1. $TsNHNH_2$ (5 eq.), H_2SO_4 (2.5 eq.), molecular sieves, EtOH, rt<br>2. BBr_3 (6 eq.), DCM, -40 °C<br>3. Fetizon's reagent (2.5 eq.), NEt_3 (15 eq.), acetone	Step 1 worked by TLCMS, Step 2 did not work and gave many spots by TLC, Step 3 was attempted anyways but was confirmed by TLCMS to not have any product	-	24
8	29 mg	1. $NH_2NH_2 \cdot H_2O$ (70 eq.), EtOH, 90 °C, µW<br>2. BBr_3 (6 eq.), DCM, -40 °C	Step 1 was successful by TLCMS, Step 2 showed lots of spots by TLC	-	13
9	32 mg	1. BBr_3 (3 eq.), DCM, -70 °C to rt<br>2. N_2HNH_2 (anhyd.) (70 eq.), 90 °C<br>3. Fetizon's reagent (13 eq.), NEt_3 (20 eq.), DCM	-	40% (over three steps)	13

[a] Yield for condensation step only.

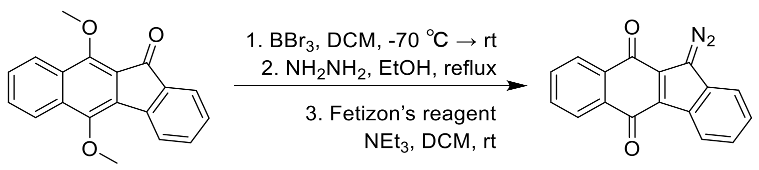

24 **16**

Comments Hydrazine monohydrate was made anhydrous by refluxing over an equal weight of NaOH (i.e. 5 g of hydrazine monohydrate requires 5 g of NaOH) for 1.5 h in 130 °C oil bath and then distilled under nitrogen. Fetizon's reagent was prepared by washing Celite (4.5 g) with 100 mL of 10% HCl in methanol (prepared by adding 10 mL of 37% aq. HCl to 90 mL MeOH), followed by 2x100 mL DI water washes. The Celite was then flame dried under vacuum until all water evaporated. To the Celite was added a soln. of 5 g $AgNO_3$ in 50 mL water. Began stirring at rt and then a solution of 1.67 g Na_2CO_3 in 45 mL water was added slowly, which caused a yellow precipitate. The rxn was then allowed to stir for 10 min and then was filtered. The filter cake was then dried in a rotavap overnight in a 55 °C water bath under a pressure of <100 mbar. Quantitative formation of silver carbonate was assumed, leading to an assumed weight loading of 50 wt% Ag_2CO_3 on the purified Celite. Triethylamine was kept over KOH pellets at rt.

Procedure To a flame dried vial screwcap vial was added SM **24** (32 mg, 0.11 mmol, 1 eq.) that was dissolved in sieve-dried DCM (2 mL). Cooled to -70 °C in dry ice MeOH bath and flushed with N_2 gas. Added BBr_3 (5 M in DCM, 66 µL, 0.33 mmol, 3 eq.) dropwise and soln. immediately turned dark red. Once addition was complete, allowed the rxn to warm up to rt over course of 1 h. TLC indicated rxn completion and appearance of new, more polar fluorescent spot

that smeared on the plate (Fig. S6) and TLCMS confirmed hydroquinone intermediate at 263.1 m/z. Added water to rxn and then added solid sodium bicarbonate until aqueous layer reached neutral pH. Separated layers and then washed the organic layer with 5 M sodium bisulfite. Separated layers and washed aqueous layer with DCM. Combined organic layers, dried, and concentrated down to red solid and assumed quantitative conversion. To flame dried microwave vial was added hydroquinone intermediate obtained from last step (0.11 mmol, 1 eq.) that was dissolved in anhydrous ethanol (3 mL) that had been degassed for 15 min with N_2 gas. The soln. was yellow. Sealed and purged rxn with N_2 gas. Hydrazine monohydrate (246 µL, 7.7 mmol, 70 eq.) was added hydrazine via syringe and then the rxn was heated at 90 °C for 1 h in the microwave. The soln. was now black. TLC indicated rxn completion and a new less polar spot that had lost the fluorescence of the SM (Fig. S7). The spot also had red visible color. TLCMS confirmed hydrazone intermediate at 275 m/z. Concentrated rxn and then added another 3 mL of EtOH and concentrated again (to ensure removal of hydrazine). Assumed quantitative conversion. To vial containing hydrazone intermediate obtained from last step (0.11 mmol, 1 eq.) was added sieve dried DCM (5 mL). Then added NEt_3 (308 µL, 2.2 mmol, 20 eq.) followed by Fetizon's reagent (50 wt% on Celite, 788 mg, 1.43 mmol, 13 eq.). Stirred vigorously at rt for 10 min. TLC did not indicate rxn completion as SM was still observed but did indicate a new, less polar spot that had yellow color. Added another 13 eq. Fetizon's reagent and stirred for another 10 min at rt. TLC looked the same. Added another 13 eq. and stirred at rt overnight. TLC indicated rxn completion (Fig. S8). TLCMS indicated product at 286.1 m/z (this results from molecular ion $[M+1]^+$ losing N_2 and then forming MeCN adduct). Filtered rxn through celite and eluted with DCM. Concentrated the filtrate in a rotavap and dry loaded onto silica gel column and purified with 1:9 EtOAc-Hex. Obtained **16** as 12 mg red solid (40% yield over 3 steps).

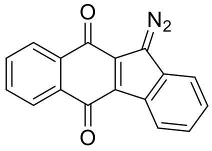

16 1,7-Dideoxy-3-demethylprekinamycin

11-(-λ^5-diazynylidene)-4bH,5H,10H,10aH,11H-benzo[b]fluorene-5,10-dione (**16**)

^{1}H NMR (400 MHz, $CDCl_3$) δ 8.52-8.50 (m, 1H), 8.21-8.19 (dd, *J* = 7.7 Hz, 1.4 Hz, 1H), 8.13-8.10 (dd, *J* = 7.3 Hz, 1.2 Hz, 1H), 7.75-7.66 (m, 2H), 7.54-7.51 (m, 1H), 7.44-7.39 (m, 2H). ^{13}C NMR (100 MHz, $CDCl_3$) δ 180.84, 180.18, 134.52, 134.37, 134.01, 133.94, 133.30, 132.92, 130.48, 127.34, 127.00, 126.81, 126.75, 126.17, 126.03, 118.50.

FT-IR (neat, ATR) 2100, 1650 cm^{-1}.

APCI-MS $[M+1-N_2]^+$ = 245.0

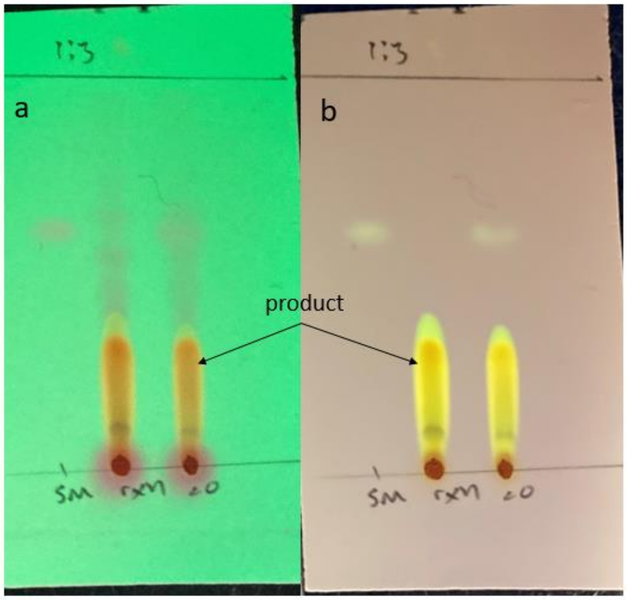

Figure S6. (a) TLC (UV 254 nm) BBr_3 demethylation rxn in 1:3 EtOAc-Hex eluent. Going from left: 1st lane: SM 24, 2nd lane: rxn, 3rd lane: cospot. The product is marked with an arrow. (b) TLC (UV 365 nm) BBr_3 demethylation rxn in 1:3 EtOAc-Hex eluent. Going from left: 1st lane: SM 24, 2nd lane: rxn, 3rd lane: cospot. The product is marked with an arrow.

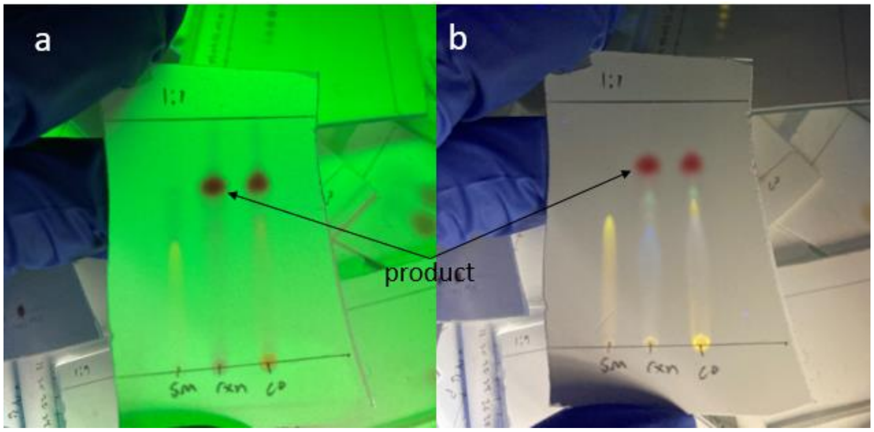

Figure S7. (a) TLC (UV 254 nm) hydrazine condensation in 1:1 EtOAc-Hex eluent. Going from left: 1st lane: SM (hydroquinone intermediate), 2nd lane: rxn, 3rd lane: cospot. The product is marked with an arrow. (b) TLC (UV 365 nm) hydrazine condensation in 1:1 EtOAc-Hex eluent. Going from left: 1st lane: SM (hydroquinone intermediate), 2nd lane: rxn, 3rd lane: cospot. The product is marked with an arrow.

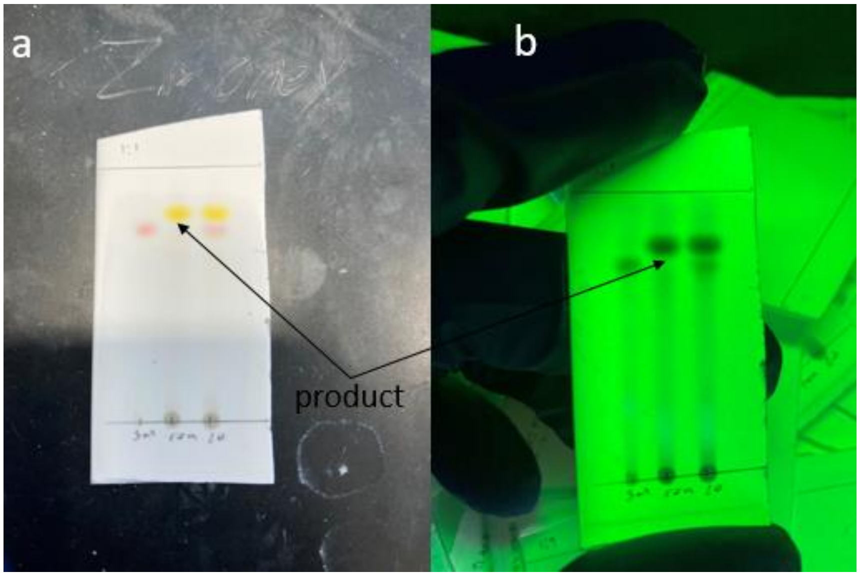

Figure S8. (a) TLC (no visualization) Fetizon oxidation in 1:1 EtOAc-Hex eluent. Going from left: 1st lane: SM (hydrazone intermediate), 2nd lane: rxn, 3rd lane: cospot. The product 16 is marked with an arrow. (b) TLC (UV 254 nm) Fetizon oxidation in 1:1 EtOAc-Hex eluent. Going from left: 1st lane: SM (hydrazone intermediate), 2nd lane: rxn, 3rd lane: cospot. The product 16 is marked with an arrow.

Successful Total Synthesis of Homoseongomycin

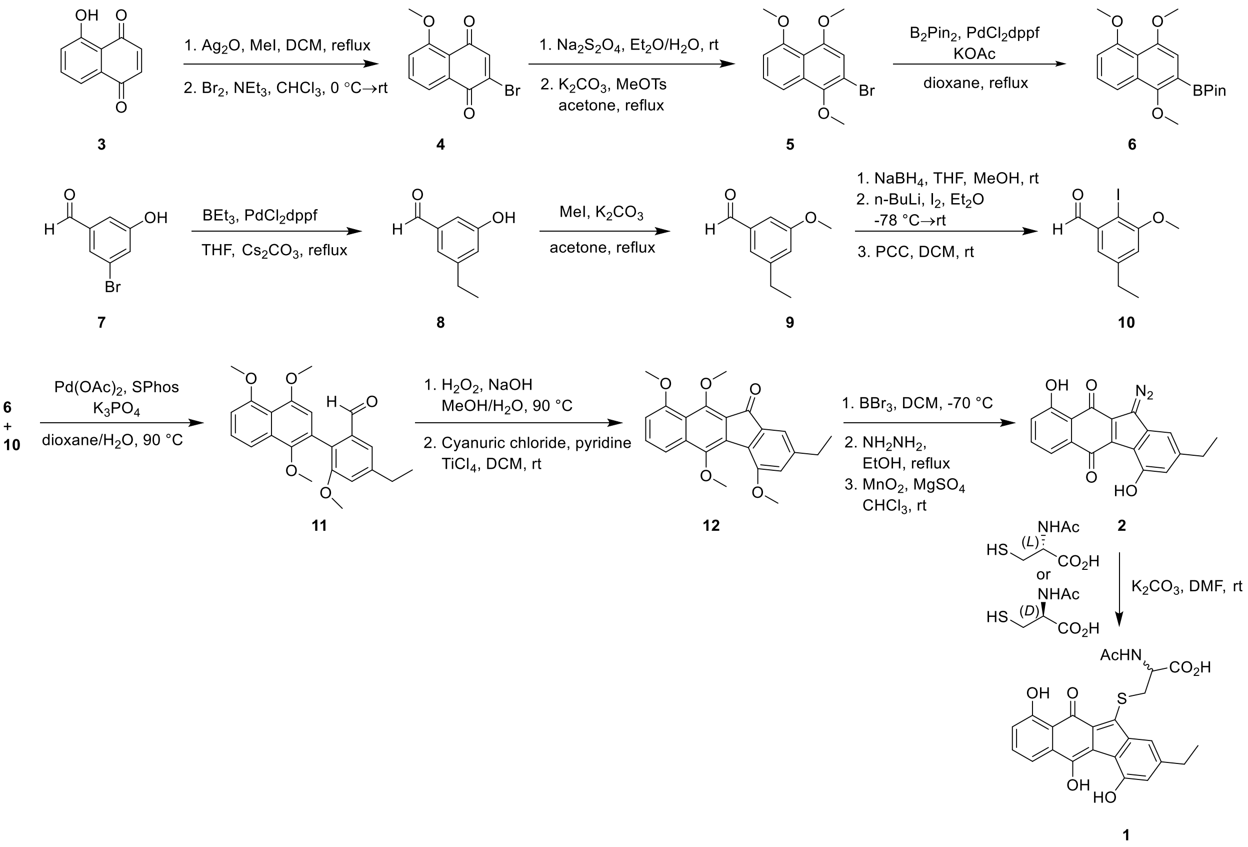

Scheme S5. Suzuki Coupling-Intramolecular Friedel-Crafts (IMFC) Acylation Strategy

Scheme S5 Step 1 & 2: Methylation and Bromination of Juglone

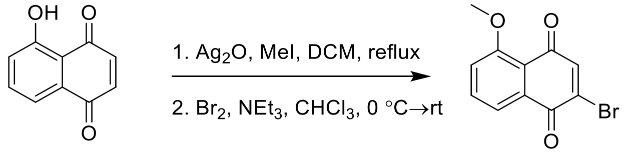

3 **4**

Comments Alkene bromination of juglone (**3**) was a low yielding rxn (11%) that suffered from poor conversion. Addition of more Br_2 would likely lead to mixture of brominated congeners. Phenol protection of juglone with methyl group followed by bromination leads to much higher yields. At small scale (<~250 mg) the rxn reaches completion at rt. Larger scales (>2 g) require reflux to reach completion.

Ag_2O was prepared by dissolving NaOH (2400 mg, 60 mmol, 1 eq.) in 100 mL DI water. added $AgNO_3$ (10,200 mg, 60 mmol, 1 eq.) was dissolved in 50 mL DI water and this soln. was added all at once to NaOH solution. The rxn immediately formed brown precipitate. Stirred for 10 min. at rt, filtered, and then vacuum dried solid, and stored in amber bottle. Yield was quantitative.

For Step 2, EtOAc-Hex eluent does not separate SM from product which is why DCM-Hex solvent system was used for purification. At larger scales (~3 g), the silica gel purification step can be skipped and the crude **4** can be subjected to the next rxn.

Procedure To flame-dried rbf was added juglone (**3**) (1000 mg, 5.74 mmol, 1 eq.), which was then dissolved in sieve-dried DCM (50 mL). Methyl iodide (1.1 mL, 17.22 mmol, 3 eq.) was added followed by Ag_2O (2660 mg, 11.5 mmol, 2 eq.). The rxn was then refluxed for 48 h in 65 °C oil bath. TLC indicated rxn completion and appearance of new, more polar spot (Fig. S9), which was confirmed by TLCMS to be product at 188.6 m/z. The rxn was filtered through celite and eluted with DCM. The filtrate was then concentrated down to methyljuglone intermediate as a 994 mg orange solid (92% yield) and used in the next step without further purification. To rbf was added methyljuglone intermediate (994 mg, 5.3 mmol, 1 eq.) obtained from the previous step, which was dissolved in $CHCl_3$ (50 mL) and then cooled to 0 °C in an ice-water bath. Br_2 (356 µL, 6.9 mmol, 1.3 eq.) was added dropwise and the rxn was allowed to stir for 10 min. at 0 °C. NEt_3 (1.2 mL, 8.5 mmol, 1.6 eq.) was added all at once, which produced white fumes, and the rxn was then allowed to warm to rt overnight. TLC indicated rxn completion and appearance of new, less polar spot (Fig. S10). TLCMS confirmed product **4** at 266.8 and 268.8 m/z. To the rxn was added water. Stirred, separated layers, dried organic layer, concentrated, and dry loaded onto silica gel column and purified with 3:1 DCM-Hex followed by 100% DCM to give **4** as 1220 mg orange solid (86% yield).

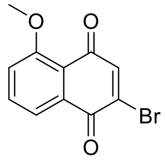

4 2-bromo-5-methoxy-1,4-dihydronaphthalene-1,4-dione (**4**)

^{1}H NMR (400 MHz, $CDCl_3$) δ 7.85-7.82 (dd, J = 7.7 Hz, 1.0 Hz, 1H), 7.72-7.68 (t, J = 8.4 Hz, 1H), 7.41 (s, 1H), 7.36-7.33 (dd, J = 8.5 Hz, 0.6 Hz, 1H), 4.02 (s, 3H). The spectral data matched the literature.[18]

APCI-MS $[M+1]^+$ = 266.6/268.6

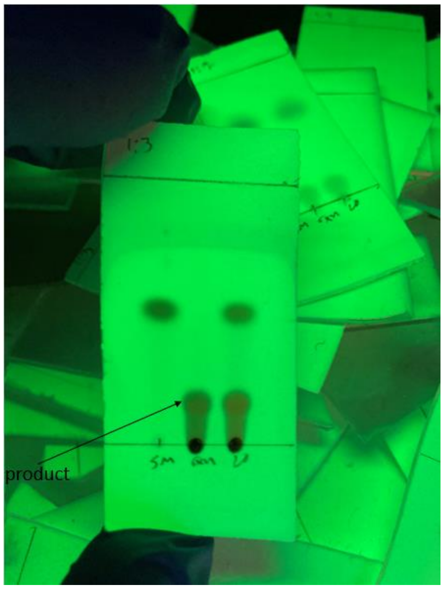

Figure S9. ***TLC (UV 254 nm) methylation rxn in 1:3 EtOAc-Hex eluent. Going from left: 1st lane: SM*** *3*, ***2nd lane: rxn, 3rd lane: cospot. The methyljuglone intermediate is marked with an arrow.***

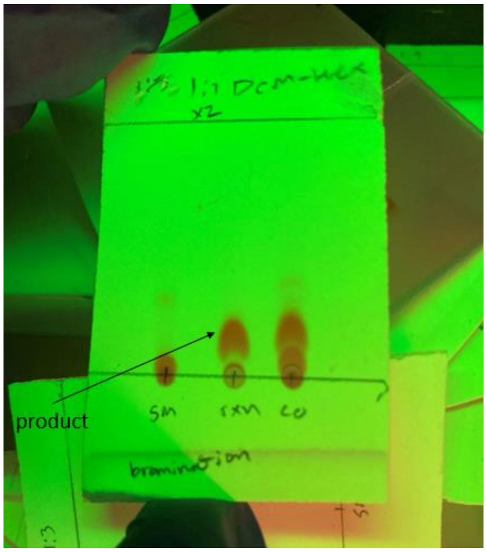

Figure S10. ***TLC (UV 254 nm) bromination rxn in 1:1 DCM-Hex eluent (doubly developed). Going from left: 1st lane: SM (methyljuglone intermediate), 2nd lane: rxn, 3rd lane: cospot. The product*** *4* ***is marked with an arrow.***

Scheme S5 Step 3 & 4: Quinone Reduction-Methylation Sequence

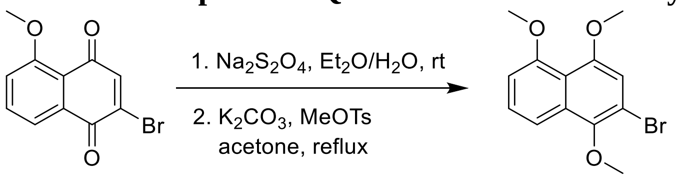

4 **5**

Comments At larger scales, the product can be purified and separated from residual MeOTs using an alternative procedure than what is listed below: After the methylation rxn is complete, the rxn can be filtered to remove the K_2CO_3. The filtrate can be concentrated and then taken up in DCM. Residual MeOTs may be reacted with 1,3-diaminopropane in a 2:1 ratio by refluxing for 8 h and disappearance of MeOTs can be monitored by TLC. The rxn can then filtered through plug of silica using DCM and the filtrate can be concentrated to afford **5**, which can be subjected to the next rxn without further purification.

Procedure To rbf was added SM **4** (1220 mg, 4.57 mmol, 1 equiv.) and Et_2O (150 mL). Added sodium hydrosulfite (6364 mg, 36.55 mmol, 8 equiv.) and then water (100 mL) and stirred the layers vigorously at rt overnight. TLC indicated new, less polar UV active spot (Fig. S11a). TLCMS showed this new spot had same exact mass as SM at 266.8 and 268.8 m/z, likely due to oxidation of hydroquinone intermediate back to quinone **4** at the MS source. Poured rxn into separatory funnel, separated layers, and washed aqueous layer with one more portion of ether. Combined organic layers, dried, and concentrated to afford brown solid which was dissolved in acetone (100 mL). To this was added K_2CO_3 (3789 mg, 27.42 mmol, 6 equiv.) followed by MeOTs (4.2 mL, 27.42 mmol, 6 equiv.). Refluxed the rxn for 12 h in 85 °C oil bath. TLC indicated rxn completion and two new, less polar spots (Fig. S11b). TLCMS confirmed top spot was product **5** at 296.8 and 298.8 m/z and bottom spot was leftover MeOTs. Concentrated rxn in rotavap and then diluted with EtOAc and water. Stirred an separated the layers and washed aqueous layer two more times with EtOAc. Combined EtOAc fractions, dried, concentrated, and dry loaded onto silica gel column and purified first with 1:19 EtOAc-Hex and then 1:9 EtOAc. Collection of top spot afforded **5** as 643 mg beige solid (47% yield over two steps).

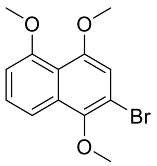

5 2-bromo-1,4,5-trimethoxynaphthalene (**5**)

^{1}H NMR (400 MHz, $CDCl_3$) δ 7.70-7.68 (dd, *J* = 8.4 Hz, 0.7 Hz, 1H), 7.46-7.42 (t, *J* = 8.1 Hz, 1H), 6.91-6.89 (m, 2H), 3.97 (s, 3H), 3.94 (s, 3H), 3.92 (s, 3H). The spectral data matched the literature.[18]

APCI-MS $[M+1]^+$ = 296.8/298.8

HRESIMS *m/z* 297.0123, $[M+H]^+$ (calc'd for $C_{13}H_{14}BrO_3$, 297.0126)

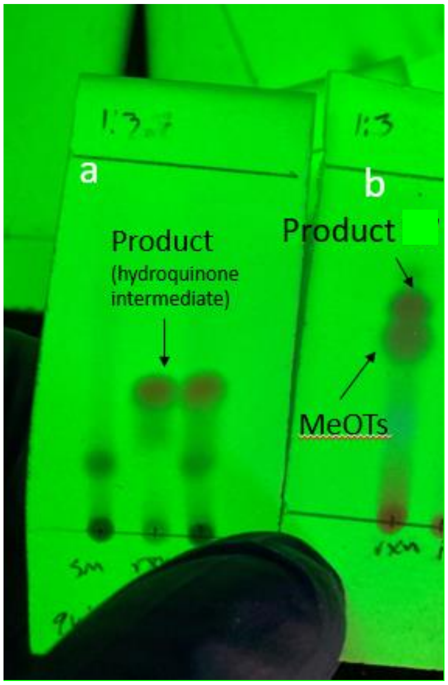

Figure S11. (a) TLC (UV 254 nm) quinone reduction rxn in 1:3 EtOAc-Hex eluent. Going from left: 1st lane: SM 4, 2nd lane: rxn, 3rd lane: cospot. The hydroquinone intermediate is marked with an arrow. (b) TLC (UV 254 nm) methylation rxn in 1:3 EtOAc-Hex eluent. The product 5 is marked with an arrow.

Scheme S5 Step 5: Suzuki Borylation

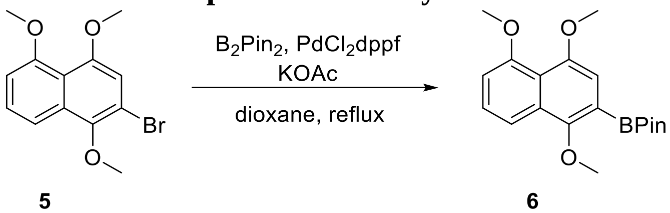

Comments The rxn was attempted in DMF solvent with $Pd(OAc)_2$ catalyst but little conversion of SM to product occurred after 12 h at 80 °C. KOAc was dehydrated by flame-drying under vacuum until the solid melted, which was then allowed to cool back down to rt under vacuum. This process was repeated a second time. The KOAc was then used right away. Dioxane was freshly distilled from Na benzophenone before use. Sometimes the compound was isolated as a white solid or as an amber oil, even after extended pumping. Yield of subsequent Suzuki coupling does not appear to be affected by this.

The synthetic steps from **3** to **6** may be telescoped at larger scales (as described above in the comments section for each of those reactions) with no formal chromatographic purification except for the last step from **5** to **6**.

Procedure To flame dried microwave vial was added $PdCl_2dppf{\cdot}CH_2Cl_2$ (176 mg, 0.216 mmol, 10 mol%), bis(pinacolato)diboron (1208 mg, 4.76 mmol, 2 eq.), and KOAc (1270 mg, 12.96 mmol, 6 eq.). The rxn was sealed and purged with N_2 gas. Distilled dioxane (20 mL) was used to dissolve the SM **5** (643 mg, 2.16 mmol, 1 eq.) and this soln. was then added to the microwave vial via syringe. The soln. was red. The rxn was purged with N_2 gas again and then heated in a 100 °C oil bath for 18 h. The soln. was now black. TLC indicated rxn completion and formation of two new, more polar spots, one of which had blue color under UV 254 nm light (Fig. S12). TLCMS indicated the top spot was protodehalogenated byproduct of **5** at 218.6 m/z and the bottom, blue colored spot was desired product **6** at 345.2 m/z. The rxn was concentrated, dry loaded onto silica gel column, and purified with 1:19 EtOAc-Hex and then 1:9 EtOAc-Hex. Collection of bottom spot afforded 650 mg amber oil (87% yield).

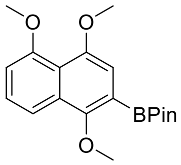

6 4,4,5,5-tetramethyl-2-(1,4,5-trimethoxynaphthalen-2-yl)-1,3,2-dioxaborolane (**6**)

^{1}H NMR (400 MHz, $CDCl_3$) δ 7.81-7.79 (dd, *J* = 8.4 Hz, 1.0 Hz, 1H), 7.41-7.37 (t, *J* = 7.9 Hz, 1H), 7.07 (s, 1H), 6.92-6.90 (d, *J* = 7.4 Hz, 1H), 3.98 (s, 3H), 3.96 (s, 3H), 3.92 (s, 3H), 1.40 (s, 12H). ^{13}C NMR (100 MHz, $CDCl_3$) δ 157.24, 157.13, 152.56, 131.48, 126.34, 120.48, 115.75, 111.10, 107.87, 83.71, 83.50, 63.52, 57.03, 56.58, 24.92.

APCI-MS $[M+1]^+$ = 345.2

HRESIMS *m/z* 367.1709, $[M+Na]^+$ (calc'd for $C_{19}H_{25}BO_5Na$, 367.1693)

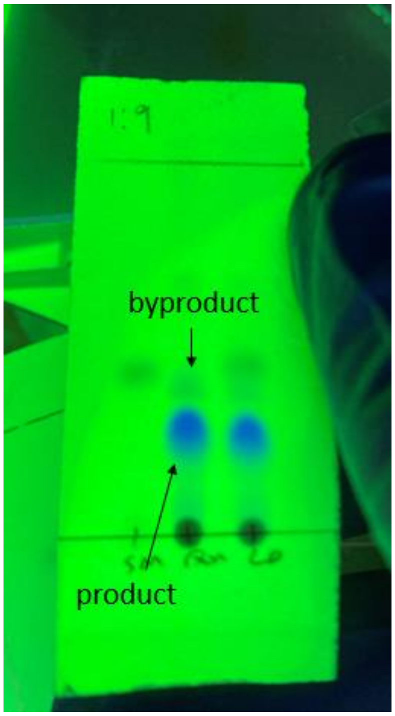

Figure S12. TLC (UV 254 nm) Suzuki borylation rxn in 1:9 EtOAc-Hex eluent. Going from left: 1st lane: SM 5, 2nd lane: rxn, 3rd lane: cospot. The product 6 is marked with an arrow as well as protodehalogenated byproduct.

Scheme S5 Step 6: Pd-catalyzed Ethylation

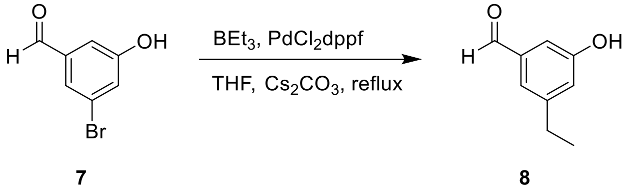

Comments THF was freshly distilled from Na benzophenone before use. Methylation of the phenol in **7** followed by Pd-catalyzed ethylation results in much lower yield than the reverse sequence (i.e. Pd-catalyzed ethylation of **7** and then phenol methylation on **8**). Note, skipping chromatography and subjecting crude **8** to methylation conditions described below does not successfully produce **9** so column chromatography to isolate **8** is necessary.

Procedure The procedure was adopted from Wang *et al.*[25] To flame dried rbf was added SM **7** (750 mg, 3.7 mmol, 1 eq.), Cs_2CO_3 (3649 mg, 11.2 mmol 3 eq.), $PdCl_2dppf{\cdot}CH_2Cl_2$ (155 mg, 0.19 mmol, 5 mol%), and then freshly distilled THF (10 mL). Sealed and purged rxn with N_2. Triethylborane solution (1 M in THF, 11 mL, 11.2 mmol, 3 eq.) was added via syringe all at once while rxn was under N_2 atmosphere. The rxn was heated in a 70 °C oil bath for 6 hr. TLC indicated rxn completion and appearance of new, more polar spot that stained a different color with DNP than the SM (Fig. S13). TLCMS indicated this to be product at 148.9 m/z. To the rxn was added water and the rxn was acidified to pH 7 with 12 M HCl. The product was then extracted with 3 portions of Et_2O. Combined the organic layers, dried, concentrated, and dry loaded onto silica gel column and purified with 1:9 EtOAc-Hex and then 1:4 EtOAc-Hex. Obtained **8** as 506 mg cream solid (91% yield).

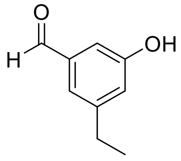

8 3-ethyl-5-hydroxybenzaldehyde (**8**)

^{1}H NMR (400 MHz, $CDCl_3$) δ 9.89 (s, 1H), 7.28 (s, 1H), 7.25-7.24 (t, *J* = 2.2 Hz, 1H), 7.03 (s, 1H), 6.96 (s, 1H), 2.69-2.63 (q, *J* = 7.6 Hz, 2H), 1.25-1.21 (t, *J* = 7.6 Hz, 3H). ^{13}C NMR (100 MHz, $CDCl_3$) δ 193.65, 156.80, 147.31, 137.69, 123.13, 122.14, 112.78, 28.57, 15.25.

APCI-MS $[M-1]^-$ = 148.9

HRESIMS *m/z* 151.0748, $[M+H]^+$ (calc'd for $C_9H_{11}O_2$, 151.0754)

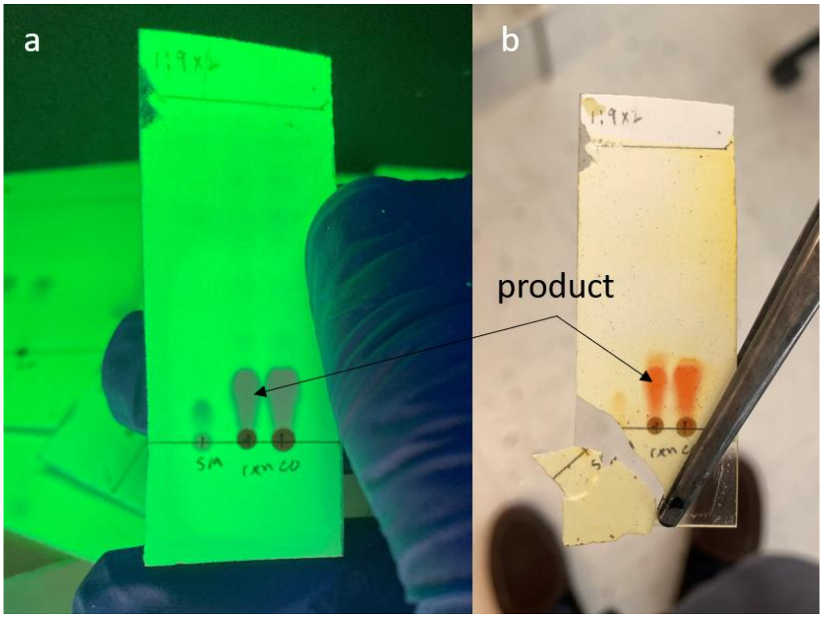

Figure S13. (a) TLC (UV 254 nm) Pd-catalyzed ethylation rxn in 1:9 EtOAc-Hex eluent (doubly developed). Going from left: 1st lane: SM 7, 2nd lane: rxn, 3rd lane: cospot. The product 8 is marked with an arrow. (b) TLC (DNP stain) Pd-catalyzed ethylation rxn in 1:9 EtOAc-Hex eluent (doubly developed). Going from left: 1st lane: SM 7, 2nd lane: rxn, 3rd lane: cospot. The product 8 is marked with an arrow.

Scheme S5 Step 7: Phenol Methylation

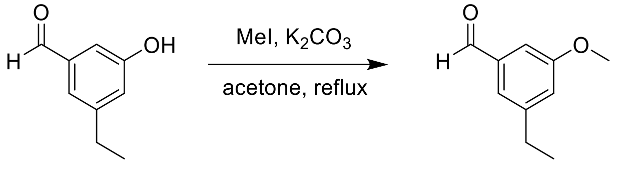

Comments Stirring **8** overnight at rt in the presence of 2 eq. MeI and 6 eq. K_2CO_3 does not result in full conversion to **9**. Addition of another 2 eq. MeI and 4 eq. K_2CO_3 and stirring at rt over the weekend resulted in decomposition. Therefore, the procedure was adjusted to reflux temperature for 12 h. If there is unreacted SM, it was chromatographically separated from product and subjected to the reaction conditions again. If the solvent evaporates completely to dryness during the course of reflux due to insufficient condensation, this results in much lower yields of **9** and it is recommended the rxn be restarted.

Procedure SM **8** (660 mg, 4.4 mmol, 1 eq.) was dissolved in acetone (15 mL) and transferred to a microwave vial. K_2CO_3 (6080 mg, 44 mmol, 10 eq.) in micropellet form was added followed by MeI (1.35 mL, 22 mmol, 5 eq.). The rxn was sealed and heated at 60 °C in the microwave for 12 h. TLC indicated some SM as a faint spot but also indicated significant amount of a new, less polar spot (Fig. S14). TLCMS confirmed this to be product at 163.8 m/z. Diluted rxn with EtOAc and water and stirred the layers. Separated the layers and washed aqueous layer with another portion of EtOAc. Combined EtOAc fractions, dried, concentrated, and dry loaded onto silica gel column and purified with 1:19 EtOAc-Hex to obtain **9** as 565 mg yellow oil (78% yield). 1:9 EtOAc-Hex can be run through the column to recover any unreacted SM.

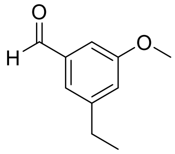

9 3-ethyl-5-methoxybenzaldehyde (**9**)

^{1}H NMR (400 MHz, $CDCl_3$) δ 9.91 (s, 1H), 7.27 (s, 1H), 7.19-7.18 (dd, *J* = 2.4 Hz, 1.3 Hz, 1H), 6.99-6.98 (t, *J* = 1.7 Hz, 1H), 3.82 (s, 3H), 2.69-2.64 (q, *J* = 7.6 Hz, 2H), 1.26-1.22 (t, *J* = 7.6 Hz, 3H). ^{13}C NMR (100 MHz, $CDCl_3$) δ 192.35, 160.26, 146.72, 137.88, 123.18, 121.12, 109.59, 55.44, 28.59, 15.26.

APCI-MS $[M]^+$ = 163.8

HRESIMS *m/z* 165.0895, $[M+H]^+$ (calc'd for $C_{10}H_{13}O_2$, 165.0910)

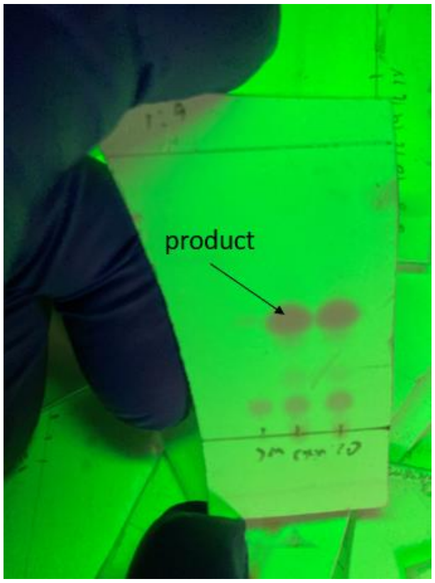

Figure S14. TLC (UV 254 nm) phenol methylation rxn in 1:9 EtOAc-Hex eluent. Going from left: 1st lane: SM 8, 2nd lane: rxn, 3rd lane: cospot. The product 9 is marked with an arrow.

Scheme S5 Step 8-10: Aldehyde *ortho*-Functionalization

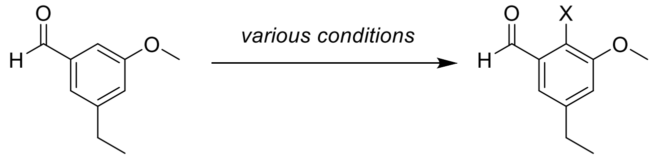

9 10

Table S7. Aldehyde ortho-functionalization rxn optimization.

Trial	Scale	X	Conditions	Observations	Yield	Ref.
1	50 mg	BMIDA	1. *N*,*N*,*N*-trimethylethylenediamine (1.1 eq.), *n*-BuLi (1.05 eq.) then *n*-BuLi (3 eq.), $B(OEt)_3$ (3.2 eq.), THF, -20 °C<br>2. MIDA (1.1 eq.), DMF, 85 °C	Rxn was stirred for 1 h after $B(OEt)_3$ addition	42% (over two steps)	[24,26]
2	293 mg	BMIDA	*Same as Trial 1*	Rxn was stirred overnight after $B(OEt)_3$ addition	decomp.	[24,26]
3	200 mg	BMIDA	*Same as Trial 1*	Scale-up of Trial 1	10% yield (over two steps)	[24,26]
4	100 mg	BMIDA	1. *N*,*N*,*N*-trimethylethylenediamine (1.6 eq.), *n*-BuLi (1.5 eq.) then *n*-BuLi (3.5 eq.), $B(OEt)_3$ (4.5 eq.), THF, -20 °C<br>2. MIDA (1.5 eq.), DMF, 85 °C	Increased equivalence of all reagents. MIDA boronate formation did not reach completion even after additional reagent and extended heating	0%	[24,26]
5	100 mg	$B(OH)_2$	*Same as Step 1 Trial 1*	Did not do Step 2 of Trial 1	46%	[24]
6	200 mg	$B(OH)_2$	*Same as Step 1 Trial 1*	Scale-up of Trial 5	16%	[24]
7	100 mg	BPin	*N*,*N*,*N*-trimethylethylenediamine (2.2 eq.), *n*-BuLi (2 eq.) then *n*-BuLi (4 eq.), (*i*PrO)BPin (4.5 eq.), THF, -20 °C	-	0%	[24]
8	35 mg	Br	NBS, DCM, 0 °C□rt	TLCMS confirmed product but isolation of this product revealed mixture of regioisomers by NMR	-	-
9	50 mg	BMIDA	*Same as Trial 1*	Attempted to repeat the success of Trial 1 in multiple 50 mg batches, since scale-up was unsuccessful	24% (over two steps)	[24,26]

10	50 mg	I	*N,N,N*-trimethylethylenediamine (1.1 eq.), *n*-BuLi (1.05 eq.) then *n*-BuLi (3 eq.), I_2 (3.2 eq.), THF, -70 °C□-20 °C	-	7%	24
11	50 mg	BMIDA	1. *N,N,N*-trimethylethylenediamine (1.1 eq.), *n*-BuLi (1.05 eq.) then *n*-BuLi (3 eq.), $B(OEt)_3$ (4.5 eq.), toluene, 0 °C<br>2. MIDA (1.1 eq.), DMF, 85 °C	Solvent was switched from THF to toluene. Crude boronic acid was detected by TLCMS but MIDA boronate formation was unsuccessful	0%	27
12	50 mg	I	*N,N,N*-trimethylethylenediamine (1.1 eq.), *n*-BuLi (1.05 eq.) then *n*-BuLi (3 eq.), I_2 (3.5 eq.), toluene, 0 °C	No product detected	-	27
13	50 mg	I	1. 1,3-propanediol (2 eq.), PPTS (5 mol%), toluene, reflux<br>2. *n*-BuLi (1.3 eq.), I_2 (1.5 eq.), cyclohexane:Et2O (1:1), THF<br>3. H_3O^+	Second step afforded no product	66%[a]	28
14	40 mg	BPin	1. $NaBH_4$ (5 eq.), MeOH, THF, 0 °C□rt<br>2. *n*-BuLi (2.5 eq.), (*i*PrO)BPin (1.2 eq.), Et_2O:THF (1:1), 0 °C to rt<br>3. [O]	N.R. for second step	95%[a]	29
15	50 mg	I	1. $NaBH_4$ (5 eq.), MeOH, THF, 0 °C□rt<br>2. *n*-BuLi (2.2 eq.), I_2 (1.5 eq.), Et_2O:cyclohexane (1:2), THF, 0 °C□rt<br>3. MnO_2 (10 eq.), $CHCl_3$, 65 °C	Protodehalogenation side reaction in Step 3 gave back SM **9** (12% yield)	14% (over 3 steps)	29
16	40 mg	I	1. $NaBH_4$ (5 eq.), MeOH, THF, 0 °C□rt<br>2. *n*-BuLi (2.2 eq.), I_2 (1.5 eq.), Et_2O:THF (1:1), THF, -70 °C□rt<br>3. MnO_2 (10 eq.), $CHCl_3$, 65 °C	Protodehalogenation side reaction in Step 3 gave back SM **9** (30% yield)	17% (over 3 steps)	29
17	43 mg	I	1. $NaBH_4$ (3 eq.), MeOH, THF, 0 °C□rt<br>2. *n*-BuLi (2.5 eq.), I_2 (2 eq.), Et_2O:THF (1:1), THF, -70 °C□rt<br>3. PCC (2 eq.), DCM, rt	Oxidizing agent was changed. No protodehalogenation product detected	34% (over 3 steps)	29
18	219 mg	I	*Same as Trail 17*	Scale-up of Trial 17	22% (over 3 steps)	29
19	265 mg	I	*Same as Trail 17*	Extended extraction time was done after Step 1 and distilled THF was used for Step 2	55% (over 3 steps)	29

[a] Yield of first step only.

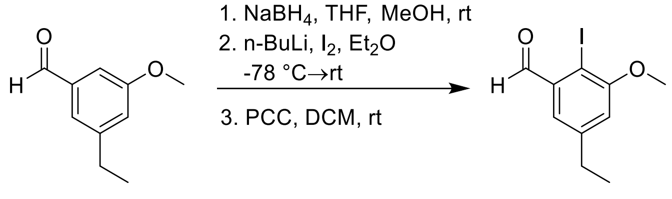

Comments THF was freshly distilled from Na benzophenone before use in Step 2 (distilled THF was not necessary for Step 1). For larger scales, lower than expected yields (~50%) were obtained for Step 1 despite TLC showing complete disappearance of SM and only one product spot. It was later discovered that extended stirring of EtOAc and H_2O layers during aqueous workup was necessary to maximize recovery of product, suggesting appreciable water solubility of benzyl alcohol intermediate. *n*-BuLi was titrated with menthol using bipyridine (bipy) as an indicator. For Step 3, MnO_2 forms product as well as protodehalogenated product, which gives back **9**. A DCM-Hex solvent system effectively separates **9** from **10** whereas an EtOAc-Hex does not. PCC led to much higher yields and negligible protodehalogenation side rxn.

Procedure The strategy and procedures were adopted from Jagot *et al.* with some modifications.[29] SM **9** (265 mg, 1.6 mmol, 1 eq.) was dissolved in THF (10 mL) and MeOH (2 mL). The soln. cooled to 0 °C and then $NaBH_4$ (182 mg, 4.8 mmol, 3 eq.) was added all at once. After letting the rxn warm up to rt overnight, TLC indicated rxn completion and new, more polar spot (Fig. S15a) [Note, the reduction rxn is complete within 1-2 hours and does not *need* to be stirred overnight]. TLCMS confirmed benzylalcohol intermediate at 166.0 m/z and 149.0 m/z $[M – OH]^+$. The rxn was diluted with water and extracted with EtOAc 4 times with extended stirring (10 min) for each extraction. Combined organic layers, dried, and concentrated down to 234 mg clear/yellow oil (88% crude yield). This crude benzylalcohol intermediate was dissolved in Et_2O (8 mL) and added via syringe through the septum of a flame dried microwave vial that was under an N_2 atmosphere. Cooled to -70 °C and added *n*-BuLi (1.5 M in hexanes, 2.34 mL, 3.5 mmol, 2.5 eq.) dropwise. The soln. gradually became orange. The rxn was allowed to slowly warm to rt over the course of 3 h, after which it became cloudy. Cooled to 0 °C, added distilled THF (2 mL) dropwise, and let stir at 0 °C for 15 min. I_2 (710 mg, 2.8 mmol, 2 eq.) dissolved in distilled THF (2 mL) was then added dropwise to reaction at 0 °C. The dark red I_2 color immediately disappeared upon contact with soln. but gradually persisted as addition neared completion. Once addition was complete, the rxn was stirred at 0 °C for 15 min. TLC indicated completion and appearance of new, less polar spot (Fig. S15b). TLCMS confirmed this iodoalcohol intermediate at 291.8 m/z and 274.8 m/z $[M – O]^+$. Saturated aq. sodium thiosulfate was added to the rxn and stirred. The layers were separated and washed aqueous layer was washed two more times with EtOAc. Combined organic layers, dried, and concentrated down to 399 mg orange solid (97% crude yield). The iodoalcohol intermediate was taken up in sieve dry DCM (30 mL) and transferred to a flame dried screw cap vial. PCC (586 mg, 2.72 mmol, 2 eq.) was added all at once, causing rxn to go from yellow to black. The rxn was stirred at rt for 2 h. TLC indicated rxn completion (Fig. S15c) and TLCMS confirmed product **10** at 290.0 m/z. The rxn was

concentrated and dry loaded onto silica gel column where the crude was purified with 1:4 DCM-Hex and then 2:5 DCM-Hex. Obtained **10** as 230 mg yellow crystalline solid (50% yield over 3 steps).

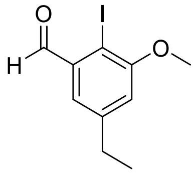

10 5-ethyl-2-iodo-3-methoxybenzaldehyde (**10**)

^{1}H NMR (400 MHz, $CDCl_3$) δ 10.09 (s, 1H), 7.28 (s, 1H), 6.85-6.85 (d, J = 1.5 Hz, 1H), 3.88 (s, 3H), 2.65-2.59 (q, J = 7.6 Hz, 2H), 1.23-1.19 (t, J = 7.6 Hz, 3H). ^{13}C NMR (100 MHz, $CDCl_3$) δ 196.34, 158.08, 146.13, 136.14, 121.45, 116.03, 90.34, 56.67, 28.39, 15.08.

APCI-MS $[M+1]^+$ = 290.0

HRESIMS *m/z* 290.9883, $[M+H]^+$ (calc'd for $C_{10}H_{12}IO_2$, 290.9882)

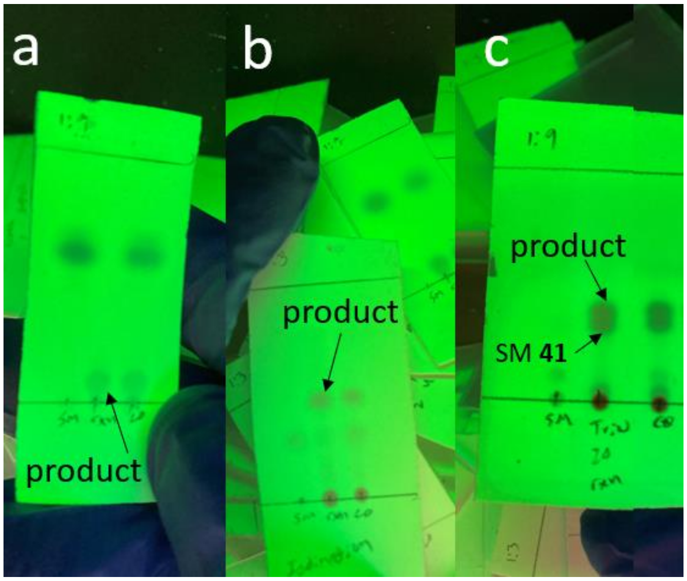

Figure S15. (a) TLC (UV 254 nm) reduction rxn in 1:9 EtOAc-Hex eluent. Going from left: 1st lane: SM 9, 2nd lane: rxn, 3rd lane: cospot. The benzylalcohol intermediate is marked with an arrow. (b) TLC (UV 254 nm) iodination rxn in 1:3 EtOAc-Hex eluent. Going from left: 1st lane: SM (benzylalcohol intermediate), 2nd lane: rxn, 3rd lane: cospot. The iodoalcohol intermediate is marked with an arrow. (c) TLC (UV 254 nm) oxidation rxn in 1:9 EtOAc-Hex eluent. Going from left: 1st lane: SM (iodoalcohol intermediate), 2nd lane: rxn, 3rd lane: cospot. The product (10) is marked with an arrow as well as protodehalogenated byproduct (9).

Scheme S5 Step 11: Suzuki Cross-Coupling

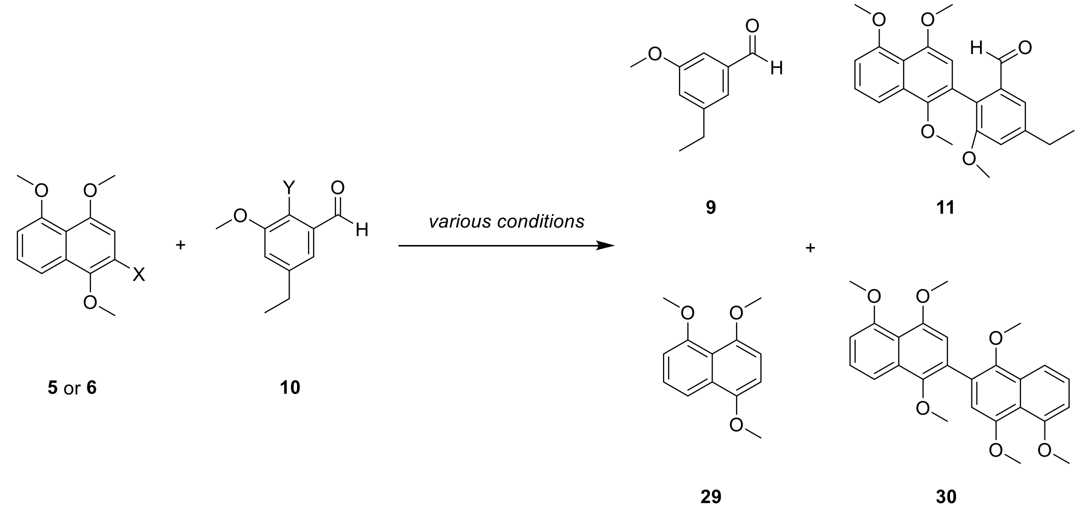

Table S8. Suzuki cross-coupling rxn optimization.

Trial	Scale	X	Y	Conditions	Observations	Yield	Ref.
1	31 mg	Br (1 eq.)	BMIDA (1.2 eq.)	$Pd(OAc)_2$ (5 mol%), SPhos (10 mol%), K_3PO_4 (7 eq.), Dioxane/H_2O (5:1)	-	37% of **11**	17
2	53 mg	Br (1 eq.)	$B(OH)_2$ (1.5 eq.)	$Pd(OAc)_2$ (2 mol%), SPhos (4 mol%), K_3PO_4 (2 eq.), toluene	-	51% of **11**<br>9% of **9**<br>32% of **29**	16
3	39 mg	Br (1 eq.)	$B(OH)_2$ (1.5 eq.)	*Same as Trial 2 except $Pd(OAc)_2$ and SPhos catalyst loadings were increased to 5 mol% and 10 mol%, respectively*	Repeat of Trial 2. Saw product by TLC and TLCMS but got 0% yield upon isolation, showing difficulty in reproducing rxn	0%	16
4	21 mg	BPin (1.5 eq.)	I[a] (1.0 eq)	$PdCl_2dppf{\cdot}CH_2Cl_2$ (10 mol%), NEt_3 (5 eq.), THF/H_2O (4:1)	Observed only **30** by TLCMS	-	30
5	31 mg	Br (1 eq.)	BMIDA (1.5 eq.)	*Same as Trial 1*	Increased BMIDA eq.	61% of **11**	17
6	48 mg	BPin (1.5 eq.)	I (1 eq.)	*Same as Trial 1*	-	58% of **11**	17
7	317 mg	BPin (1.5 eq.)	I (1 eq.)	*Same as Trial 1*	Scale up of Trial 6. Two purifications done as the first one did not	50% of **11**<br>12% of **30**	17

					efficiently separate **11** from **30**.		
8	272 mg	BPin (1.5 eq.)	I (1 eq.)	*Same as Trial 1*	Only one purification with EtOAc-DCM system was done which separated **11** from **30**.	81% of **11**<br>n.d. of **30**	17

[a] The iodo coupling partner was the corresponding benzyl alcohol of aldehyde **10**.

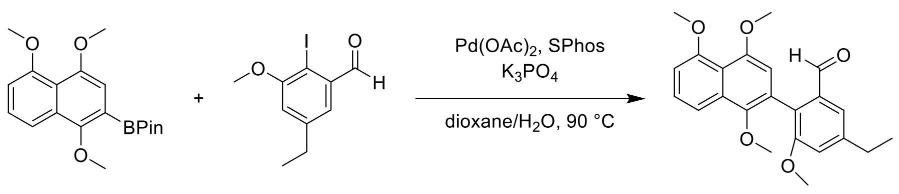

6 10 11

Comments Dioxane was freshly distilled from Na benzophenone prior to use in the rxn. DI water was degassed. At larger scale couplings (>300 mg), product **30** was observed.

Procedure To microwave vial was added $Pd(OAc)_2$ (12 mg, 0.055 mmol, 5 mol%) and SPhos (45 mg, 0.109 mmol, 10 mol%). SM **10** (272 mg, 0.93 mmol, 1 eq.) and SM **6** (484 mg, 1.4 mmol, 1.5 eq.) were both dissolved in distilled dioxane (10 mL) and transferred to the microwave vial containing the catalysts. The vial was sealed and purged with N_2. K_3PO_4 (1381 mg, 6.5 mmol, 7 eq.) was added to degassed water (2 mL) to prepare a ~3 M soln. This soln. was added via syringe to the microwave vial, giving a 5:1 ratio between dioxane and water that formed two layers. The rxn was heated at 80 °C in microwave for 15 h. TLC showed faint presence of some SM but indicated several new UV active spots (Fig. S16). TLCMS confirmed the large, more polar UV active spot was desired coupled product **11** at 380.9 m/z. TLCMS also indicated the blue fluorescent UV active spot was homocoupled side product **30** at 435.3 m/z. The rxn was diluted with water and the layers were separated. The aqueous layer was then washed twice with EtOAc. The organic layers were combined, dried, and the crude was dry loaded onto a silica gel column and purified with 2% EtOAc-DCM to obtain **11** as 286 mg orange-yellow solid (81% yield).

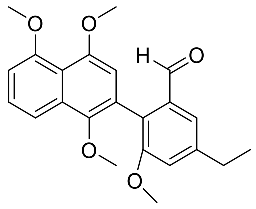

11 5-ethyl-3-methoxy-2-(1,4,5-trimethoxynaphthalen-2-yl)benzaldehyde (**11**)

^{1}H NMR (400 MHz, $CDCl_3$) δ 9.71 (s, 1H), 7.77-7.74 (dd, *J* = 8.4 Hz, 0.9 Hz, 1H) 7.53-7.53 (d, *J* = 1.5 Hz, 1H), 7.46-7.42 (t, *J* = 8.0 Hz, 1H), 7.10-7.10 (d, *J* = 1.4 Hz, 1H), 6.95-6.93 (dd, *J* = 7.7 Hz, 0.5 Hz, 1H), 6.70 (s, 1H), 4.00 (s, 3H), 3.92 (s, 3H), 3.81 (s, 3H), 3.45 (s, 3H), 2.82-2.76

(q, J = 7.6 Hz, 2H), 1.36-1.33 (t, J = 7.6 Hz, 3H) . ^{13}C NMR (100 MHz, $CDCl_3$) δ 192.87, 157.45, 157.20, 152.77, 147.80, 145.67, 134.84, 131.26, 128.39, 136.91, 122.25, 118.50, 118.04, 116.10, 115.23, 109.67, 107.26, 60.83, 56.89, 56.63, 56.06, 29.06, 15.33.

APCI-MS $[M+1]^+$ = 380.9

HRESIMS m/z 381.1686, $[M+H]^+$ (calc'd for $C_{23}H_{25}O_5$, 381.1702)

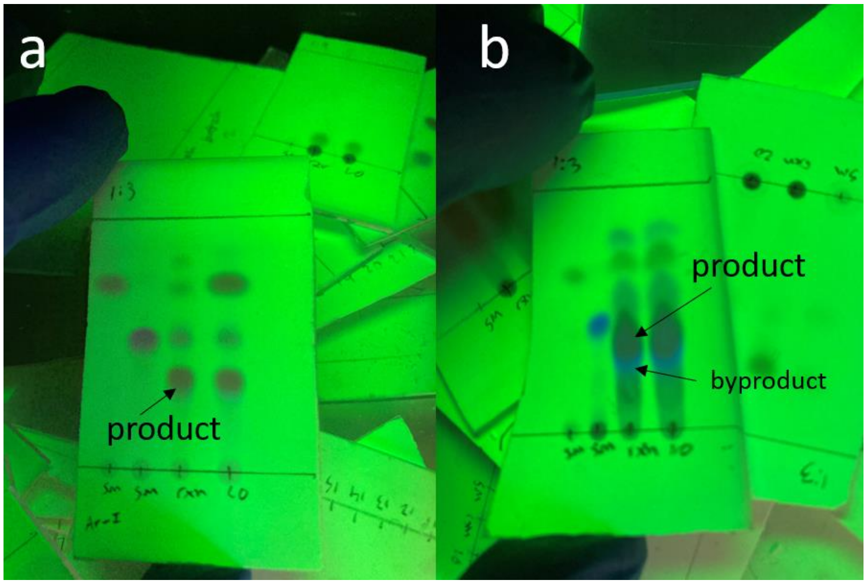

Figure S16. (a) TLC (UV 254 nm) Suzuki coupling rxn in 1:3 EtOAc-Hex eluent. Going from left: 1st lane: SM 10, 2nd lane: SM 6, 3rd lane: rxn, 4th lane: cospot. The product 11 is marked with an arrow. (b) TLC (UV 254 nm) Suzuki coupling rxn scale-up in 1:3 EtOAc-Hex eluent. All lanes are the same as (a). The product 11 and homocoupled byproduct 30 are marked with an arrow.

Scheme S5 Step 12 & 13: Oxidation-Intramolecular Friedel-Crafts (IMFC) Acylation Sequence

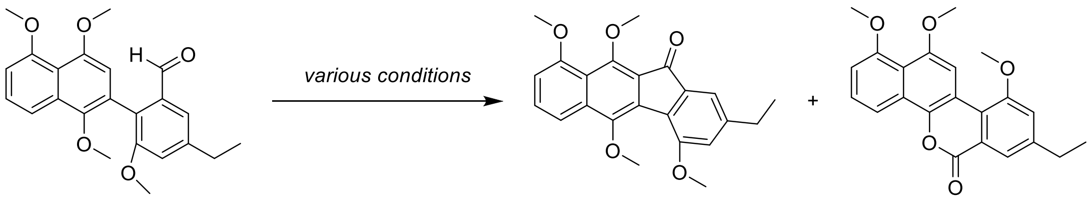

11 12 31

Table S9. IMFC acylation rxn optimization.

Trial	Scale	Conditions	Observations	Yield	Ref.
1	14 mg	1. NaOH (50 eq.), H_2O_2 (75 eq.), MeOH, 110 °C, µW<br>2. TFAA (5 eq.), molecular sieves, $MeNO_2$, 90 °C	Decomp.	-	[19]
2	10 mg	$NH_4S_2O_8$ (2 eq.), MeCN/H_2O (1:1)	TLC indicated rxn completion and TLCMS showed spot at 350 m/z which appears to be some sort of decarbonylative product	-	[31]
3	10 mg	1. *same as Step 1 Trial 1*<br>2. $(COCl)_2$ (6 eq.), MeCN, 80 °C, then $AlCl_3$ (10 eq.), DCM, rt	Rxn was stirred with $AlCl_3$ overnight. Observed decomp. By TLC.		[24]
4	21 mg	*Same as Trial 3*	Rxn was stirred with $AlCl_3$ for 1 h. Unlike Trial 3, decomp. was not observed but **12** was not seen by TLCMS. Instead, a mass of 350 m/z was observed, suggesting premature demethylation	-	[24]
5	10 mg	1. *same as Step 1 Trial 1*<br>2. $(COCl)_2$ (12 eq.), MeCN, 80 °C	Step 2 stirred for 1.5 h. **12** and **31** observed by TLCMS.	11% (over two steps) of **12**	[24]
6	9 mg	*Same as Trial 5*	Step 2 stirred for overnight. Only **31** observed by TLCMS.	-	[24]
7	34 mg	1. *same as Step 1 Trial 1*<br>2. Cyanuric chloride (3.2 eq.), pyridine (2 eq.), then $TiCl_4$ (2 eq.), DCM, rt	-	50% (over two steps) of **12**, 6% (over two steps) of **31**	[20,24,32]
8	205 mg	*Same as Trial 7*	Scale-up of Trial 7. Added an additional 2 eq. $TiCl_4$.	46% (over two steps) of **12**, 5%	[20,24,32]

				(over two steps) of **31**	
9	286 mg	1. *same as Step 1 Trial 1*<br>2. Cyanuric chloride (0.8 eq.), pyridine (0.5 eq.), then $TiCl_4$ (1.5 eq.), DCM, rt	Reduced equivalents of reagents from Trial 7.	42% (over two steps) of **12**, yield of **31** n.d.	[20,24,32]

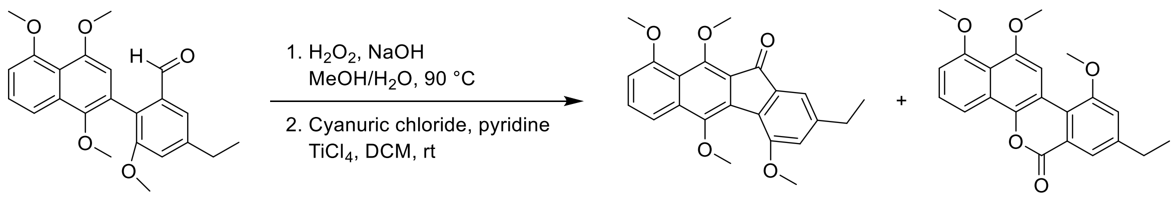

11 **12** **31**

Comments H_2O_2 oxidation of **11** generates extremely high pressures, which is exacerbated at higher scales. Biotage microwave vials with Biotage aluminum caps can withstand pressures of 15-20 bar before the vial shatters or the cap pops off and causes the rxn contents to be lost. For Step 2, additional $TiCl_4$ Lewis acid can be added if carboxylic acid SM is still observed by TLC.

Procedure SM **11** (286 mg, 0.75 mmol, 1 eq.) was dissolved/suspended in MeOH (12 mL) and transferred to microwave vial and to it was added aqueous NaOH (30% m/v, 5.0 mL, 37.5 mmol, 50 eq.) followed by aqueous H_2O_2 (30% w/w, 3.1 mL, 28 mmol, 37.5 eq.). The vial was sealed, placed in Biotage microwave, and heated to 110 °C for 30 min. **CAUTION! PRESSURE EXPONENTIALLY INCREASES IN THE FIRST COUPLE MINUTES OF THE RXN! FOR SAFETY, WHEN THE PRESSURE OF THE RXN REACHED 10 BAR, A BLEED NEEDLE WAS INSERTED INTO THE RXN AND WAS CONTINUSOULY DONE SO UNTIL THE PRESSURE FLATLINED AT ~9-10 BAR.** After 30 min. another 50 eq. NaOH and 37.5 eq. of H_2O_2 were added via syringe and the rxn was heated at 110 °C for another 30 min. TLC indicated rxn completion and appearance of more polar spot (Fig. S17). TLCMS confirmed carboxylic acid intermediate at 394.9 m/z. The rxn was diluted with water, causing a white precipitate to form, and acidified to pH 3-4 using 12 M HCl. $CHCl_3$ was added, forming two layers, and the biphasic rxn was stirred for several minutes (cloudiness disappeared). The layers were separated and aqueous layer was washed two more times with $CHCl_3$. Combined the organic layers, dried, and concentrated down to 339 mg yellow-brownish solid (>100% crude yield), which was subjected to the next rxn without further purification. The crude carboxylic acid (339 mg, 0.75 mmol, 1 eq.) obtained in previous step was dissolved in sieve dry DCM (20 mL) and transferred to flame-dried screw cap vial. Cyanuric chloride (115 mg, 0.62 mmol, 0.8 eq.) was added followed by pyridine (31 µL, 0.39 mmol, 0.5 eq.). A precipitate formed. TLC appeared to indicate no rxn progress (Fig. S18a). $TiCl_4$ (128 µL, 1.17 mmol, 1.5 eq.) was added and the rxn turned purple. After 5 min. stirring at rt, TLC indicated consumption of SM and appearance of two less polar spots that were fluorescent yellow and fluorescent blue (Fig. S18b). TLCMS indicated the top yellow fluorescent spot was **12** at 379.1

m/z and the bottom blue fluorescent spot was **31** at 365.0 m/z. The rxn was quenched with water and stirred. The layers were separated and the organic layer was washed one more time with water. The organic layer was dried, concentrated, and dry loaded onto a silica gel column and purified with 1:9 EtOAc-Hex and then 1:4 EtOAc-Hex. Obtained a total of 121 mg of **12** as yellow/orange solid (42% yield over two steps).

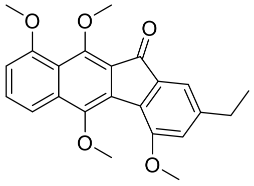

12 2-ethyl-4,5,9,10-tetramethoxy-11H-benzo[b]fluoren-11-one (**12**)

^{1}H NMR (400 MHz, $CDCl_3$) δ 7.78-7.76 (d, *J* = 8.4 Hz, 1H), 7.49-7.45 (t, *J* = 8.0 Hz, 1H), 7.28 (s, 1H), 6.95 (s, 1H), 6.89-6.87 (d, *J* = 7.8 Hz, 1H), 4.04 (s, 3H), 4.03 (s, 3H), 3.98 (s, 3H), 3.89 (s, 3H), 2.72-2.66 (q, *J* = 7.6 Hz, 2H), 1.30-1.26 (t, *J* = 7.6 Hz, 3H). ^{13}C NMR (100 MHz, $CDCl_3$) δ 190.52, 159.46, 155.31, 155.26, 147.82, 146.56, 138.98, 137.35, 129.99, 127.85, 127.08, 122.93, 121.58, 118.06, 116.18, 115.91, 108.26, 63.21, 62.50, 56.41, 56.25, 28.87, 15.21.

APCI-MS $[M+1]^+$ = 379.1

HRESIMS *m/z* 379.1550, $[M+H]^+$ (calc'd for $C_{23}H_{23}O_5$, 379.1545)

Fluorescence (*c* = 0.1 mg/mL, MeOH) $\lambda_{max,ex}$ 310 nm, $\lambda_{max,em}$ 386 nm, Int. 184

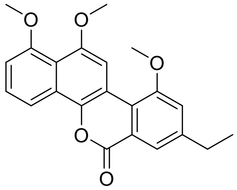

31 5-ethyl-3,15,17-trimethoxy-9-oxatetracyclo[8.8.0.0^{2,7}.0^{11,16}]octadeca-1(10),2(7),3,5,11,13,15,17-octaen-8-one (**31**)

^{1}H NMR (400 MHz, $CDCl_3$) δ 8.42 (s, 1H), 8.23-8.20 (dd, *J* = 8.5 Hz, 0.8 Hz, 1H), 7.98 (s, 1H), 7.53-7.49 (t, *J* = 8.2 Hz, 1H), 7.18 (s, 1H), 6.99-6.98 (d, *J* = 7.8 Hz, 1H), 4.08 (s, 3H), 4.03 (s, 3H), 4.01 (s, 3H), 2.82-2.76 (q, *J* = 7.6 Hz, 2H), 1.35-1.32 (t, *J* = 7.6 Hz, 3H). ^{13}C NMR (100 MHz, $CDCl_3$) δ 161.71, 157.45, 156.79, 152.93, 146.17, 140.68, 127.30, 126.82, 123.42, 122.33, 121.69, 117.62, 117.22, 114.99, 113.89, 108.08, 104.51, 56.87, 56.65, 56.38, 29.08, 15.26.

APCI-MS $[M+1]^+$ = 365.1.

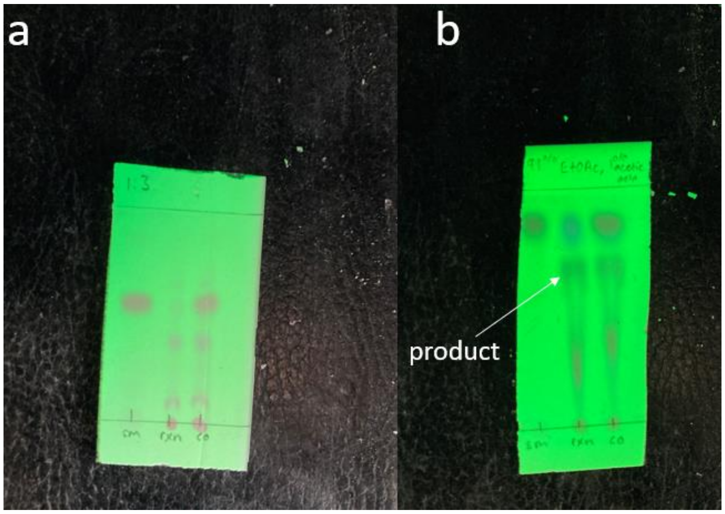

Figure S17. (a) TLC (UV 254 nm) oxidation rxn in 1:3 EtOAc-Hex eluent. Going from left: 1st lane: SM 11, 2nd lane: rxn, 3rd lane: cospot. (b) TLC (UV 254 nm) oxidation rxn in 99:1 EtOAc-AcOH. Going from left: 1st lane: SM 11, 2nd lane: rxn, 3rd lane: cospot. The carboxylic acid intermediate is marked with an arrow.

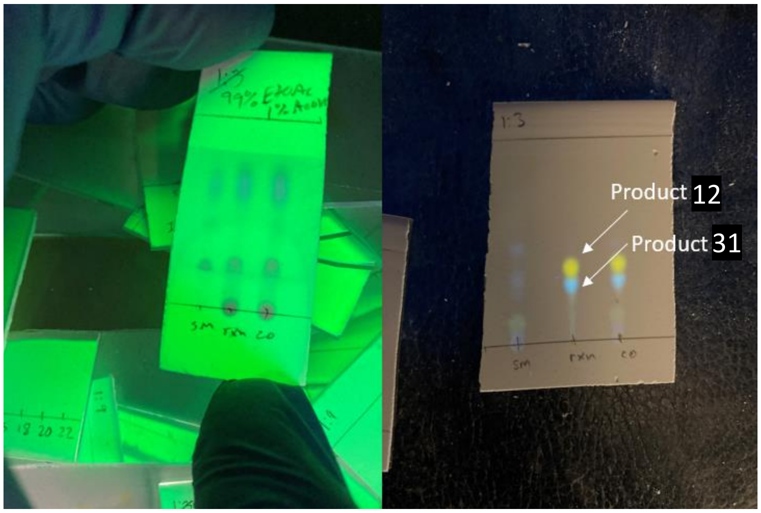

Figure S18. (a) TLC (UV 254 nm) IMFC rxn before Lewis acid addition in 99:1 EtOAc-AcOH eluent. Going from left: 1st lane: SM (carboxylic acid intermediate), 2nd lane: rxn, 3rd lane: cospot. Rxn did not appear to indicate any new spots. (b) TLC (UV 365 nm) IMFC rxn after Lewis acid addition in 1:3 EtOAc-Hex. Going from left: 1st lane: SM (carboxylic acid intermediate), 2nd lane: rxn, 3rd lane: cospot. The products 12 and 31 are marked with arrows.

Scheme S5 Steps 14-16: Final Sequence: Demethylation, Hydrazine Condensation, Oxidation

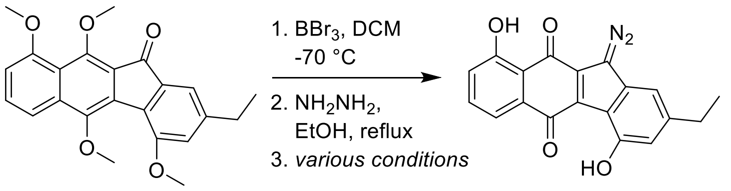

12 **2**

Table S10. Final sequence rxn optimization.

Trial	Scale	Conditions	Observations	Yield	Ref.
1	17 mg	1. BBr_3 (10 eq.), DCM, -70 °C to -20 °C<br>2. Hydrazine (70 eq.), EtOH, 90 °C, μW<br>3. Fetizon's reagent (50 wt%, 26 eq.), NEt_3 (20 eq.), DCM, rt	Purified **2** on silica gel.	14% (over 3 steps)	[13]
2	95 mg	1. *Same as Trial 1*<br>2. *Same as Trial 1*<br>3. Fetizon's reagent (50 wt%, 13 eq.), NEt_3 (20 eq.), DCM:Acetone (1:1), rt	Acetone cosolvent added in step 3 to increase solubility of SM. Purified **2** on silica gel.	9% (over 3 steps)	[13]
3	63 mg	1. *Same as Trial 1*<br>2. *Same as Trial 1*<br>3. Fetizon's reagent (50 wt%, 26 eq.), NEt_3 (20 eq.), Acetone, rt	Step 3 was run with acetone solvent and rxn was allowed to run for extended time. Purified **2** on silica gel.	2% (over 3 steps)	[13]
4	32 mg	1. *Same as Trial 1*<br>2. *Same as Trial 1*<br>3. Oxalyl chloride (1.05 eq.), NEt_3 (2.1 eq.), DMSO (1.1 eq.), THF, -70 °C	Attempted Swern oxidation for Step 3 but result was NR.	-	[13,33]
5	32 mg	1. *Same as Trial 1*<br>2. *Same as Trial 1*<br>3. Air balloon, EtOH, rt	Attempted autooxidation for Step 3 but result was NR.	-	[13]
6	32 mg	1. *Same as Trial 1*<br>2. *Same as Trial 1*<br>3. MnO_2 (10 eq.), $MgSO_4$ (3 eq.), DCM, 0 °C to rt	Filtered MnO_2 oxidation rxn through celite and obtained high crude yield (>100%) but had very low yield after purification of **2** on silica gel.	5% (over 3 steps)	[13]

7	30 mg	1. *Same as Trial 1*<br>2. *Same as Trial 1*<br>3. *Same as Trial 6*	Purified **2** on neutral alumina.	52% (over 3 steps)	13

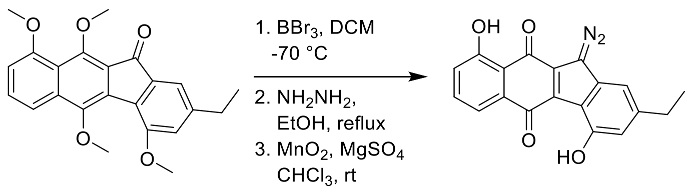

12 **2**

Comments Hydrazine monohydrate was made anhydrous by refluxing over an equal weight of NaOH (i.e. 5 g of hydrazine monohydrate requires 5 g of NaOH) for 1.5 h in 130 °C oil bath and then distilled under nitrogen. MnO_2 (5 µm, activated 85%) was used straight from the commercial bottle without any drying.

Procedure To a flame dried vial screwcap vial was added SM **12** (30 mg, 0.079 mmol, 1 eq.) that was dissolved in sieve-dried DCM (5 mL). Cooled to -70 °C in dry ice MeOH bath and added BBr_3 (5 M in DCM, 158 µL, 0.79 mmol, 10 eq.) dropwise and soln. immediately turned dark purple. Once addition was complete, the rxn was transferred to -20 °C fridge to stir overnight. TLC confirmed rxn completion and appearance of a more polar orange fluorescent spot (Fig. S19). TLCMS confirmed hydroquinone intermediate at 321.1 m/z and 363.1 $[M+MeCN]^+$. The rxn was quenched with water and then EtOAc and the layers were stirred. The layers were separated and the organic layer was washed with brine. The layers were separated, the organic layer was dried, and concentrated down to 66 mg dark red solid that was used in the next step without further purification. Quantitative conversion was assumed. To flame dried microwave vial was added hydroquinone intermediate obtained from last step (0.079 mmol, 1 eq.) that was dissolved in anhydrous ethanol (4 mL). To the red soln. was added hydrazine (277 µL, 5.53 mmol, 70 eq.) and the rxn was sealed and heated to 90 °C in microwave for 30 min. The soln. was now purple. TLC indicated rxn completion and a less polar spot that had lost the fluorescence of the SM and had red visible color (Fig. S20). TLCMS confirmed hydrazone intermediate at 335.1 m/z. Concentrated rxn down to purple solid that was used in next rxn without further purification and assumed quantitative conversion. To vial containing hydrazone intermediate obtained from last step (0.079 mmol, 1 eq.) was added sieve dried DCM (15 mL). Soln. was dark purple. Added $MgSO_4$ (28 mg, 0.23 mmol, 3 eq.) Cooled to 0 °C and then added MnO_2 (85% activated, 69 mg, 0.79 mmol, 10 eq.). As soon as addition was complete, brought out of ice bath to stir at rt overnight covered in aluminum foil. TLC indicated rxn completion and appearance of less polar spot that had brown color (Fig. S21). TLCMS indicated product at 346.1 m/z $[M+1 – N_2 + MeCN]^+$. Filtered rxn through celite and eluted with EtOAc. Concentrated brown filtrate and dry loaded onto neutral alumina and purified with a gradient of 100% hexanes, 1:9 EtOAc-Hex, 15% EtOAc-Hex, 1:3 EtOAc-Hex, 1:1 EtOAc-Hex, and then finally 3:1 EtOAc-Hex to yield 13.7 mg of prelomaiviticin (**2**) as a brown solid (52% yield over 3 steps).

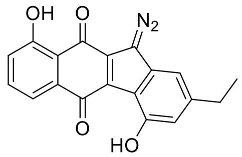

2 Prelomaiviticin

2-ethyl-4,9-dihydroxy-11-(-λ^5-diazynylidene)-5H,10H,11H-benzo[b]fluorene-5,10-dione (**2**)

^{1}H NMR (400 MHz, $CDCl_3$) δ 12.10 (s, 1H), 10.99 (s, 1H), 7.77-7.75 (dd, *J* = 7.5 Hz, 1.1 Hz, 1H), 7.61-7.57 (t, *J* = 8.3 Hz, 1H), 7.25-7.22 (dd, *J* = 8.5 Hz, 1.1 Hz, 1H), 6.82 (s, 1H), 6.71 (s, 1H), 2.73-2.67 (q, *J* = 7.4 Hz, 2H), 1.30-1.26 (t, *J* = 7.6 Hz, 3H). ^{13}C NMR (100 MHz, DMSO-d6) δ 185.88, 178.60, 161.45, 152.18, 143.23, 137.64, 136.50, 134.82, 131.70, 128.64, 123.46, 121.29, 118.97, 115.95, 108.75, 108.43, 67.39, 28.94, 15.66

FT-IR (neat, ATR) 2920, 2850, 2100, 1600 cm^{-1}.

APCI-MS $[M\text{-}N_2\text{+}MeCN\text{+}1]^+$ = 346.1

HRESIMS *m/z* 333.0862, $[M+H]^+$ (calc'd for $C_{19}H_{13}N_2O_4$, 333.0875)

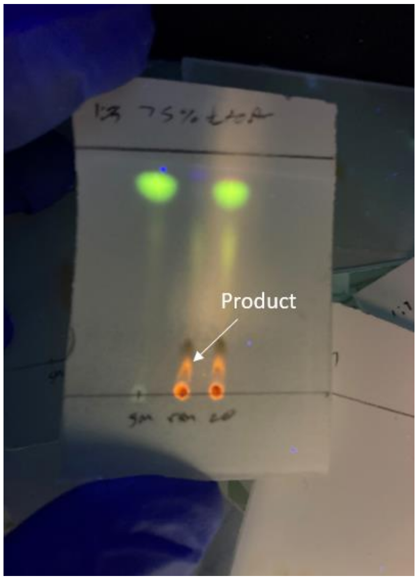

Figure S19. TLC (UV 365 nm) BBr_3 demethylation rxn in 3:1 EtOAc-Hex. Going from left: 1st lane: SM 12, 2nd lane: rxn, 3rd lane: cospot. The hydroquinone intermediate is marked with an arrow.

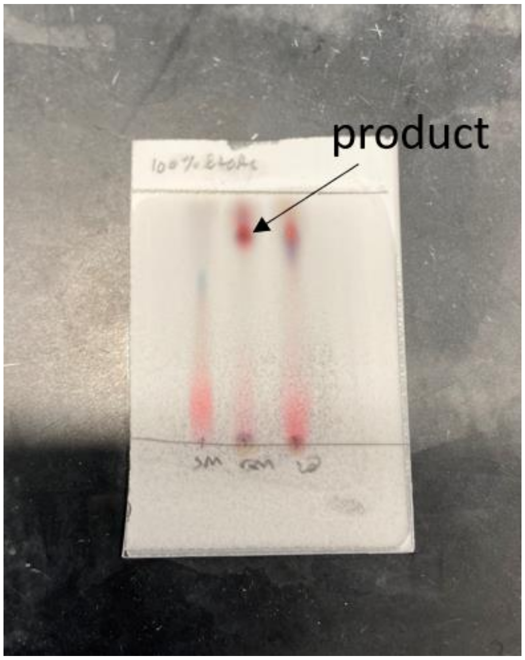

Figure S20. TLC (no visualization) hydrazine condensation rxn in 100% EtOAc. Going from left: 1st lane: SM (hydroquinone intermediate), 2nd lane: rxn, 3rd lane: cospot. The hydrazone intermediate is marked with an arrow.

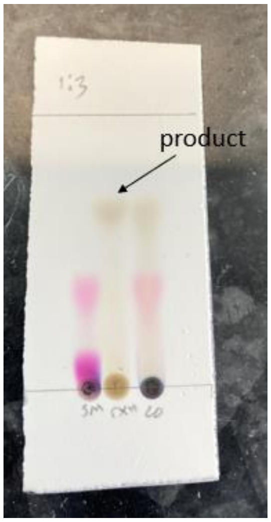

Figure S21. TLC (no visualization) oxidation rxn in 1:3 EtOAc-Hex. Going from left: 1st lane: SM (hydrazone intermediate), 2nd lane: rxn, 3rd lane: cospot. The product 2 is marked with an arrow.

Scheme S5 Step 17: Diazo Substitution

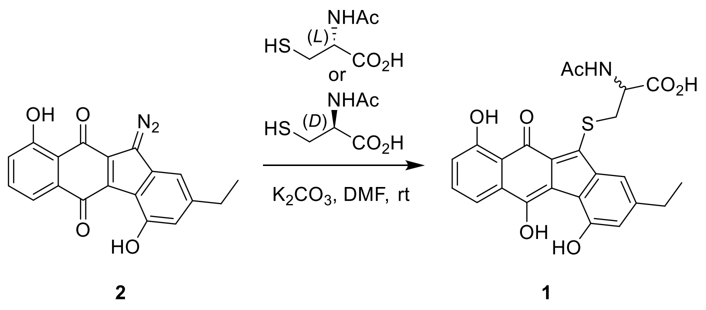

Comments Peptide-grade DMF was used. The rxn is covered by aluminum foil when stirring due to potential light-sensitivity. A Waters XBridge BEH C18 OBD Prep Column (130 Å, 5 µm, 10 mm x 250 mm) was used for reverse phase purification and the product was monitored at 220 and 280 nm.

Procedure The procedure was adopted from Herzon and corworkers.[34] To a vial that contained prelomaiviticin (**2**) (13.7 mg, 0.041 mmol, 1 eq.) was added peptide grade DMF (4 mL), which formed brown soln. To this was added *N*-acetyl-L-cysteine (60 mg, 0.37 mmol, 9 eq.) and K_2CO_3 (33 mg, 0.24 mmol, 5.8 eq.) and the rxn was stirred at rt for 2 h covered in aluminum foil. The soln. slowly became dark green/blue. TLC was not done on the rxn but LCMS on crude rxn mixture revealed presence of homoseongomycin (**1**) in negative mode at 466.1 m/z $[M – 1]^-$ and 305.1 m/z $[M – 1 – \text{cysteine}]^-$. The rxn was concentrated using a Biotage V10 touch to remove DMF and purified using reverse phase preparatory liquid chromatography (Table S11). The fractions were analyzed by LCMS and those containing **1** were first concentrated in Biotage V10 Touch to remove MeCN and then lyophilized overnight to remove water and yield 4.3 mg of *L*-homoseongomycin as a dark purple powder (22% yield). For the preparation of *D*-homoseongomycin, the exact same procedure was used starting with 1.4 mg of **2** and *N*-acetyl-*D*-cysteine to yield 0.7 mg of product (35% yield).

Table S11. Reverse phase purification method for homoseongomycin purification.

Time (min.)	Flow (mL/min)	%A	%B
Initial	2.80	60.0	40.0
5.40	2.80	60.0	40.0
27.63	2.80	32.0	68.00
30.00	2.80	0.0	100.0
34.00	2.80	0.0	100.0
39.00	2.80	0.0	100.0

40.00	2.80	60.0	40.0
43.00	2.80	60.0	40.0

A = Ultrapure Water + 0.1% Trifluoroacetic acid B = LCMS-grade Acetonitrile+ 0.1% Trifluoroacetic acid

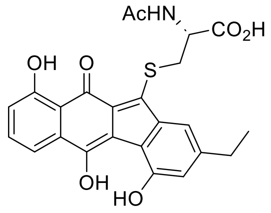

***L*-1** *L*-Homoseongomycin

(2*R*)-2-acetamido-3-({2-ethyl-4,5,9-trihydroxy-10-oxo-10H-benzo[b]fluoren-11-yl}sulfanyl)propanoic acid (*L*-**1**)

^{1}H NMR (500 MHz, DMSO-d6 + TFA) δ 13.49 (broad s, 1H), 8.30-8.28 (d, *J* = 8.0 Hz, 1H), 7.56-7.53 (t, *J* = 8.0 Hz, 1H), 7.37-7.35 (d, *J* = 8.0 Hz, 1H), 7.15 (s, 1H), 6.98-6.96 (d, *J* = 8.0 Hz, 1H), 6.73 (s, 1H), 4.44-4.39 (m, 1H), 3.88-3.84 (dd, *J* = 12.0 Hz, 4 Hz, 1H), 3.53-3.49 (dd, *J* = 8.0 Hz, 8.0 Hz, 1H), 2.63-2.58 (q, *J* = 8.0 Hz, 2 H), 1.72 (s, 3H), 1.21-1.18 (t, *J* = 8.0 Hz, 3H). ^{13}C NMR (100 MHz, DMSO-d6 + TFA) δ 184.0, 171.8, 169.4, 162.9, 149.3, 149.0, 145.1, 144.9, 141.7, 136.0, 133.9, 128.4, 120.4, 119.7, 116.1, 115.9, 115.7, 114.3, 52.4, 34.8, 28.4, 22.2, 15.6.

UV (MeOH, 0.1 mg/mL) λ_{max} 285, 514 nm

FT-IR (neat, ATR) 3700-2300, 2980, 2920, 1680, 1620, 1595, 1450, 1200 cm^{-1}.

ESI-MS $[M-1]^-$ 466.1 (31%), 305.1 (100%)

HRMS calcd. for $[C_{24}H_{21}NO_7S-1]^-$ 466.0960, found 466.0988

Experimental specific rotation:

$[\alpha]_D^{25}$ = +200°-+250° (α = +0.004 – +0.005, *c* = 0.002, MeOH) *higher concentrations (c >0.01) lead to "dark sample" error on Rudolph Autopol IV polarimeter.*

Calculated specific rotation (*c* = 0.07, MeOH) (*Jasco KK Transform from CD to ORD*):

$[\alpha]_D^{25}$ = + 2.18°, $[\alpha]_{546}^{25}$ = + 31.1°, $[\alpha]_{436}^{25}$ = + 92.8°, $[\alpha]_{406}^{25}$ = + 270°, $[\alpha]_{364}^{25}$ = + 158.6°

Calculated specific rotation (*c* = 0.07, MeOH) (*manual KK Transform from CD to ORD*):

$[\alpha]_D^{25}$ = – 37.2°, $[\alpha]_{546}^{25}$ = + 13.1°, $[\alpha]_{436}^{25}$ = + 137.1°, $[\alpha]_{406}^{25}$ = + 505.2°, $[\alpha]_{364}^{25}$ = + 239.2°

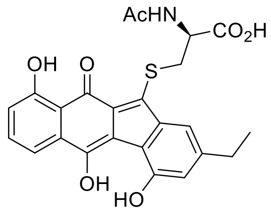

D-1 *D*-Homoseongomycin

(2*S*)-2-acetamido-3-({2-ethyl-4,5,9-trihydroxy-10-oxo-10H-benzo[b]fluoren-11-yl}sulfanyl)propanoic acid (*D*-**1**)

ESI-MS $[M-1]^-$ 466.1 (31%), 305.1 (100%)

Experimental specific rotation: $[\alpha]^{25}_{D}$ = 0° (α = 0.000, c = 0.002, MeOH) *higher concentrations (c>0.01) lead to "dark sample" error on Rudolph Autopol IV polarimeter.*

Calculated specific rotation (c = 0.07, MeOH) (*Jasco KK Transform from CD to ORD*):

$[\alpha]^{25}_{D}$ = – 36.4°, $[\alpha]^{25}_{546}$ = – 75.1°, $[\alpha]^{25}_{436}$ = – 56.6°, $[\alpha]^{25}_{406}$ = – 201.4°, $[\alpha]^{25}_{364}$ = – 231.1°

Calculated specific rotation (c = 0.07, MeOH) (*manual KK Transform from CD to ORD*):

$[\alpha]^{25}_{D}$ = – 24.6°, $[\alpha]^{25}_{546}$ = – 94.1°, $[\alpha]^{25}_{436}$ = – 51.1°, $[\alpha]^{25}_{406}$ = – 353.7°, $[\alpha]^{25}_{364}$ = – 385.4°

Synthetic Homoseongomycin NMR Data Comparison

NMR data of our synthetic sample of *L*-homoseongomycin (*L*-**1**) was compared with various literature NMR data for the compound. **Table S12** compares synthetic *L*-**1** with natural *L*-**1** isolated by Lin *et al.* **Table S13** compares synthetic *L*-**1** with natural *L*-**1** isolated by the Herzon group. Despite the ^{1}H NMR data matching well, the C5 and C11 ^{13}C signals were noted to be outside experimental error. **Table S14** shows the effect of TFA additive in DMSO-d_6 on the NMR signals. The addition of TFA was found to bring the ^{1}H and ^{13}C signals of our synthetic *L*-**1** closer to Herzon's natural *L*-**1**, with the exception of the C5 and C11 ^{13}C signals which experienced a larger Δδ upon the addition of TFA. **Table S15** compares our synthetic *L*-**1** with natural seongomycin (**32**), a structural analog of *L*-**1**, under near identical NMR solvent conditions. While NMR signals between *L*-**1** and **32** are expectedly different near the site of the structural change, the C5 and C11 signals of *L*-**1** and **32** are within experimental error.

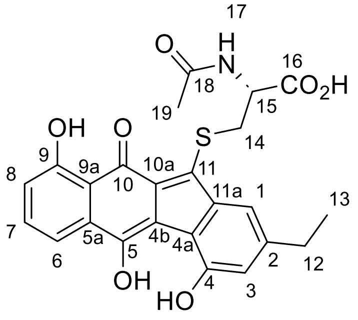

L-**1**

Table S12. Comparison of ^{1}H and ^{13}C NMR data of natural homoseongomycin (*L*-**1**) isolated by Lin et al. and synthetic homoseongomycin (*L*-**1**).

	[a] homoseongomycin (*natural*)[35]	[b] homoseongomycin (*synthetic, this work*)	
Position	δH	δH (500 MHz)	ΔδH
1	7.18	7.15	0.03
3	6.78	6.73	0.05
6	7.48	7.36	0.12
7	7.52	7.54	-0.02
8	6.95	6.97	-0.02
9-OH	13.70	13.49	0.21
12	2.60	2.6	0.00
13	1.18	1.19	-0.01
14	3.90	3.86	0.04
14	3.50	3.51	-0.01
15	4.45	4.42	0.03
17	8.30	8.29	0.01
19	1.71	1.72	-0.01
Position	δC (125 MHz)	δC (125 MHz)	ΔδC

1	116.2	115.7	0.5
2		144.9	
3	116.1	114.3	1.8
4		149.3	
4a		120.4	
4b		115.9	
5		149.0	
5a		133.9	
6	116.1	116.1	0.0
7	136.0	136.0	0.0
8	119.8	119.7	0.1
9		162.9	
9a		overlap w/ TFA	
10		184.0	
10a		128.4	
11		145.1	
11a		141.7	
12	28.7	28.4	0.3
13	15.5	15.6	-0.1
14	34.9	34.8	0.1
15	52.6	52.4	0.2
16		171.8	
18		169.4	
19	22.2	22.2	0.0

[b] DMSO-d_6+TFA, 25 °C

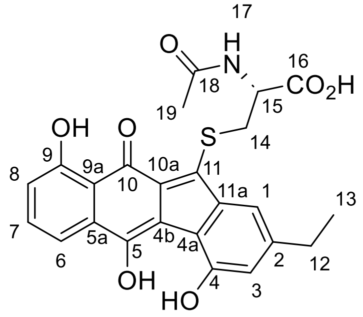

***L*-1**

Table S13. Comparison of ^{1}H and ^{13}C NMR data of natural homoseongomycin (*L*-**1**) isolated by Herzon group and synthetic homoseongomycin (*L*-**1**).

	[a] homoseongomycin (*natural*)[34]	[b] homoseongomycin (*synthetic, this work*)	
Position	δH (400 MHz)	δH (500 MHz)	ΔδH
1	7.09	7.15	-0.06

3	6.63	6.73	-0.10
6	7.36	7.36	0.00
7	7.52	7.54	-0.02
8	6.95	6.97	-0.02
9-OH	13.54	13.49	0.05
12	2.58	2.6	-0.02
13	1.19	1.19	0.00
14	3.80	3.86	-0.06
14	3.45	3.51	-0.06
15	4.37	4.42	-0.05
17	8.27	8.29	-0.02
19	1.73	1.72	0.01

Position	δC (125 MHz)	δC (125 MHz)	ΔδC
1	114.4	115.7	-1.3
2	143.9	144.9	-1.0
3	114.3	114.3	0.0
4	149.9	149.3	0.6
4a	121.2	120.4	0.8
4b	114.8	115.9	-1.1
5	152.5	149.0	3.5
5a	134.7	133.9	0.8
6	116.1	116.1	0.0
7	135.7	136.0	-0.3
8	119.7	119.7	0.0
9	162.8	162.9	-0.1
9a	116.7	overlap w/ TFA	-
10	184.3	184.0	0.3
10a	128.4	128.4	0.0
11	141.3	145.1	-3.8
11a	140.6	141.7	-1.1
12	28.5	28.4	0.1
13	15.6	15.6	0.0
14	34.9	34.8	0.1
15	52.4	52.4	0.0
16	171.8	171.8	0.0
18	169.3	169.4	-0.1
19	22.2	22.2	0.0

[a] DMSO-d_6, 24 °C
[b] DMSO-d_6+TFA, 25 °C

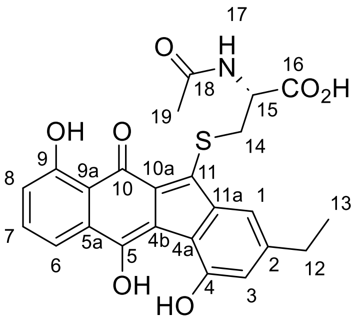

***L*-1**

Table S14. Comparison of ^{1}H and ^{13}C NMR data of synthetic homoseongomycin (*L*-**1**) with and without TFA additive.

	[a] homoseongomycin (*synthetic, this work*)	[b] homoseongomycin (*synthetic, this work*)	
Position	δH (500 MHz)	δH (500 MHz)	ΔδH
1	6.94	7.15	-0.21
3	6.42	6.73	-0.31
6	7.38	7.36	0.02
7	7.48	7.54	-0.06
8	6.92	6.97	-0.05
9-OH	13.66	13.49	0.17
12	2.55	2.6	-0.05
13	1.18	1.19	-0.01
14	3.45	3.86	-0.41
14	3.30	3.51	-0.21
15	4.28	4.42	-0.14
17	8.19	8.29	-0.10
19	1.74	1.72	0.02
Position	δC (125 MHz)	δC (125 MHz)	ΔδC
1	112.2	115.7	-3.5
2	142.0	144.9	-2.9
3	112.6	114.3	-1.7
4	151.2	149.3	1.9
4a	122.4	120.4	2.0
4b	114.5	115.9	-1.4
5	151.3	149.0	2.3
5a		133.9	
6	116.4	116.1	0.3
7	135.2	136.0	-0.8
8	119.6	119.7	-0.1
9		162.9	

9a	116.7	overlap w/ TFA	
10	185.2	184.0	1.2
10a	128.4	128.4	0.0
11	142.0	145.1	-3.1
11a	140.8	141.7	-0.9
12	28.5	28.4	0.1
13	15.6	15.6	0.0
14	35.2	34.8	0.4
15	52.4	52.4	0.0
16	172.0	171.8	0.2
18	169.1	169.4	-0.3
19	22.2	22.2	0.0

[a] DMSO-d_6, 25 °C
[b] DMSO-d_6+TFA, 25 °C

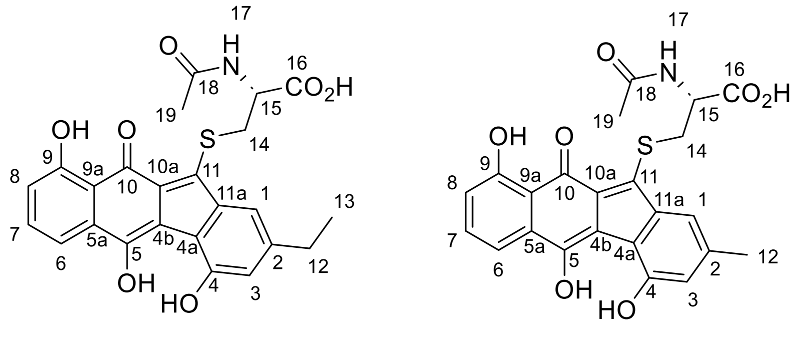

***L*-1** **32**

Table S15. Comparison of ^{1}H and ^{13}C NMR data of natural seongomycin (**32**) and synthetic homoseongomycin (*L*-**1**).

	[a] seongomycin (*natural*)[36]	[b] homoseongomycin (*synthetic, this work*)	
Position	δH (500 MHz)	δH (500 MHz)	ΔδH
1	7.12	7.15	-0.03
3	6.70	6.73	-0.03
6	7.30	7.36	-0.06
7	7.50	7.54	-0.04
8	6.91	6.97	-0.06
9-OH	13.60	13.49	0.11
12	2.27	2.6	-0.33
13	*n/a*	1.19	*n/a*
14	3.86	3.86	0.00
14	3.53	3.51	0.02
15	4.43	4.42	0.01
17	8.23	8.29	-0.06

19	1.73	1.72	0.01
Position	δC (125 MHz)	δC (125 MHz)	ΔδC
1	117.4	115.7	1.7
2	138.7	144.9	-6.2
3	117.1	114.3	2.8
4	149.2	149.3	-0.1
4a	120.5	120.4	0.1
4b	114.1	115.9	-1.8
5	148.2	149.0	-0.8
5a	133.9	133.9	0.0
6	116.1	116.1	0.0
7	135.9	136.0	-0.1
8	119.7	119.7	0.0
9	163.1	162.9	0.2
9a	116.0	overlap w/ TFA	
10	184.0	184.0	0.0
10a	128.4	128.4	0.0
11	146.1	145.1	1.0
11a	141.8	141.7	0.1
12	21.3	28.4	-7.1
13		15.6	
14	34.8	34.8	0.0
15	52.6	52.4	0.2
16	171.9	171.8	0.1
18	169.7	169.4	0.3
19	22.3	22.2	0.1

[a] DMSO-d_6+1 drop TFA-d, 24 °C
[b] DMSO-d_6+TFA, 25 °C

Spectra

Compound 1 ((*L*)-Homoseongomycin)

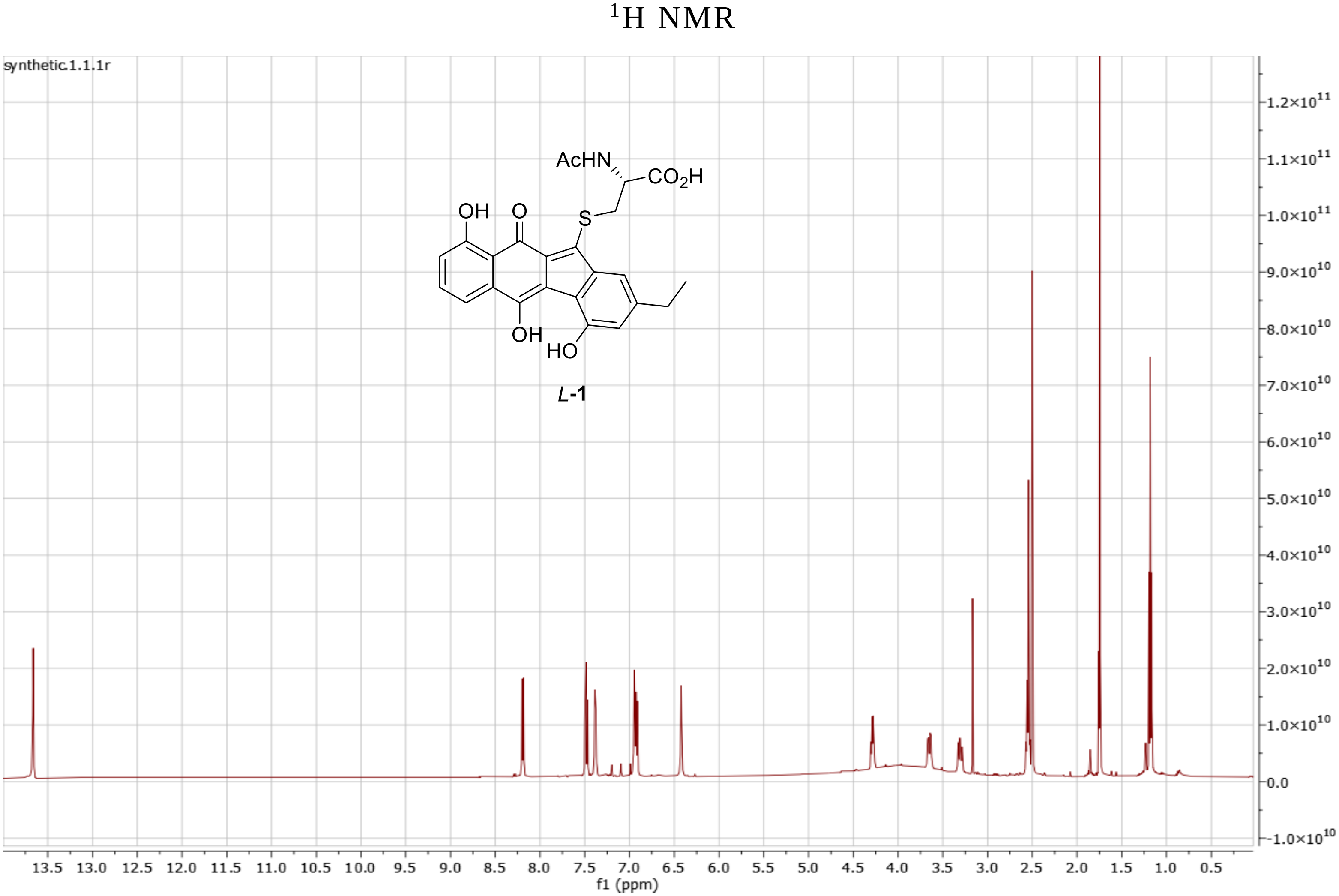

Figure S22. ^{1}H NMR spectrum of compound *L*-**1** in DMSO-d6.

^{1}H NMR

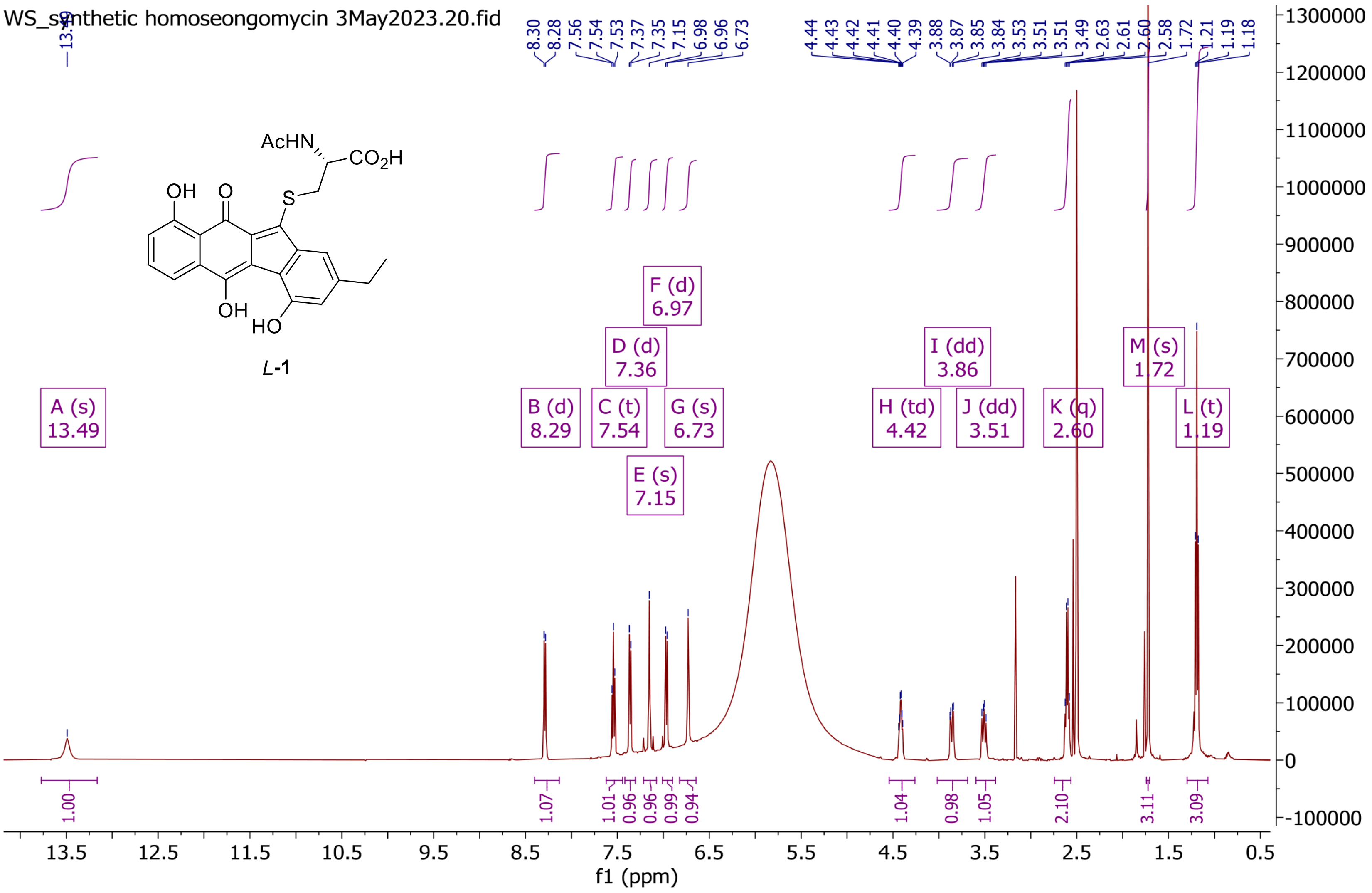

Figure S23. ^{1}H NMR spectrum of compound *L*-**1** in DMSO-d6 + TFA additive.

^{1}H NMR

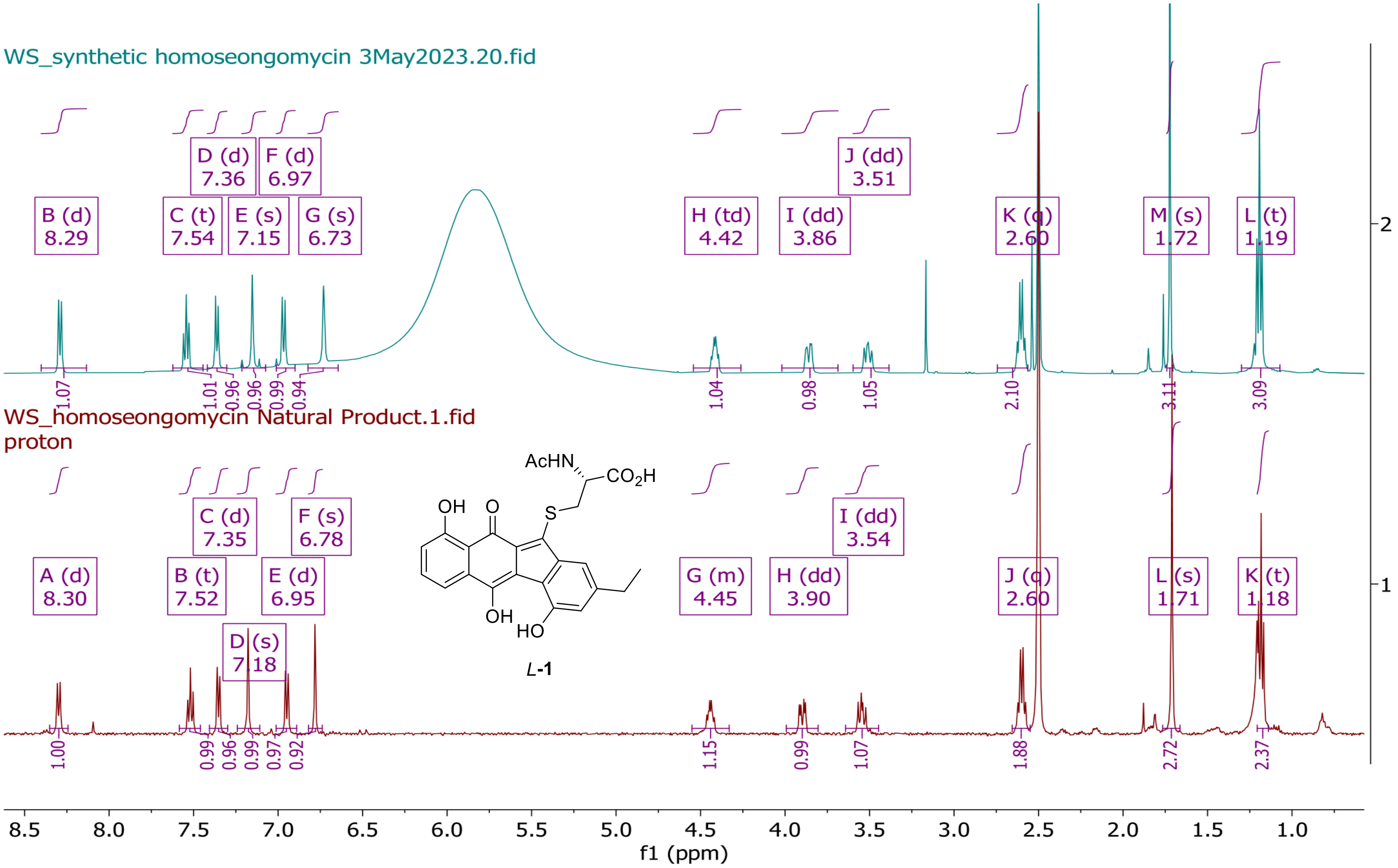

Figure S24. Overlaid ^{1}H NMR spectrums of compound *L*-**1** in DMSO-d6. Top spectrum (cyan): synthetic homoseongomycin + TFA additive. Bottom spectrum (red): synthetic homoseongomycin.

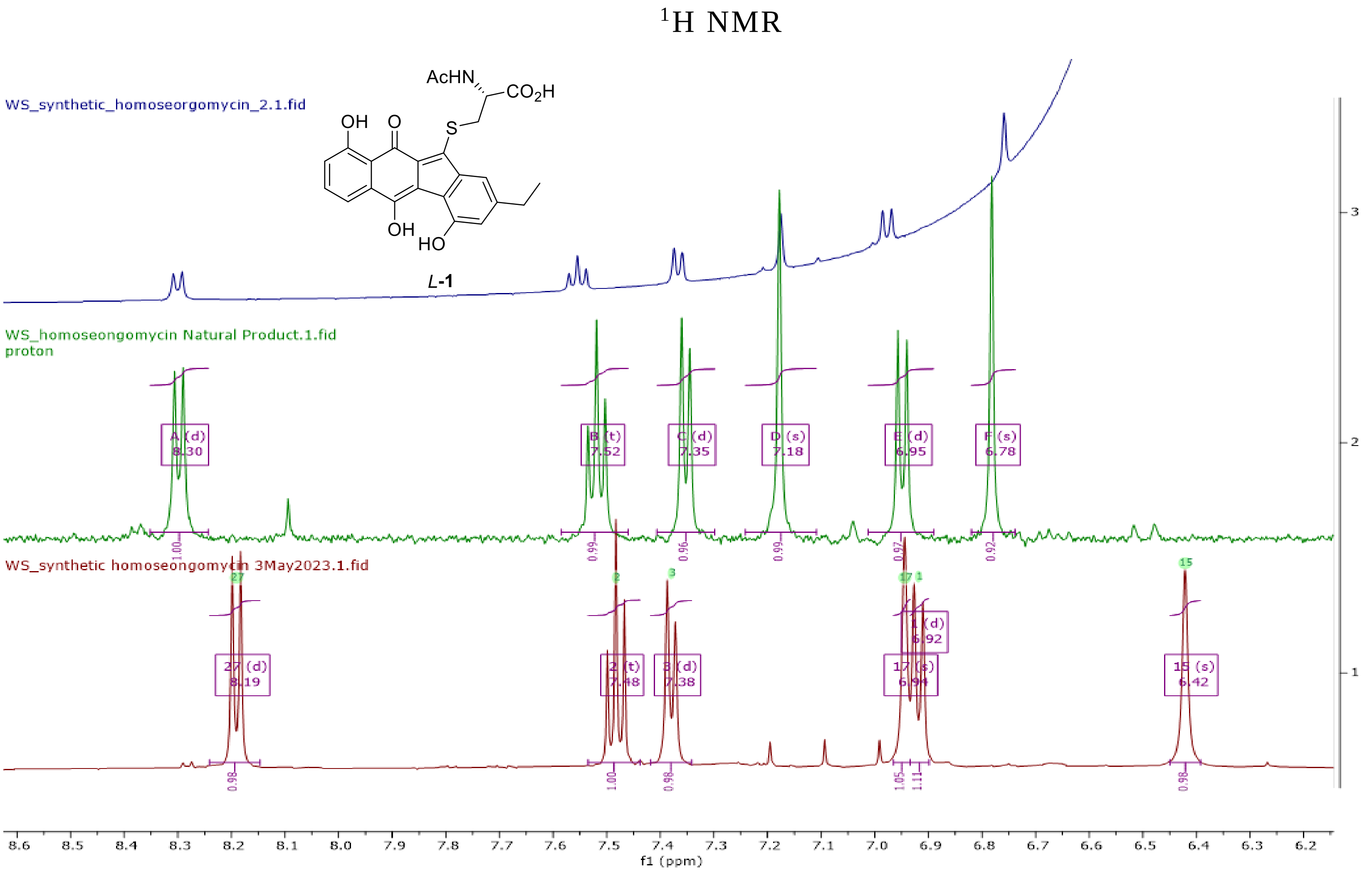

Figure S25. Overlaid ^{1}H NMR spectrums of compound *L*-**1** in DMSO-d6. Top spectrum (blue): synthetic homoseongomycin + TFA additive. Middle spectrum (green): natural homoseongomycin. Bottom spectrum (red): synthetic homoseongomycin.

^{13}C NMR

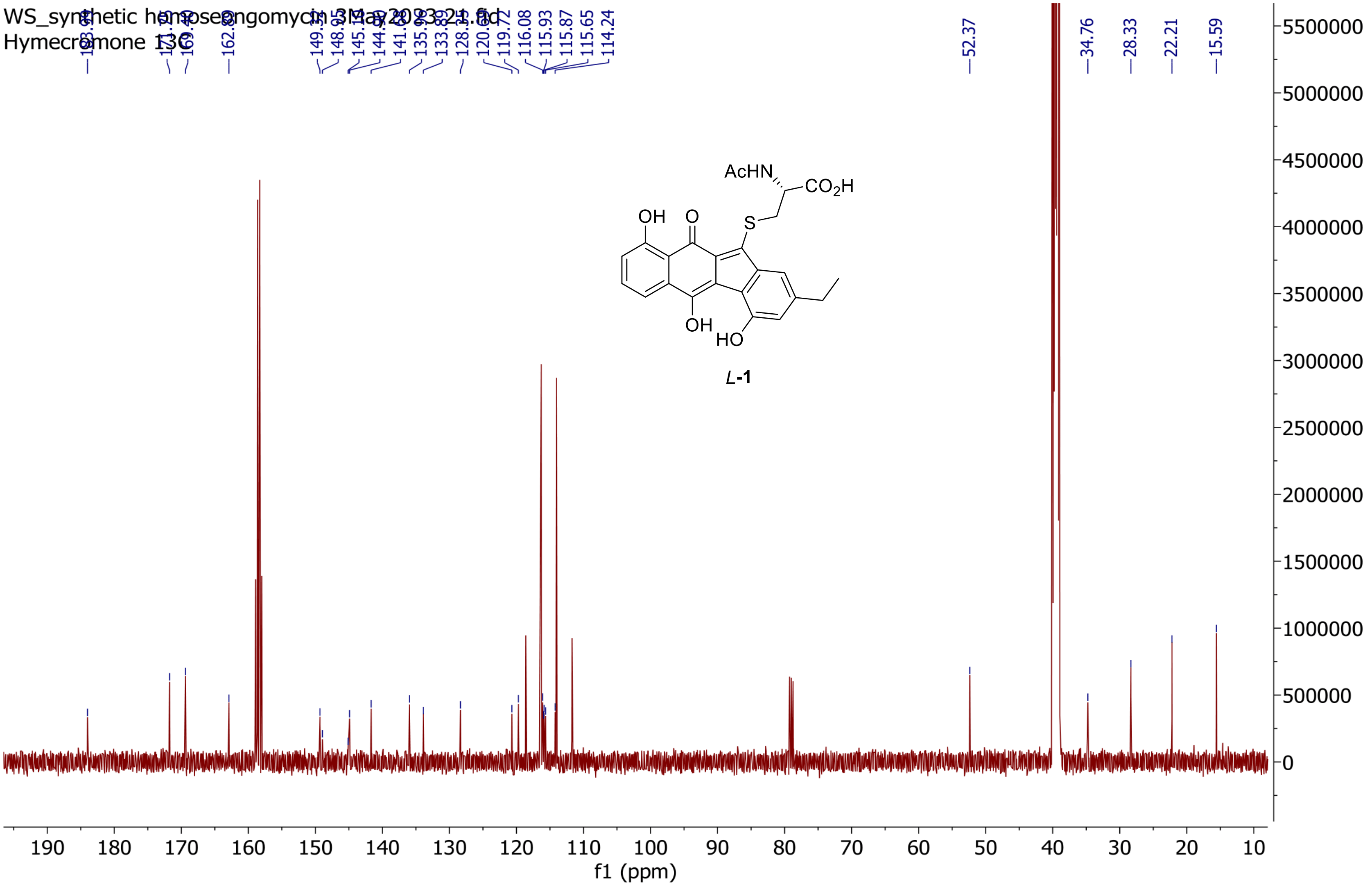

Figure S26. ^{13}C NMR spectrum of compound *L*-**1** in DMSO-d6.

HMBC

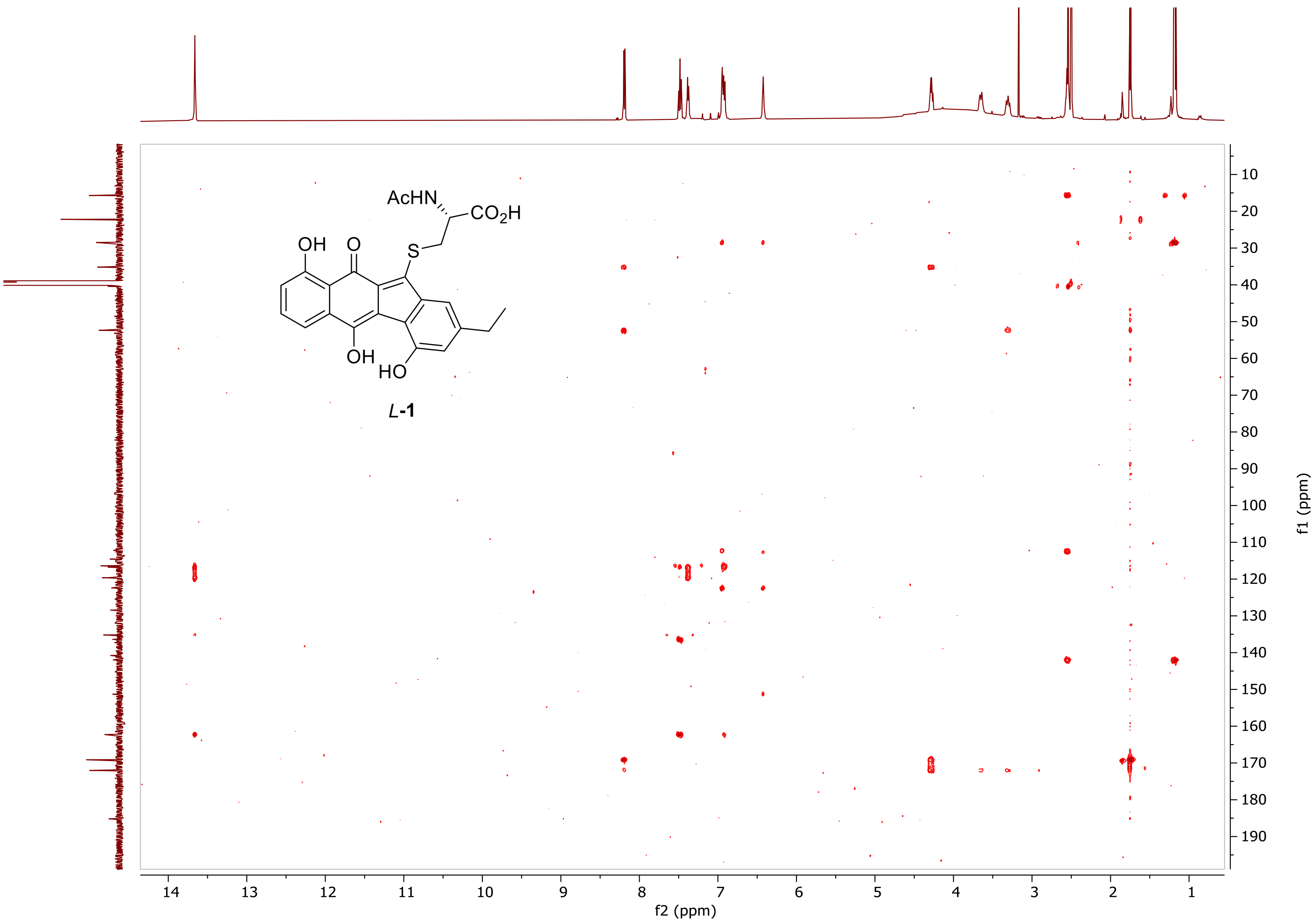

Figure S27. HMBC NMR spectrum of compound *L*-**1** in DMSO-d6.

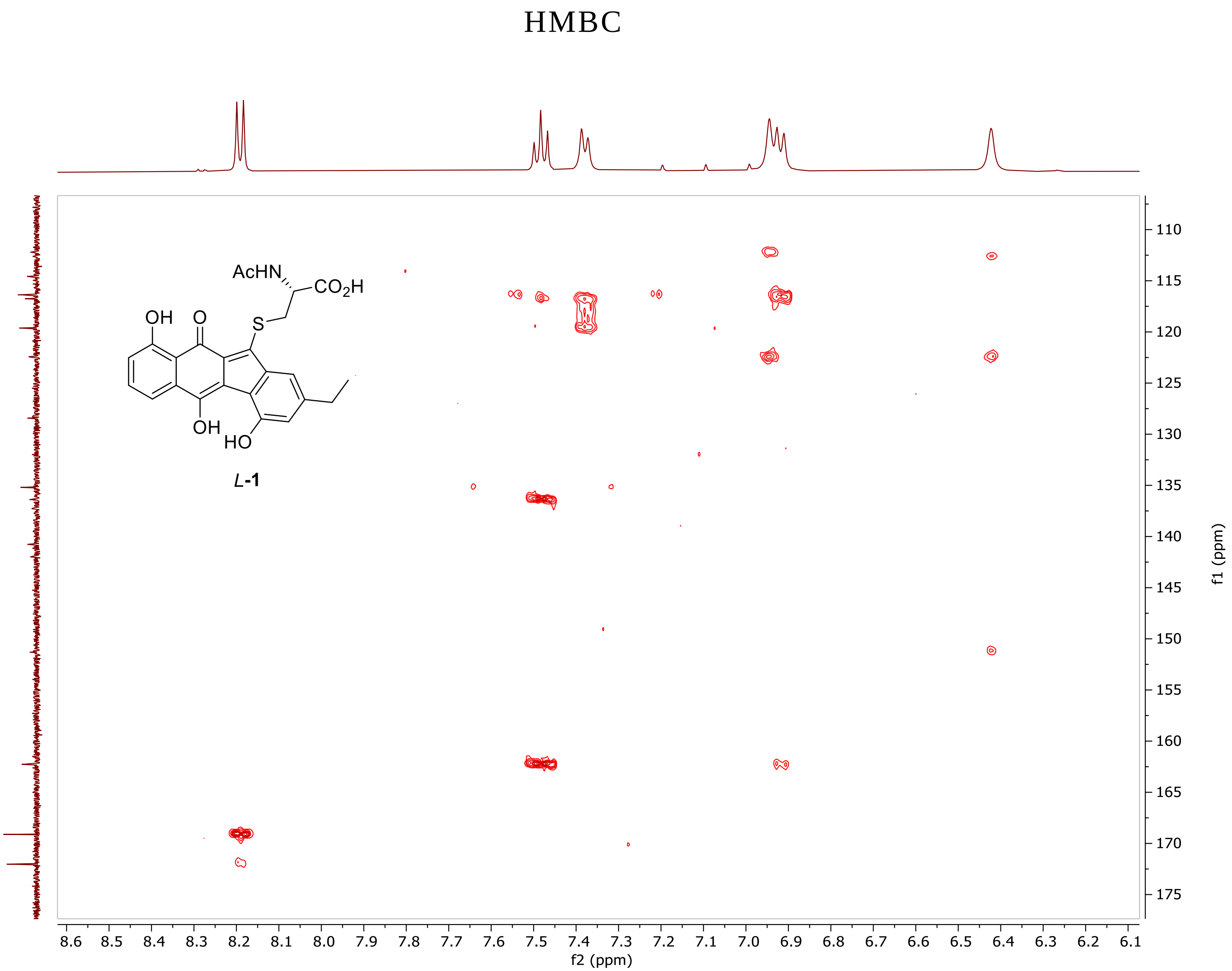

Figure S28. Zoomed in HMBC NMR spectrum of compound *L*-**1** in DMSO-d6.

HMBC

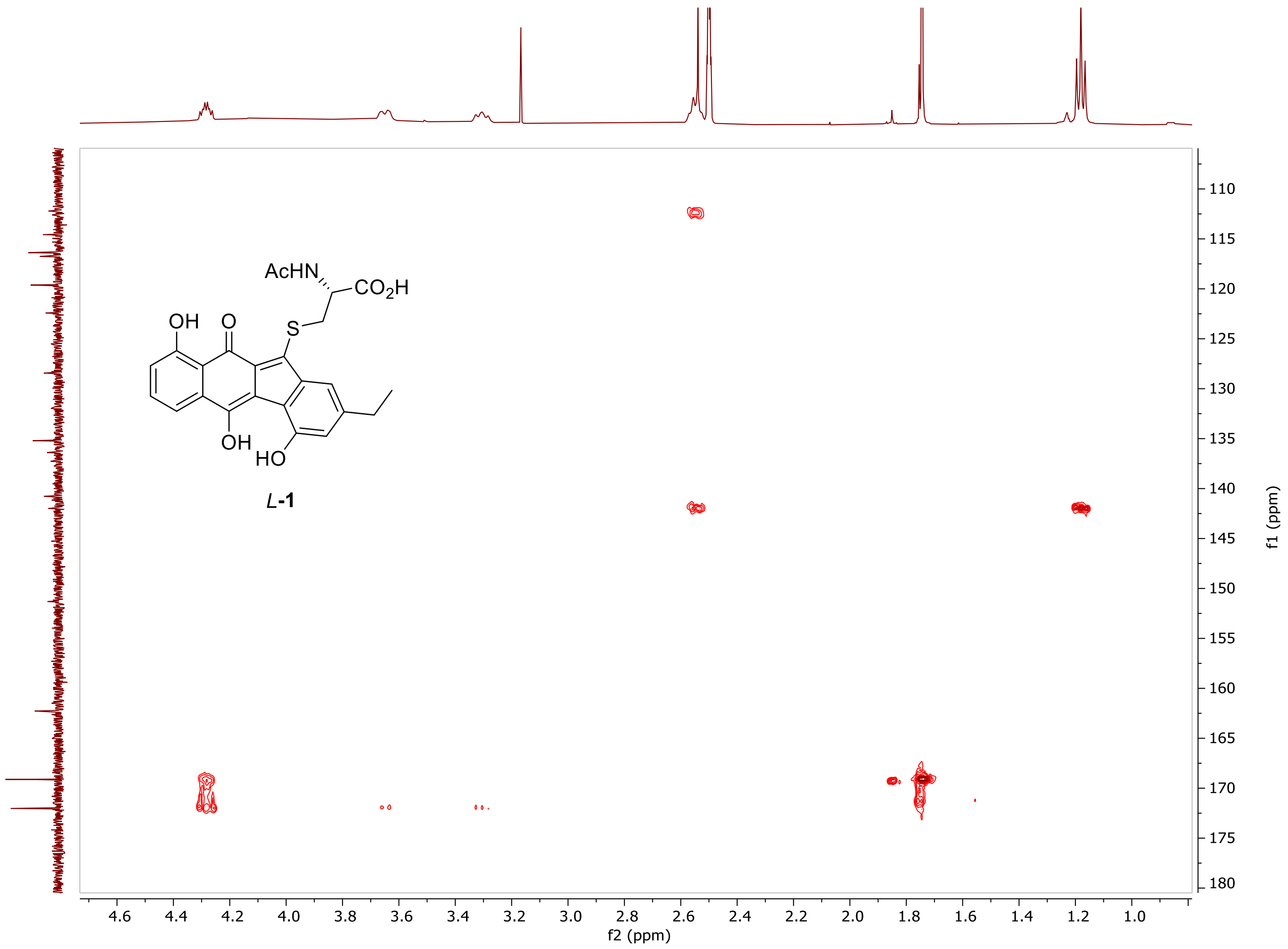

Figure S29. Zoomed in HMBC NMR spectrum of compound *L*-**1** in DMSO-d6.

EASY-ROESY

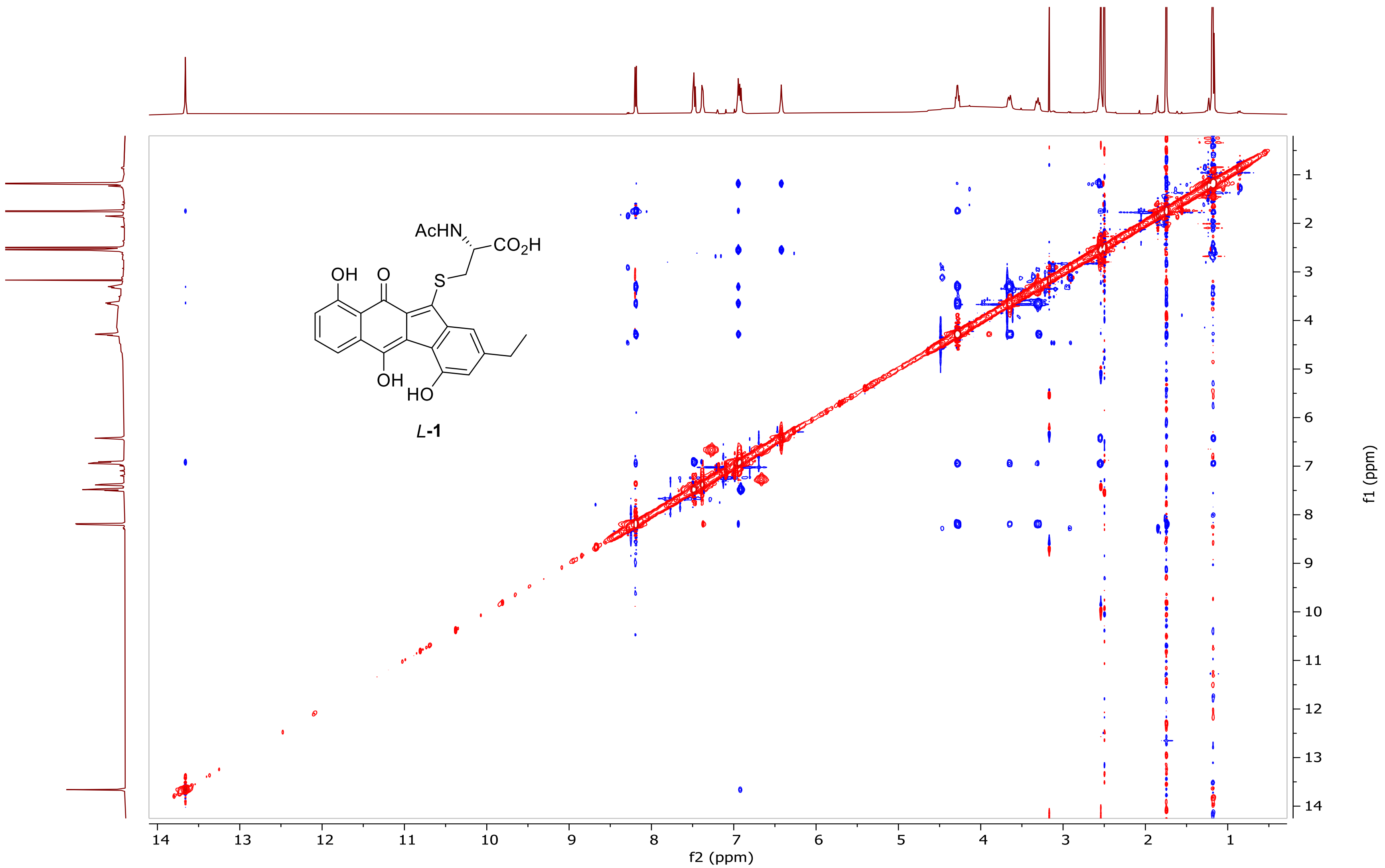

Figure S30. EASY-ROESY NMR spectrum of compound *L*-**1** in DMSO-d6.

EASY-ROESY

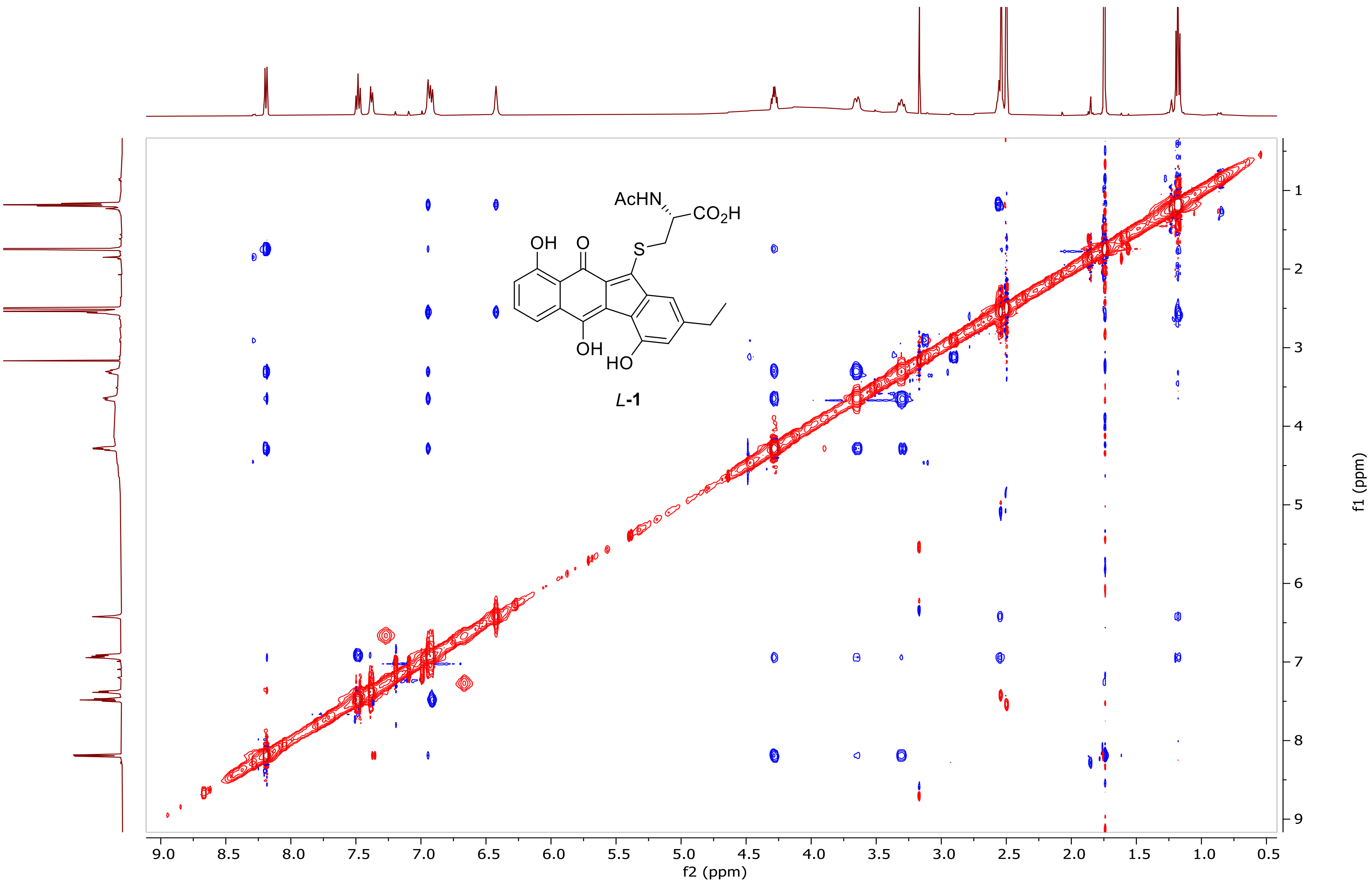

Figure S31. Zoomed in EASY-ROESY NMR spectrum of compound *L*-**1** in DMSO-d6.

LCMS

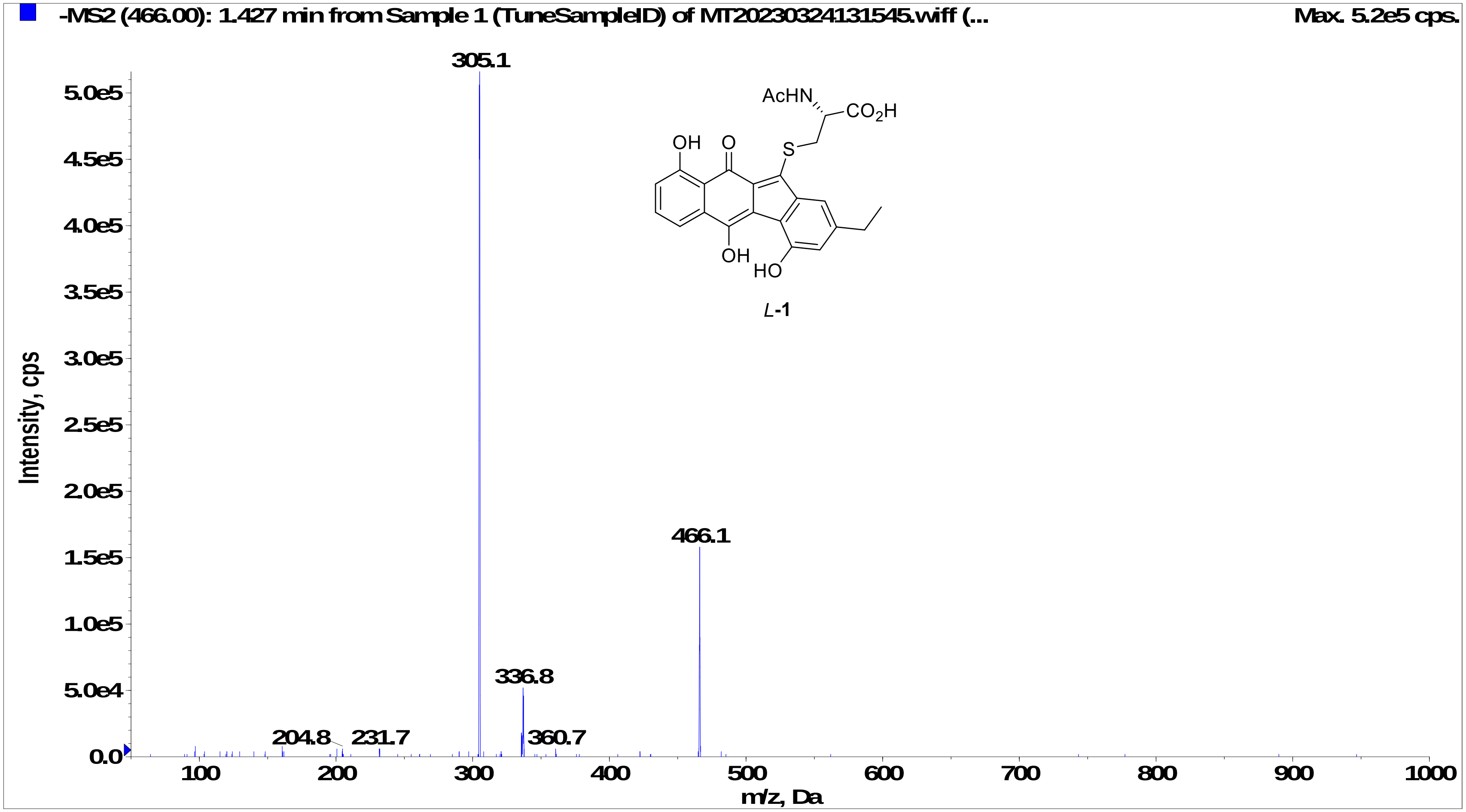

Figure S32. LCMS spectrum of compound *L*-**1**. Polarity: negative

HRMS

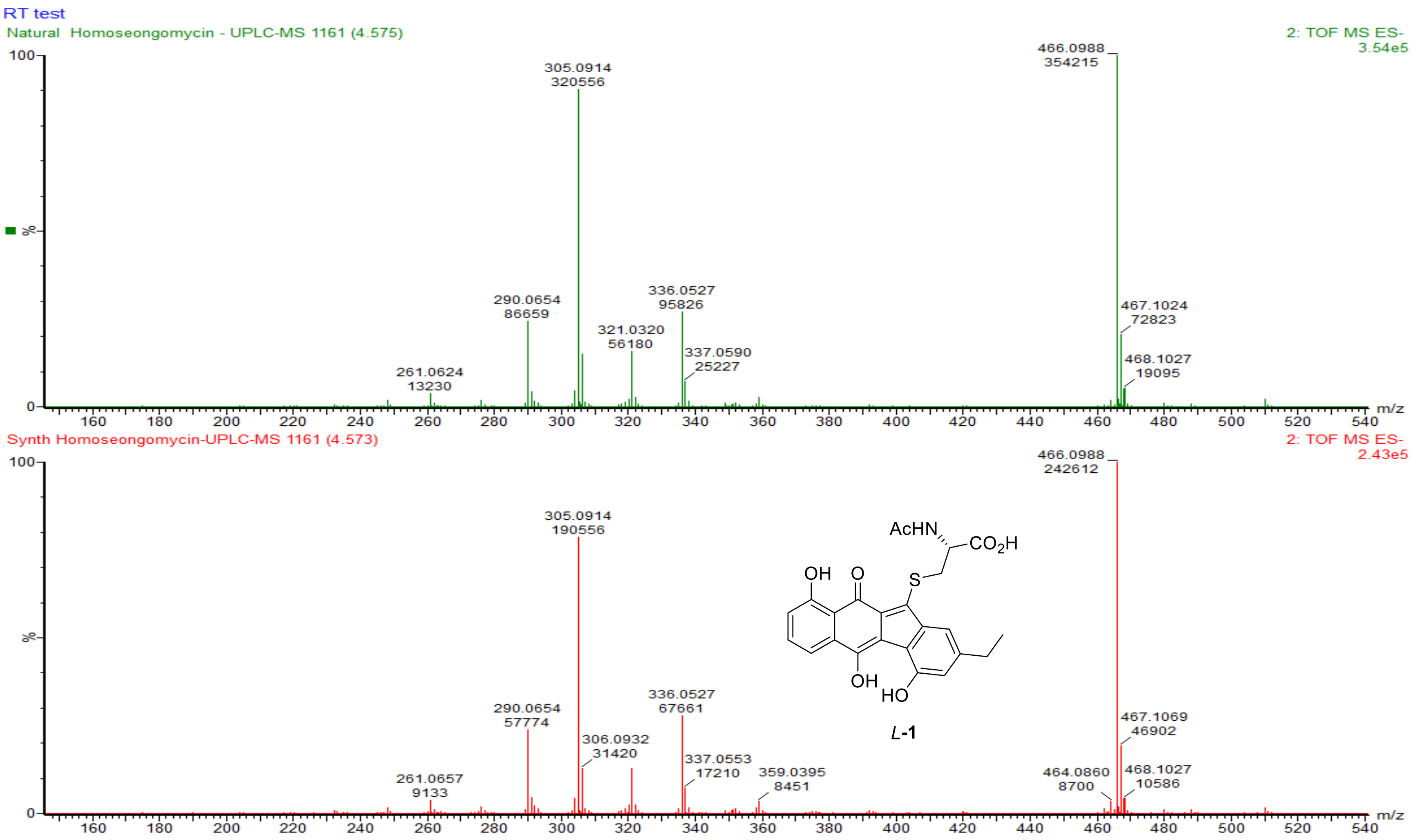

Figure S33. Overlaid HRMS spectrums of compound *L*-**1**. Top spectrum (green): natural homoseongomycin. Bottom spectrum (red): synthetic homoseongomycin.

IR

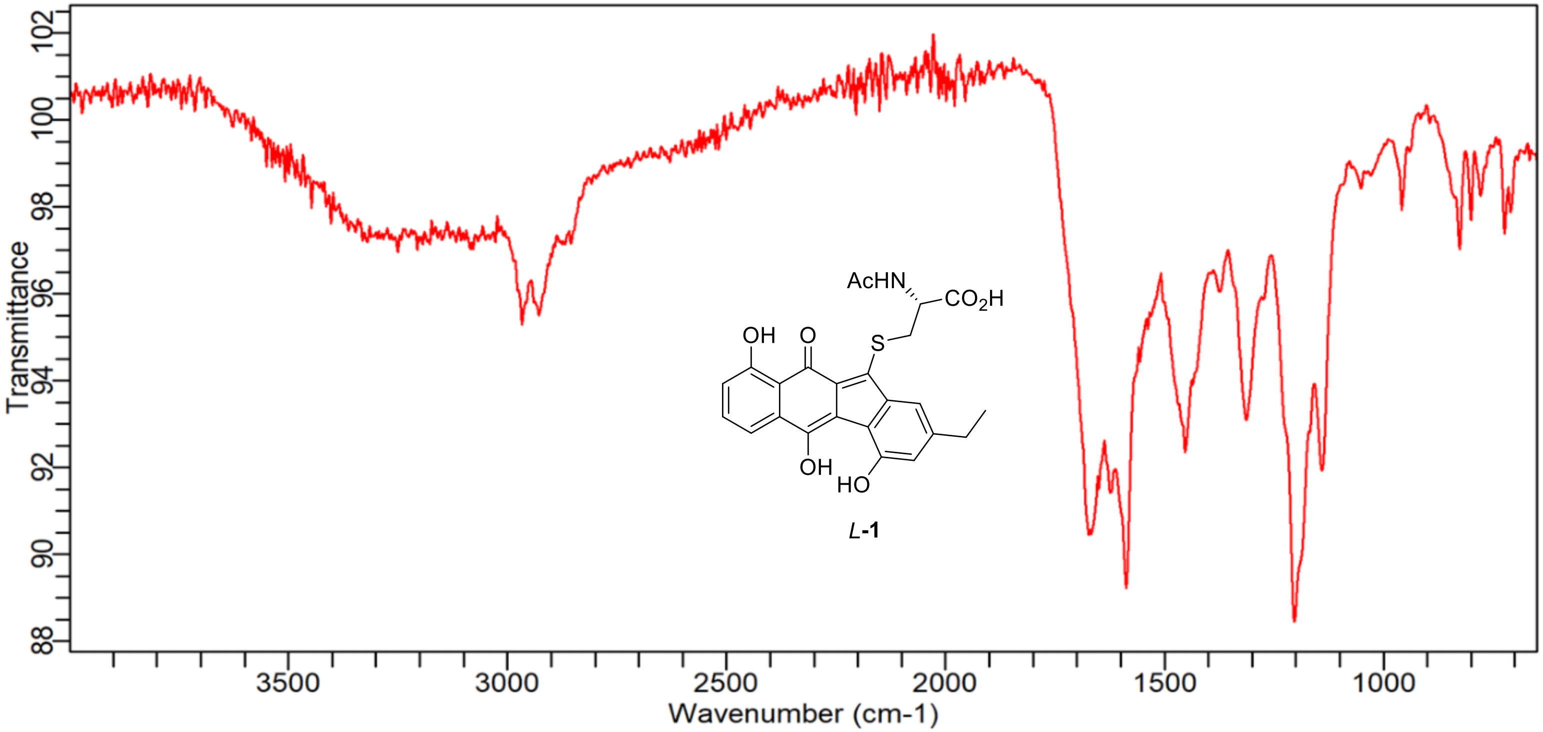

Figure S34. ATR-IR spectrum of *L*-**1**.

UPLC

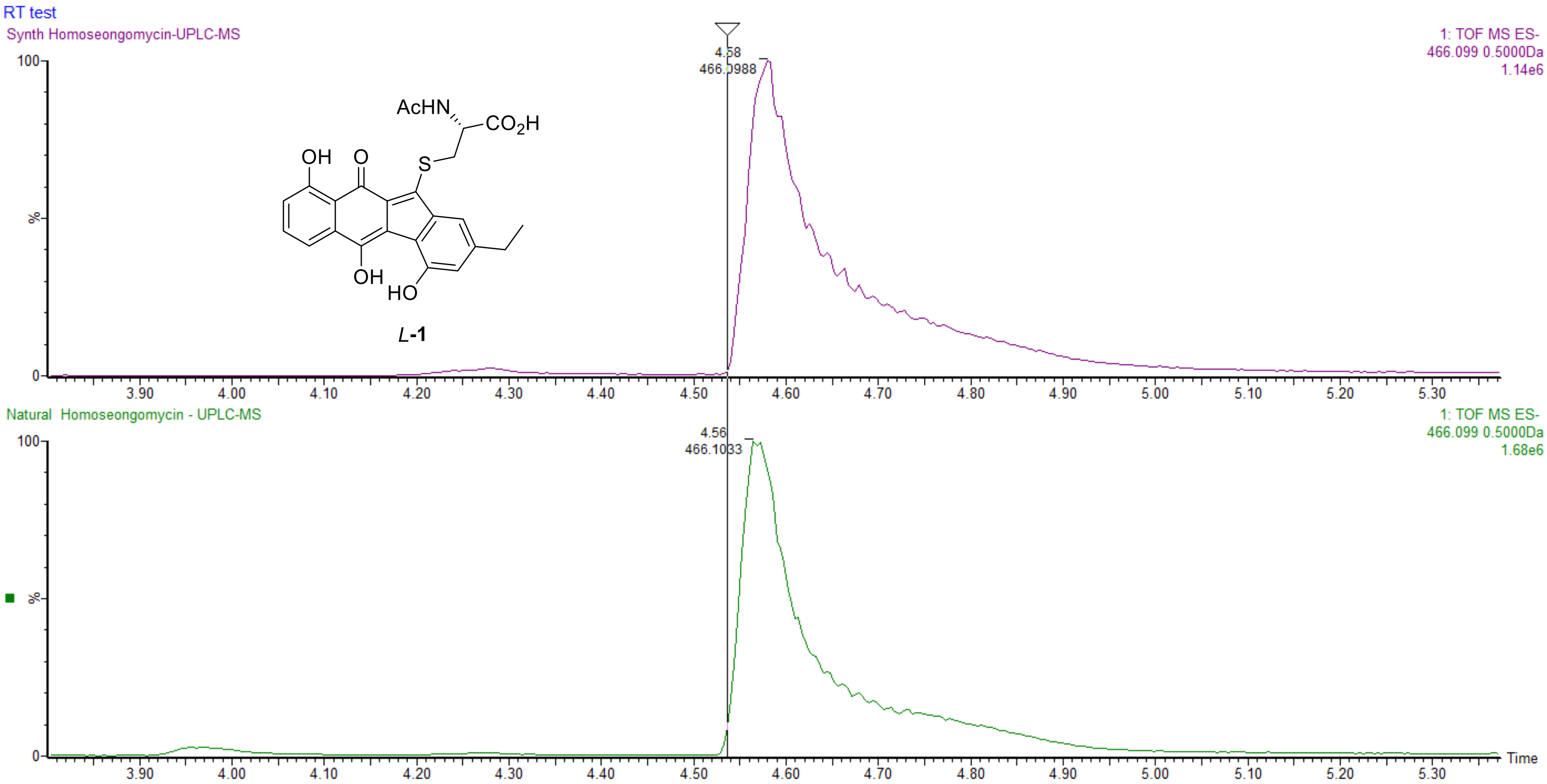

Figure S35. Overlaid UPLC spectrums of *L*-**1**. Top spectrum (purple): synthetic homoseongomycin. Bottom spectrum (green): natural homoseongomycin.

Prep-LC Chromatogram

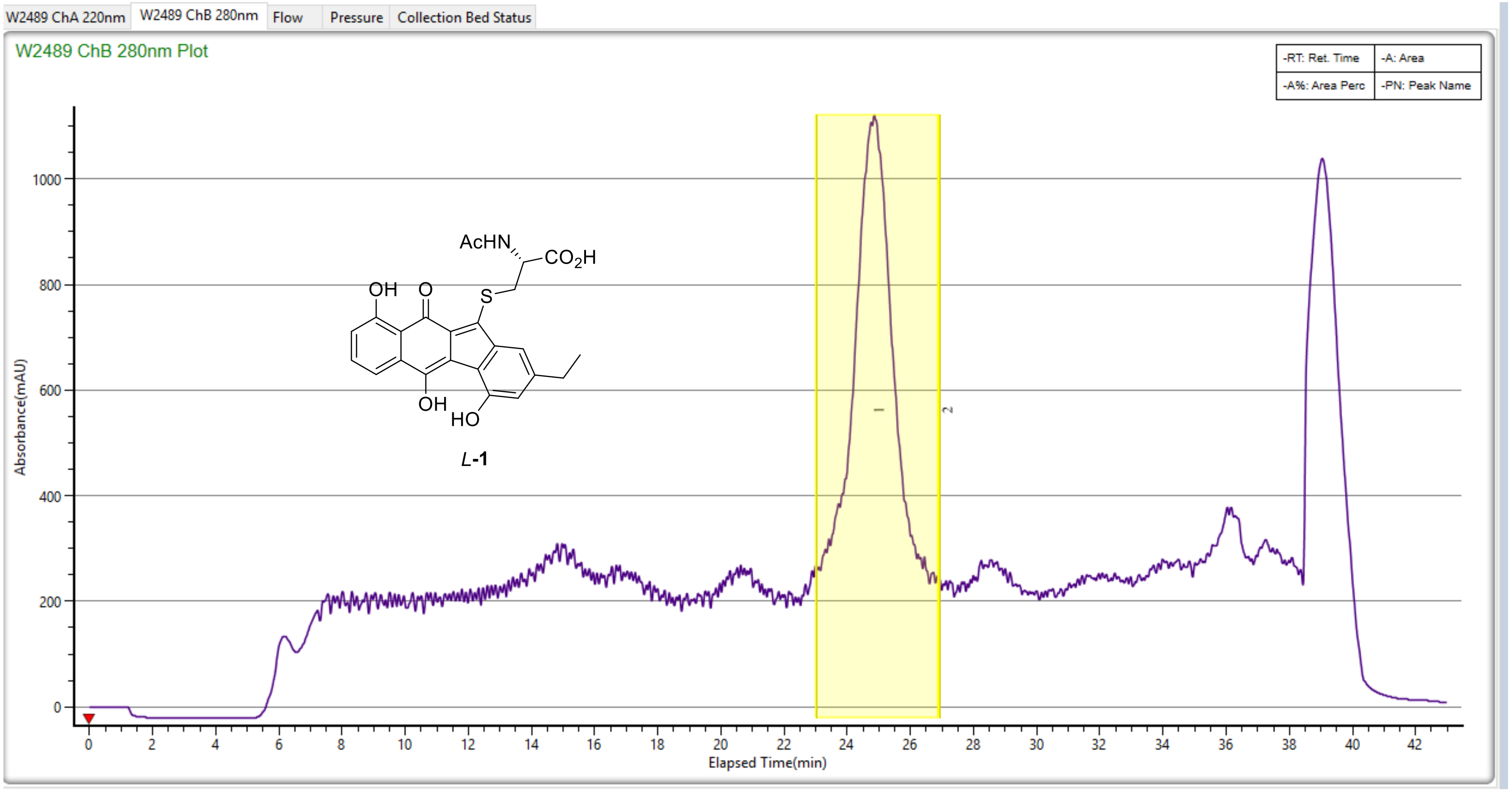

Figure S36. Chromatogram of preparatory reverse-phase chromatography purification of *L*-**1**. Wavelengths monitored: 220 nm, 280 nm.

HPLC-UV Trace

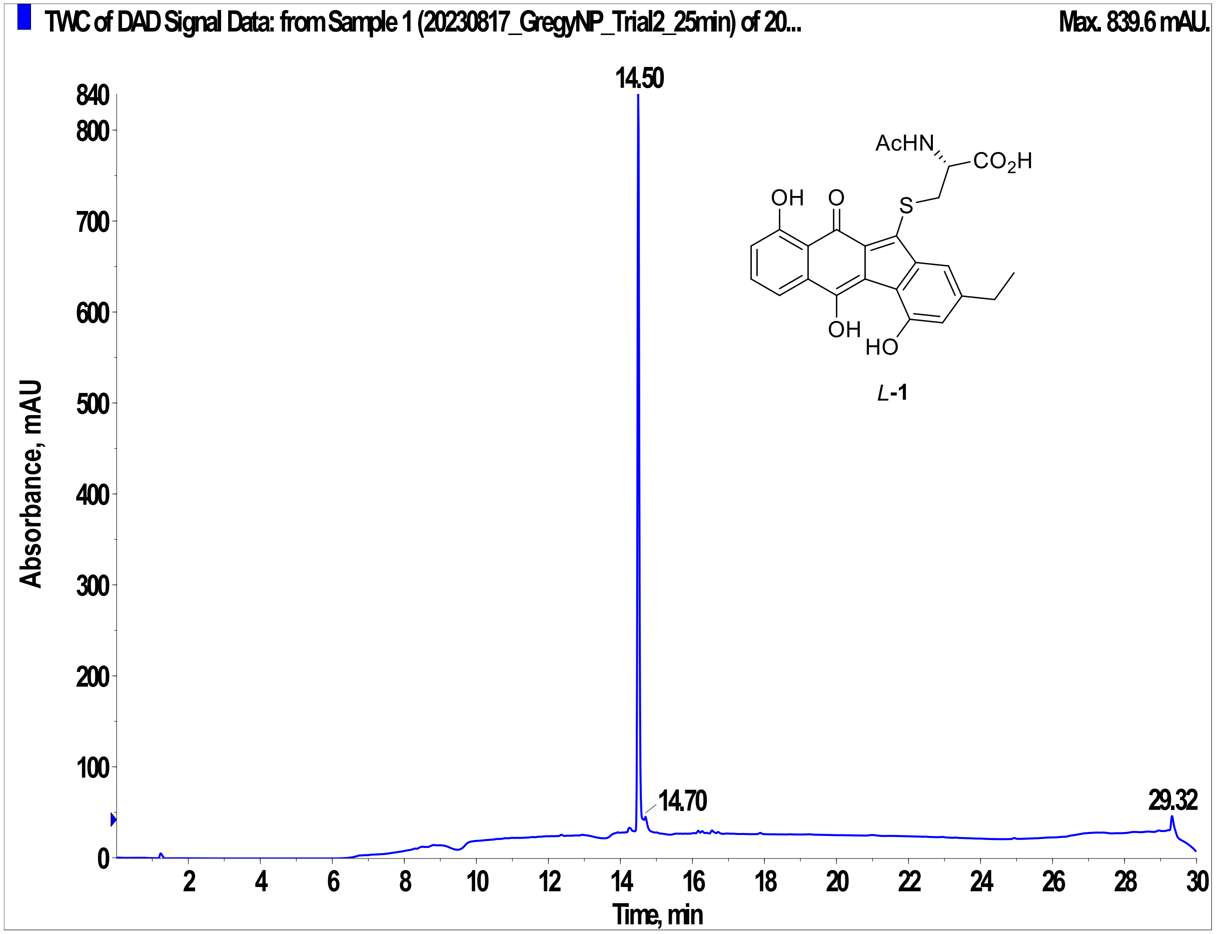

L-**1**

Figure S37. Analytical HPLC chromatogram of purified *L*-**1** to validate purity. Wavelength monitored: 254 nm.

UV-Vis

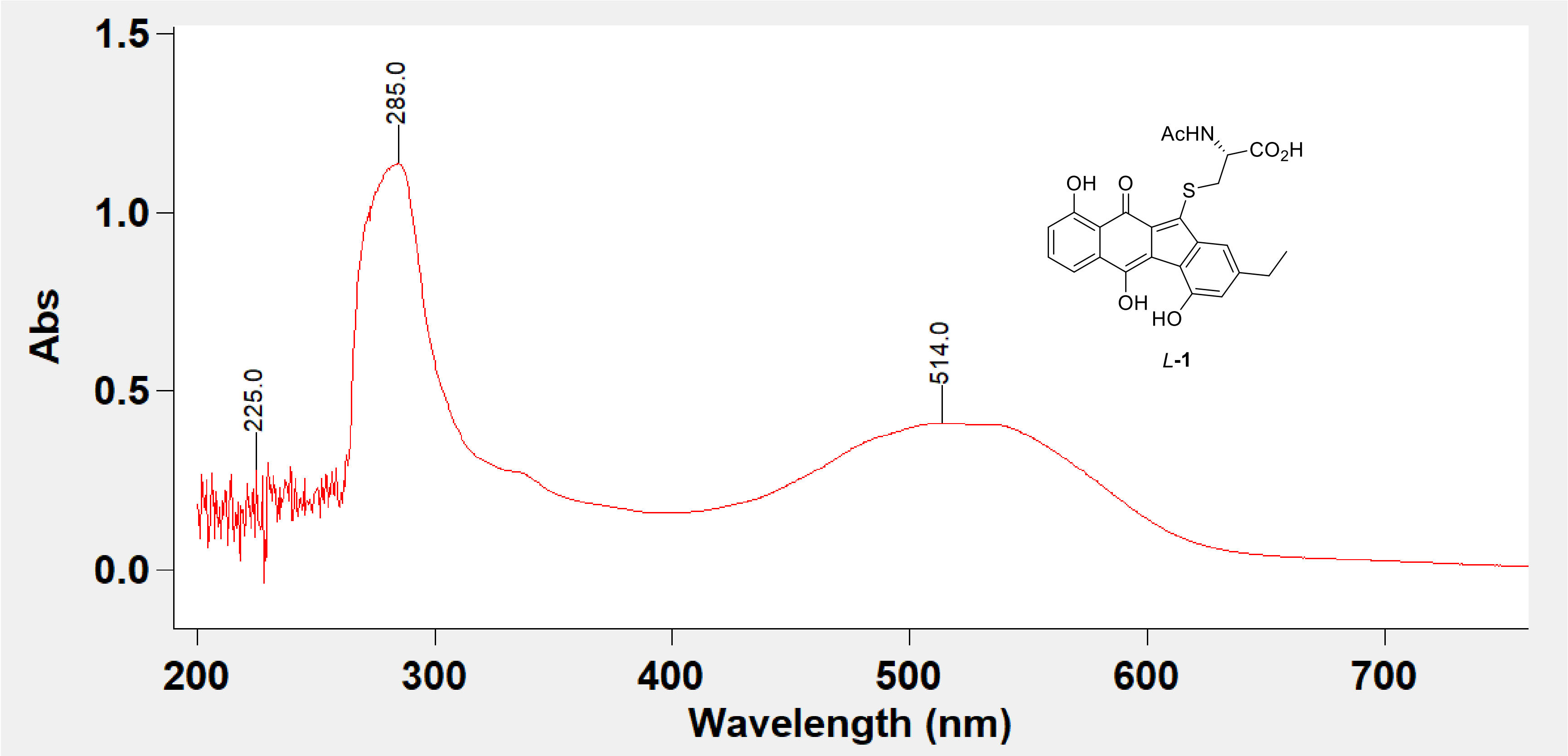

Figure S38. UV-Vis spectrum of *L*-**1**. Concentration: 0.1 mg/mL in MeOH. Cuvette: 10 mm.

ECD

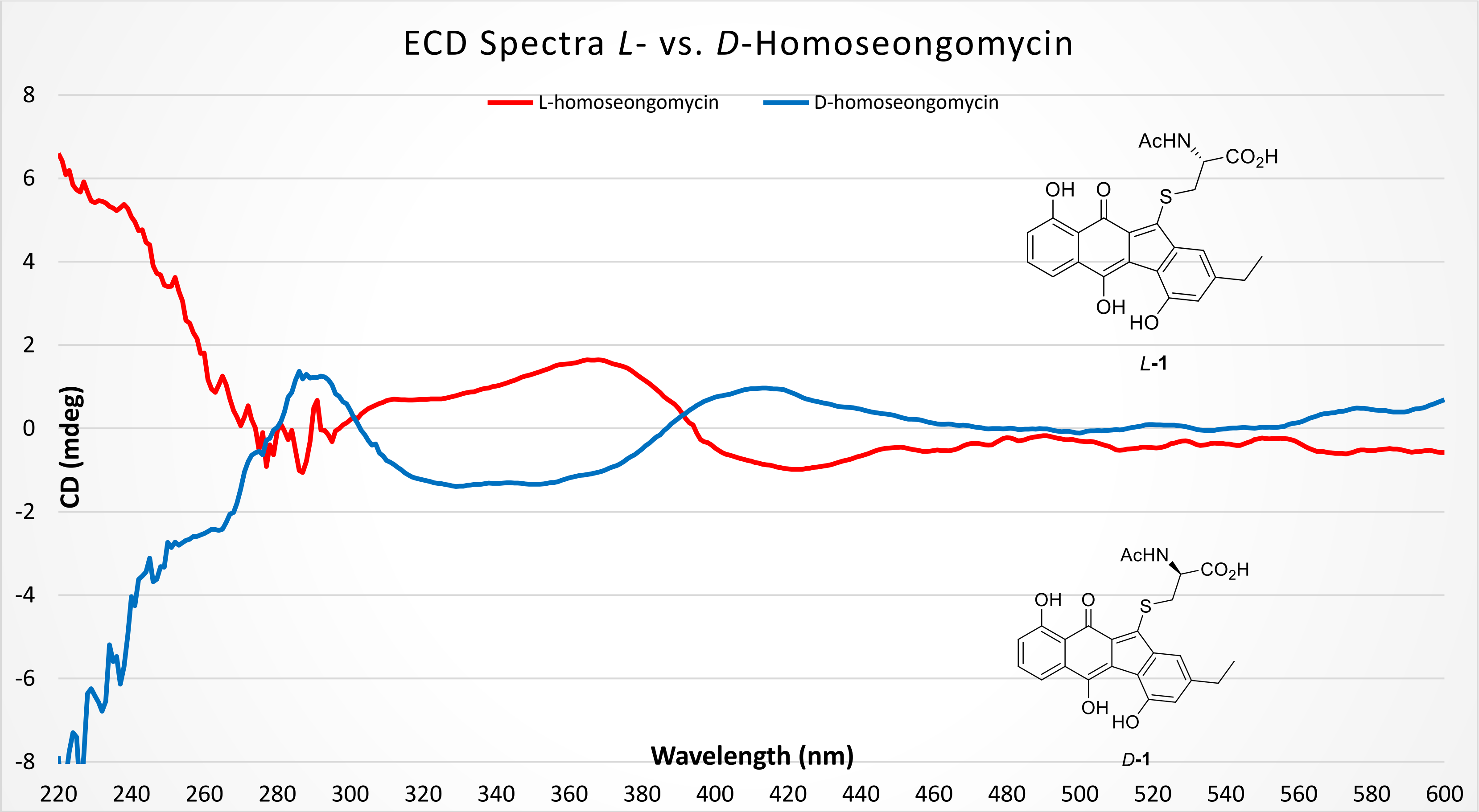

Figure S39. ECD spectra of *L*-**1** and *D*-**1**. Concentration: 0.7 mg/mL in MeOH. Bandwidth: 1.0 nm. D.I.T: 4 sec. Cuvette: 1mm. Scanning speed: 50 nm/min.

KK Transform ORD

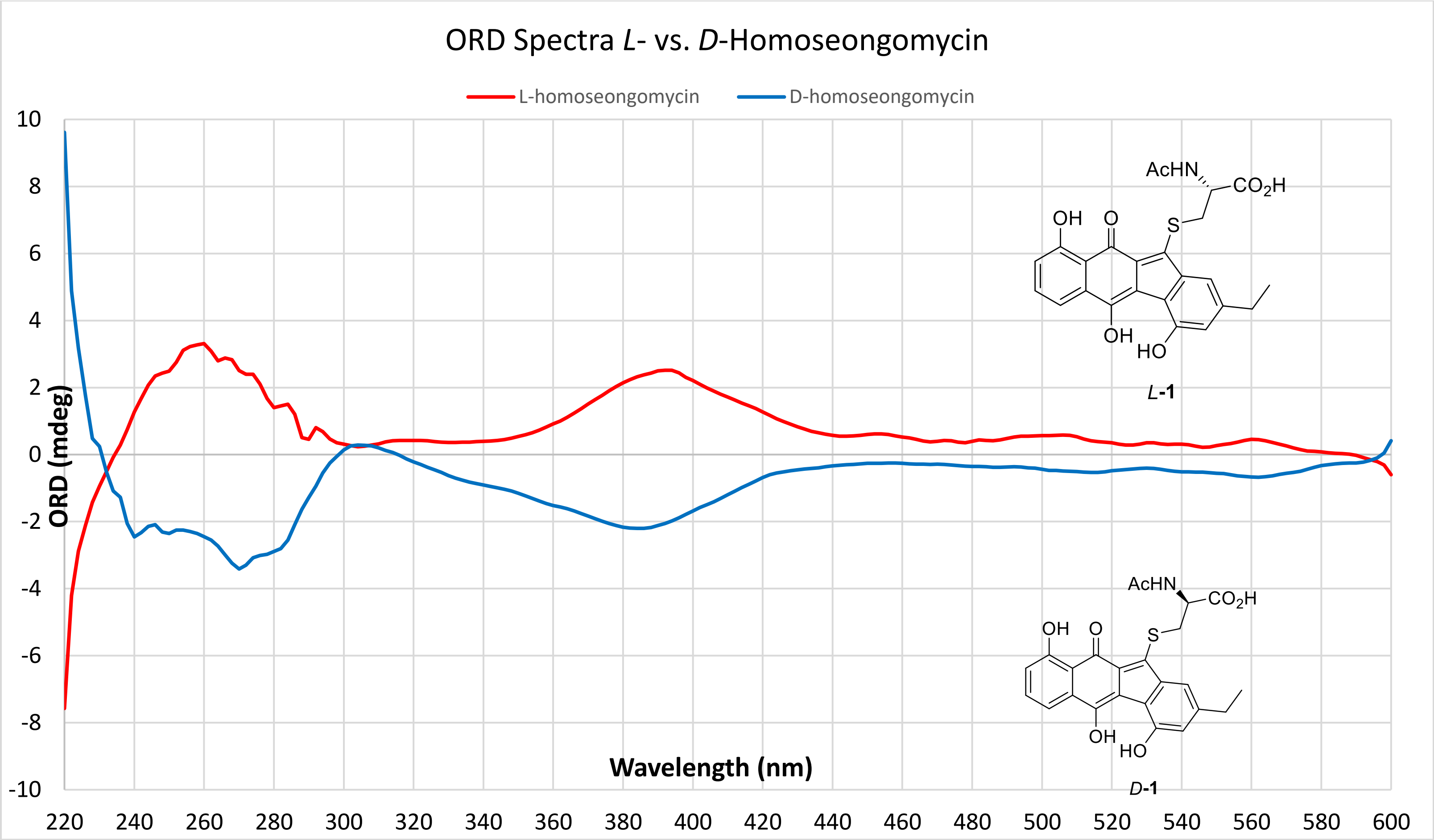

Figure S40. Transformed ECD spectra of *L*-**1** and *D*-**1** into corresponding ORD spectra using KK transform function in Jasco CD spectrometer.

Compound 1 ((*D*)-Homoseongomycin)

HPLC-LCMS Trace

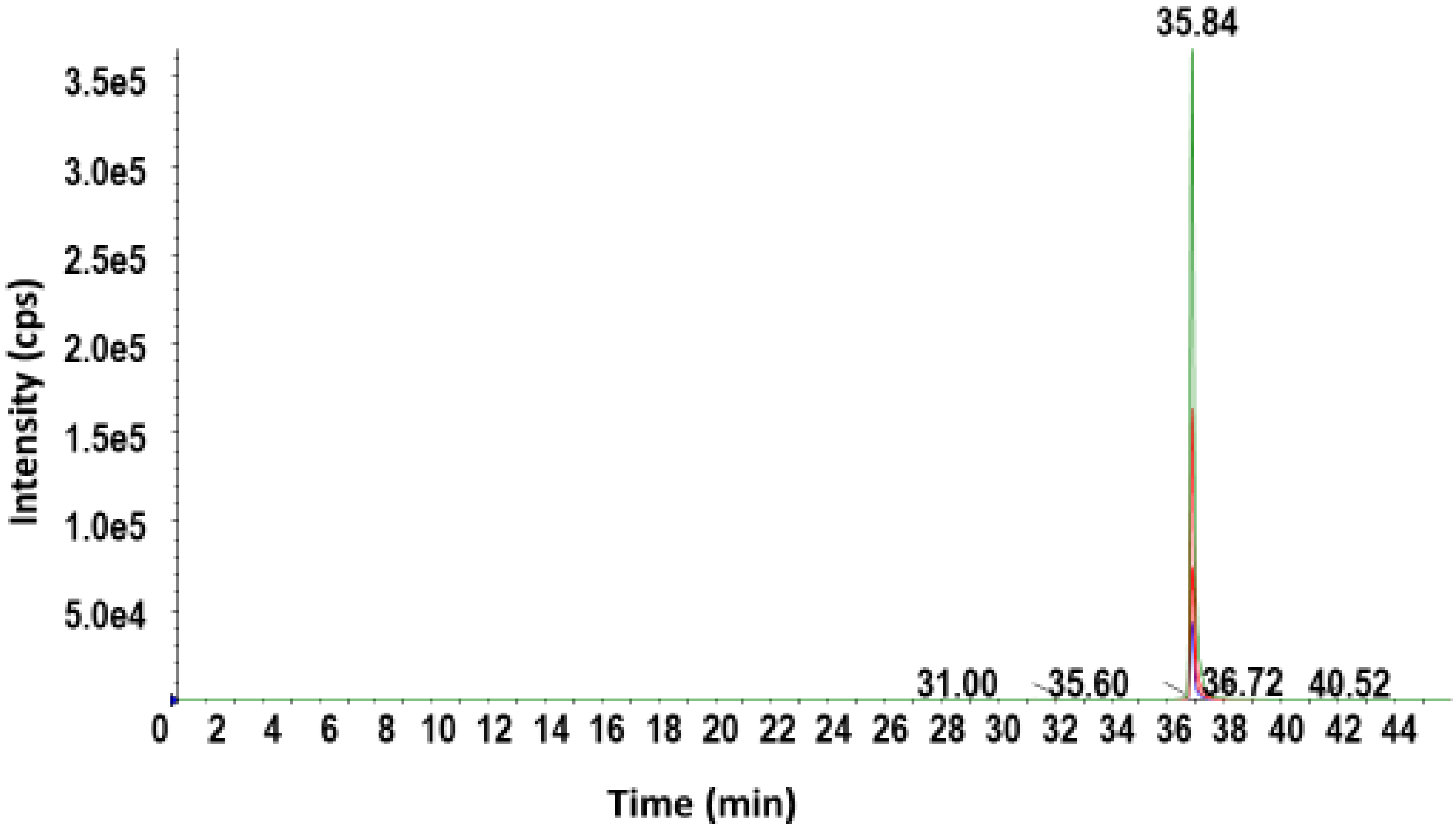

Figure S41. Analytical HPLC chromatogram of purified *D*-**1** to validate purity. Monitored by MS.

Compound 2 (prelomaiviticin)

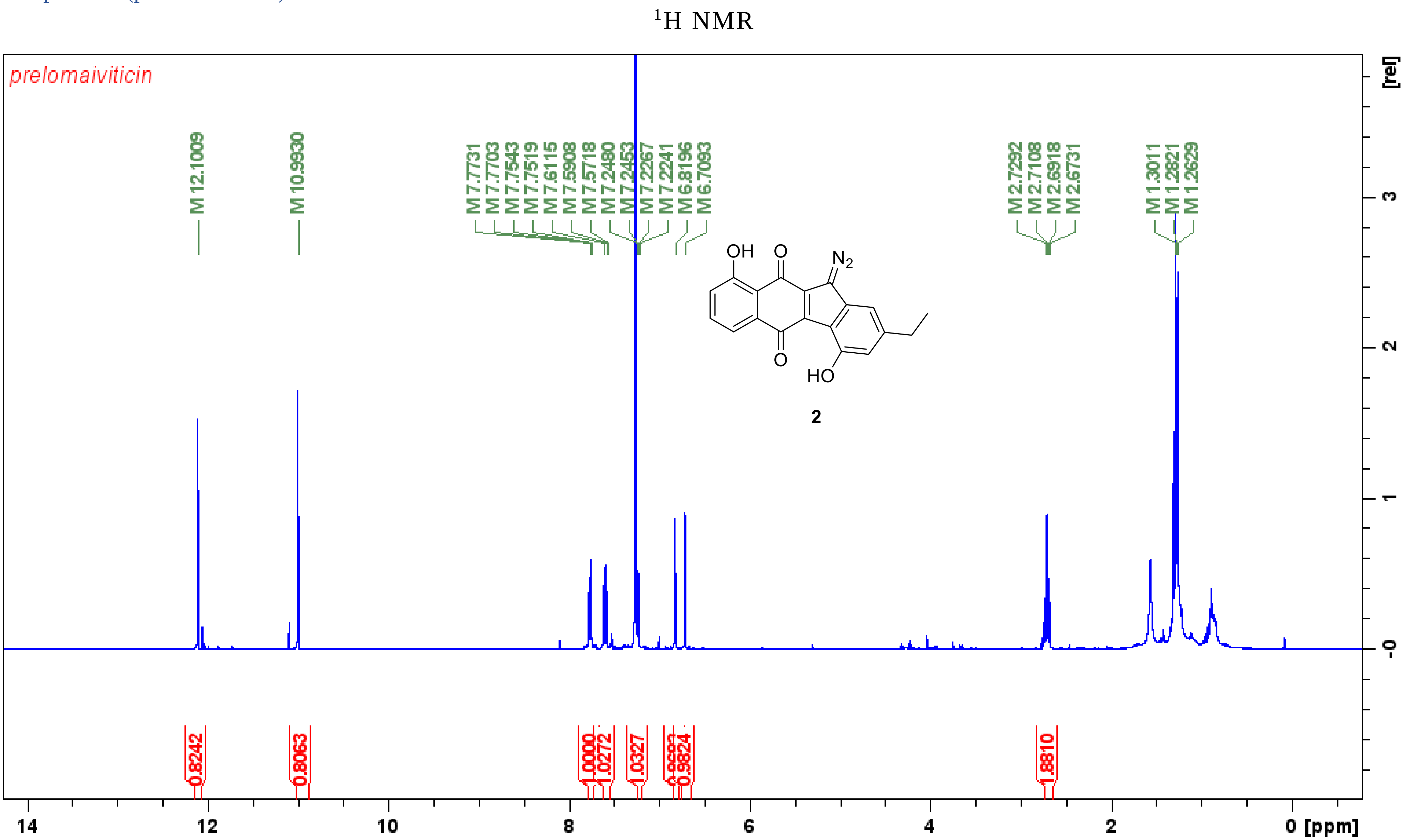

Figure S42. ^{1}H NMR spectrum of compound **2**.

^{1}H NMR

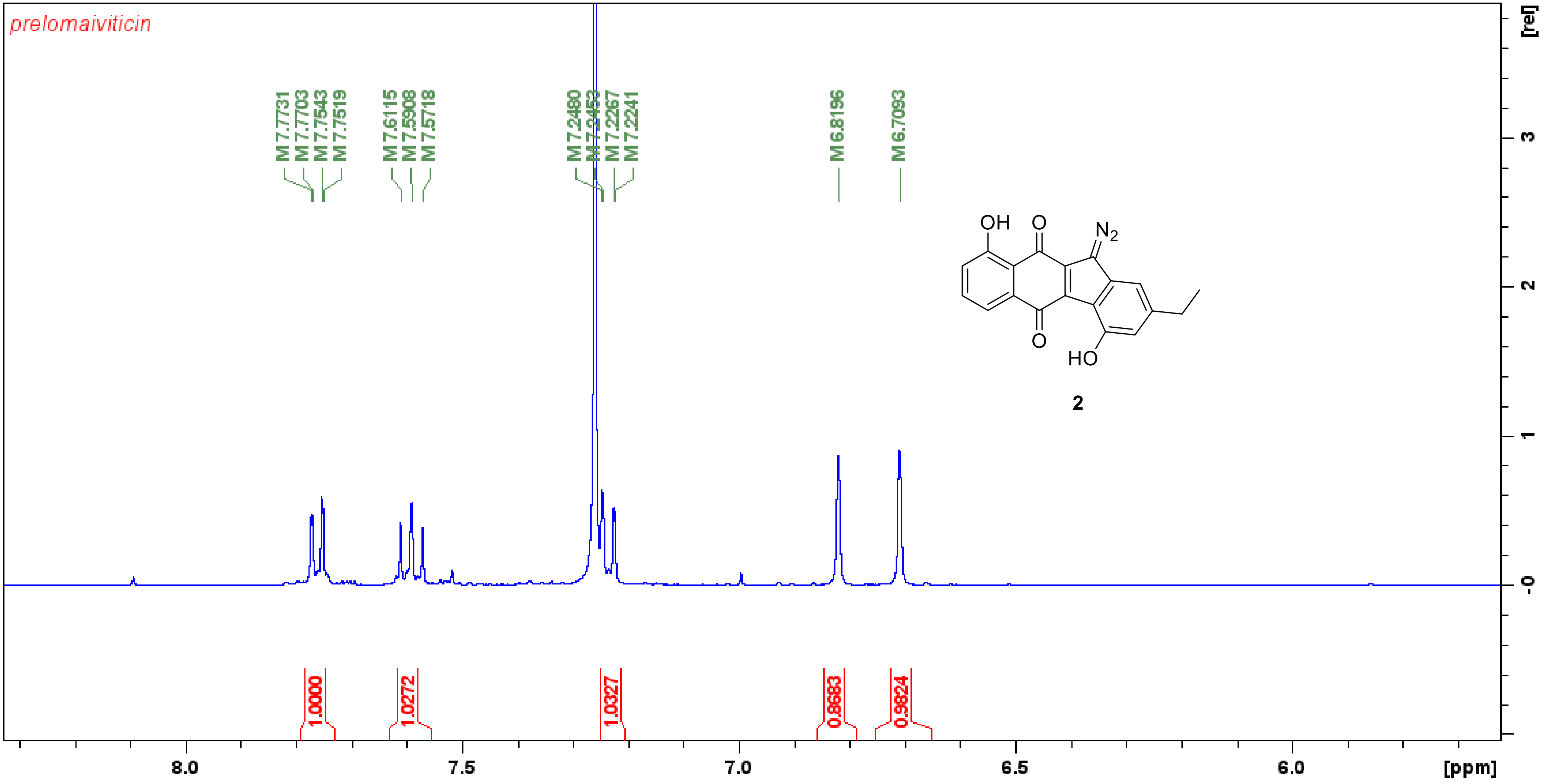

Figure S43. Zoomed in ^{1}H NMR spectrum of compound **2**.

^{13}C NMR

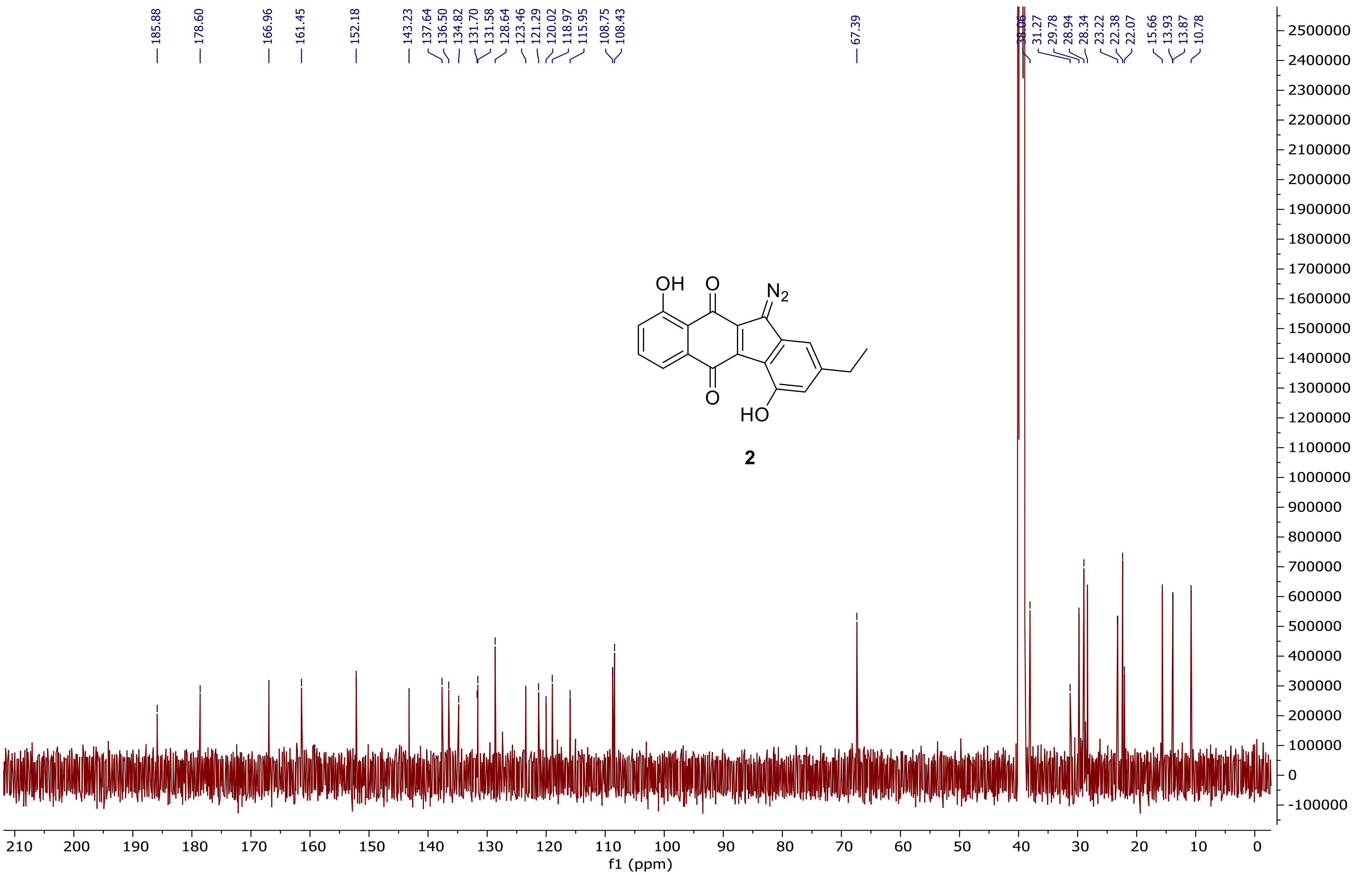

Figure S44. ^{13}C NMR spectrum of compound **2**.

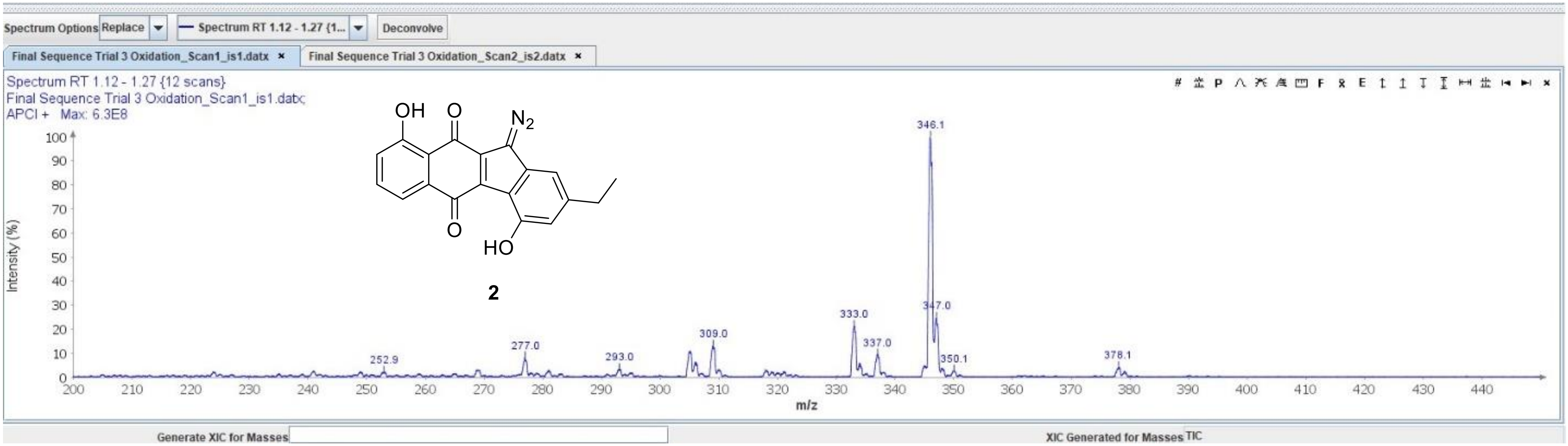

Figure S45. APCI-MS spectrum of compound **2**. Polarity: positive

TLCMS

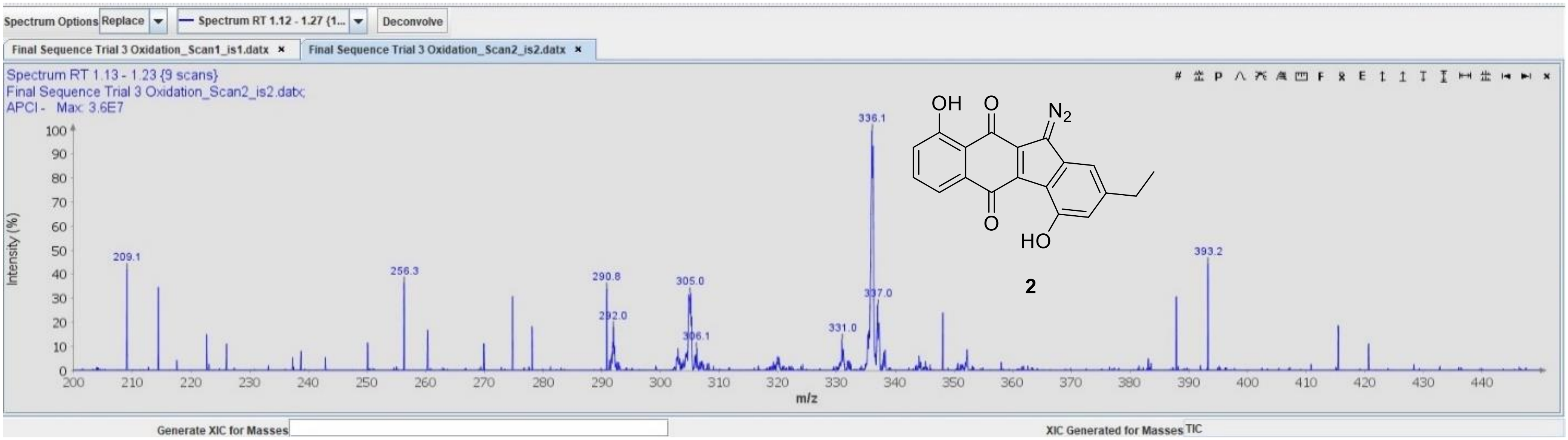

Figure S46. APCI-MS spectrum of compound **2**. Polarity: negative

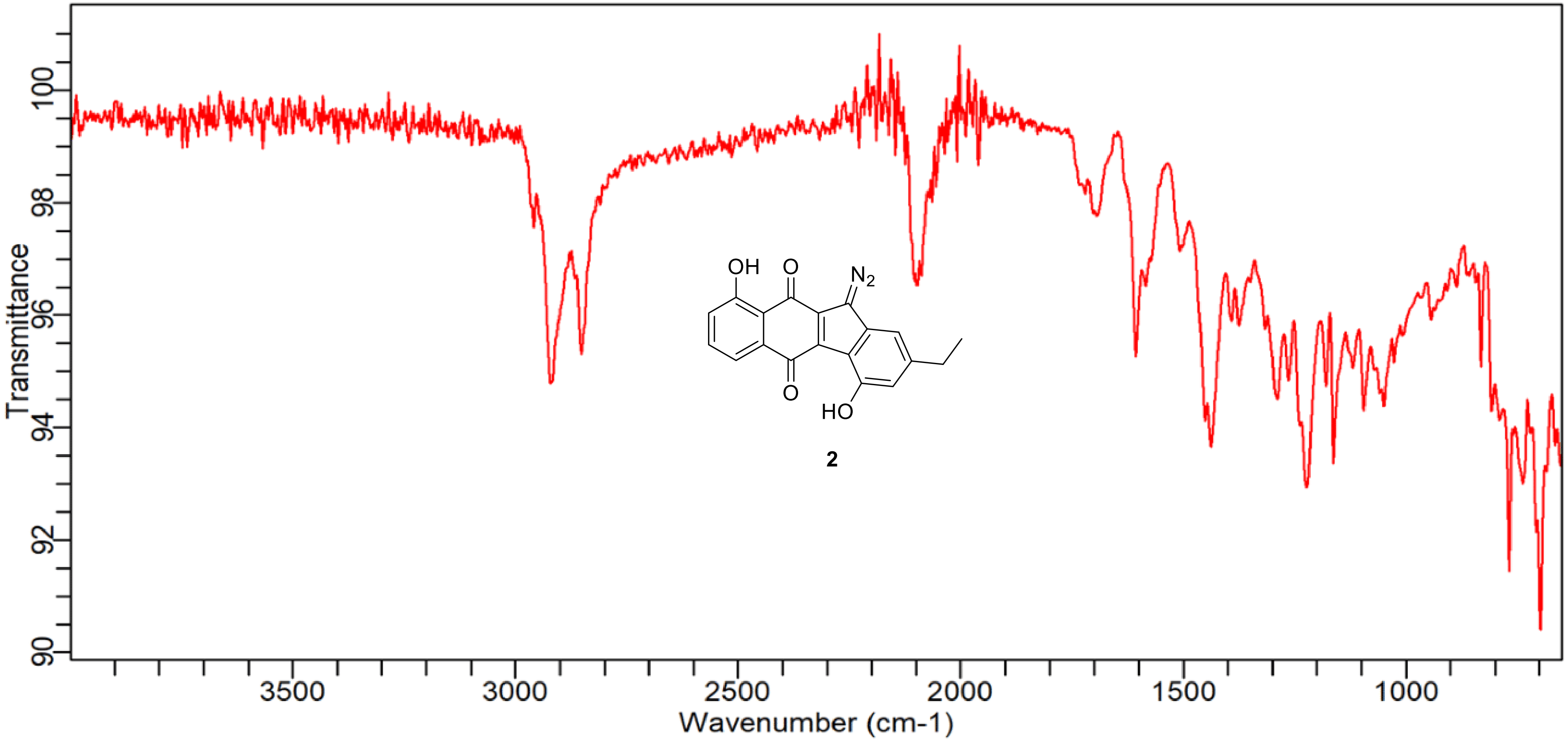

Figure S47. ATR-IR of compound **2**.

Compound 4

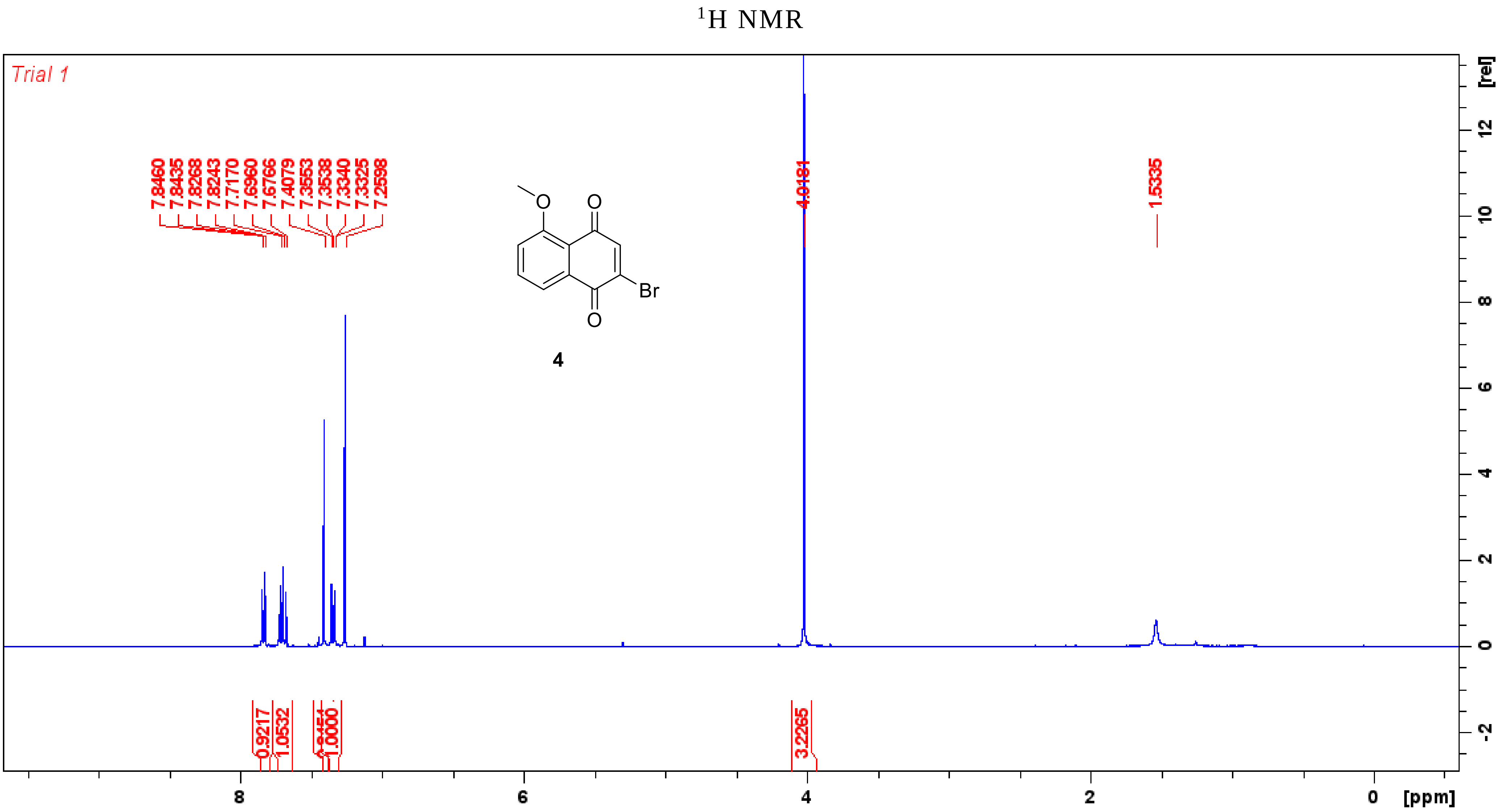

Figure S48. ^{1}H NMR spectrum of compound **4**.

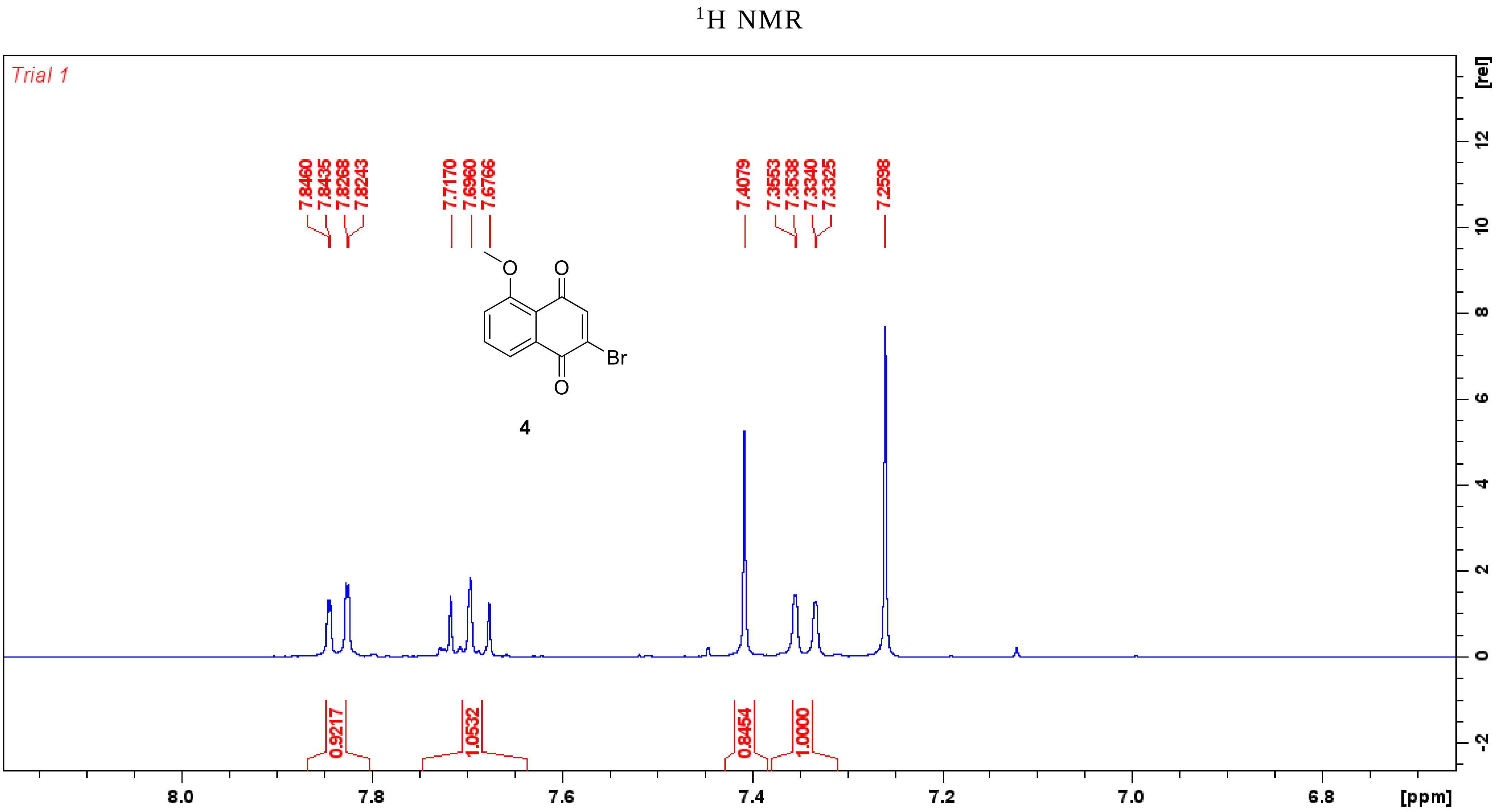

Figure S49. Zoomed in ¹H NMR spectrum of compound **4**.

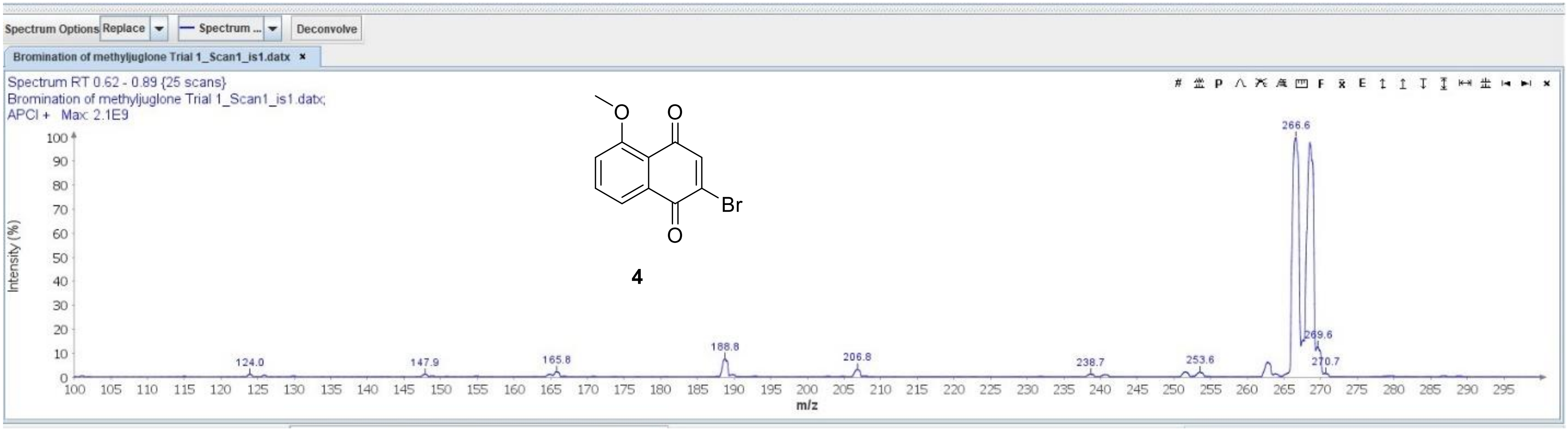

Figure S50. APCI-MS spectrum of compound **4**. Polarity: Positive

Compound 5

^{1}H NMR

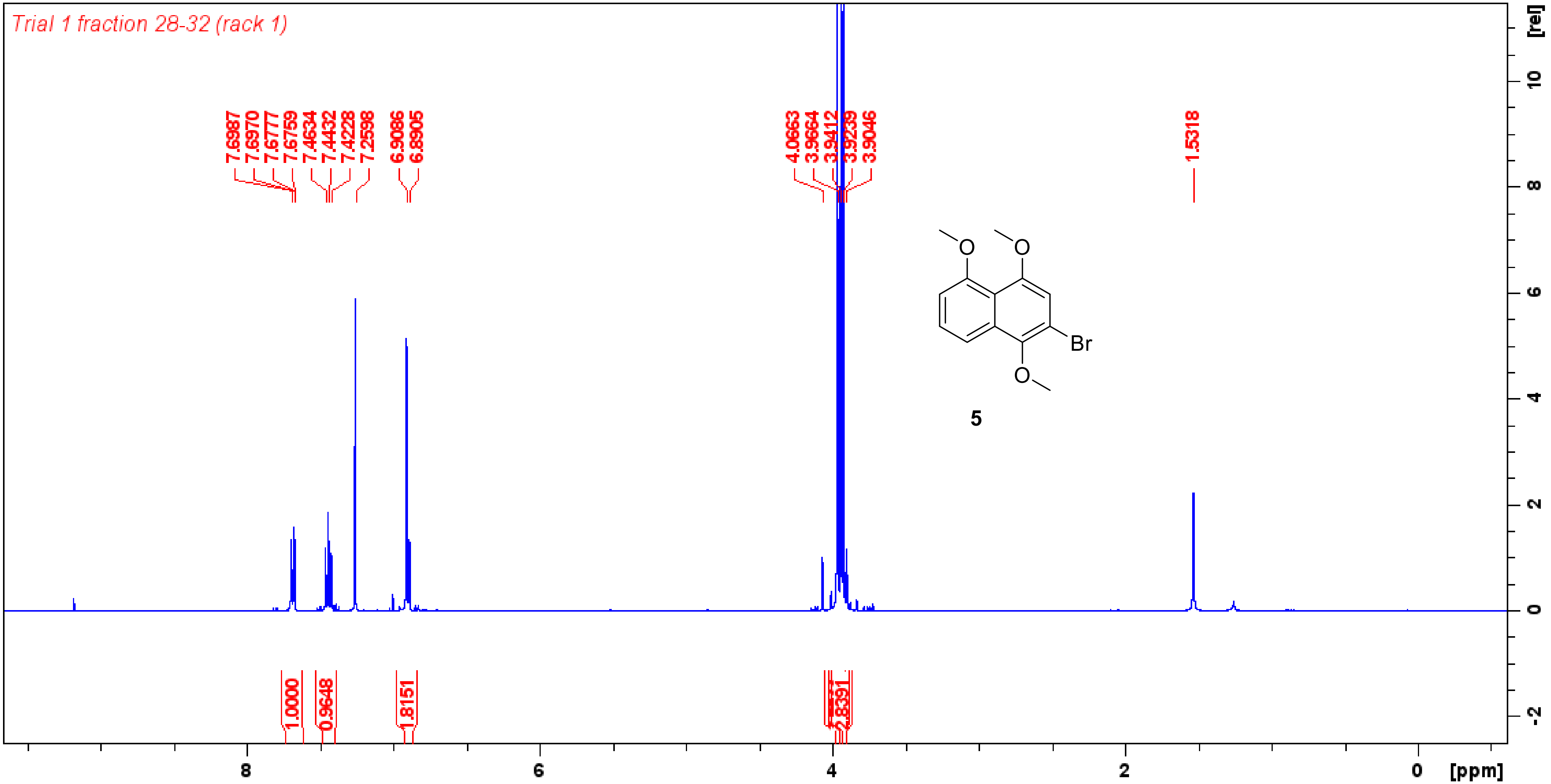

Figure S51. ^{1}H NMR spectrum of compound **5**.

^{1}H NMR

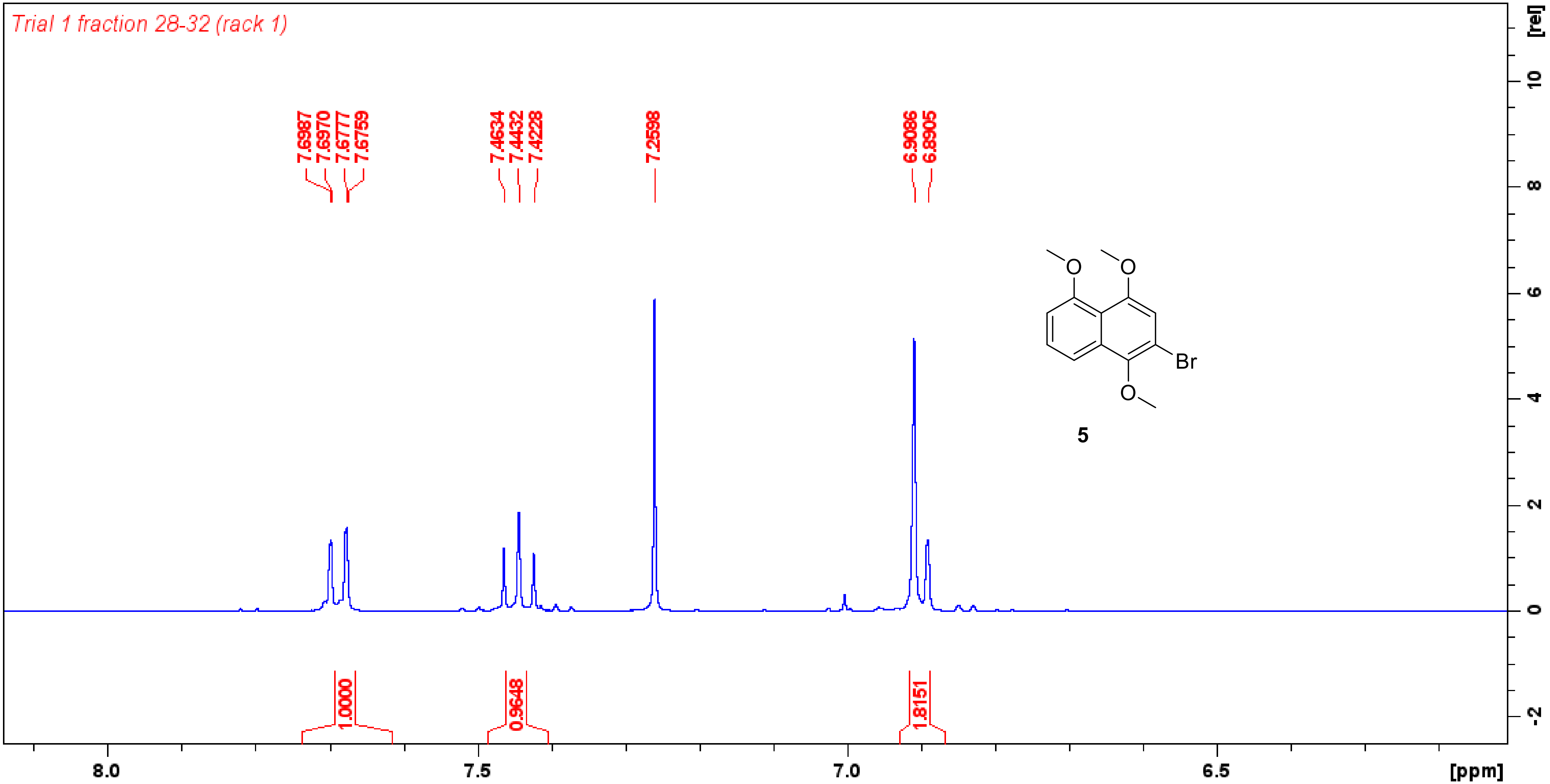

Figure S52. Zoomed in ^{1}H NMR spectrum of compound **5**.

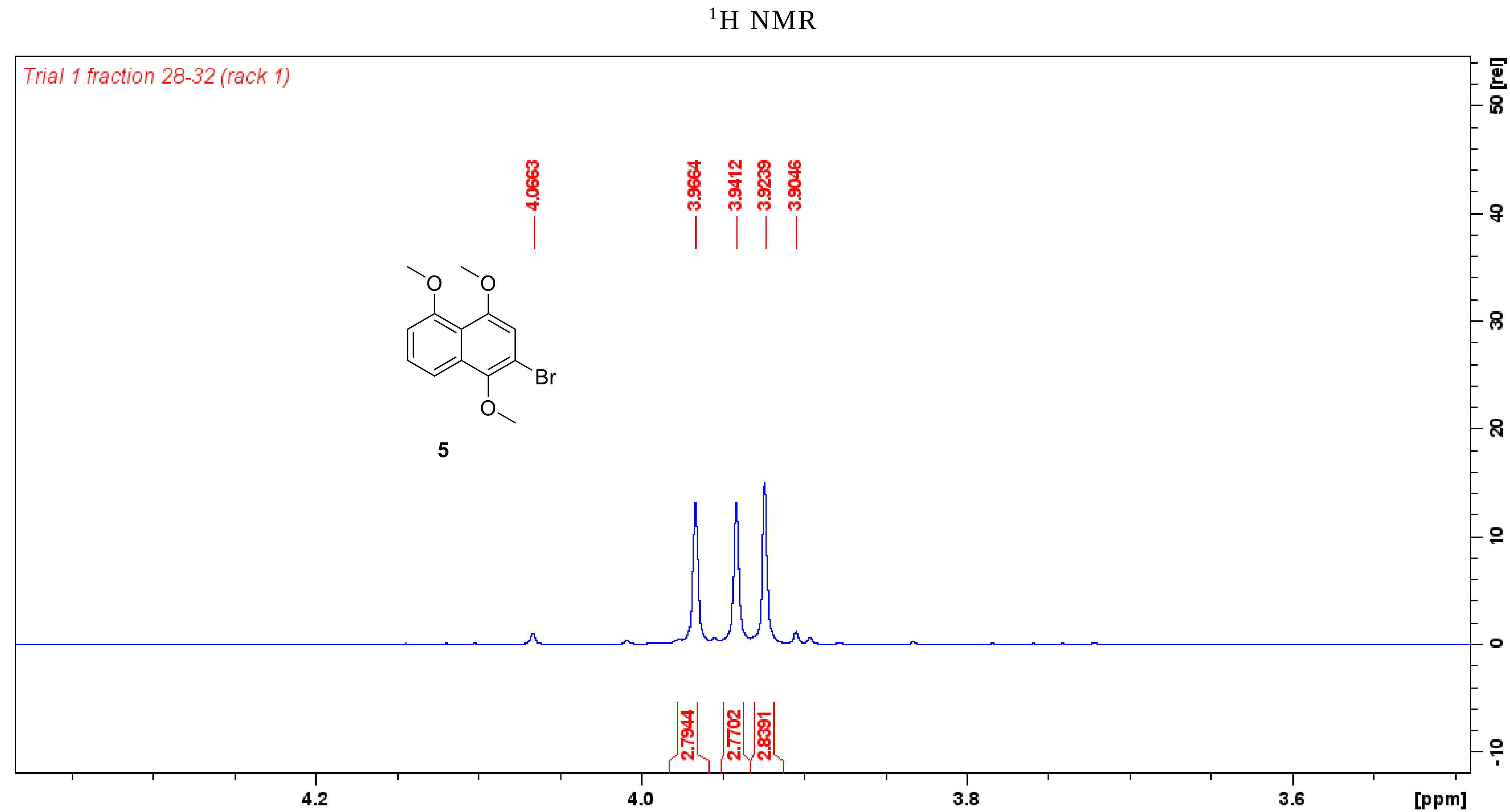

Figure S53. Zoomed in ^{1}H NMR spectrum of compound **5**.

TLCMS

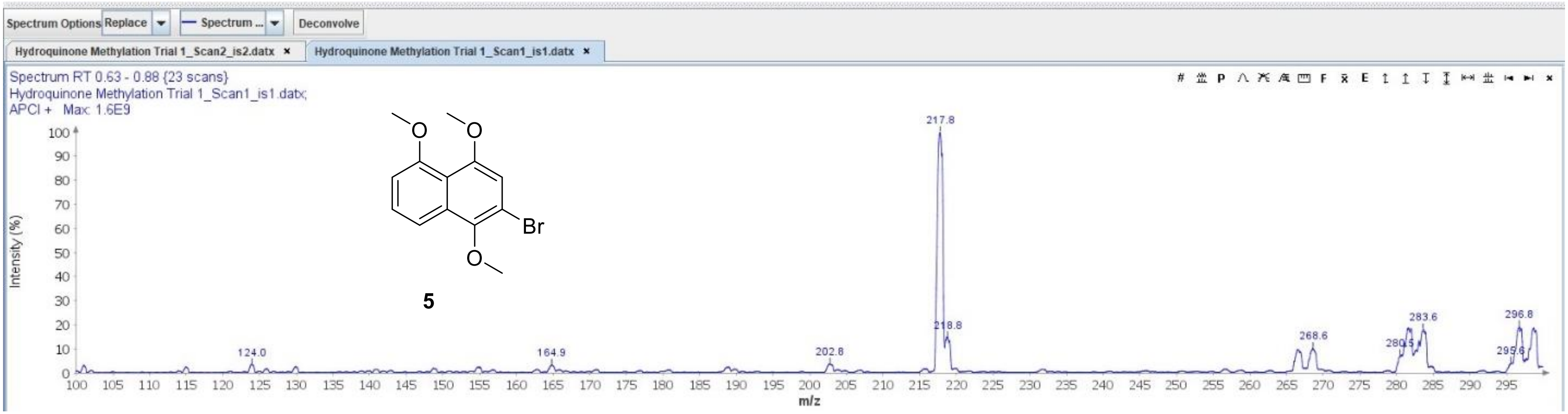

Figure S54. APCI-MS spectrum of compound **5**. Polarity: Positive

Compound 6

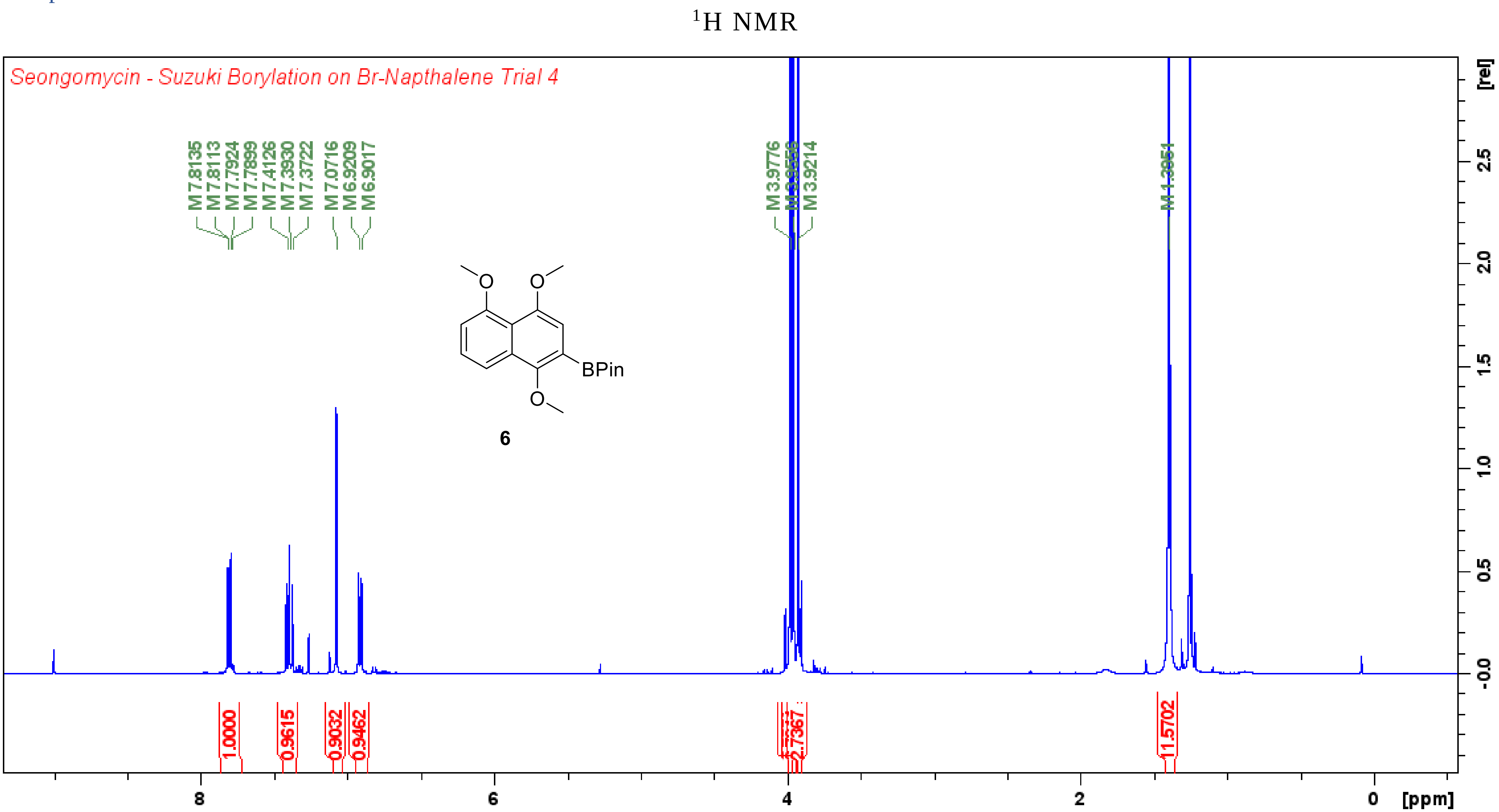

Figure S55. ¹H NMR spectrum of compound **6**.

^{1}H NMR

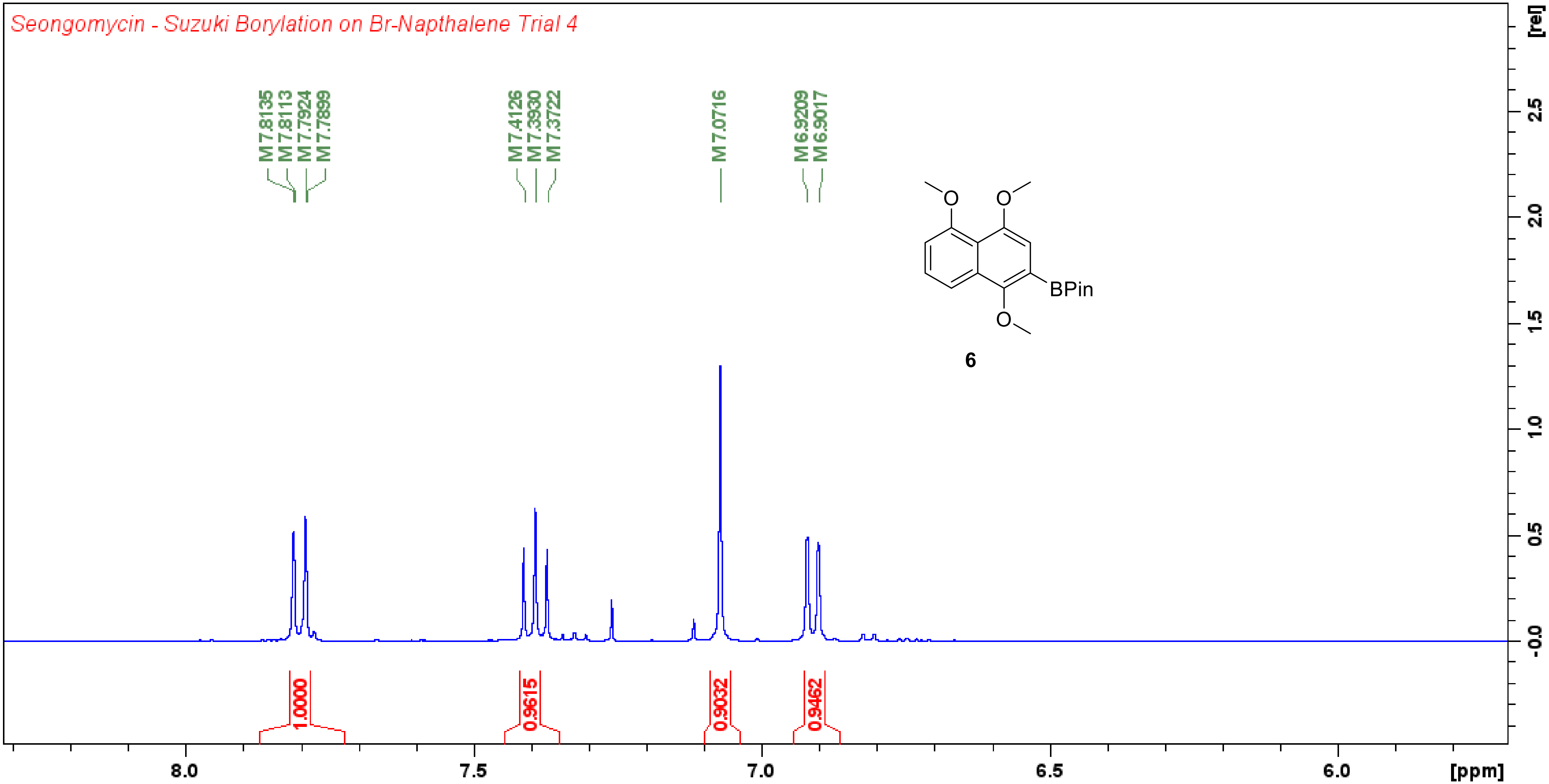

Figure S56. Zoomed in ^{1}H NMR spectrum of compound **6**.

^{1}H NMR

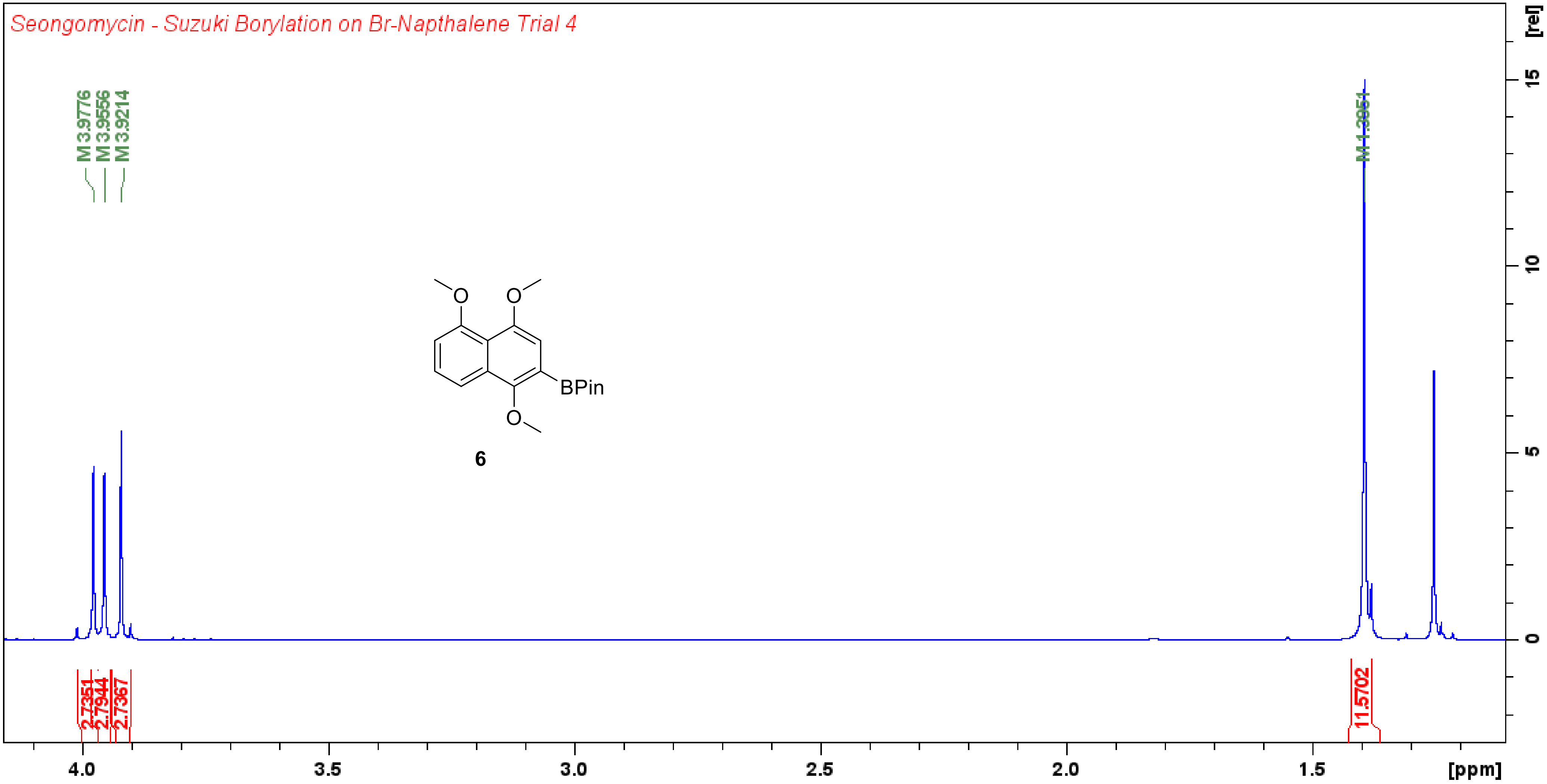

Figure S57. Zoomed in ^{1}H NMR spectrum of compound **6**.

^{13}C NMR

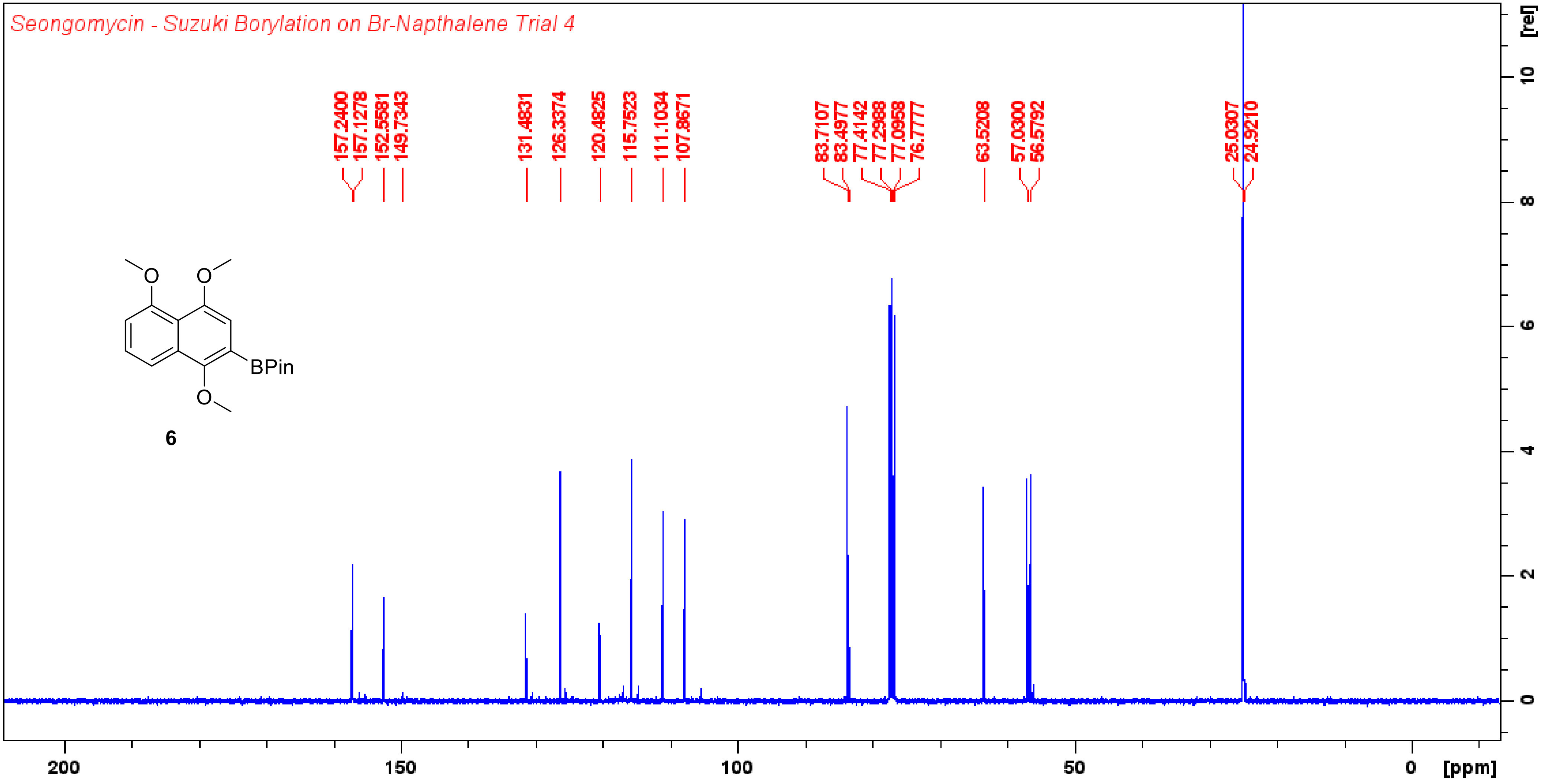

Figure S58. ^{13}C NMR spectrum of compound **6**.

TLCMS

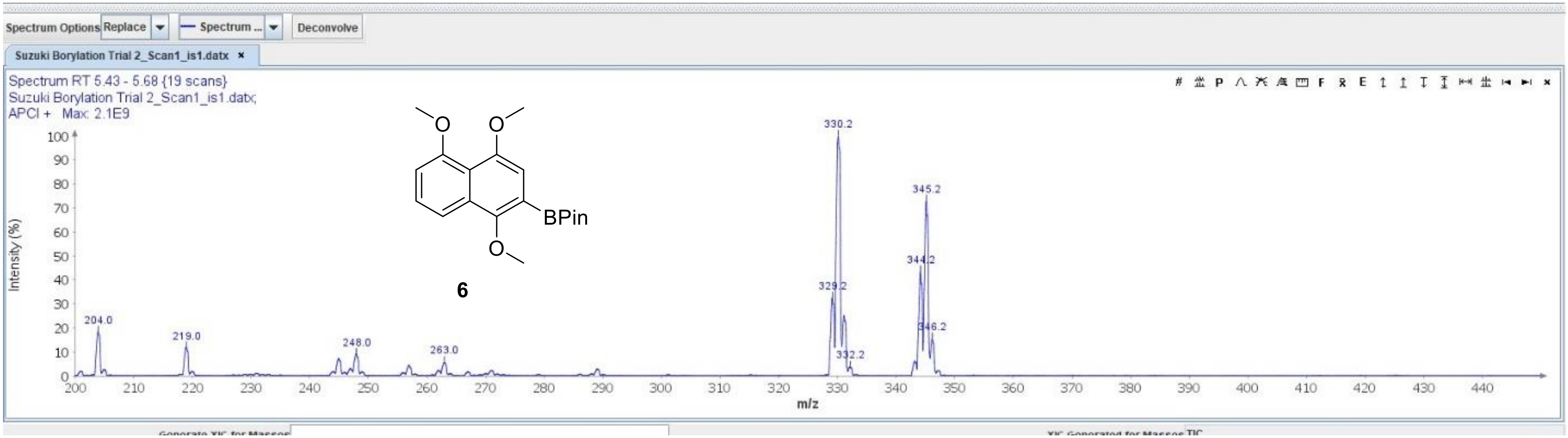

Figure S59. APCI-MS spectrum of compound **6**. Polarity: Positive

Compound 8

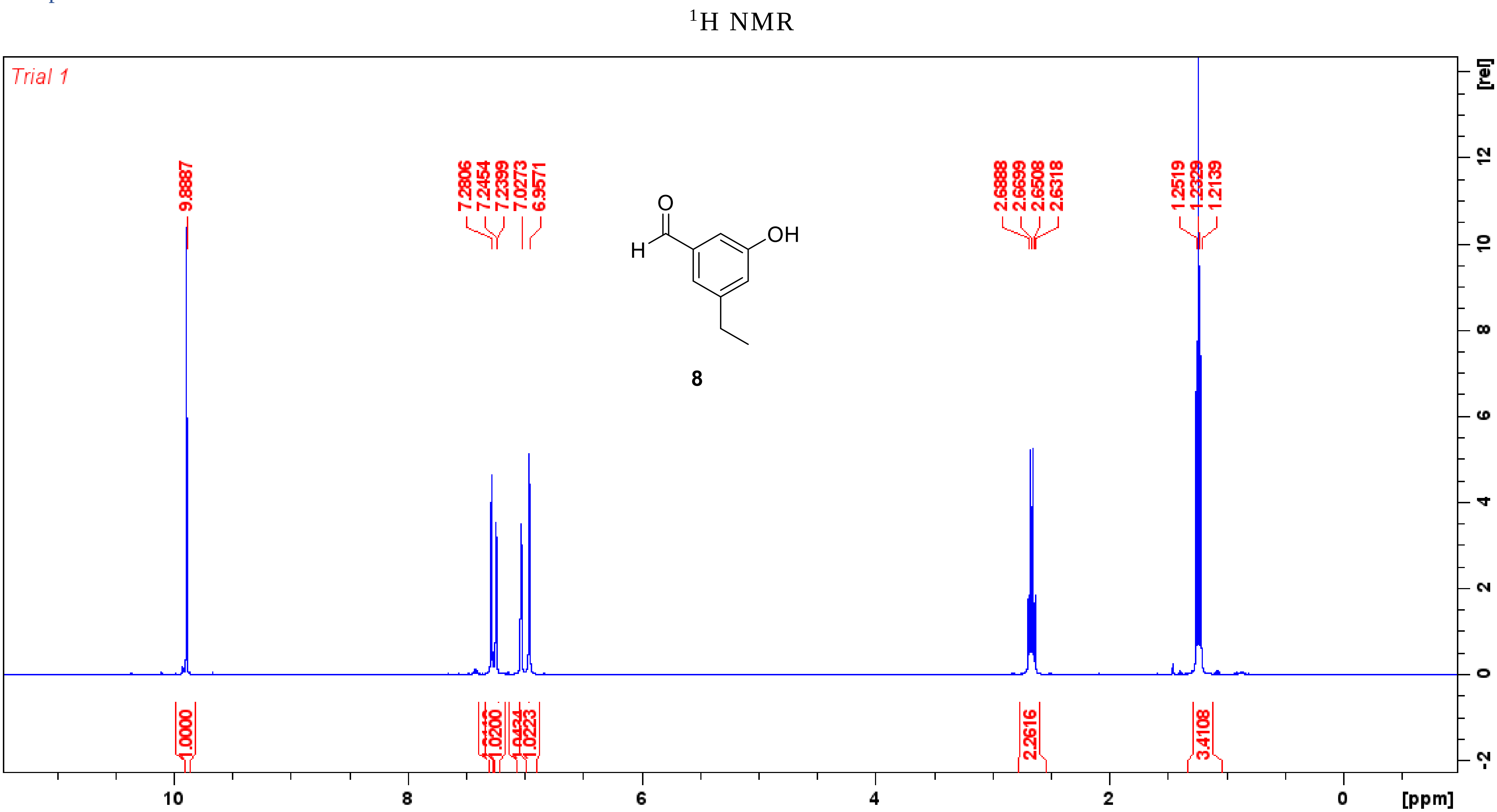

Figure S60. ¹H NMR spectrum of compound **8**.

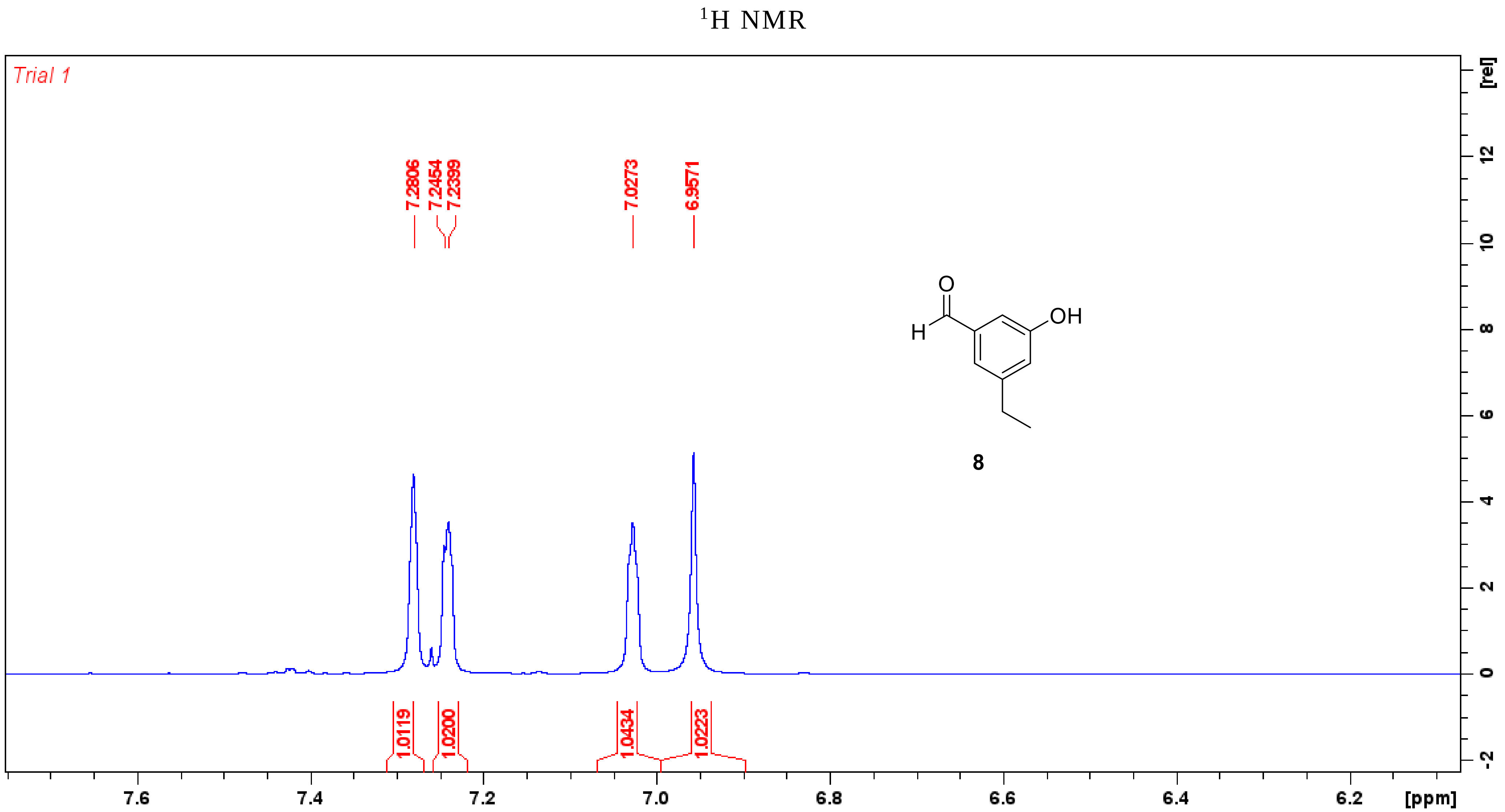

Figure S61. Zoomed in ^{1}H NMR spectrum of compound **8**.

^{13}C NMR

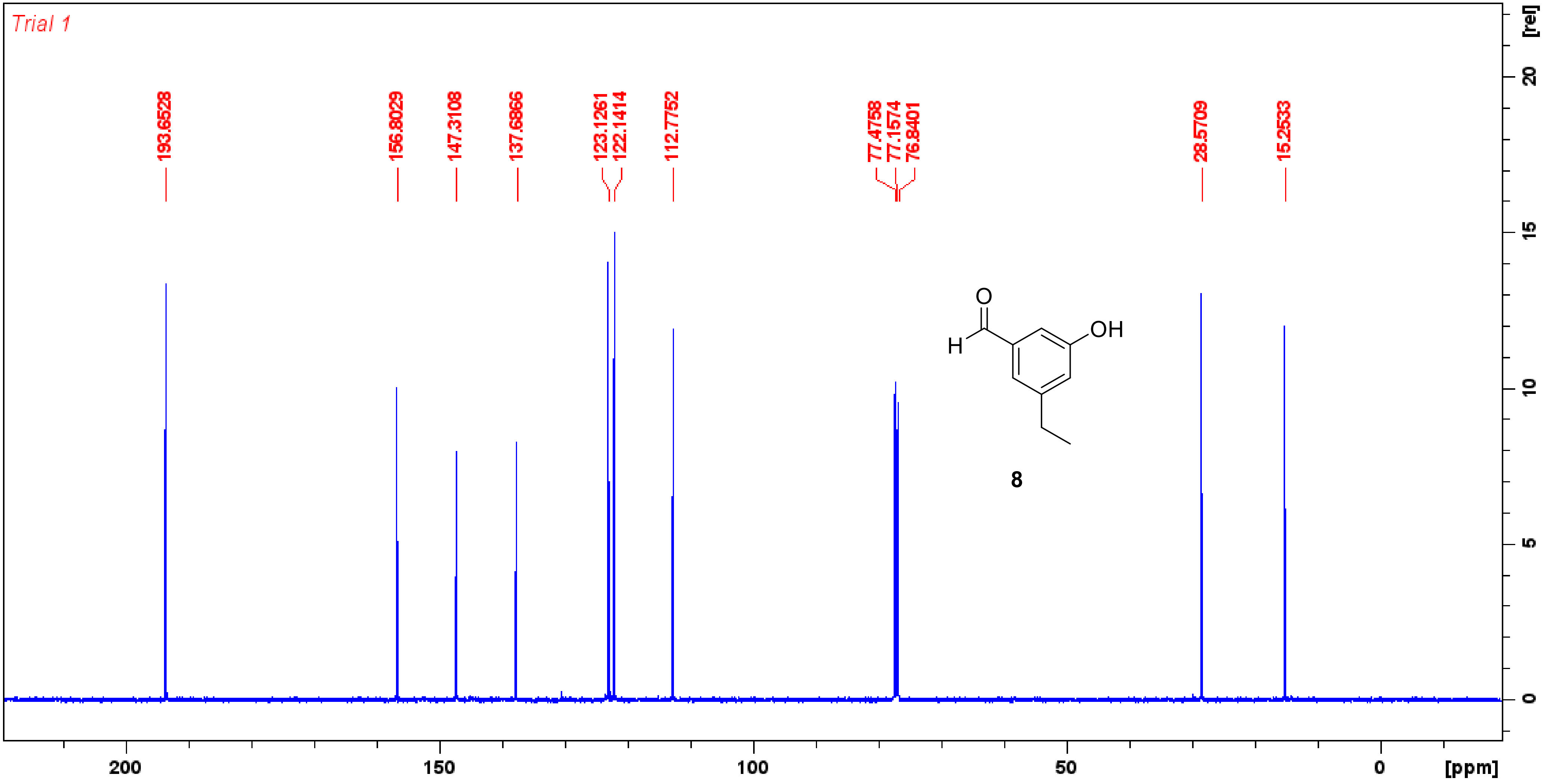

Figure S62. ^{13}C NMR spectrum of compound **8**.

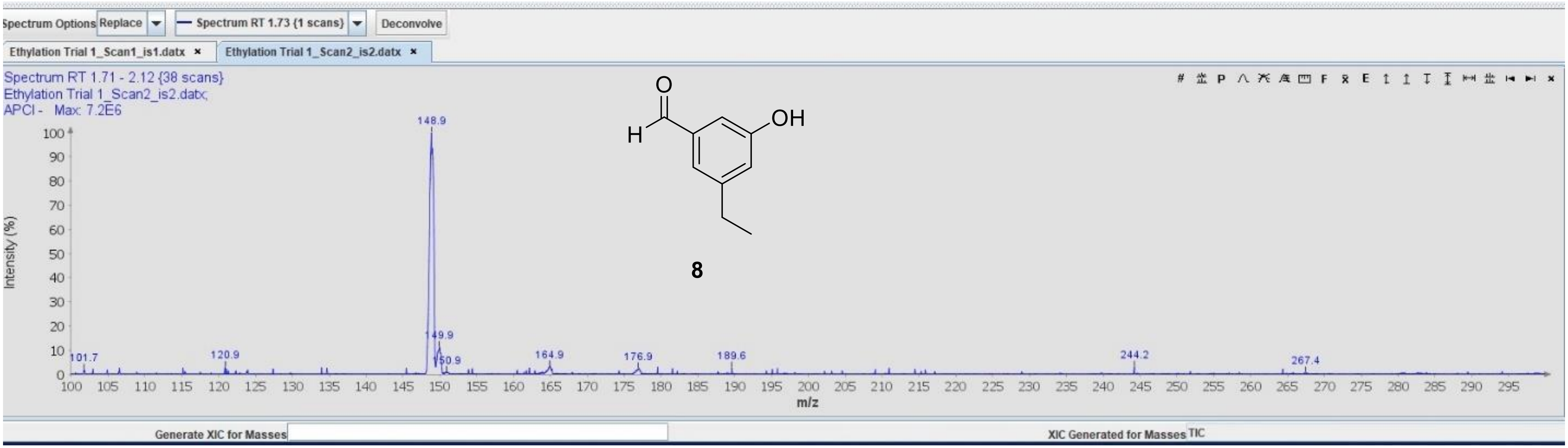

Figure S63. APCI-MS spectrum of compound **8**. Polarity: Negative

Compound 9

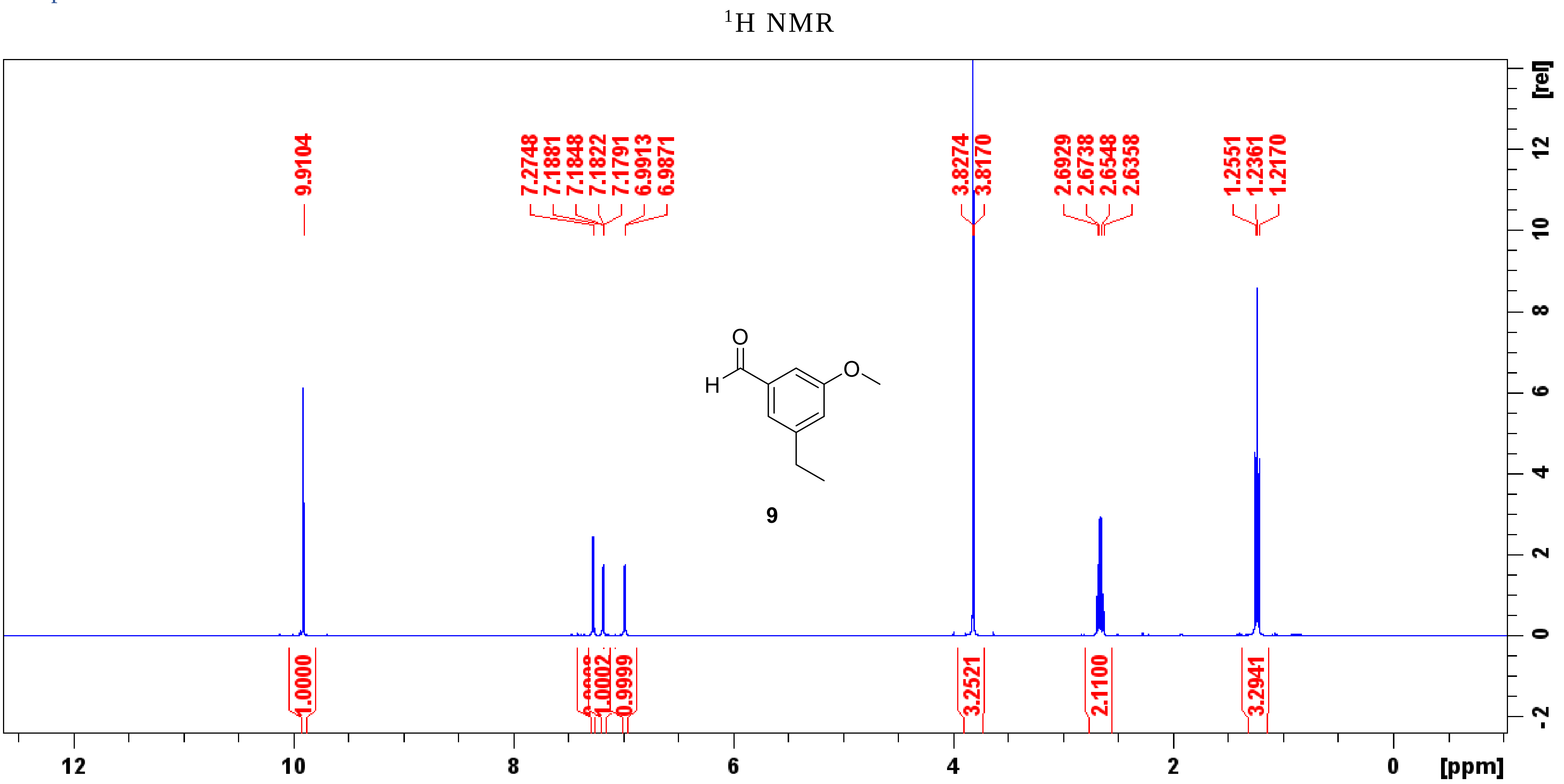

Figure S64. ^{1}H NMR spectrum of compound **9**.

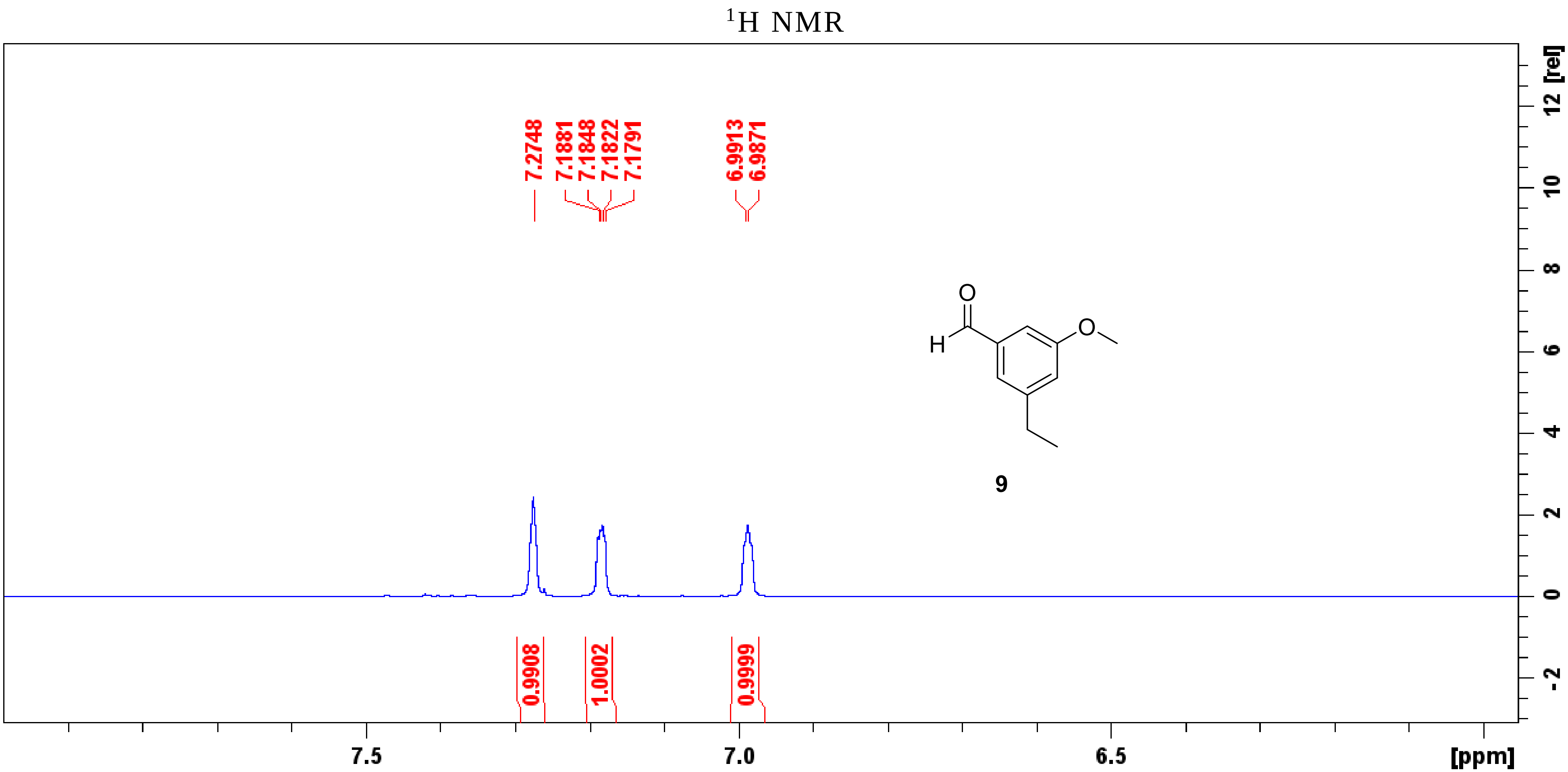

Figure S65. Zoomed in ^{1}H NMR spectrum of compound **9**.

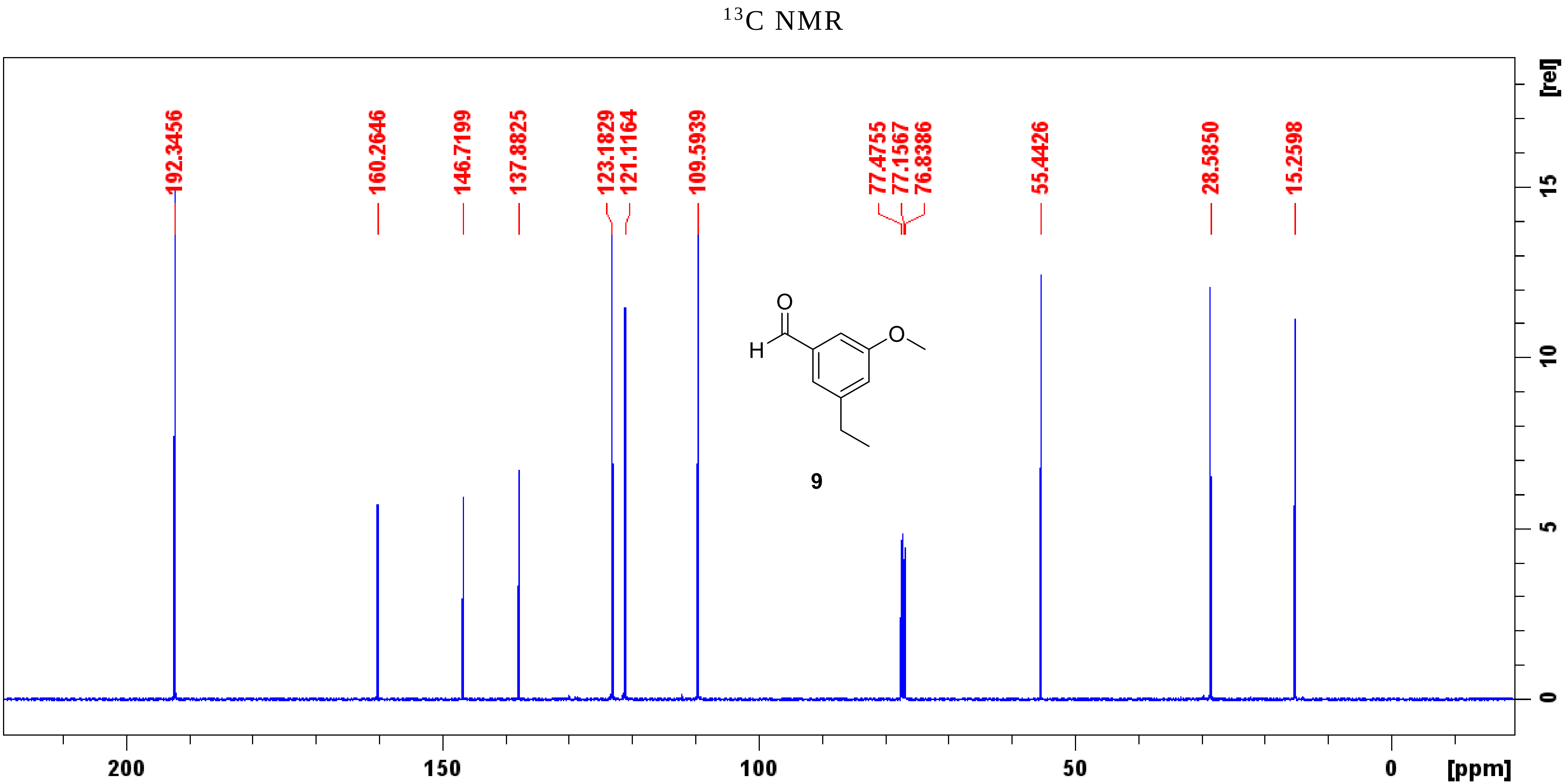

Figure S66. ^{13}C NMR spectrum of compound **9**.

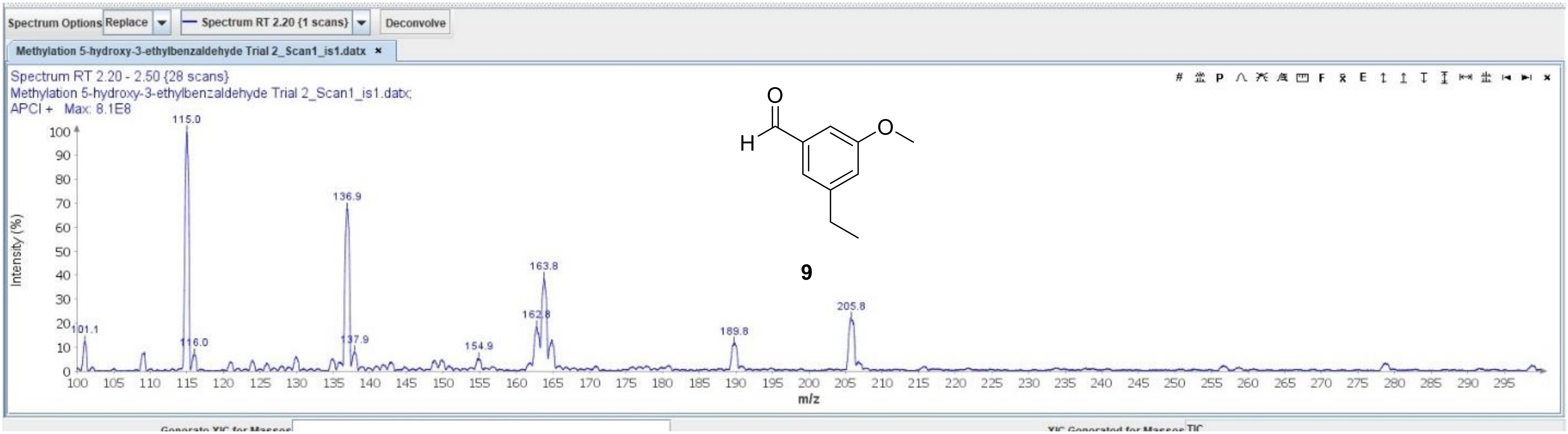

Figure S67. APCI-MS spectrum of compound **9**. Polarity: Positive

Compound 10

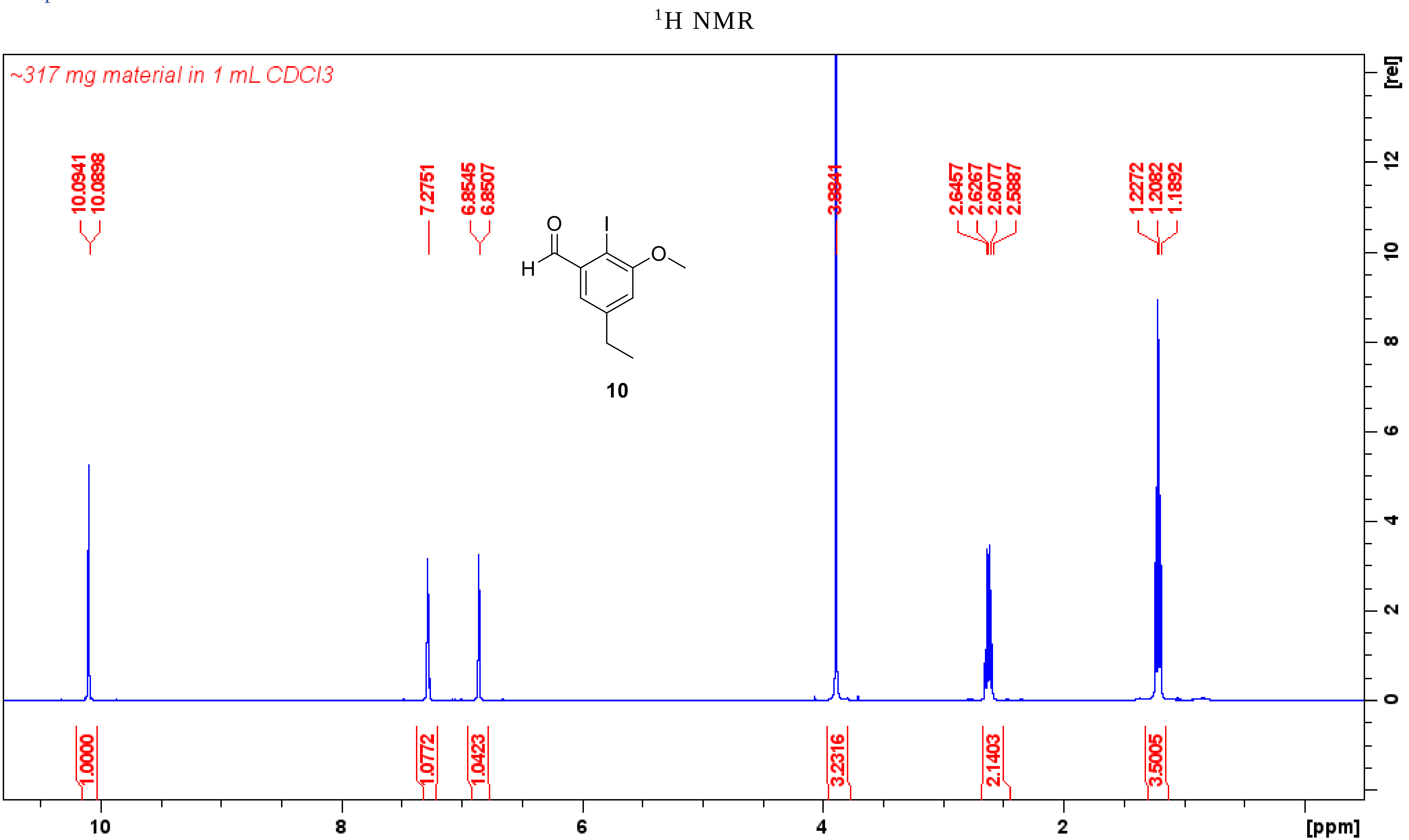

Figure S68. ^{1}H NMR spectrum of compound **10**.

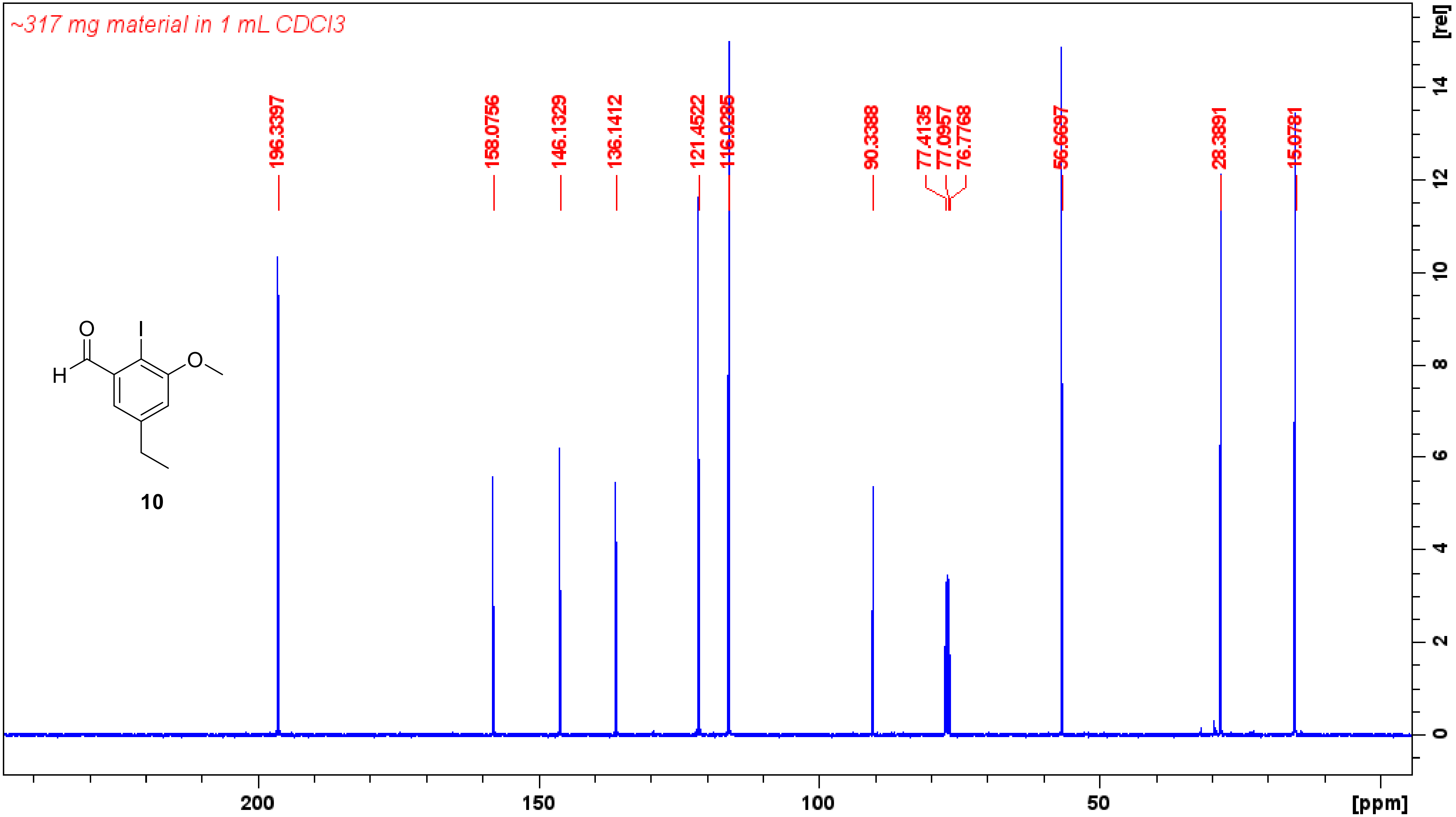

Figure S69. ^{13}C NMR spectrum of compound **10**.

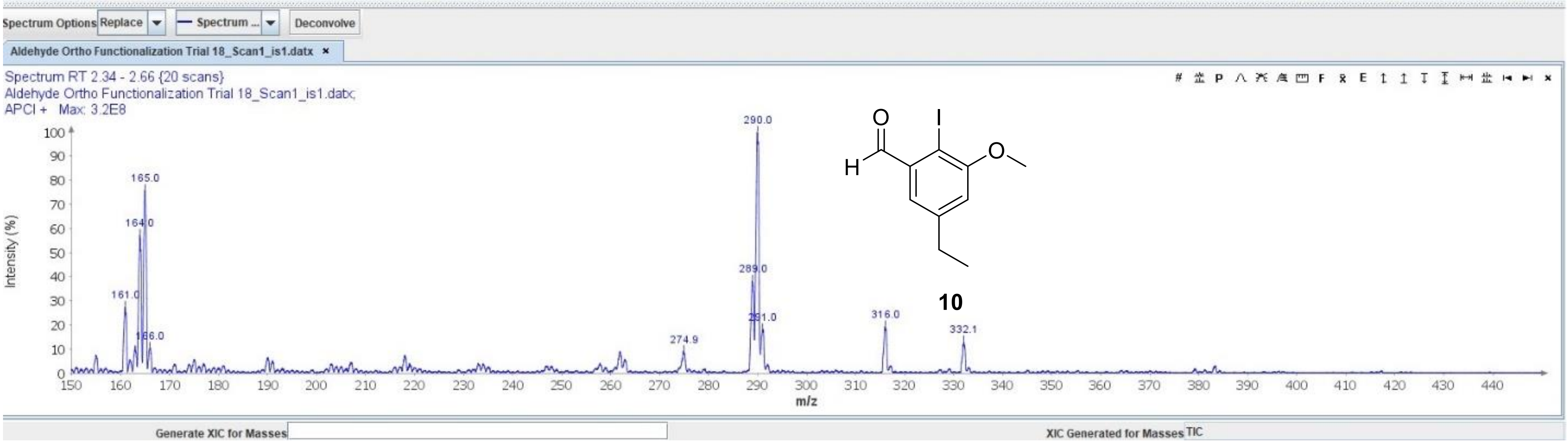

Figure S70. APCI-MS spectrum of compound **10**. Polarity: Positive

2D ^{13}C-^{13}C CORRELATION (INADEQUATE)

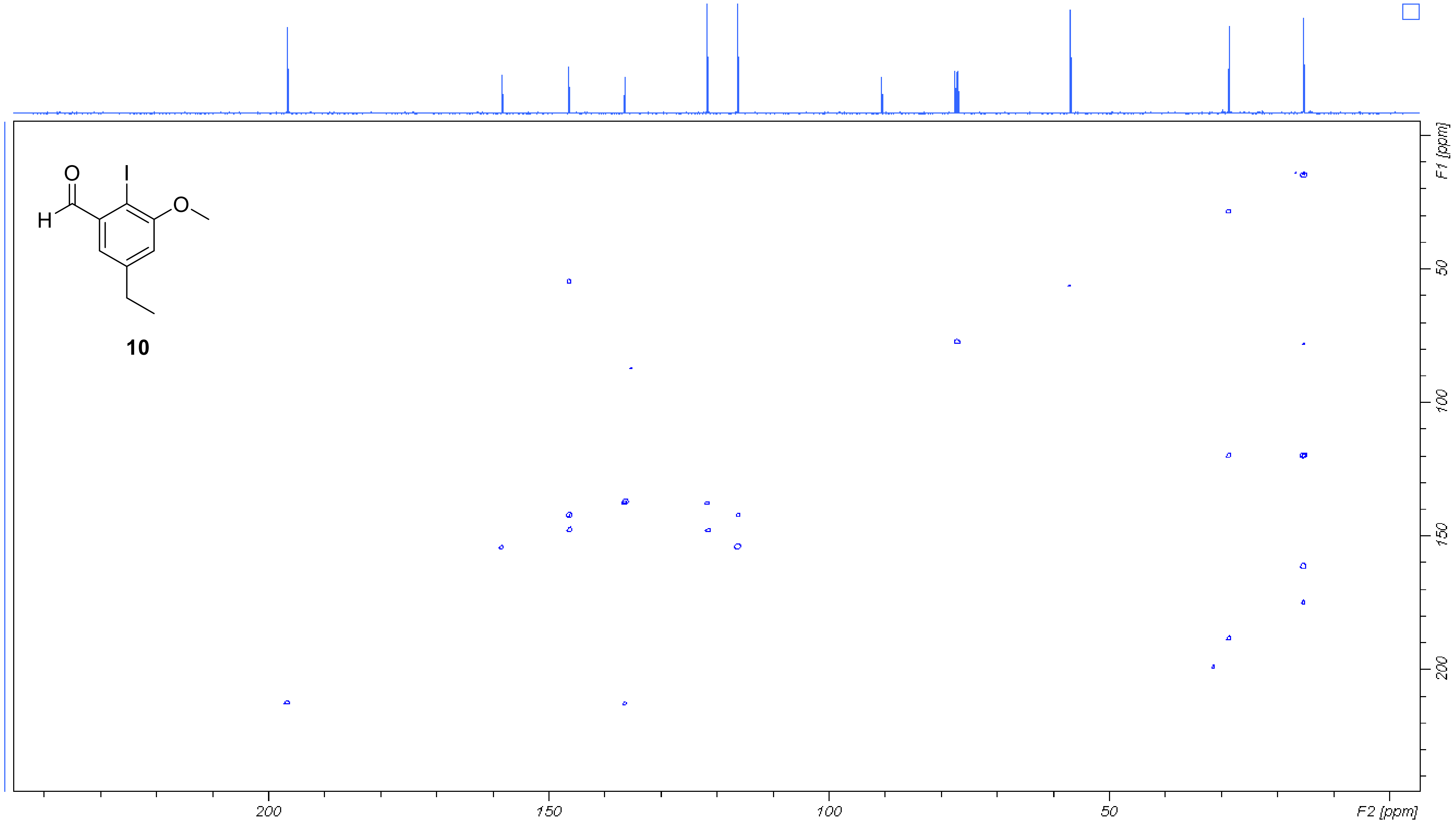

Figure S71. INADEQUATE spectrum of compound **10**.

2D ^{13}C-^{13}C CORRELATION (INADEQUATE)

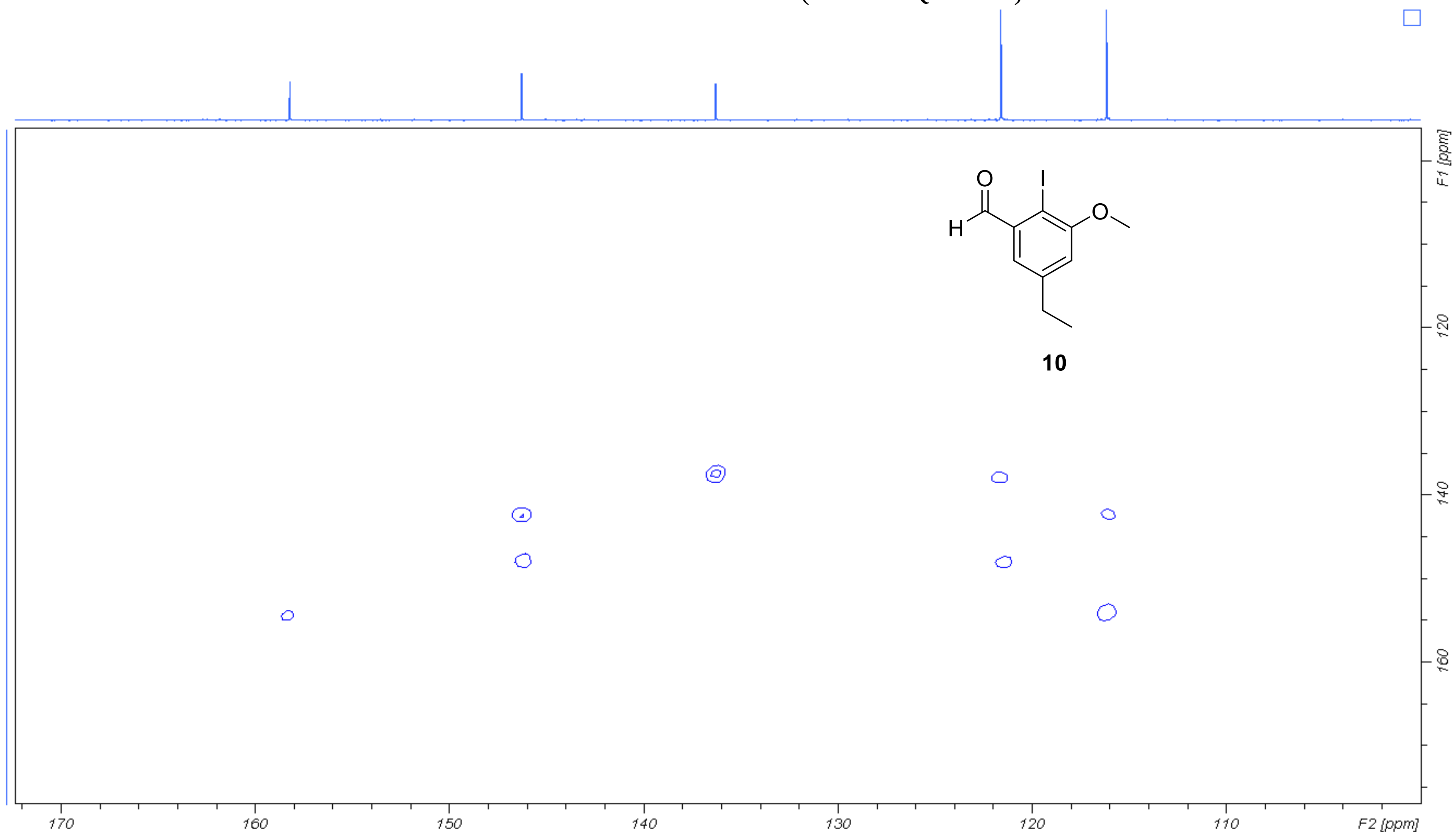

Figure S72. Zoomed in INADEQUATE spectrum of compound **10**.

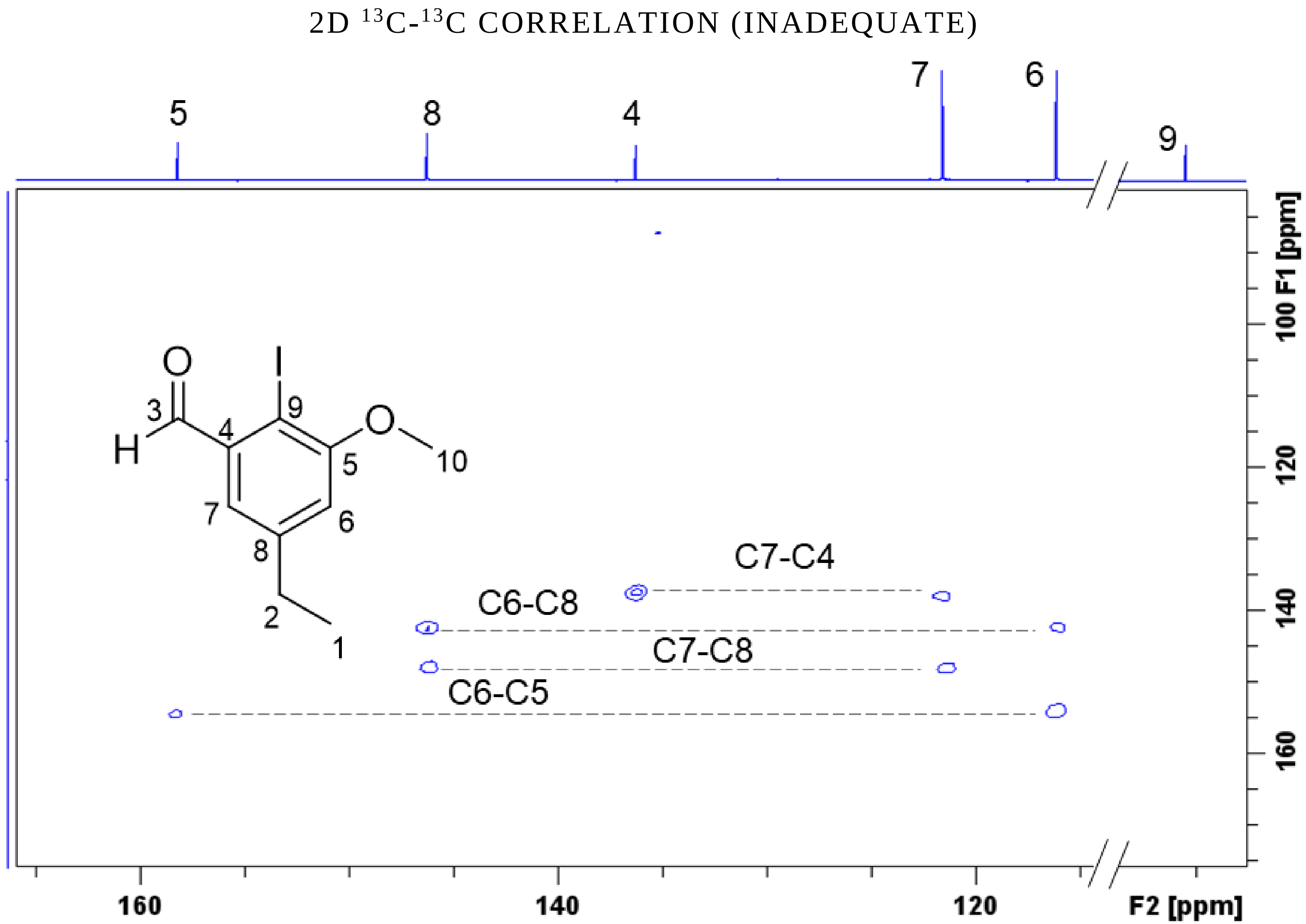

Figure S73. Zoomed in INADEQUATE spectrum of compound **10**.

Compound 11

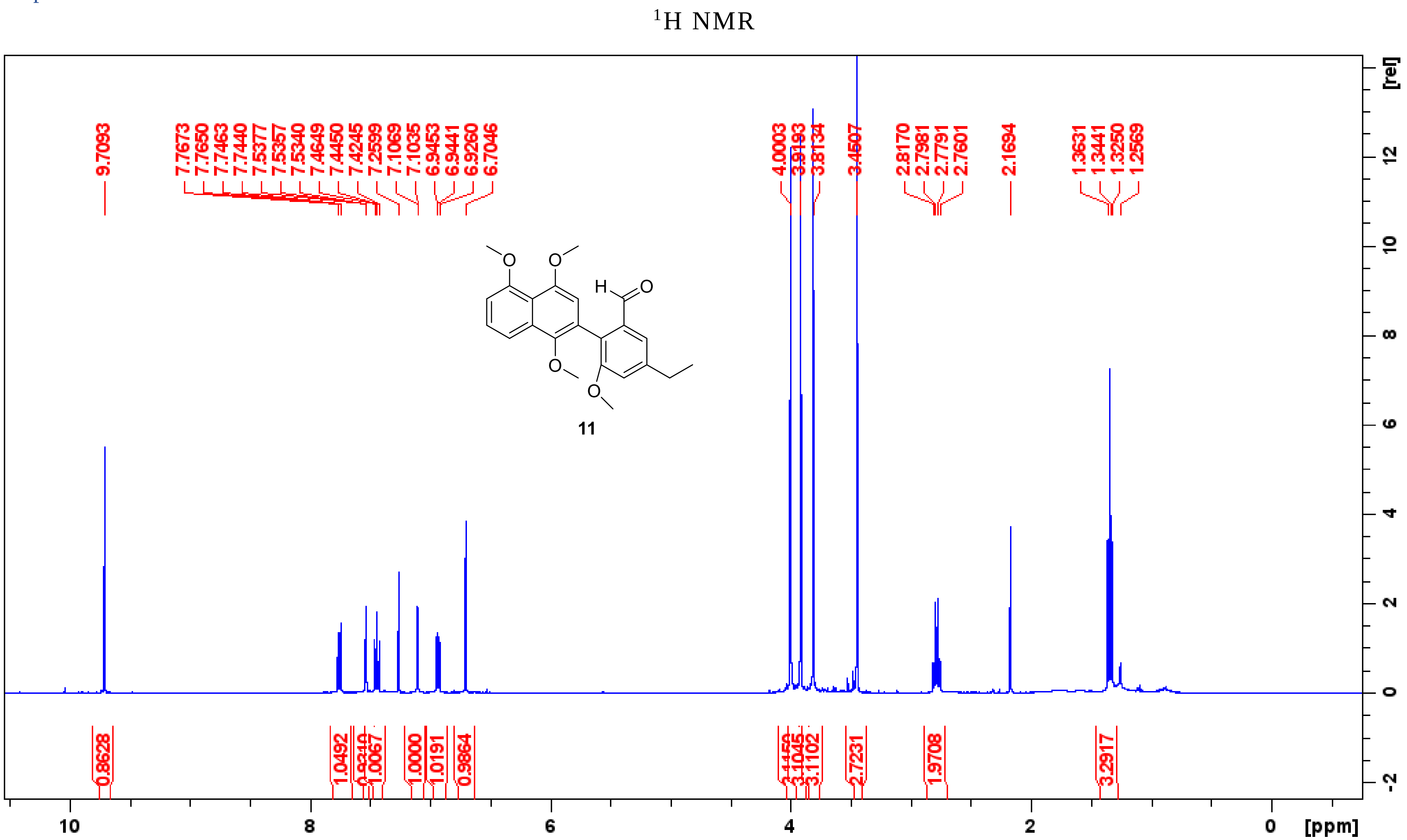

Figure S74. ^{1}H NMR spectrum of compound **11**.

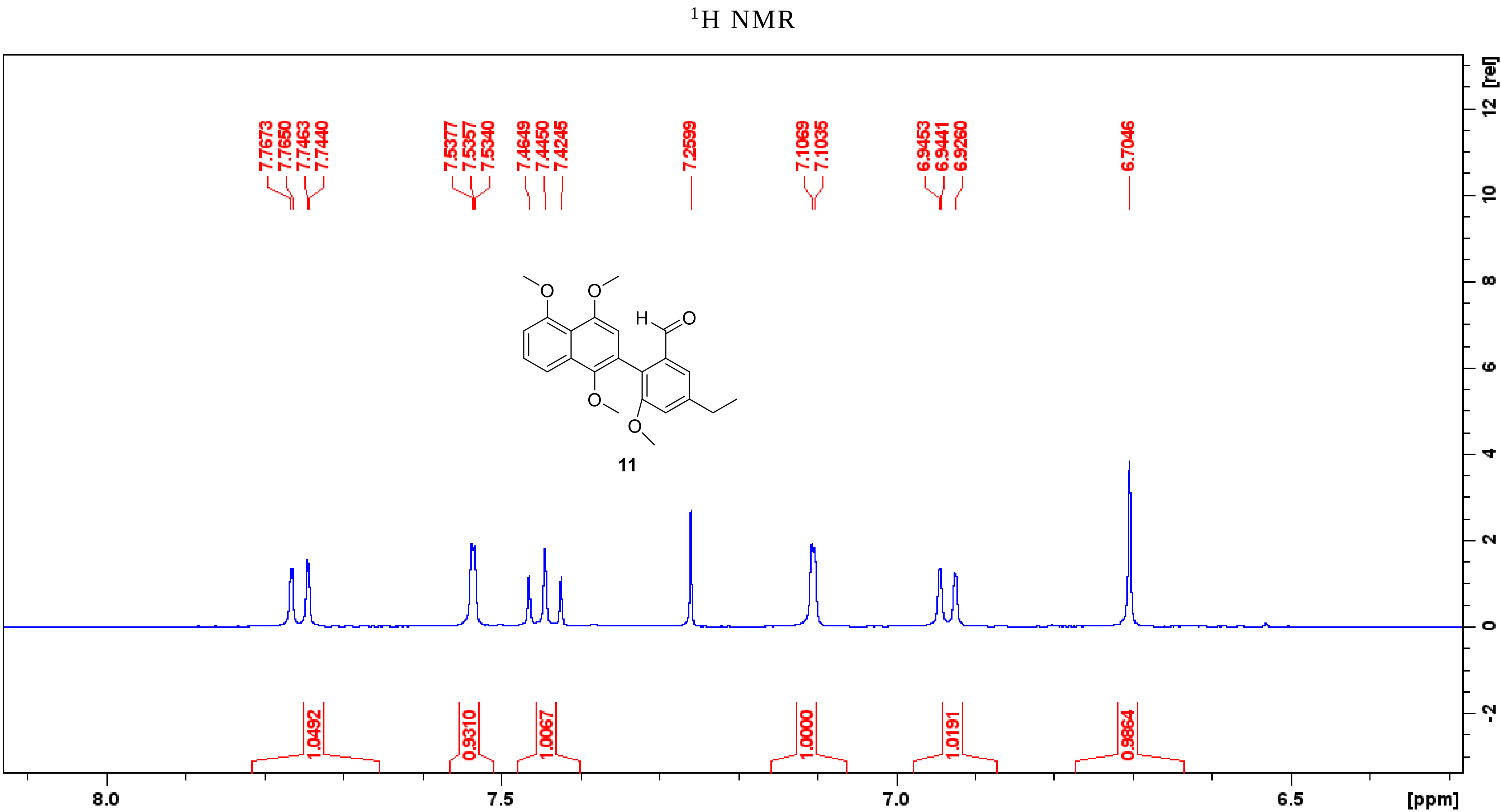

Figure S75. Zoomed in ¹H NMR spectrum of compound **11**.

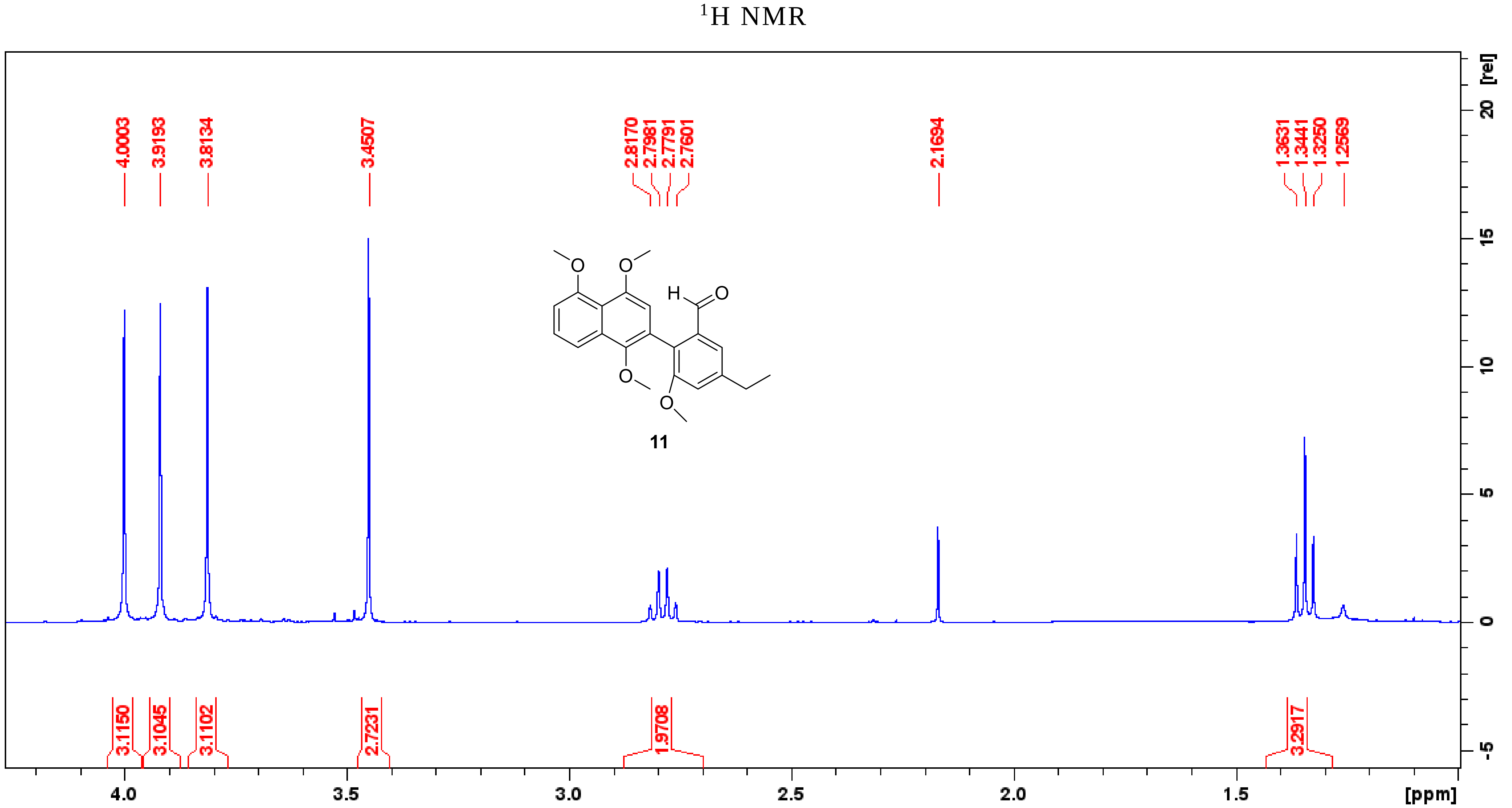

Figure S76. Zoomed in ^{1}H NMR spectrum of compound **11**.

^{13}C NMR

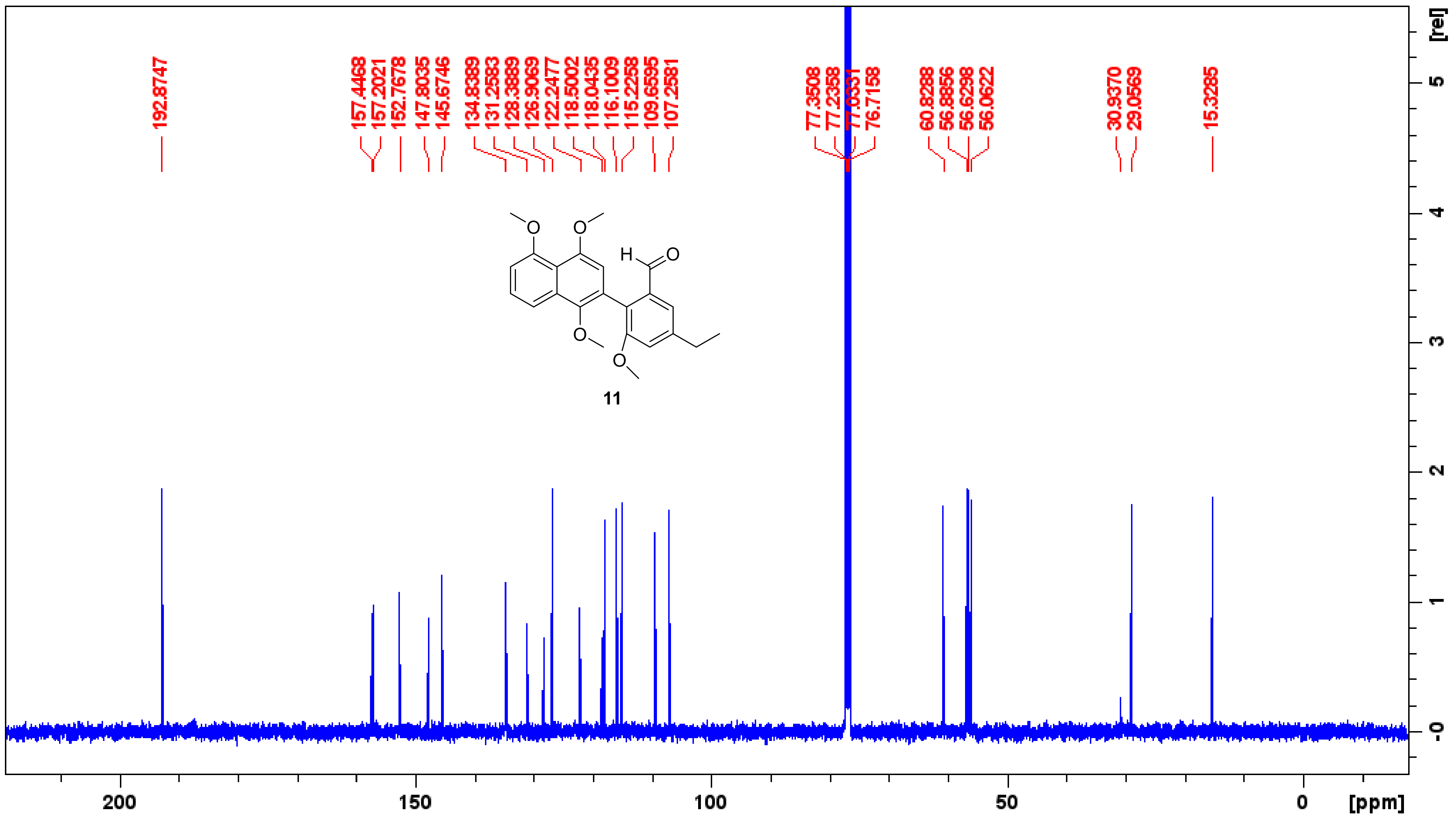

Figure S77. ^{13}C NMR spectrum of compound **11**.

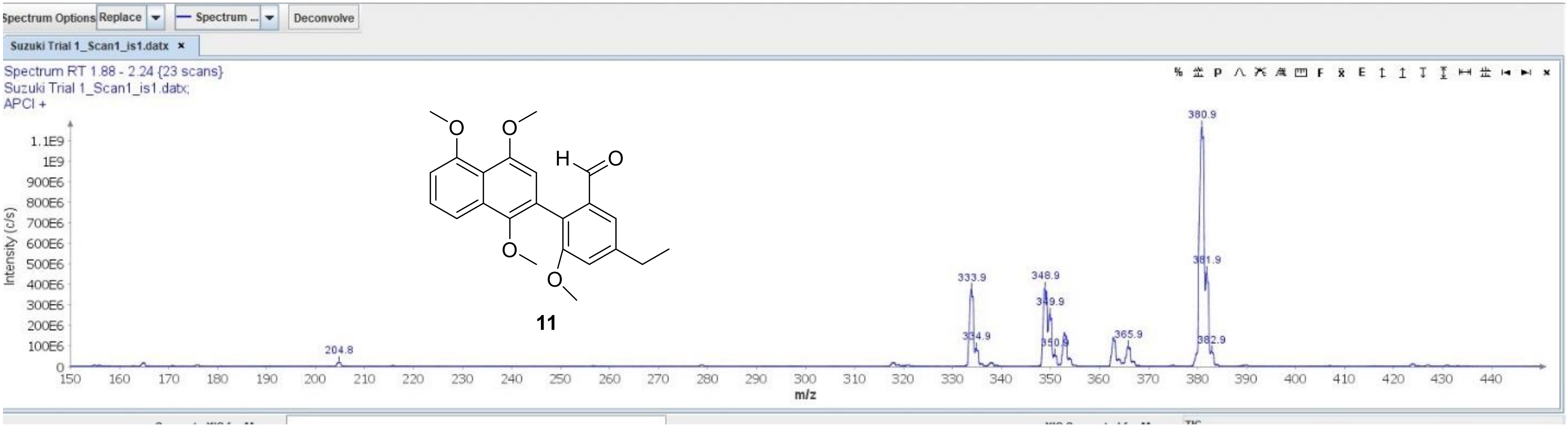

Figure S78. APCI-MS spectrum of compound **11**. Polarity: Positive

Compound 12

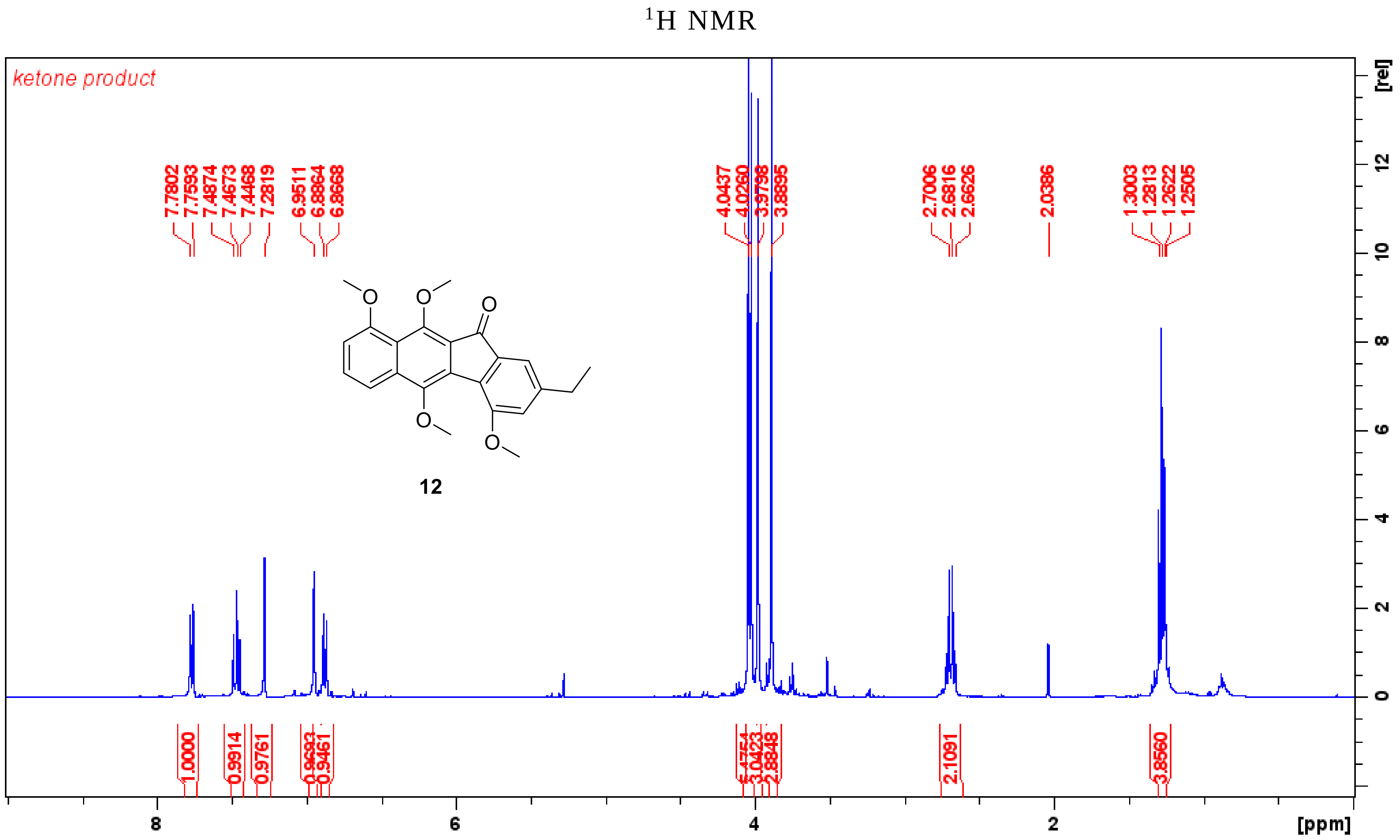

Figure S79. ^{1}H NMR spectrum of compound **12**.

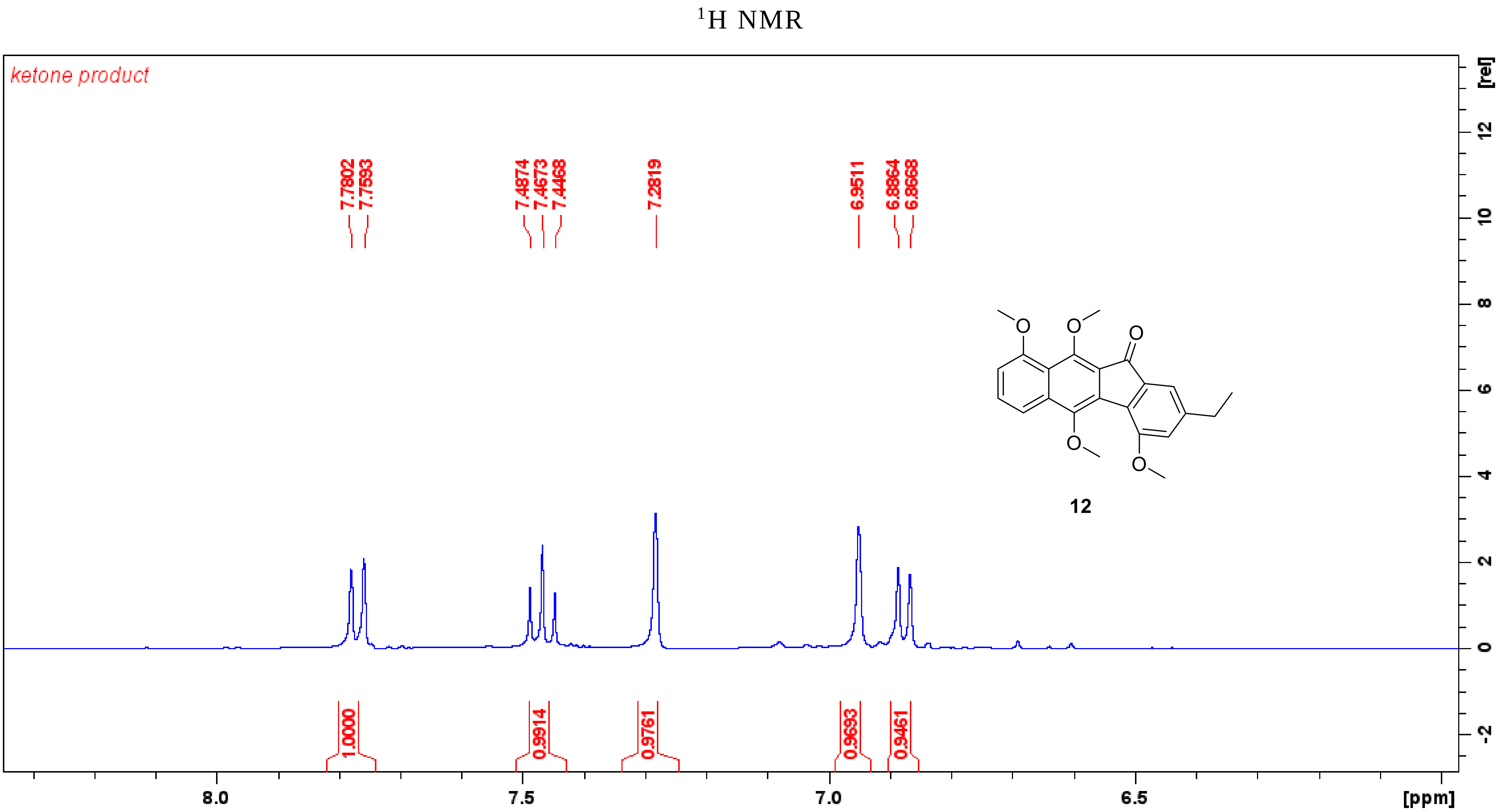

Figure S80. Zoomed in ¹H NMR spectrum of compound **12**.

^{1}H NMR

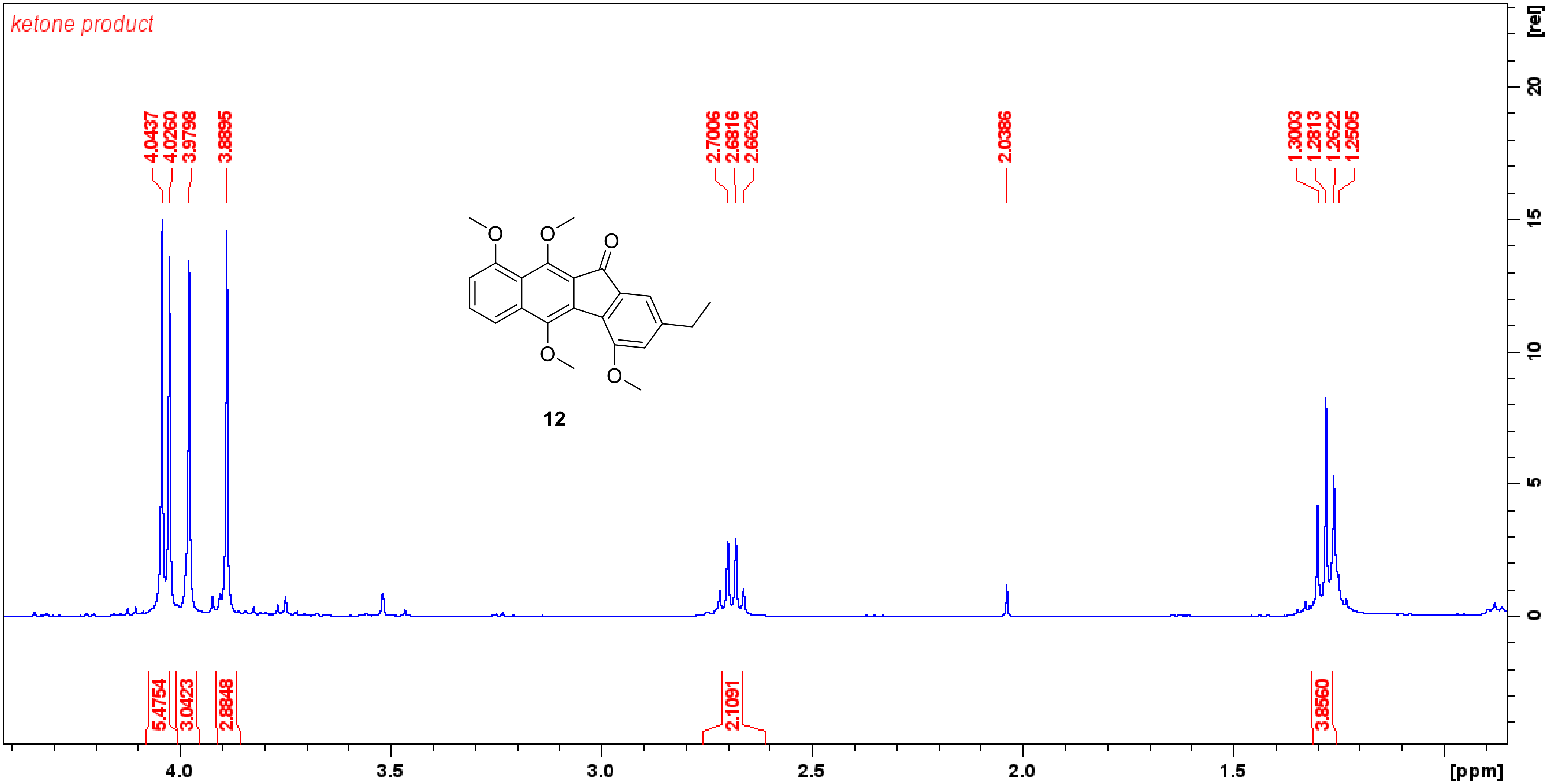

Figure S81. Zoomed in ^{1}H NMR spectrum of compound **12**.

^{13}C NMR

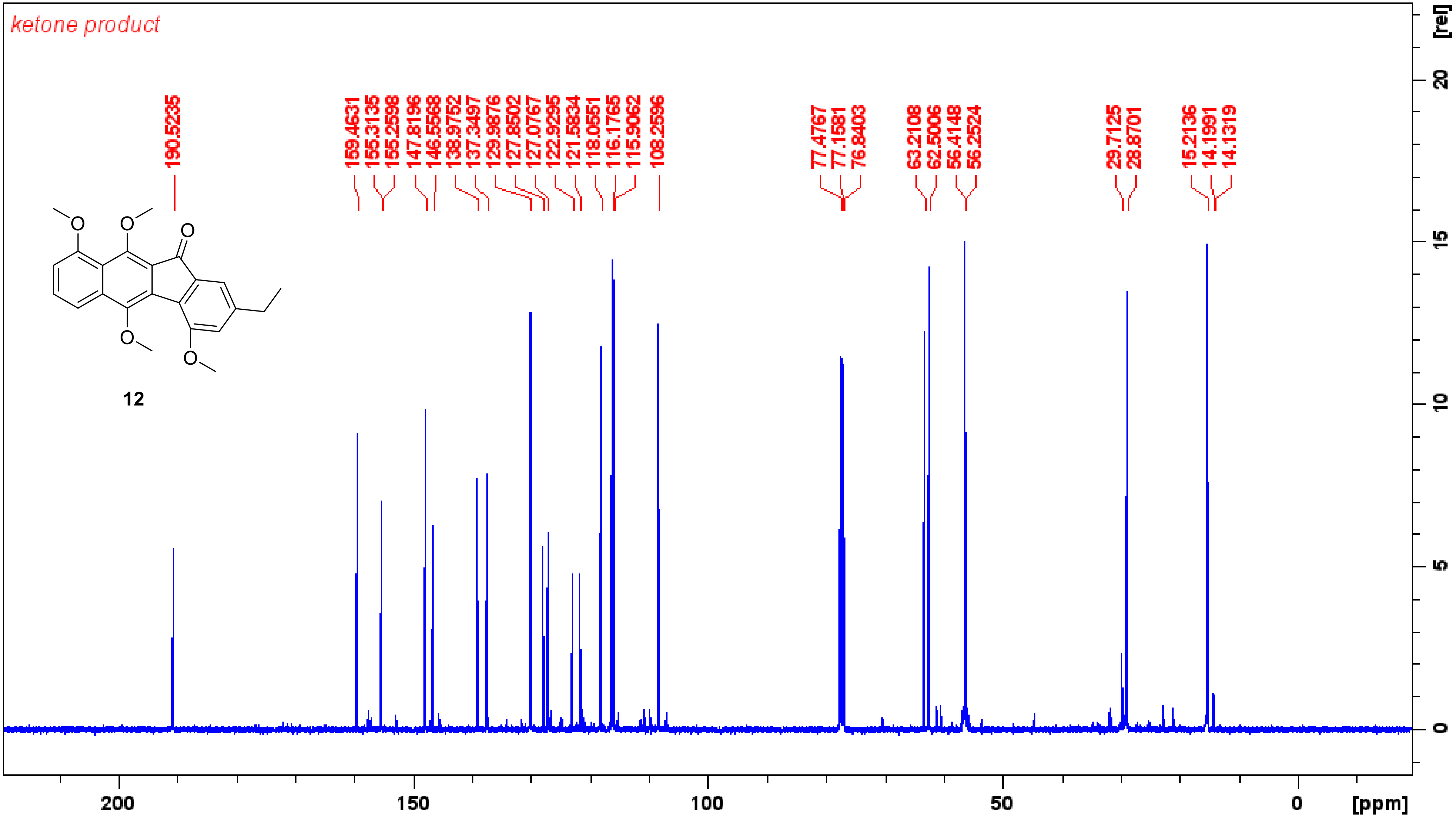

Figure S82. ^{13}C NMR spectrum of compound **12**.

TLCMS

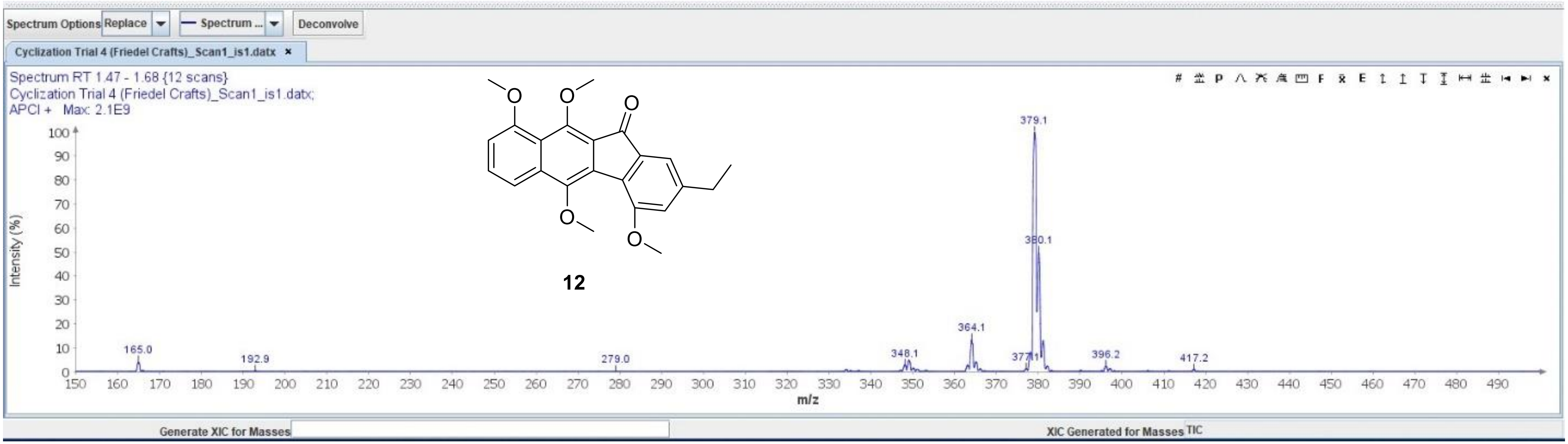

Figure S83. APCI-MS spectrum of compound **12**. Polarity: Positive

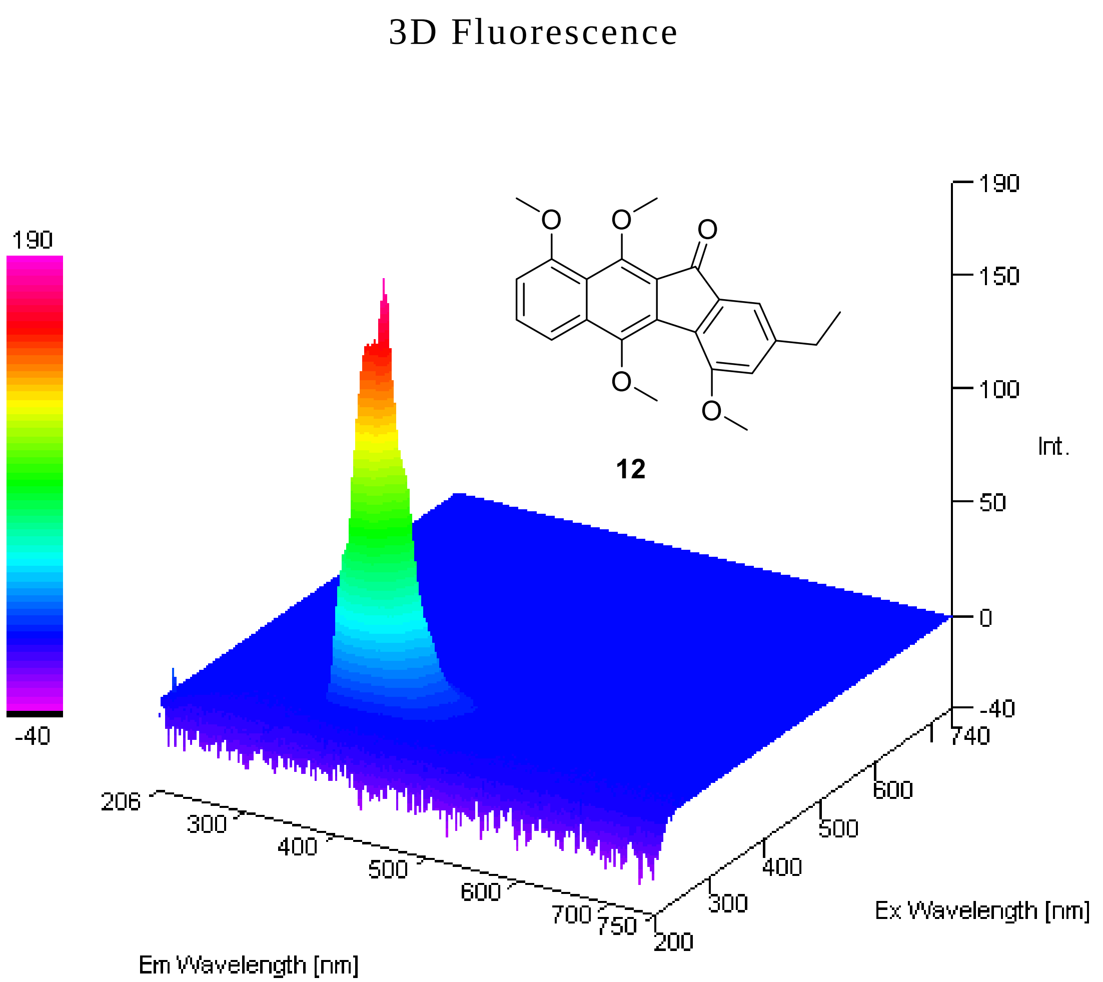

Figure S84. 3D excitation-emission fluorescence spectrum of compound **12**. Concentration: 0.1 mg/mL in MeOH. Cuvette: 10 mm.

3D Fluorescence

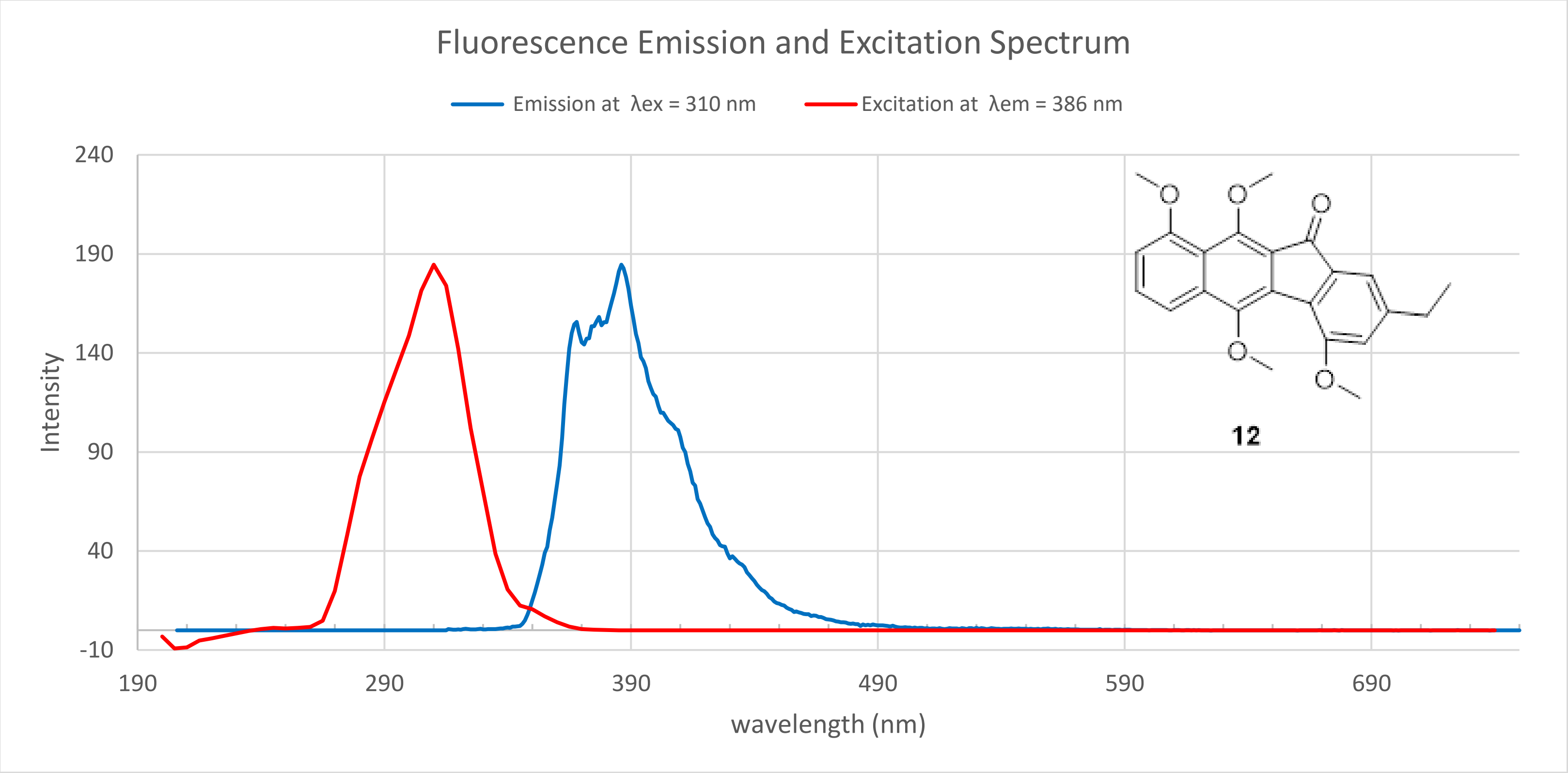

Figure S85. Overlaid emission (blue) and excitation (red) fluorescence spectra of compound **12**. Concentration: 0.1 mg/mL in MeOH. Cuvette: 10 mm.

Compound 16

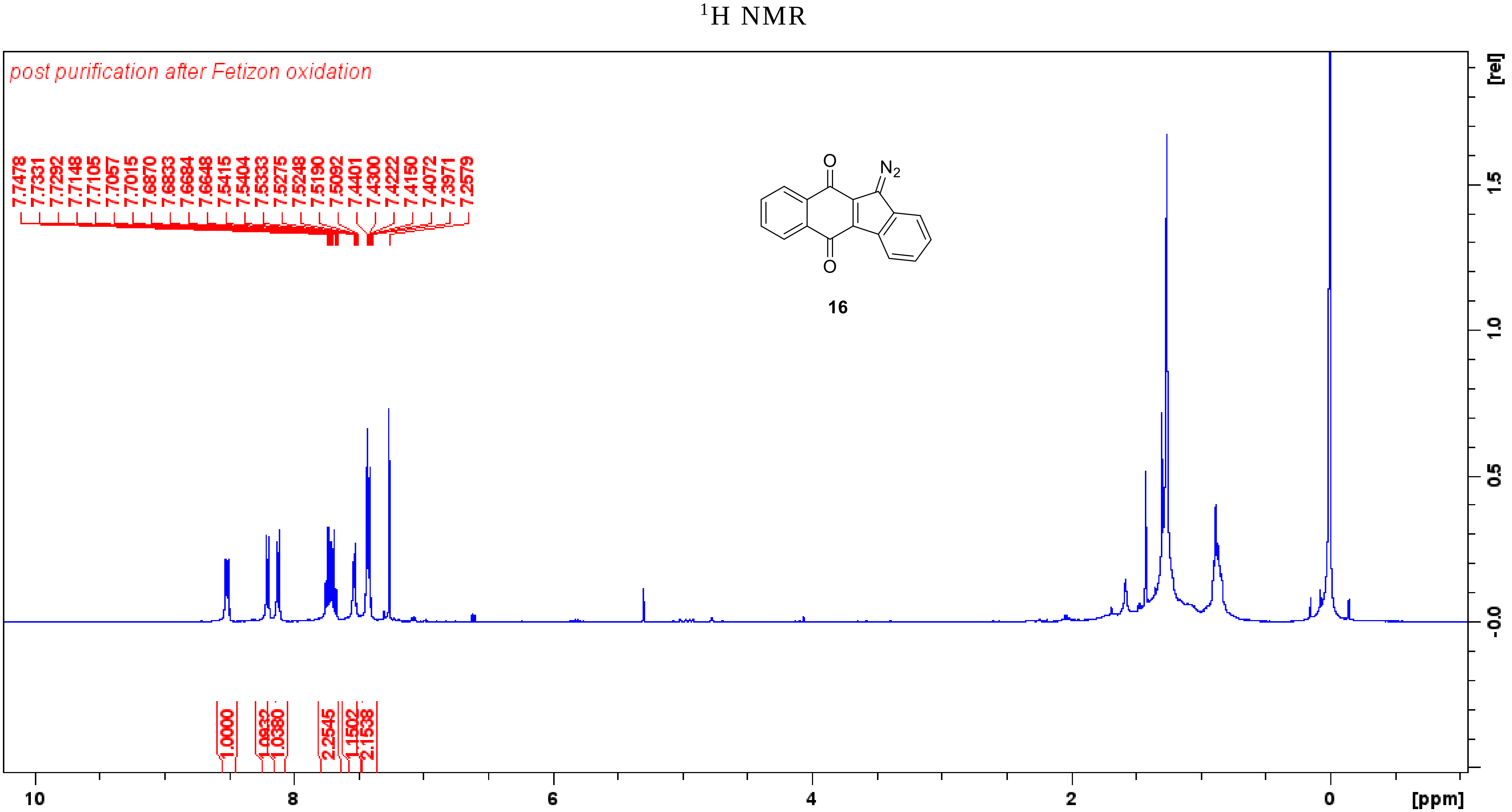

Figure S86. ^{1}H NMR spectrum of compound **16**.

^{1}H NMR

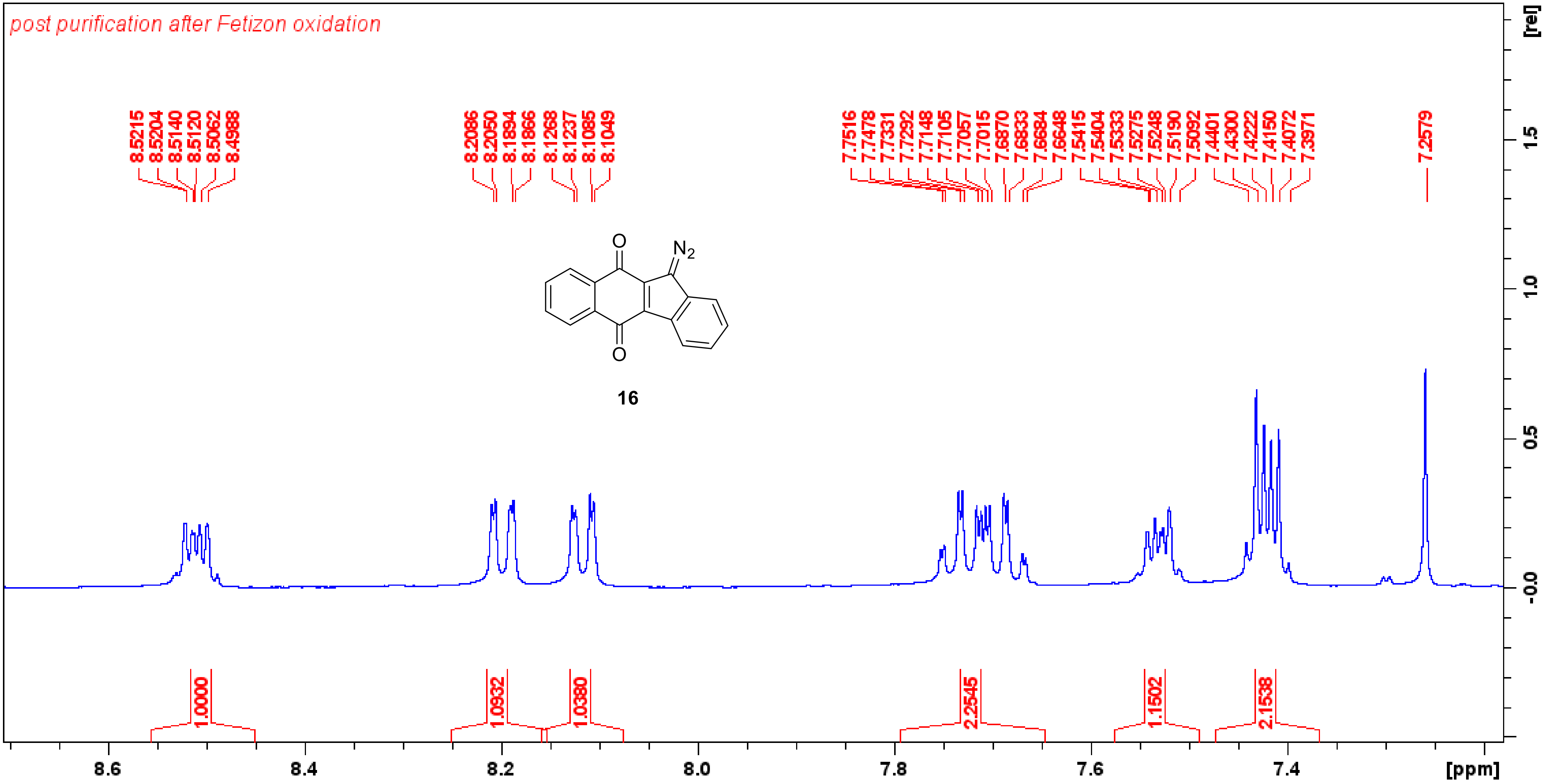

Figure S87. Zoomed in ^{1}H NMR spectrum of compound **16**.

^{13}C NMR

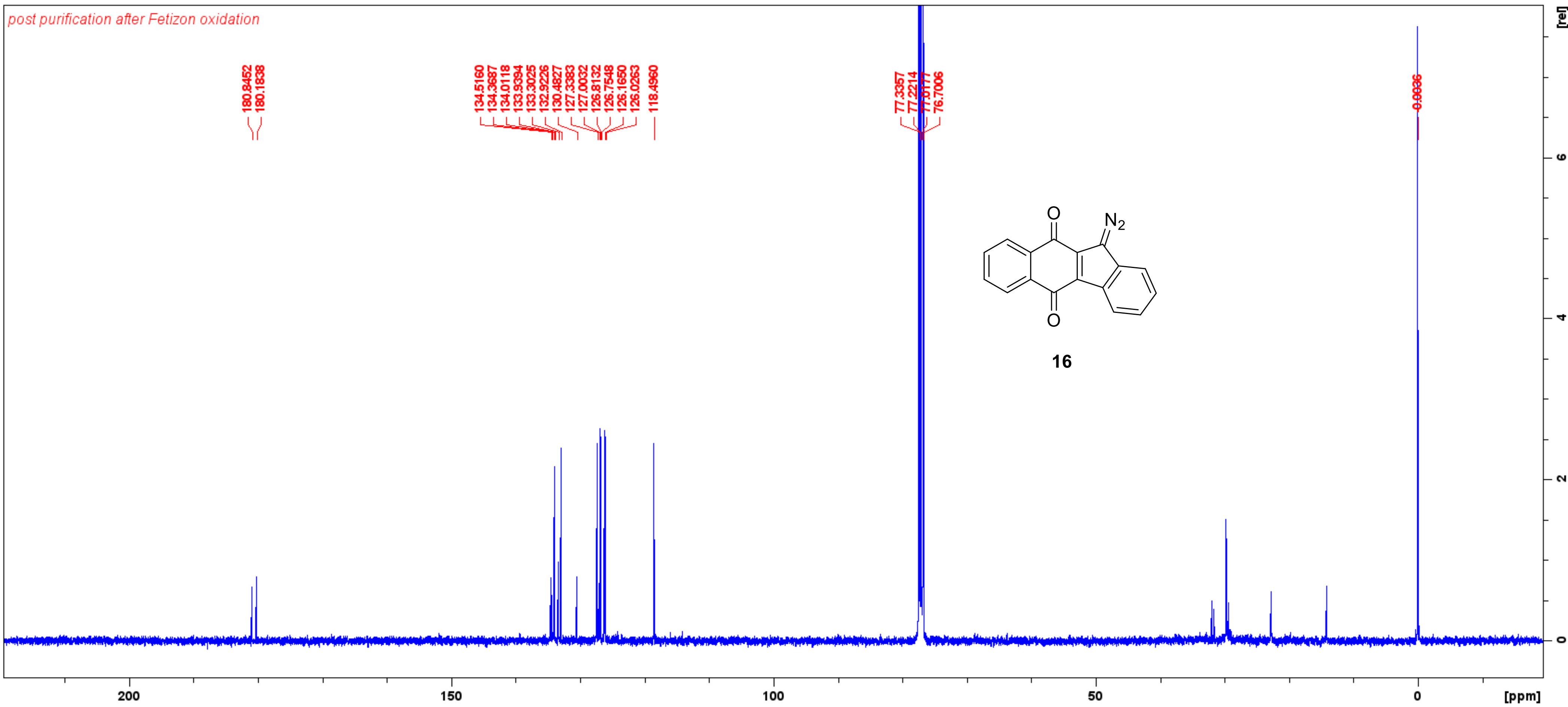

16

Figure S88. ^{13}C NMR spectrum of compound **16**.

^{13}C NMR

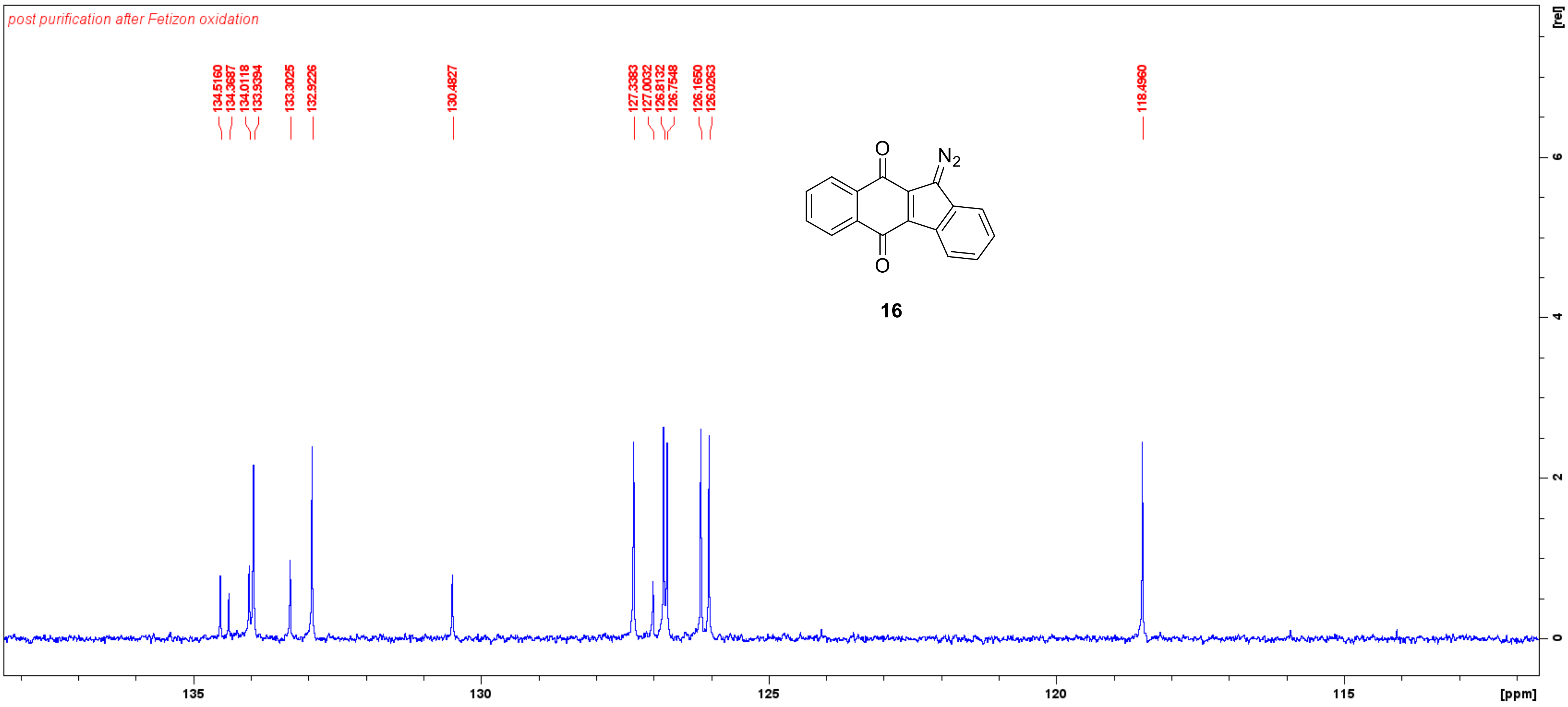

Figure S89. Zoomed in ^{13}C NMR spectrum of compound **16**.

TLCMS

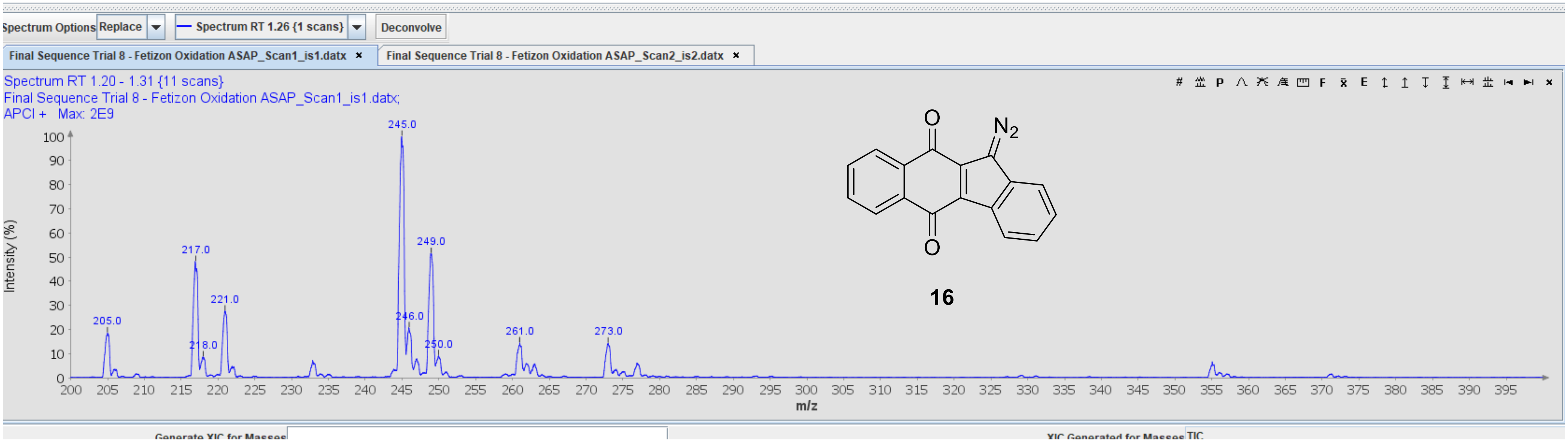

Figure S90. APCI-MS spectrum of compound **16**. Polarity: Positive

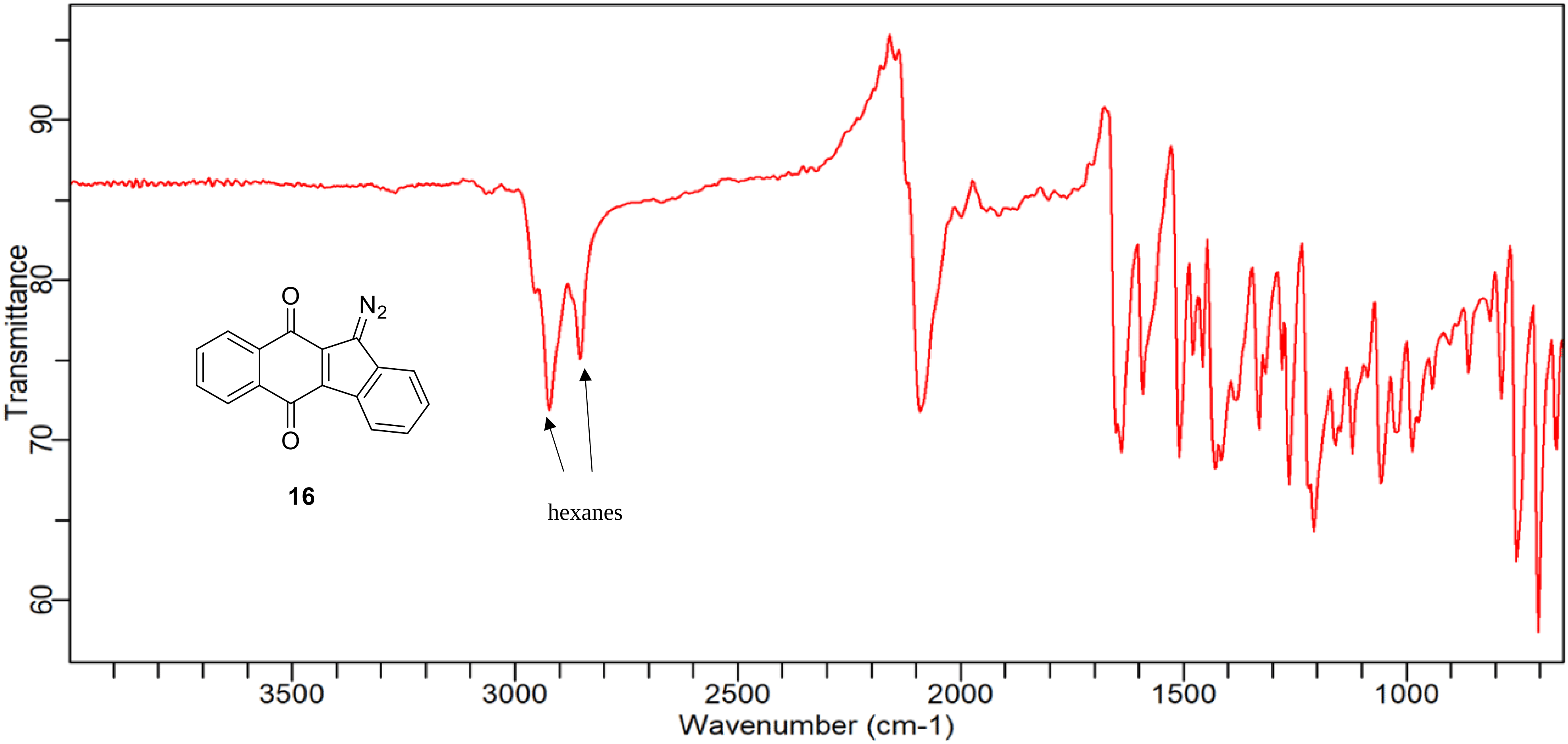

Figure S91. ATR-IR spectrum of compound **16**.

Compound 22

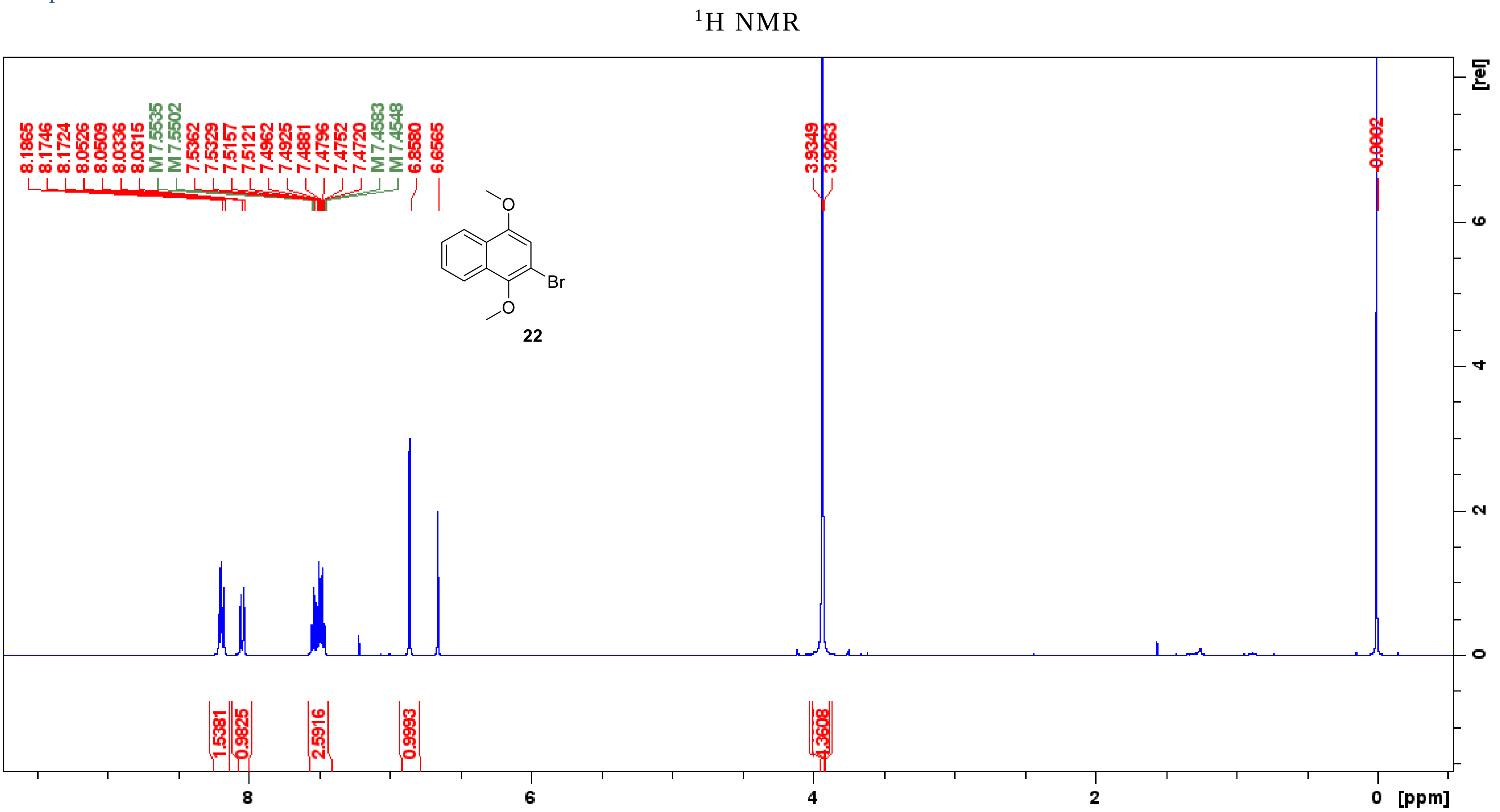

Figure S92. ^{1}H NMR spectrum of compound **22**.

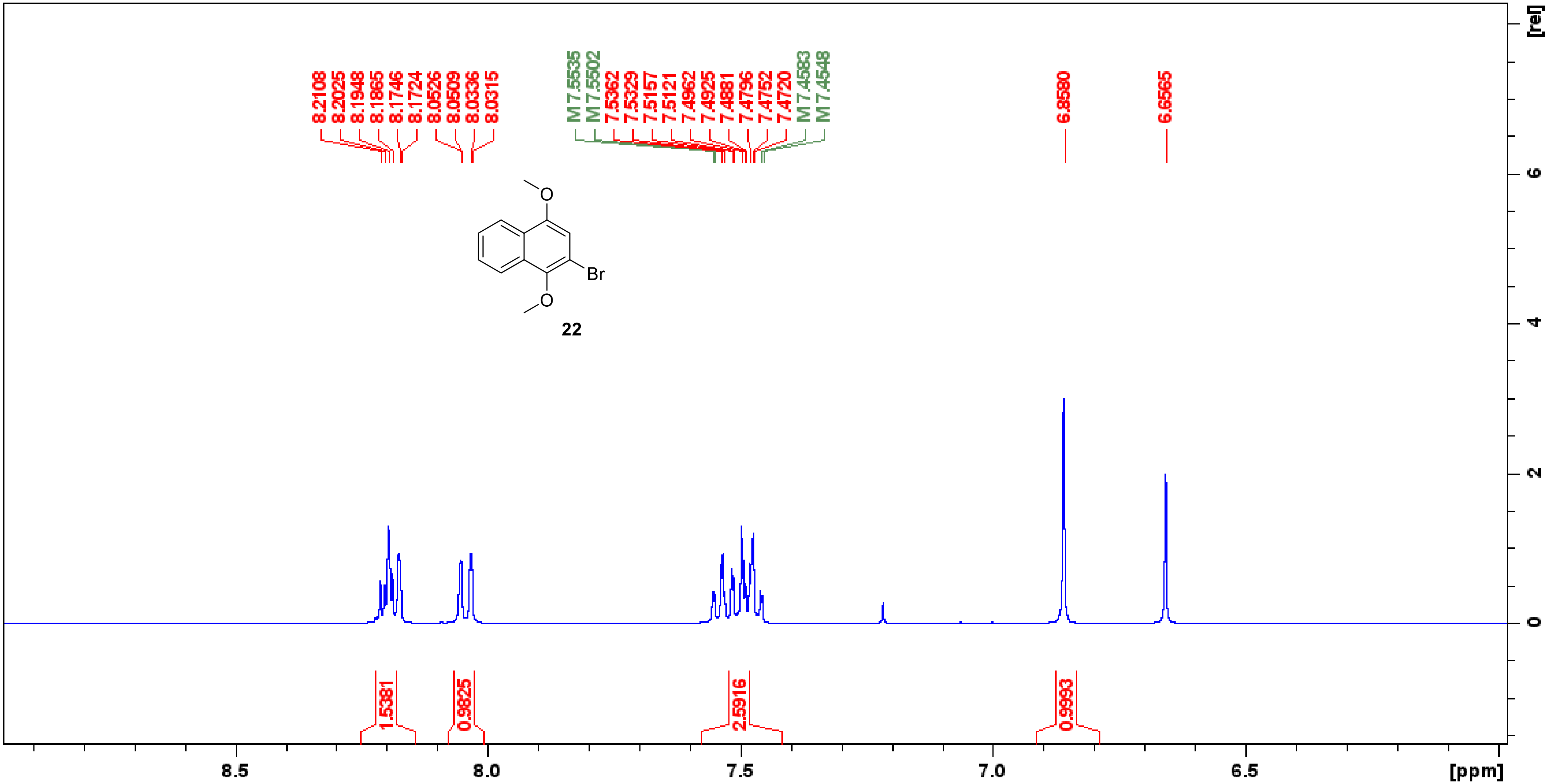

Figure S93. Zoomed in ^{1}H NMR spectrum of compound **22**.

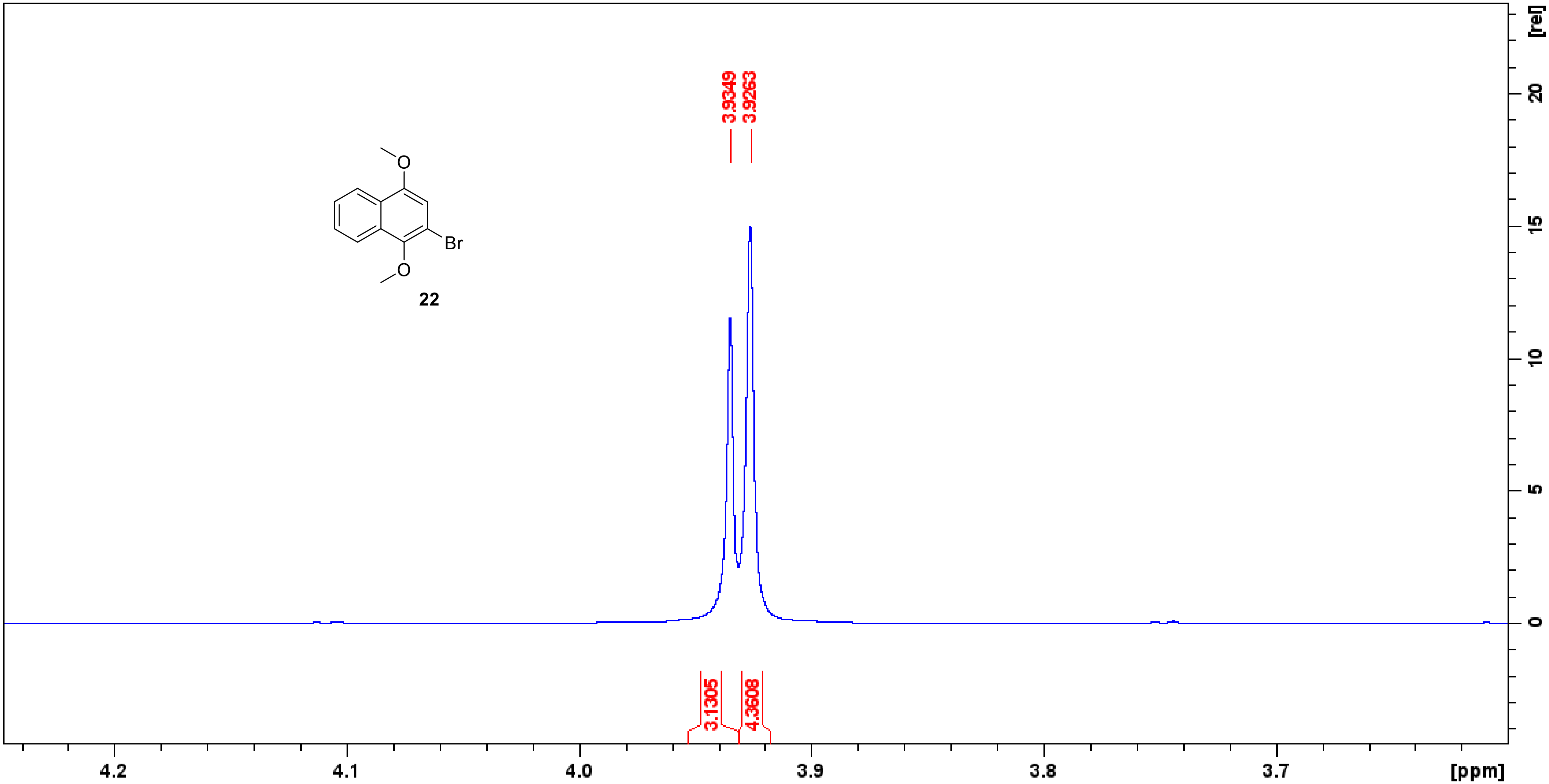

Figure S94. Zoomed in ^{1}H NMR spectrum of compound **22**.

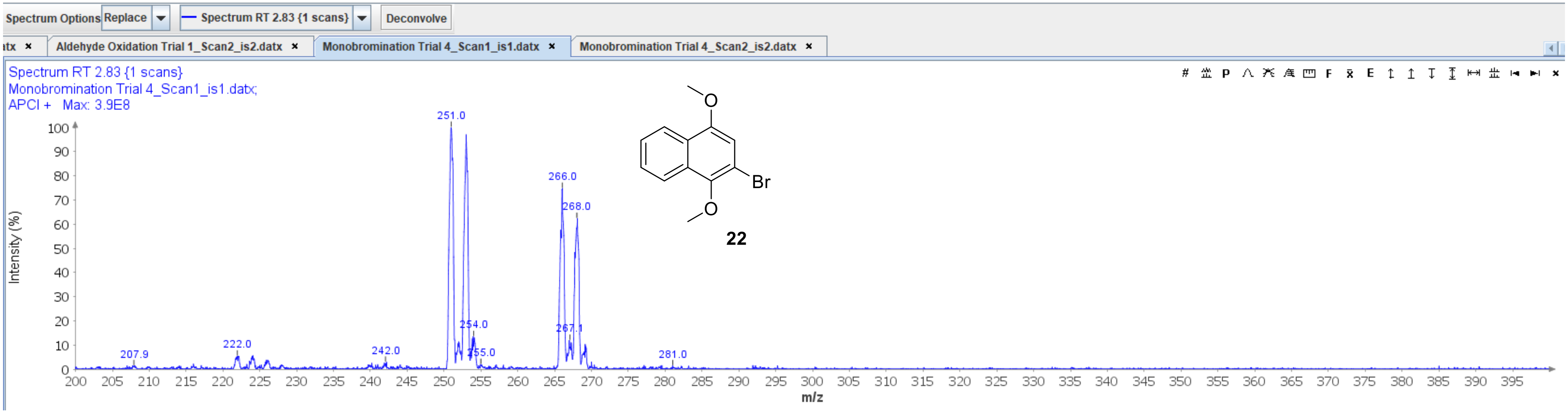

Figure S95. APCI-MS spectrum of compound **22**. Polarity: Positive

Compound 24

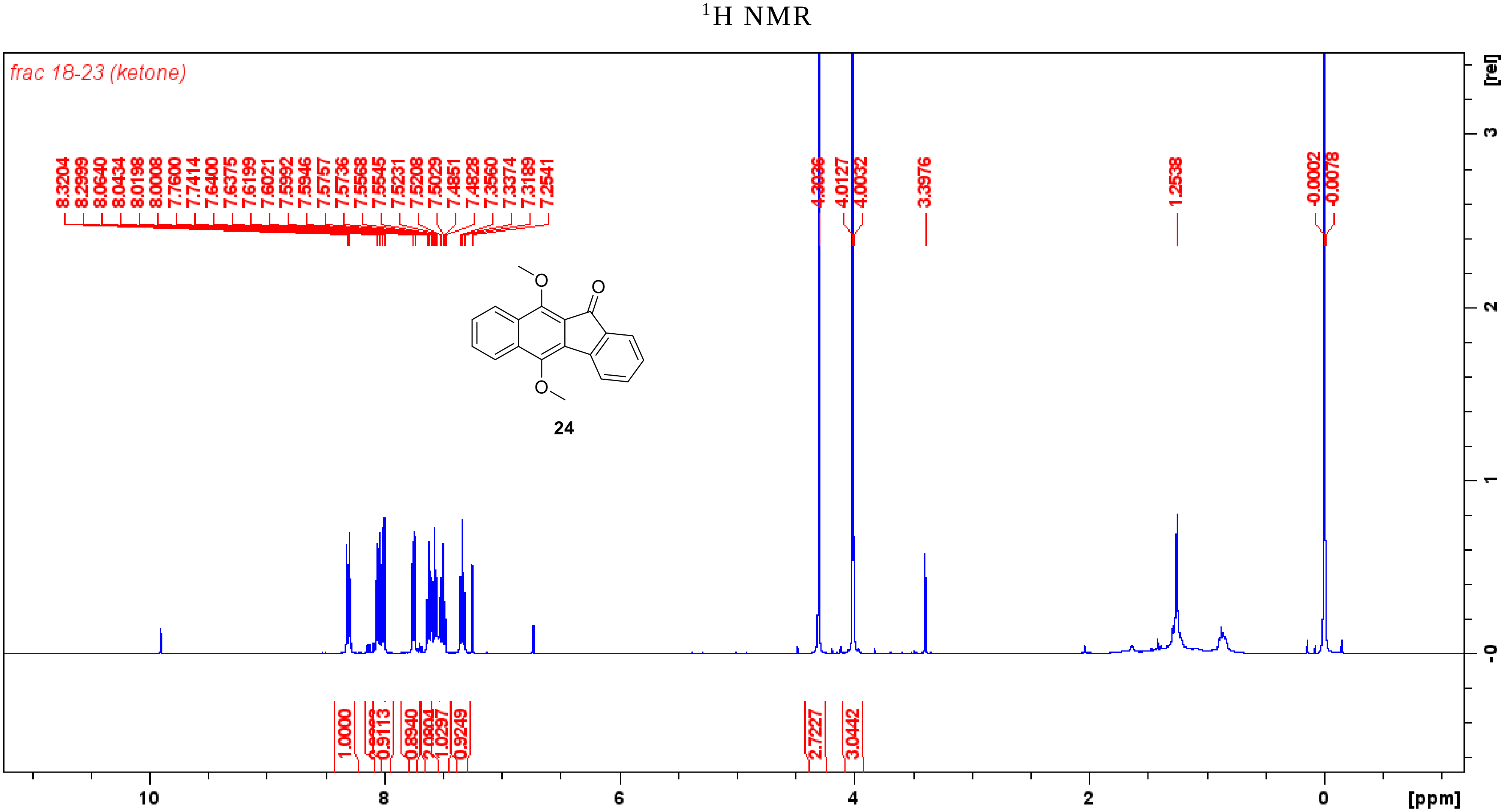

Figure S96. ¹H NMR spectrum of compound **24**.

^{1}H NMR

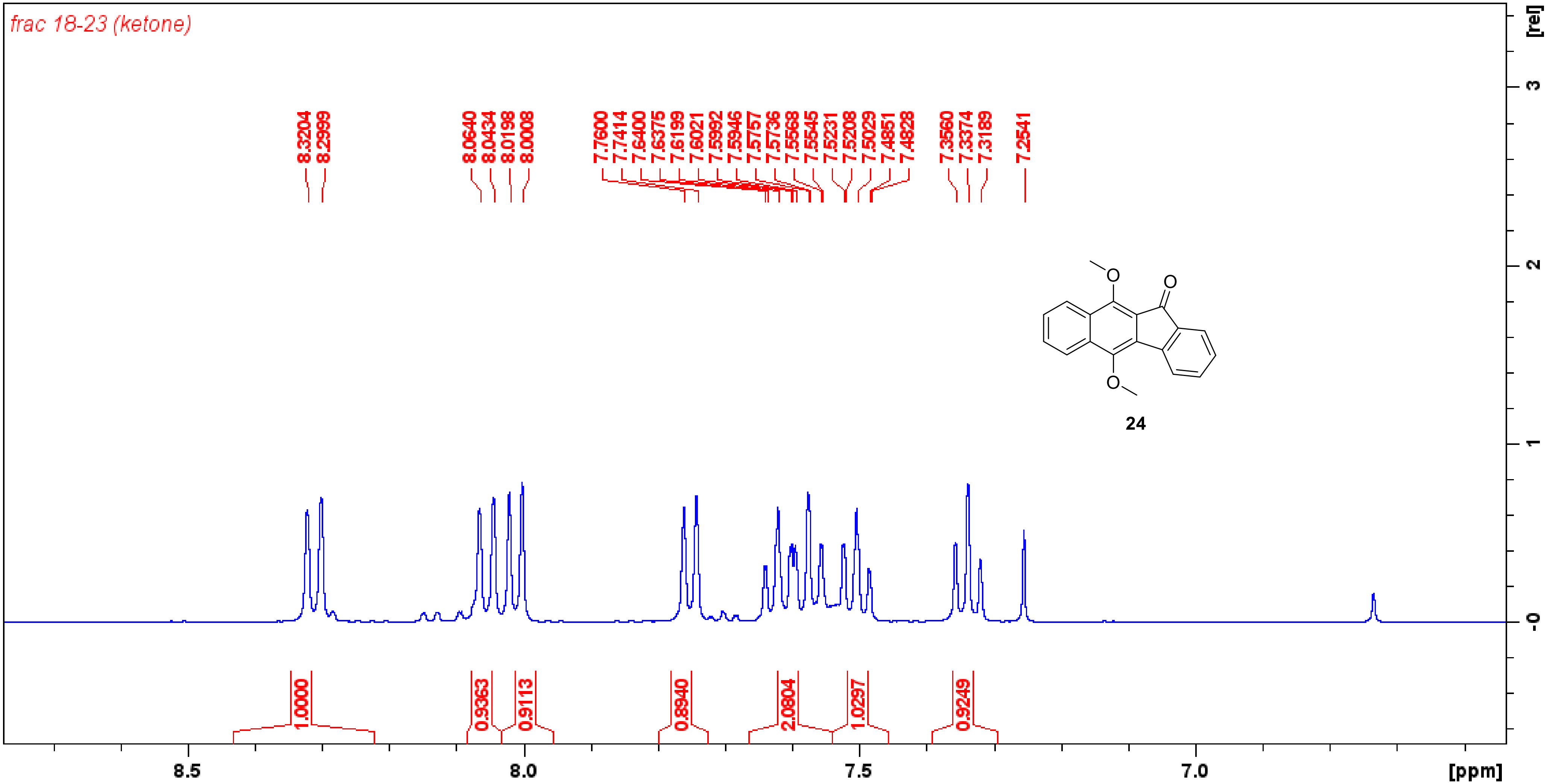

Figure S97. Zoomed in ^{1}H NMR spectrum of compound **24**.

^{13}C NMR

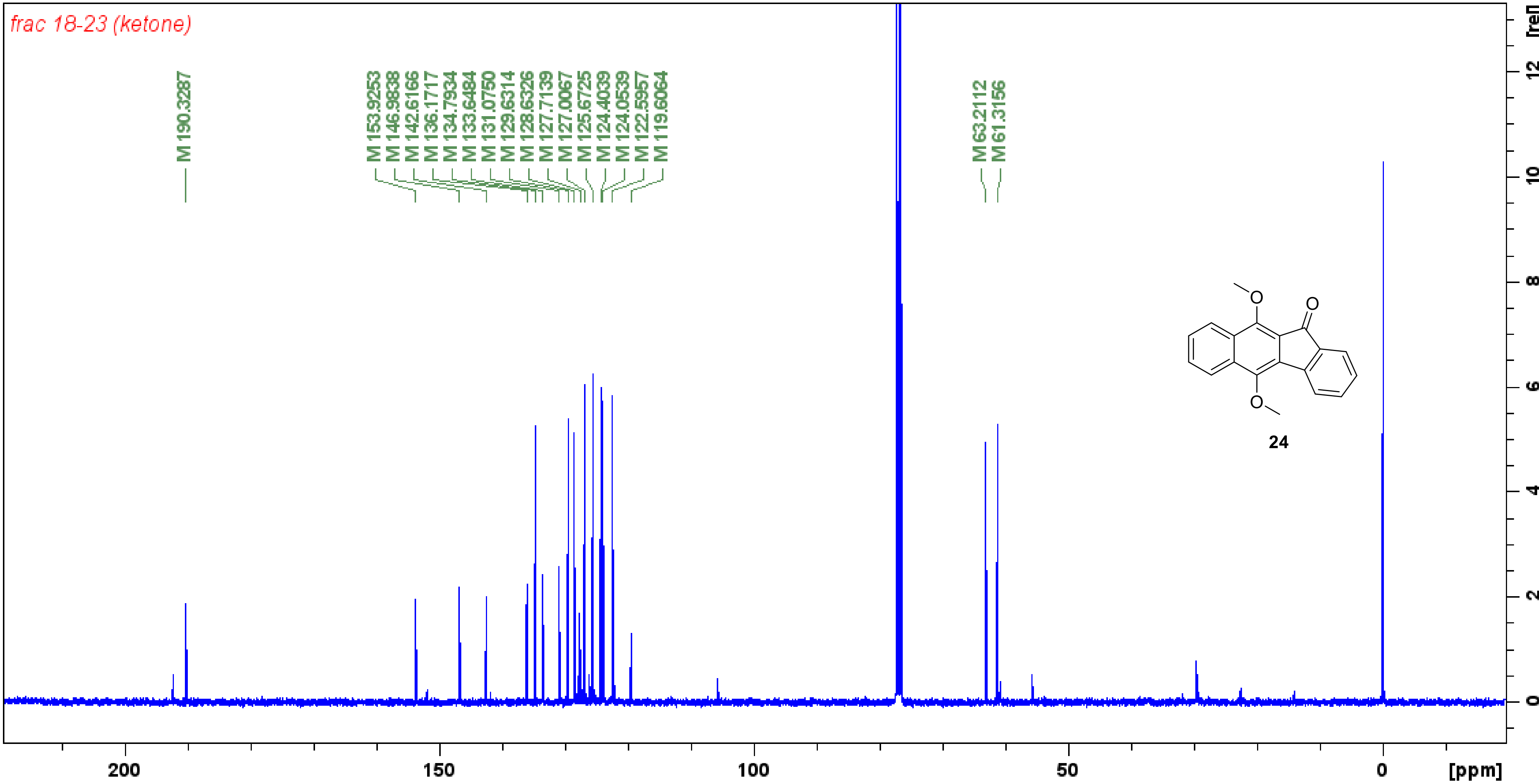

Figure S98. ^{13}C NMR spectrum of compound **24**.

^{13}C NMR

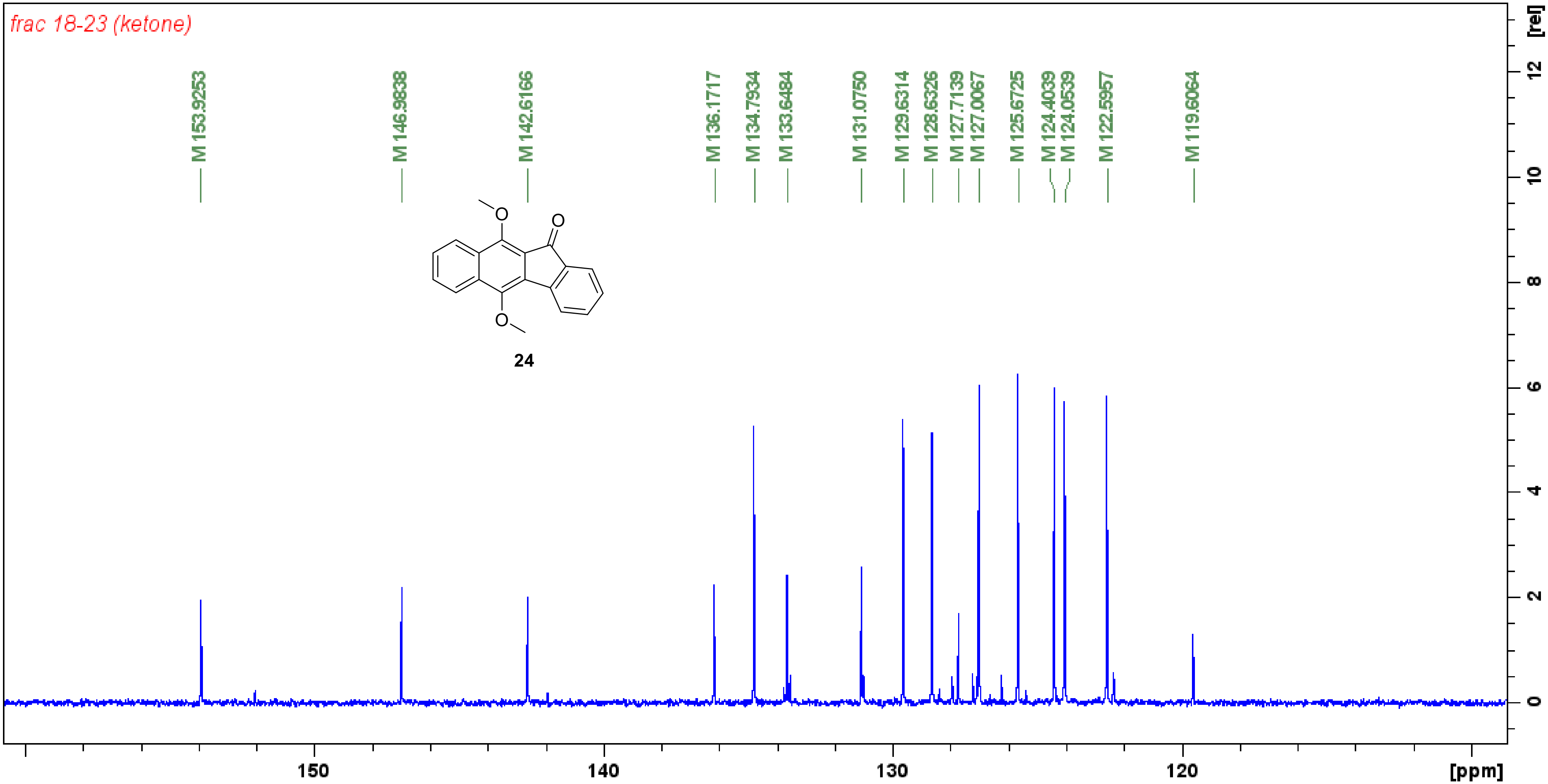

Figure S99. Zoomed in ^{13}C NMR spectrum of compound **24**.

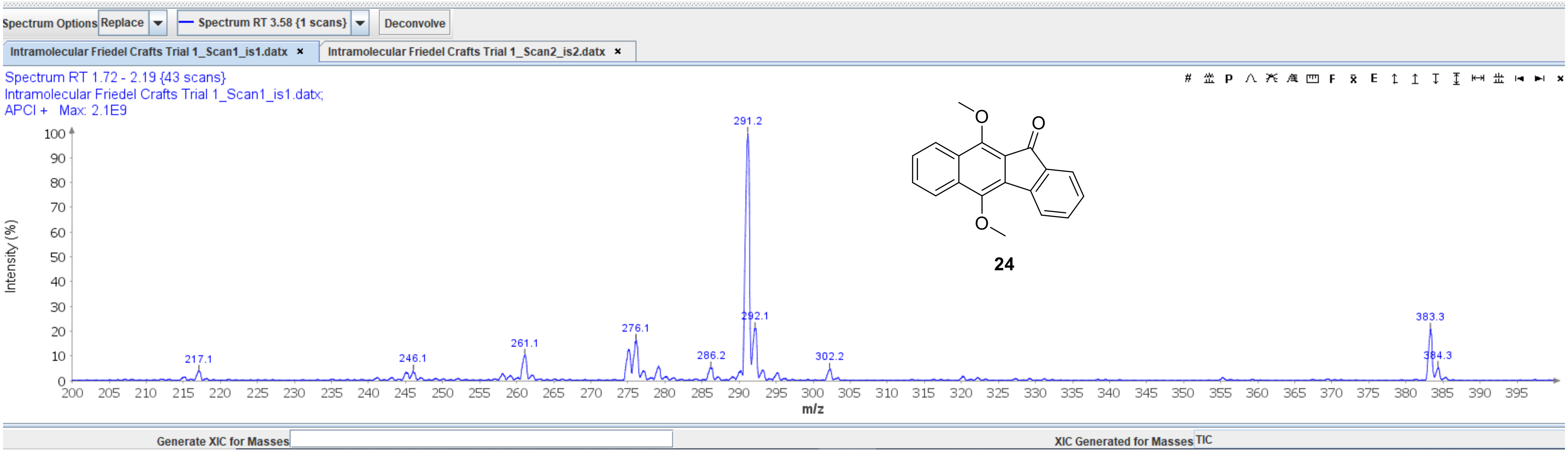

Figure S100. APCI-MS spectrum of compound **24**. Polarity: Positive

Compound 25

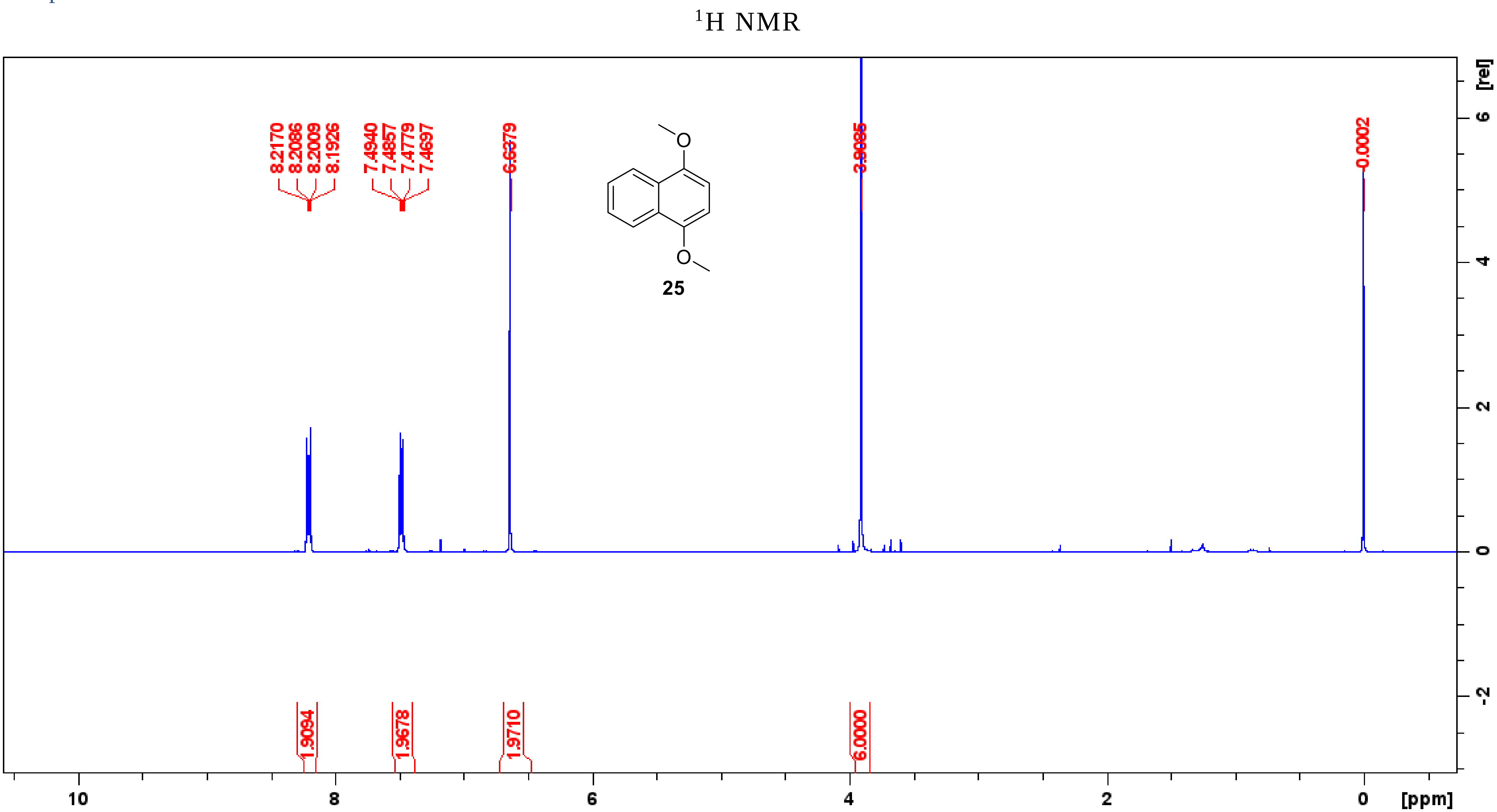

Figure S101. ^{1}H NMR spectrum of compound **25**.

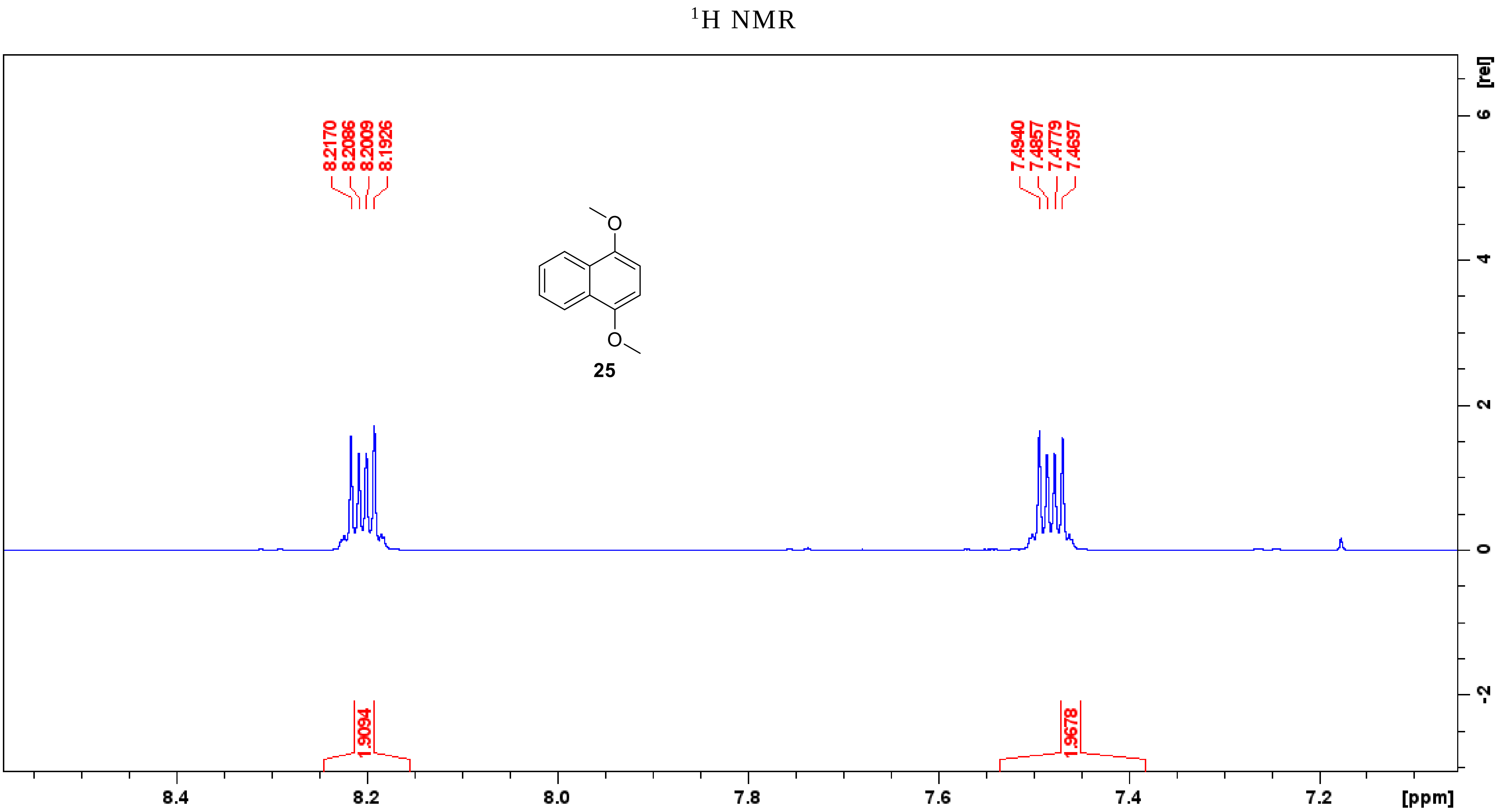

Figure S102. Zoomed in ^{1}H NMR spectrum of compound **25**.

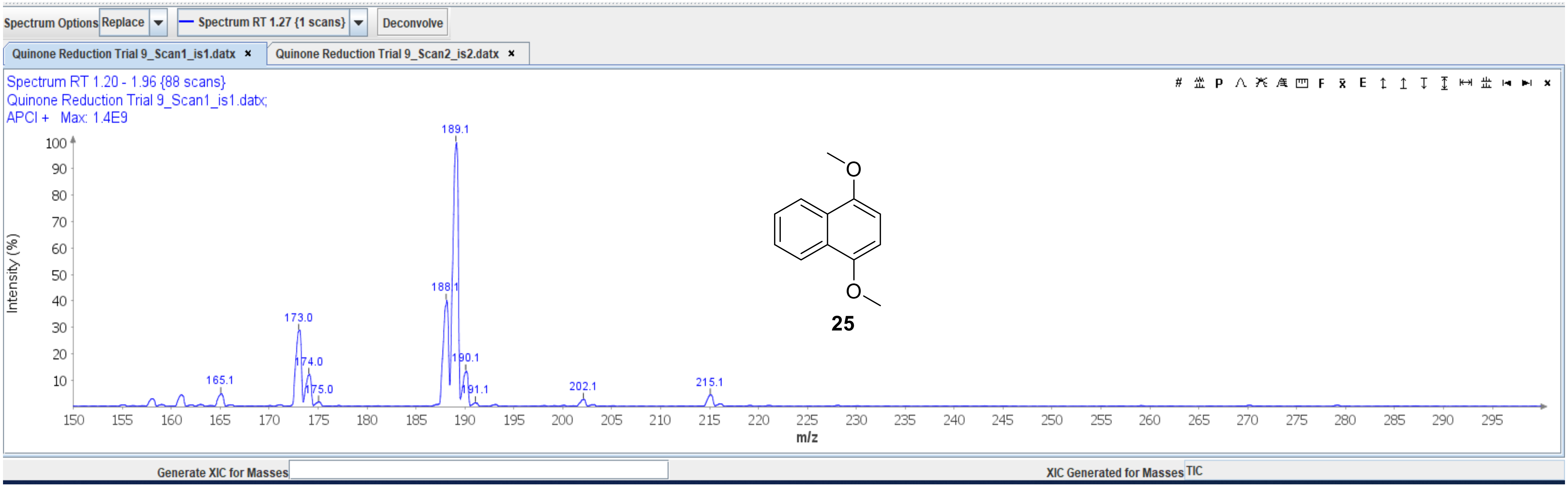

Figure S103. APCI-MS spectrum of compound **25**. Polarity: Positive

Compound 27

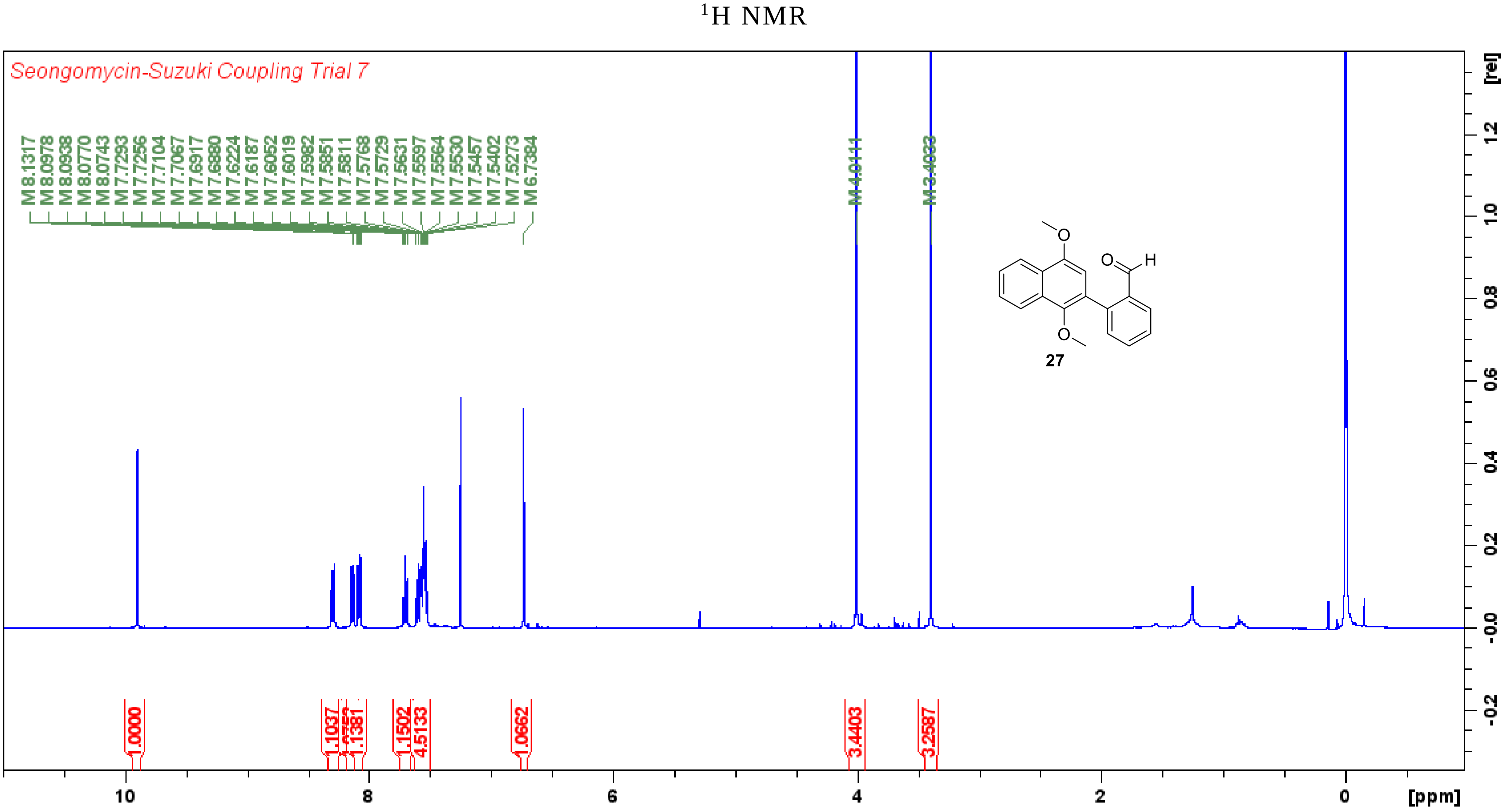

Figure S104. ¹H NMR spectrum of compound **27**.

^{1}H NMR

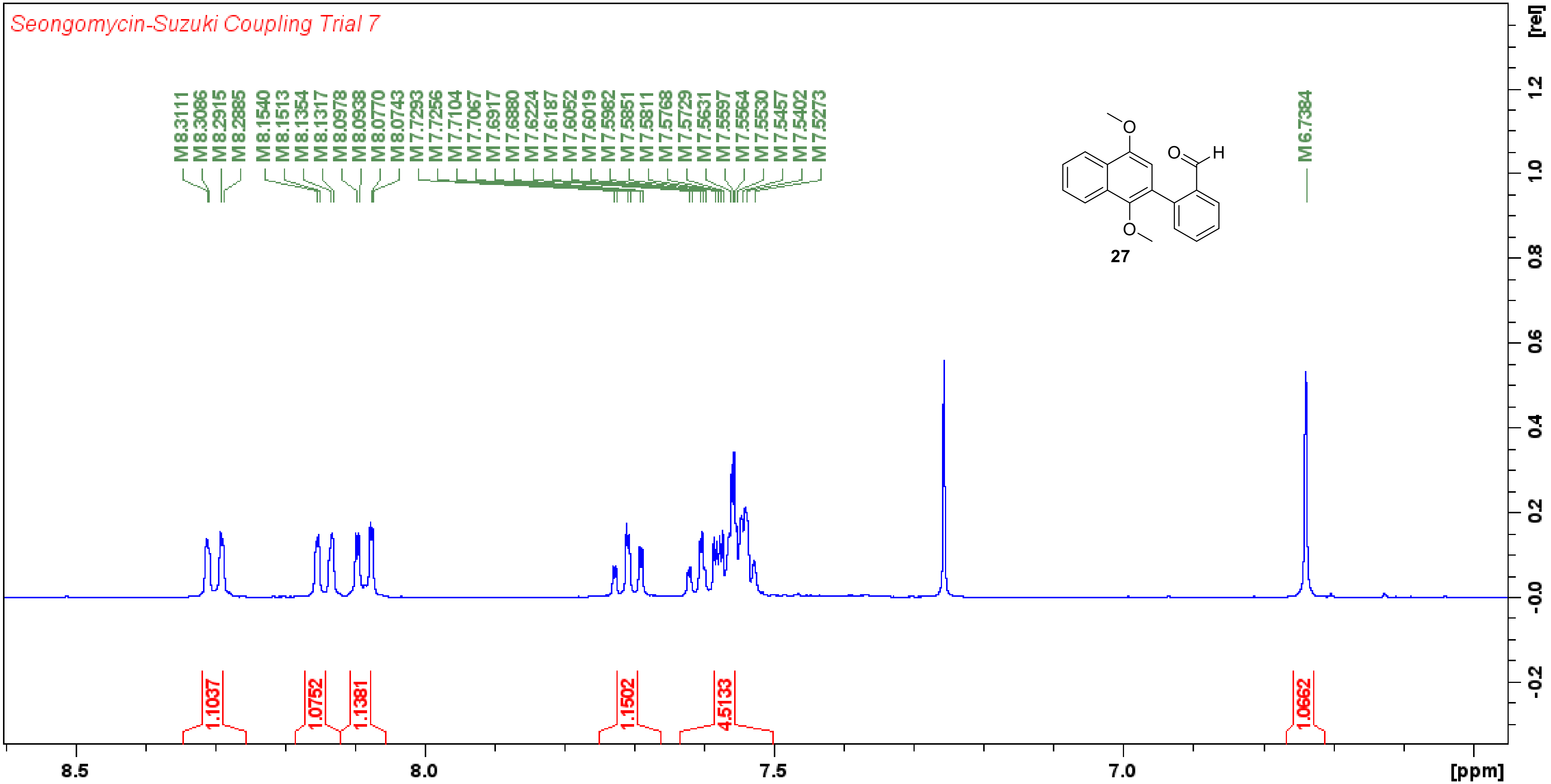

Figure S105. Zoomed in ^{1}H NMR spectrum of compound **27**.

Compound 28

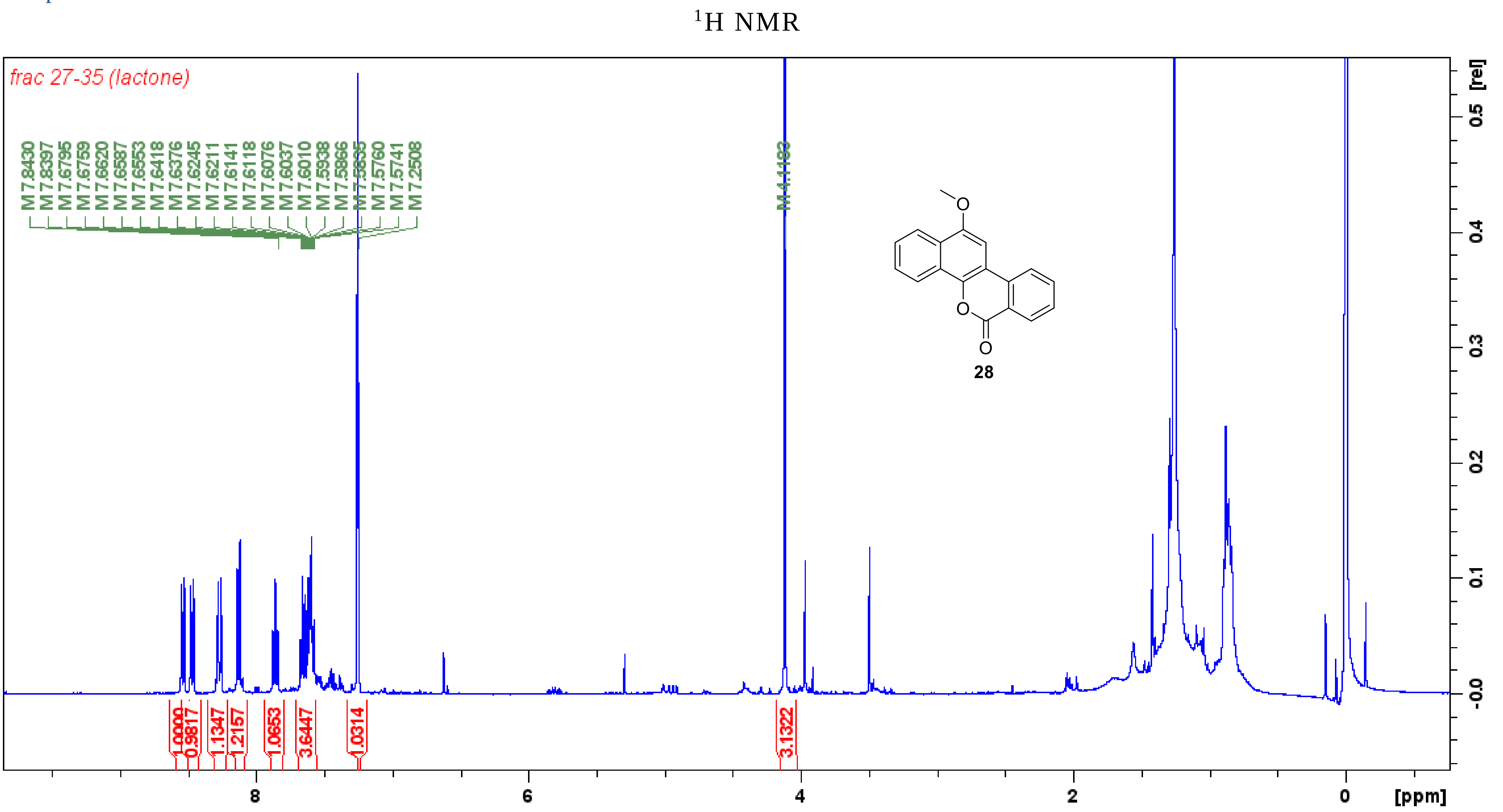

Figure S106. ¹H NMR spectrum of compound **28**.

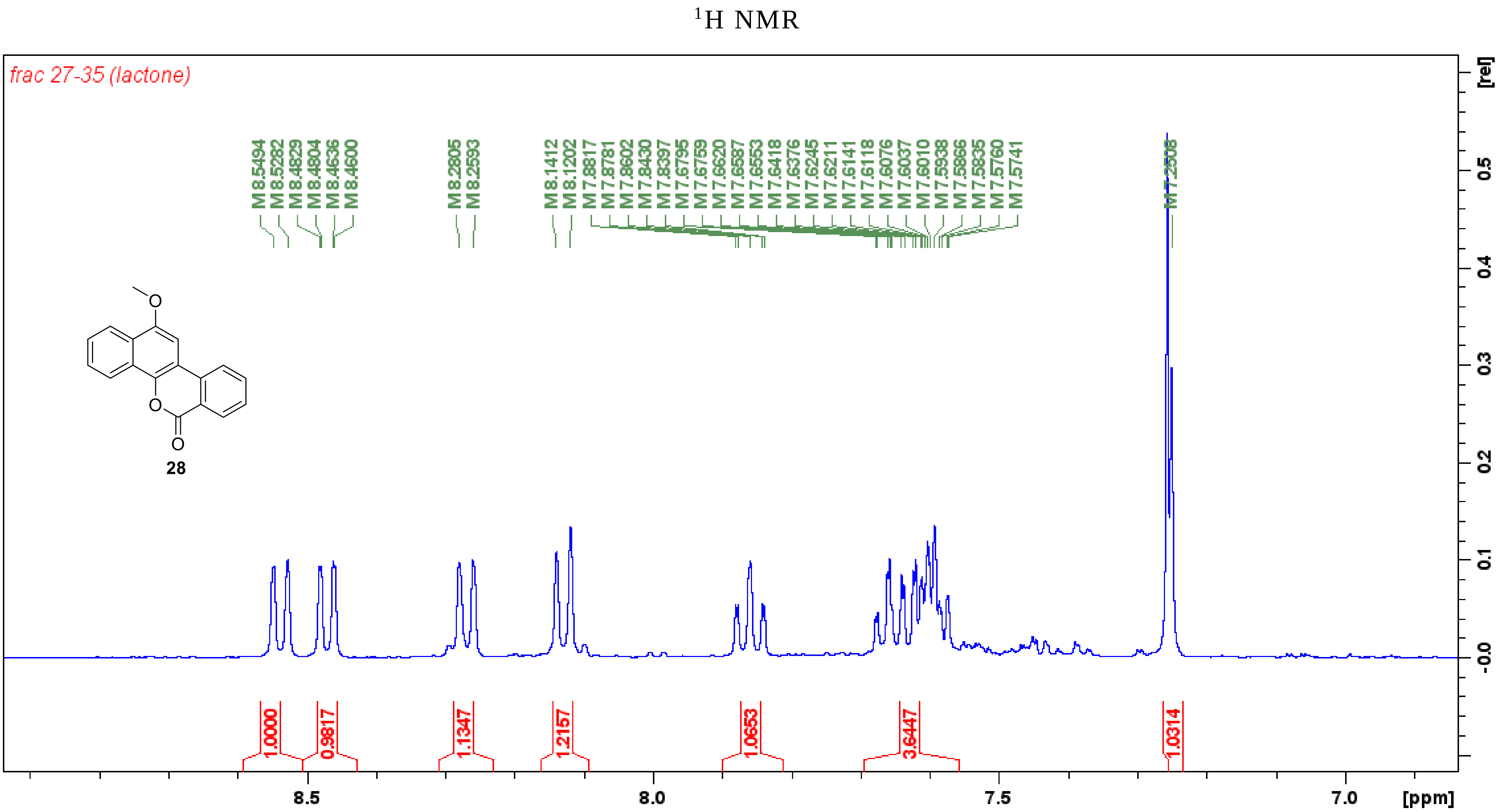

Figure S107. Zoomed in ^{1}H NMR spectrum of compound **28**.

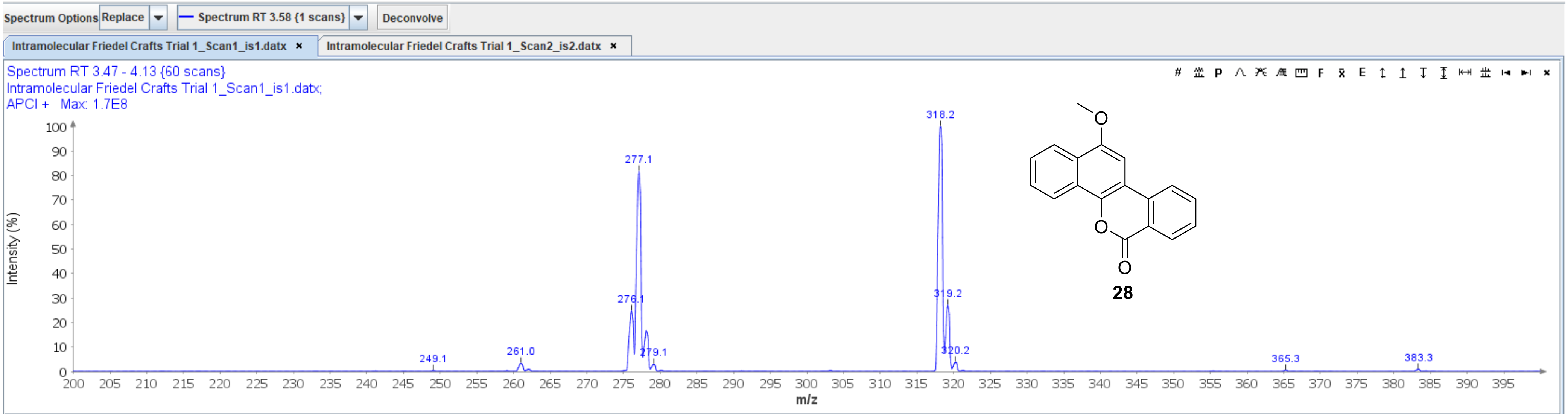

Figure S108. APCI-MS spectrum of compound **28**. Polarity: Positive

Compound 31

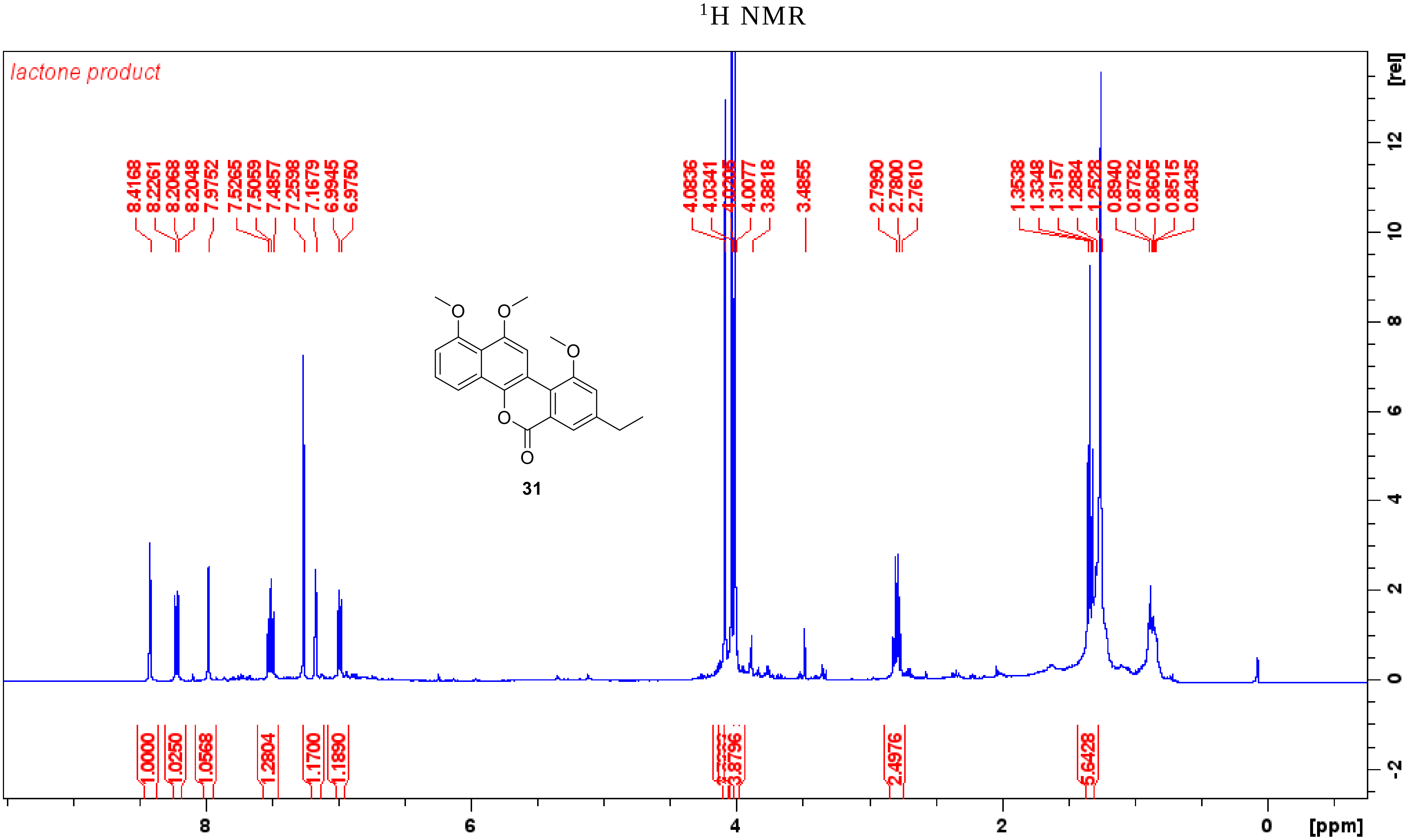

Figure S109. ^{1}H NMR spectrum of compound **31**.

^{1}H NMR

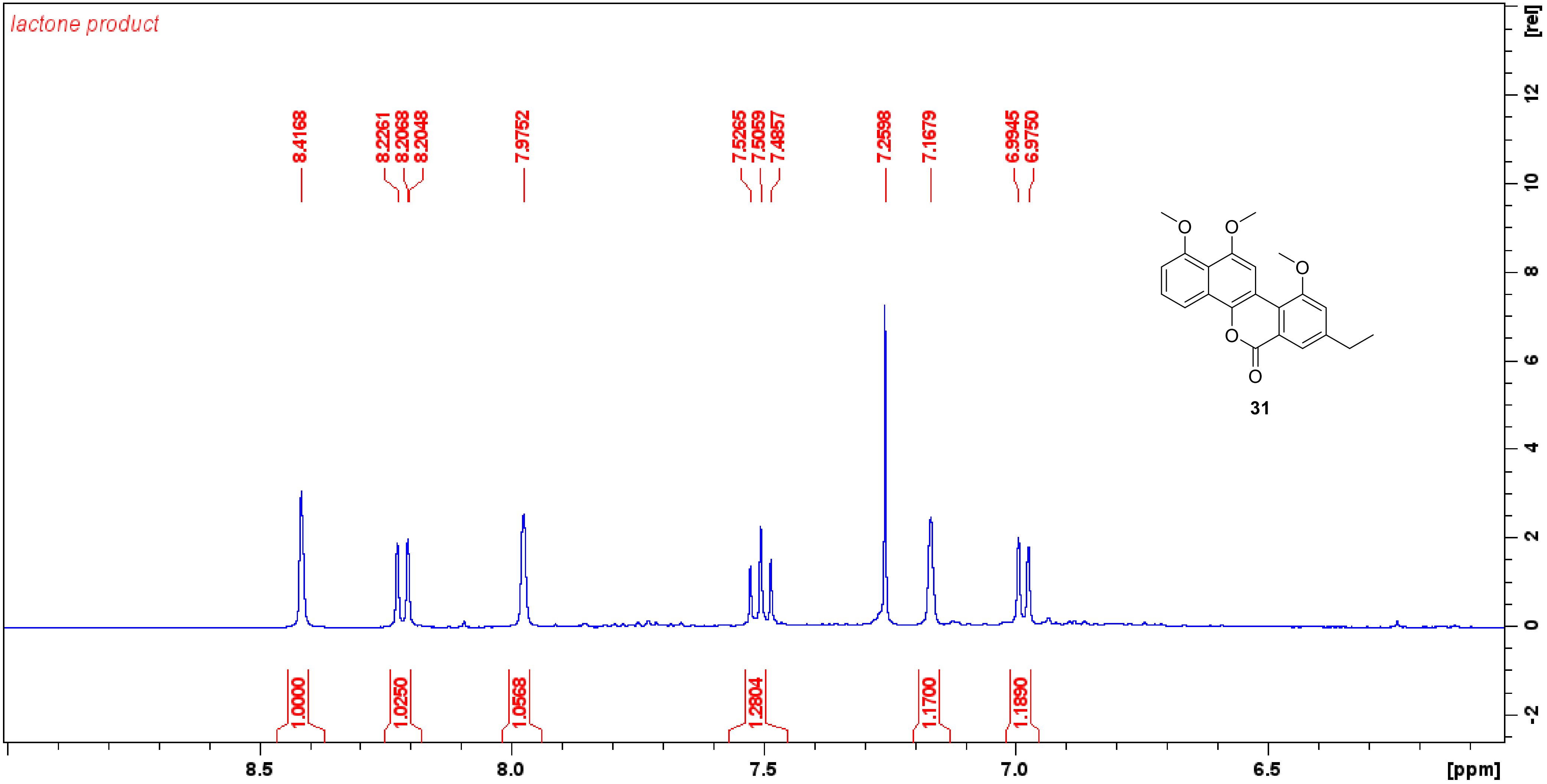

Figure S110. Zoomed in ^{1}H NMR spectrum of compound **31**.

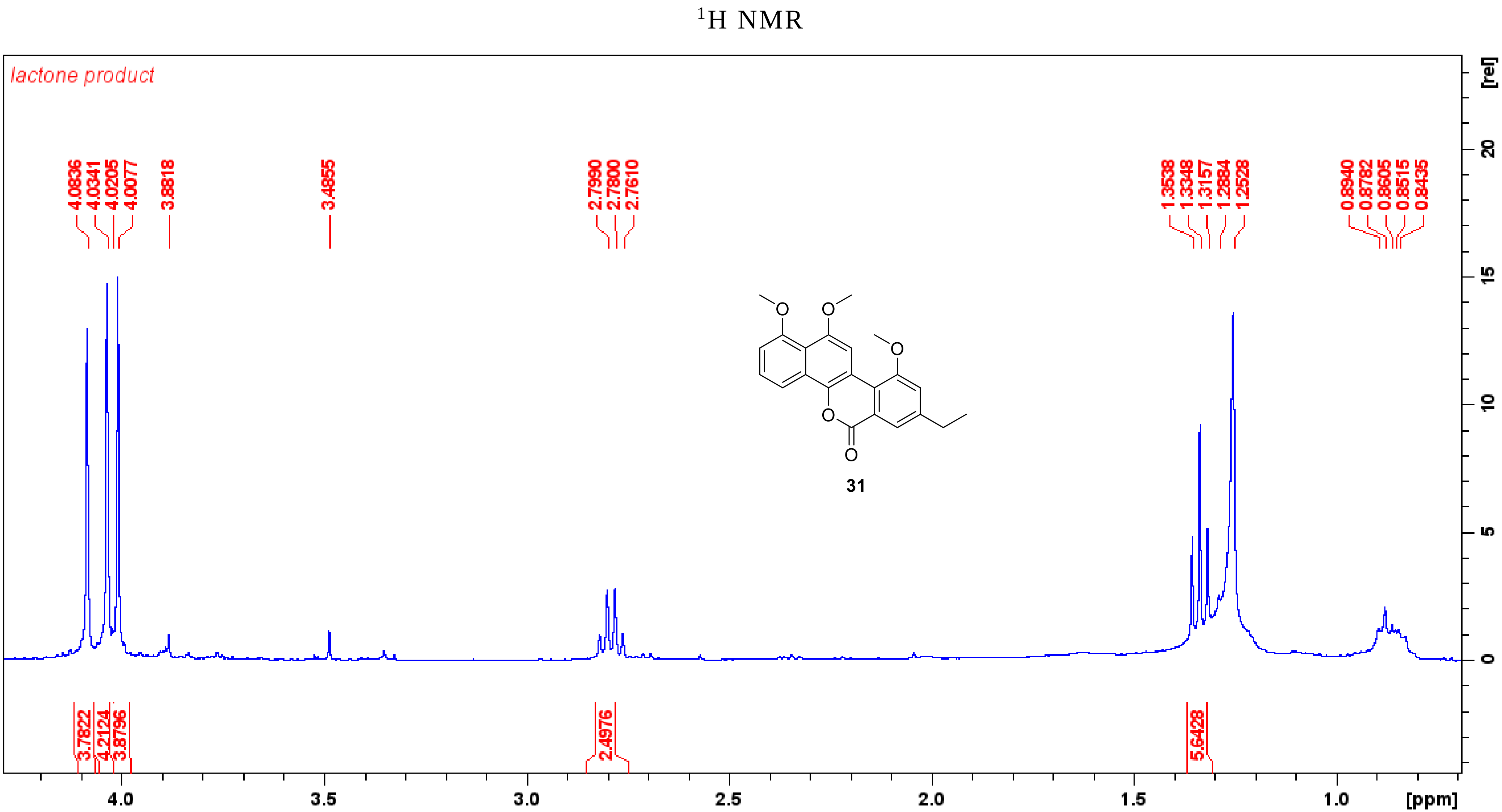

Figure S111. Zoomed in ^{1}H NMR spectrum of compound **31**.

13C NMR

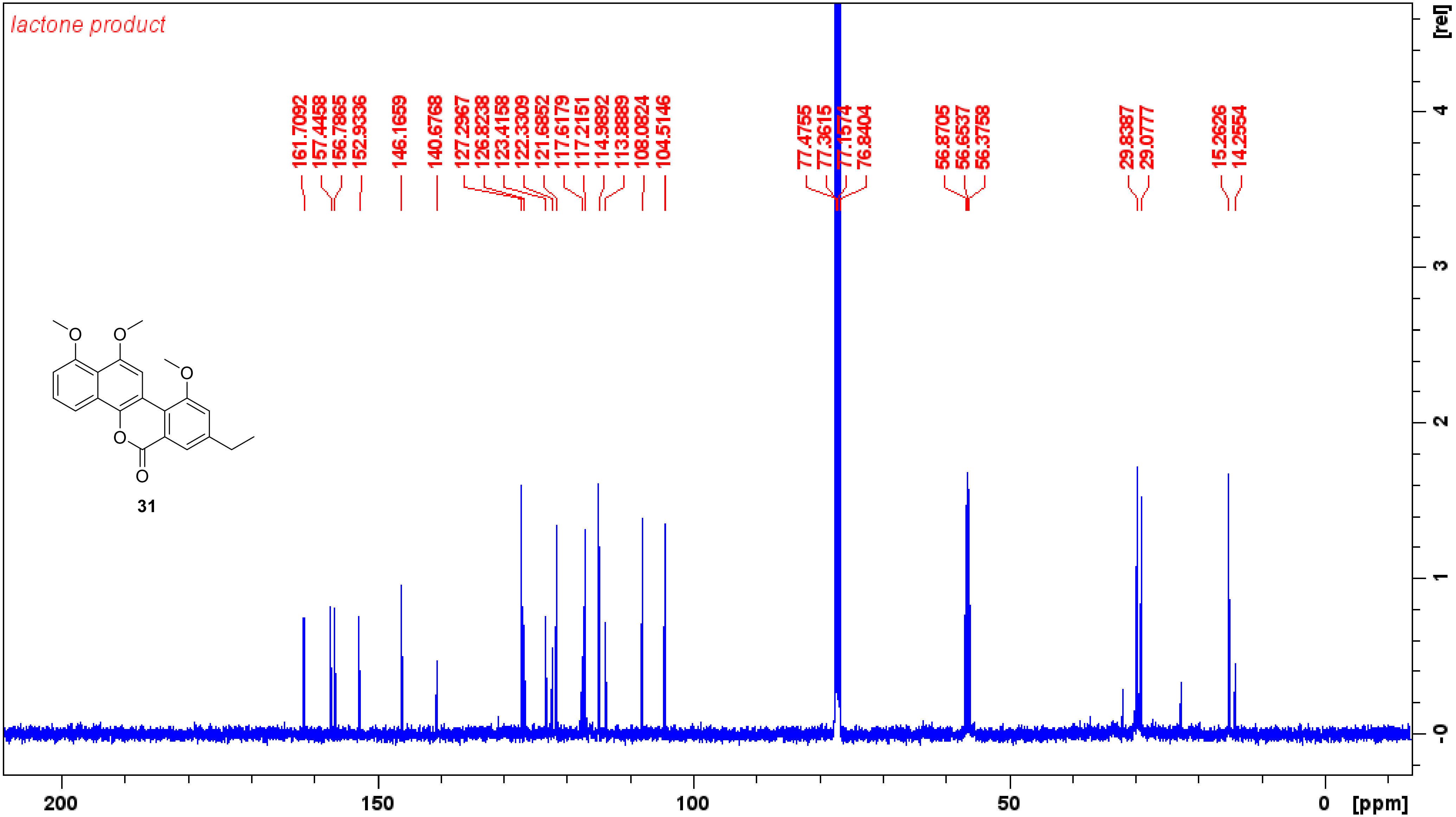

Figure S112. ^{13}C NMR spectrum of compound **31**.

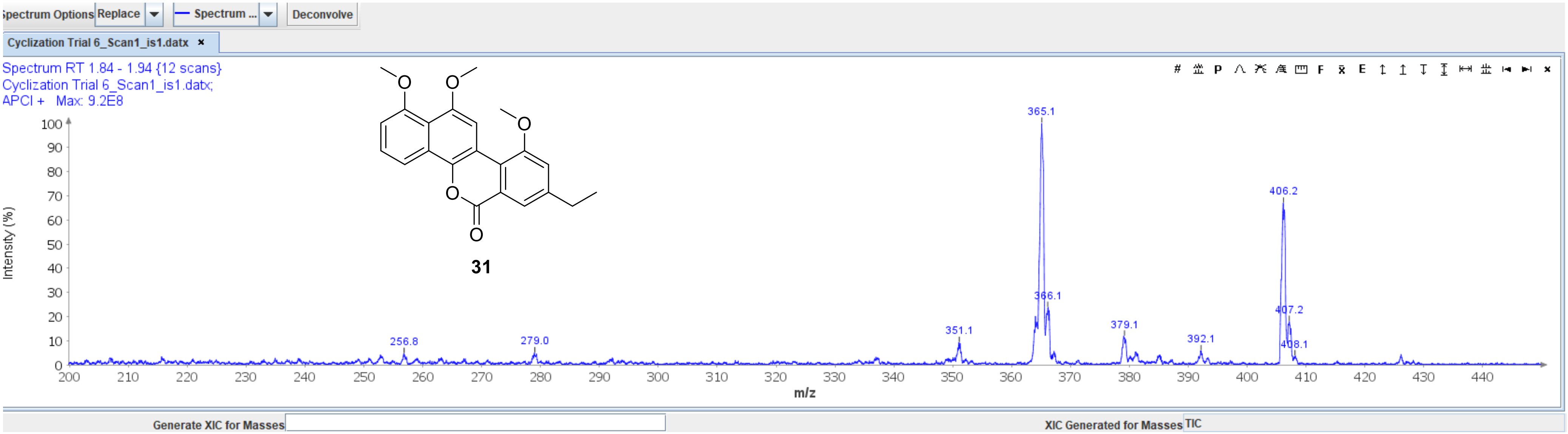

Figure S113. APCI-MS spectrum of compound **31**. Polarity: Positive

ECD Raw Data and KK Transform

The inability to acquire reliable optical rotation measurements on the Rudolph polarimeter, which we attribute to homoseongomycin's absorbance in the visible region (λ_{max} = 514 nm), led us to pursue the KK transform of ECD spectra into ORD spectra (Eq. 1).[37]

$$[\phi(\lambda)] = \frac{2}{\pi}\int_0^{\infty} [\theta(\mu)] \frac{\mu}{\lambda^2 - \mu^2} d\mu \qquad \text{Eq. (1)}$$

where $[\theta(\mu)]$ is the molar ellipticity (in units of: deg·L·mol^{-1}·cm^{-1}) as a function of wavelength μ obtained from the CD spectrometer, and $[\varphi(\lambda)]$ is the molar rotation as a function of wavelength λ for the calculated ORD spectrum.

The Jasco J-1500 CD Spectrometer SpectraManager software comes equipped with a KK transform function, which was used to produce the ORD spectrum above on page 246 of the SI and tabulate optical rotation values of homoseongomycin isomers at different wavelengths. To further validate the optical rotation of both homoseongomycin enantiomers, we also carried out a manual KK transform using the Ohta-Ishida analytical method (Eq. 2).[37]

$$[\phi(\lambda)] \approx \frac{2}{\pi}(2h)\left(\frac{1}{2}\right)\sum_{j}^{\#}\left[\frac{[\theta(\mu_j)]}{\lambda - \mu_j} - \frac{[\theta(\mu_j)]}{\lambda + \mu_j}\right] \qquad \text{Eq. (2)}$$

where j = 1, 2, 3, ..., N and corresponds to the measured wavelengths rounded to the nearest whole number, and $h = \frac{wavelength\ range}{\#\ of\ intervals}$. The summation of j to #, signifies to use alternate data points to avoid singularity of $\lambda = \mu$. For example, if λ is an odd number, use alternating j that only includes μ as an even number. Likewise, if λ is an even number, use alternating j that only includes μ as an odd number.

Ellipticity, in units of millidegrees (mdeg), as a function of wavelength ($\theta(\mu)$) is converted to molar ellipticity by (Eq. 3):

$$[\theta(\mu)] = \frac{\frac{\theta}{1000}}{cl} \qquad \text{Eq. (3)}$$

where c is the molar concentration (mol/L) and l is the path length (cm). After conversion from molar ellipticity to molar rotation, specific rotation ($\alpha(\lambda)$) (in units of: deg·mL·g^{-1}·dm^{-1}) is then calculated by (Eq. 4):

$$\alpha(\lambda) = \frac{[\phi(\lambda)]}{M} \times \frac{1000\ mL}{L} \times \frac{1\ cm}{0.1\ dm} \qquad \text{Eq. (4)}$$

where *M* is the molar mass (g/mol).

Table S16. Raw ECD data for *L*- and *D*-homoseongomycin

Constants		
concentration of sample for experimental ECD (mol/L)	cell path (cm)	molecular weight (g/mol)
0.00149	0.1	467.5

wavelength (nm)	*L*-homoseongomycin θ (mdeg)	*D*-homoseongomycin θ (mdeg)	*L*-homoseongomycin HT voltage (V)	*D*-homoseongomycin HT voltage (V)
600	-0.575655	0.684509	396.949	394.741
599	-0.576724	0.65263	397.943	394.808
598	-0.562433	0.616047	398.848	394.806
597	-0.544875	0.587679	399.503	394.602
596	-0.526138	0.556178	399.938	394.199
595	-0.531226	0.518724	400.268	393.715
594	-0.541537	0.491688	400.746	393.247
593	-0.555722	0.47262	401.218	392.838
592	-0.559801	0.465136	401.696	392.299
591	-0.568587	0.439885	402.083	391.68
590	-0.548297	0.409613	402.743	391.221
589	-0.534024	0.392781	403.511	390.996
588	-0.513382	0.392437	404.282	390.831
587	-0.498479	0.396292	405.017	390.54
586	-0.495863	0.392449	405.475	390.035
585	-0.482817	0.409727	406.194	389.745
584	-0.494058	0.427857	406.891	389.477
583	-0.500136	0.435925	407.806	389.403
582	-0.51269	0.433974	408.515	389.239
581	-0.531721	0.441159	409.384	389.079
580	-0.529255	0.452983	410.114	388.796

wavelength (nm)	*L*-homoseongomycin θ (mdeg)	*D*-homoseongomycin θ (mdeg)	*L*-homoseongomycin HT voltage (V)	*D*-homoseongomycin HT voltage (V)
579	-0.531752	0.467545	410.782	388.44
578	-0.520158	0.482235	411.406	388.144
577	-0.514952	0.484571	412.071	387.9
576	-0.534853	0.487047	412.755	387.719
575	-0.570152	0.476739	413.504	387.607
574	-0.595277	0.454462	414.105	387.33
573	-0.611322	0.427131	414.736	387.095
572	-0.599961	0.405776	415.566	386.868
571	-0.597433	0.403605	416.338	386.586
570	-0.600955	0.380475	417.017	386.348
569	-0.582632	0.369401	417.694	386.123
568	-0.576129	0.361503	418.347	385.915
567	-0.557765	0.351685	419.121	385.825
566	-0.549904	0.334391	419.846	385.714
565	-0.550189	0.299647	420.494	385.617
564	-0.514271	0.271781	420.977	385.378
563	-0.483891	0.241197	421.48	385.155
562	-0.43948	0.20436	422.087	384.941
561	-0.39466	0.165117	422.499	384.603
560	-0.34379	0.14341	422.925	384.304
559	-0.296711	0.124371	423.455	384.216
558	-0.268381	0.113081	423.929	384.054
557	-0.252688	0.0831862	424.465	383.945
556	-0.244239	0.0466064	424.817	383.703
555	-0.238914	0.0371965	425.267	383.511
554	-0.245707	0.0231385	425.572	383.203
553	-0.245059	0.0222972	425.973	383.018
552	-0.2519	0.0298498	426.309	382.792
551	-0.243565	0.0245578	426.532	382.538
550	-0.229352	0.0311831	426.743	382.259
549	-0.247217	0.0187771	426.938	381.98
548	-0.264041	0.00631708	427.197	381.794
547	-0.314407	0.00951398	427.376	381.426
546	-0.33717	0.0138733	427.581	381.149
545	-0.372802	0.020757	427.672	380.748
544	-0.407842	0.0173816	427.764	380.451
543	-0.408009	0.0113961	427.78	380.149
542	-0.395209	0.0070906	427.679	379.774
541	-0.370177	0.000641029	427.686	379.465
540	-0.372472	-0.0110771	427.713	379.15

wavelength (nm)	*L*-homoseongomycin θ (mdeg)	*D*-homoseongomycin θ (mdeg)	*L*-homoseongomycin HT voltage (V)	*D*-homoseongomycin HT voltage (V)
539	-0.359506	-0.0156349	427.698	378.856
538	-0.361294	-0.0211373	427.394	378.474
537	-0.349997	-0.0436101	426.878	377.962
536	-0.359399	-0.0503186	426.378	377.525
535	-0.384397	-0.049554	425.729	376.996
534	-0.401472	-0.0437791	425.1	376.56
533	-0.410502	-0.0365655	424.383	376.113
532	-0.387666	-0.0214722	423.671	375.641
531	-0.346378	0.00508413	423.041	375.264
530	-0.311341	0.0226423	422.275	374.752
529	-0.295744	0.0420827	421.649	374.428
528	-0.307502	0.0558793	420.945	373.978
527	-0.3255	0.0655289	420.298	373.584
526	-0.354674	0.075851	419.628	373.134
525	-0.369853	0.0751411	419.042	372.815
524	-0.41172	0.0776698	418.392	372.459
523	-0.440154	0.0791875	417.704	372.085
522	-0.462185	0.0855169	417.015	371.708
521	-0.472305	0.0883445	416.358	371.349
520	-0.463747	0.0905541	415.745	371.006
519	-0.456002	0.0927141	415.156	370.668
518	-0.450099	0.0841881	414.614	370.418
517	-0.449	0.0562157	414.066	370.132
516	-0.473371	0.0441205	413.402	369.763
515	-0.481258	0.0315625	412.811	369.453
514	-0.486369	0.0188606	412.168	369.141
513	-0.495639	-0.00558499	411.647	368.95
512	-0.519573	-0.0268817	411.07	368.648
511	-0.524932	-0.0361248	410.571	368.411
510	-0.516924	-0.0271987	410.007	368.113
509	-0.468402	-0.0353737	409.288	367.761
508	-0.43063	-0.0466569	408.578	367.356
507	-0.404467	-0.0518293	407.822	366.894
506	-0.38169	-0.0599748	406.915	366.305
505	-0.351516	-0.0540473	406.002	365.727
504	-0.324077	-0.0593772	405.088	365.207
503	-0.312865	-0.05622	404.308	364.794
502	-0.316686	-0.0686241	403.615	364.464
501	-0.314935	-0.0959921	402.852	364.002
500	-0.308282	-0.112502	401.855	363.383

wavelength (nm)	*L*-homoseongomycin θ (mdeg)	*D*-homoseongomycin θ (mdeg)	*L*-homoseongomycin HT voltage (V)	*D*-homoseongomycin HT voltage (V)
499	-0.282488	-0.105841	400.674	362.677
498	-0.265484	-0.0858712	399.267	361.796
497	-0.26795	-0.0743514	397.716	360.79
496	-0.26799	-0.0718331	395.649	359.261
495	-0.249968	-0.076531	392.955	357.091
494	-0.23399	-0.0677393	389.733	354.542
493	-0.212743	-0.0510334	387.303	352.845
492	-0.194109	-0.0334509	387.259	353.262
491	-0.17545	-0.012653	388.341	354.636
490	-0.17752	-0.00743894	389.595	356.03
489	-0.185732	-0.00692077	389.424	356.142
488	-0.200661	-0.0185718	388.41	355.547
487	-0.215916	-0.00921277	386.873	354.475
486	-0.238242	-0.0152782	384.798	352.889
485	-0.272223	-0.0206015	382.056	350.702
484	-0.291314	-0.0236425	379.39	348.617
483	-0.295695	-0.0251172	376.948	346.793
482	-0.257636	-0.0071127	375.783	346.329
481	-0.228388	8.16E-06	376.27	347.221
480	-0.222773	-0.00350682	377.29	348.612
479	-0.243909	-0.00805938	378.211	349.742
478	-0.29113	-0.00525188	377.746	349.665
477	-0.341863	-0.00409641	376.147	348.567
476	-0.391254	-0.00544845	373.729	346.625
475	-0.399565	-0.0116584	369.163	342.57
474	-0.392086	-0.000498126	364.34	338.903
473	-0.370528	0.0159778	361.707	336.983
472	-0.358507	0.0227926	359.938	336.1
471	-0.362694	0.0404223	358.88	335.235
470	-0.365836	0.0587498	354.617	331.529
469	-0.389507	0.0694655	350.77	328.502
468	-0.418176	0.0788186	350.077	328.387
467	-0.46586	0.0717934	351.569	330.355
466	-0.50325	0.0808428	353.822	332.739
465	-0.525533	0.095756	354.102	333.225
464	-0.535363	0.102921	352.693	332.288
463	-0.531554	0.102032	351.768	331.897
462	-0.533335	0.0974412	352.043	332.665
461	-0.5201	0.106952	352.818	333.745
460	-0.527442	0.124304	352.516	333.789

wavelength (nm)	*L*-homoseongomycin θ (mdeg)	*D*-homoseongomycin θ (mdeg)	*L*-homoseongomycin HT voltage (V)	*D*-homoseongomycin HT voltage (V)
459	-0.54324	0.139295	351.533	333.284
458	-0.553917	0.157537	350.917	333.081
457	-0.552656	0.187648	350.91	333.451
456	-0.532994	0.207759	351.125	333.958
455	-0.520057	0.217755	350.731	333.884
454	-0.504088	0.22168	348.781	332.302
453	-0.483033	0.222504	347.019	331.048
452	-0.459128	0.240765	344.107	328.472
451	-0.448659	0.260789	342.096	327.195
450	-0.45722	0.287748	342.544	327.978
449	-0.461866	0.315098	343.184	329.056
448	-0.465836	0.328413	345.367	331.474
447	-0.475326	0.3427	345.442	331.72
446	-0.488424	0.352969	344.524	331.231
445	-0.513493	0.367322	343.439	330.593
444	-0.535585	0.37998	342.295	329.945
443	-0.571781	0.39475	341.277	329.357
442	-0.604454	0.41201	340.371	328.824
441	-0.635379	0.434149	339.442	328.197
440	-0.659098	0.458647	338.35	327.408
439	-0.682027	0.477119	337.303	326.781
438	-0.718236	0.49275	336.662	326.481
437	-0.745633	0.504819	336.42	326.532
436	-0.76486	0.511512	336.247	326.608
435	-0.781025	0.514813	335.814	326.43
434	-0.811226	0.520619	335.234	326.132
433	-0.841481	0.540391	334.608	325.759
432	-0.864805	0.572541	334	325.38
431	-0.885777	0.5937	333.353	324.992
430	-0.897624	0.608044	332.726	324.592
429	-0.912869	0.626654	332.12	324.178
428	-0.925227	0.645881	331.52	323.769
427	-0.937899	0.663238	330.913	323.334
426	-0.953586	0.686972	330.251	322.944
425	-0.96404	0.709616	329.637	322.581
424	-0.979973	0.74532	329.041	322.229
423	-0.979894	0.781947	328.342	321.775
422	-0.985018	0.824387	327.403	321.038
421	-0.982691	0.85982	326.209	320.084
420	-0.970652	0.89405	325.444	319.619

wavelength (nm)	*L*-homoseongomycin θ (mdeg)	*D*-homoseongomycin θ (mdeg)	*L*-homoseongomycin HT voltage (V)	*D*-homoseongomycin HT voltage (V)
419	-0.961377	0.924942	325.198	319.653
418	-0.944309	0.941061	325.415	320.128
417	-0.925281	0.945169	325.409	320.363
416	-0.900249	0.947745	325.014	320.21
415	-0.881022	0.958198	324.504	319.929
414	-0.871233	0.966176	323.879	319.53
413	-0.856124	0.970869	323.344	319.26
412	-0.835309	0.964578	322.952	319.1
411	-0.816124	0.955334	322.668	319.062
410	-0.787209	0.95361	322.431	319.035
409	-0.767212	0.942818	322.231	319.054
408	-0.745169	0.928207	322.036	319.144
407	-0.723937	0.901944	321.861	319.214
406	-0.695027	0.873455	321.615	319.195
405	-0.669642	0.848207	321.364	319.123
404	-0.642986	0.817979	321.099	319.063
403	-0.617712	0.800647	320.711	318.924
402	-0.576898	0.783147	320.186	318.701
401	-0.524375	0.770217	319.551	318.326
400	-0.467783	0.747935	318.848	317.917
399	-0.392201	0.717662	318.228	317.592
398	-0.336459	0.680726	317.718	317.401
397	-0.321933	0.64437	317.538	317.552
396	-0.266192	0.599211	317.621	317.964
395	-0.115601	0.550097	317.861	318.495
394	0.0878788	0.490864	318.089	319.051
393	0.134855	0.431818	318.225	319.521
392	0.23434	0.360472	318.308	319.968
391	0.357254	0.308633	318.425	320.417
390	0.480285	0.243862	318.607	320.897
389	0.574992	0.172792	318.753	321.379
388	0.639297	0.108206	318.861	321.84
387	0.694209	0.0180419	318.871	322.201
386	0.765	-0.0544634	318.833	322.504
385	0.856356	-0.151653	318.773	322.763
384	0.932775	-0.200482	318.593	322.955
383	1.00815	-0.277767	318.475	323.174
382	1.07945	-0.351033	318.37	323.371
381	1.14073	-0.417157	318.332	323.618
380	1.20019	-0.49511	318.288	323.9

wavelength (nm)	*L*-homoseongomycin θ (mdeg)	*D*-homoseongomycin θ (mdeg)	*L*-homoseongomycin HT voltage (V)	*D*-homoseongomycin HT voltage (V)
379	1.26546	-0.563171	318.264	324.207
378	1.33234	-0.631168	318.245	324.542
377	1.39186	-0.677032	318.223	324.896
376	1.44573	-0.732592	318.187	325.207
375	1.48347	-0.799358	318.14	325.552
374	1.50865	-0.852275	318.078	325.834
373	1.53143	-0.893708	317.981	326.133
372	1.54968	-0.919596	317.857	326.393
371	1.58476	-0.954424	317.78	326.722
370	1.61984	-0.995874	317.762	327.048
369	1.6412	-1.02819	317.793	327.427
368	1.64407	-1.05228	317.846	327.809
367	1.64139	-1.07216	317.9	328.185
366	1.64138	-1.09055	318.026	328.573
365	1.64261	-1.10313	318.143	328.972
364	1.63136	-1.11696	318.299	329.383
363	1.6079	-1.12702	318.459	329.863
362	1.5806	-1.14671	318.618	330.229
361	1.56191	-1.16674	318.829	330.678
360	1.55073	-1.18512	319.065	331.069
359	1.54234	-1.21482	319.404	331.512
358	1.53695	-1.24548	319.683	331.919
357	1.52538	-1.27944	320.032	332.304
356	1.49667	-1.29657	320.353	332.68
355	1.4545	-1.29866	320.799	333.035
354	1.41653	-1.31655	321.257	333.395
353	1.39187	-1.32859	321.78	333.753
352	1.36144	-1.33814	322.329	334.078
351	1.33292	-1.33904	322.929	334.426
350	1.30279	-1.33952	323.602	334.744
349	1.27854	-1.33895	324.31	334.998
348	1.25506	-1.33027	325.052	335.212
347	1.22627	-1.32078	325.869	335.41
346	1.18547	-1.30981	326.738	335.554
345	1.15097	-1.31107	327.634	335.646
344	1.12239	-1.3011	328.549	335.715
343	1.08851	-1.30438	329.45	335.786
342	1.06357	-1.30793	330.391	335.892
341	1.03829	-1.3168	331.309	335.94
340	1.01844	-1.32041	332.178	336.007

wavelength (nm)	*L*-homoseongomycin θ (mdeg)	*D*-homoseongomycin θ (mdeg)	*L*-homoseongomycin HT voltage (V)	*D*-homoseongomycin HT voltage (V)
339	0.999573	-1.31527	332.986	336.085
338	0.970578	-1.31296	333.704	336.108
337	0.961575	-1.32358	334.244	336.142
336	0.942137	-1.3371	334.647	336.033
335	0.911662	-1.34219	334.905	335.971
334	0.8814	-1.34765	335.028	335.866
333	0.870497	-1.35625	335.093	335.782
332	0.85809	-1.36675	335.148	335.698
331	0.841233	-1.38574	335.233	335.656
330	0.807944	-1.38533	335.391	335.655
329	0.780847	-1.39241	335.591	335.696
328	0.761787	-1.37659	335.91	335.829
327	0.740512	-1.36039	336.29	335.963
326	0.723366	-1.34344	336.751	336.152
325	0.710045	-1.32775	337.107	336.25
324	0.70688	-1.31835	337.525	336.437
323	0.708554	-1.30158	338.015	336.825
322	0.705274	-1.2775	338.615	337.38
321	0.696912	-1.25303	339.278	338.116
320	0.696766	-1.23252	339.848	338.852
319	0.694177	-1.21429	340.408	339.651
318	0.686683	-1.19628	341.016	340.552
317	0.680499	-1.16773	341.717	341.561
316	0.679488	-1.11656	342.519	342.735
315	0.689261	-1.06517	343.448	344.095
314	0.688636	-0.98996	344.673	345.778
313	0.695755	-0.931491	346.226	347.899
312	0.700849	-0.868151	348.218	350.484
311	0.690554	-0.81354	350.492	353.446
310	0.66929	-0.767536	352.922	356.6
309	0.625564	-0.663833	355.455	359.888
308	0.599241	-0.595044	358.229	363.418
307	0.557941	-0.374598	361.201	367.005
306	0.519135	-0.402209	364.428	370.647
305	0.472103	-0.294123	367.912	374.191
304	0.414895	-0.151261	371.817	377.684
303	0.347734	-0.0477672	376.13	380.989
302	0.233324	0.122997	380.925	384.151
301	0.280243	0.261556	386.353	387.281
300	0.211598	0.433294	392.619	390.547

wavelength (nm)	*L*-homoseongomycin θ (mdeg)	*D*-homoseongomycin θ (mdeg)	*L*-homoseongomycin HT voltage (V)	*D*-homoseongomycin HT voltage (V)
299	0.132315	0.593519	400.108	394.13
298	0.0842881	0.639198	408.439	397.903
297	-0.00638939	0.768723	418.173	402.097
296	-0.0574113	0.831639	429.744	406.846
295	-0.315553	1.04457	443.244	412.32
294	-0.154931	1.16503	457.849	418.163
293	-0.0435193	1.23829	472.6	423.936
292	-0.0389056	1.25361	488.26	430.008
291	0.673374	1.22096	504.546	436.493
290	0.49602	1.23112	519.89	442.803
289	-0.310284	1.20944	533.09	448.576
288	-0.790358	1.29638	543.312	453.286
287	-1.05762	1.18956	550.724	457.084
286	-1.01163	1.3704	555.189	459.929
285	-0.53311	1.15668	557.182	462.001
284	-0.0421396	0.862366	557.243	463.362
283	-0.268999	0.747703	556.084	464.266
282	-0.0559306	0.391194	554.223	464.848
281	0.0974352	0.164823	551.997	465.151
280	-0.0657768	0.0233276	549.469	465.271
279	-0.635868	-0.0350152	546.452	465.247
278	-0.396456	-0.239446	543.005	464.9
277	-0.910837	-0.286662	539.042	464.217
276	-0.0972887	-0.631926	534.505	463.189
275	-0.509239	-0.553823	529.382	461.849
274	0.0168264	-0.587081	524.24	460.398
273	0.212615	-0.647677	518.986	458.756
272	0.541374	-0.794306	514.135	457.146
271	0.274242	-1.0447	509.491	455.554
270	0.0636311	-1.44556	505.06	454.174
269	0.272665	-1.79231	500.782	453.054
268	0.430741	-2.01669	496.684	452.205
267	0.710773	-2.05947	492.68	451.54
266	1.0522	-2.25259	488.709	450.953
265	1.25506	-2.41674	484.99	450.395
264	1.0421	-2.44395	481.601	449.727
263	0.863866	-2.42738	478.53	449.128
262	0.940873	-2.41917	475.923	448.768
261	1.17201	-2.47641	473.52	448.87
260	1.80818	-2.52259	471.32	449.77

wavelength (nm)	*L*-homoseongomycin θ (mdeg)	*D*-homoseongomycin θ (mdeg)	*L*-homoseongomycin HT voltage (V)	*D*-homoseongomycin HT voltage (V)
259	1.80002	-2.55696	469.583	451.596
258	2.15118	-2.5903	468.009	454.419
257	2.29542	-2.59403	466.572	458.165
256	2.5284	-2.65692	464.91	462.977
255	2.59312	-2.68796	463.002	469.006
254	3.05273	-2.73878	460.909	475.705
253	3.29371	-2.80134	458.582	482.864
252	3.62427	-2.72712	455.928	490.987
251	3.41312	-2.85903	453.064	499.235
250	3.40147	-2.73535	449.87	508.496
249	3.43745	-3.33083	446.913	516.988
248	3.68249	-3.31974	444.414	524.672
247	3.72283	-3.61415	441.904	533.285
246	3.90581	-3.67897	439.773	541.599
245	4.4014	-3.10821	438.01	550.086
244	4.46595	-3.44838	436.767	557.92
243	4.76836	-3.54137	436.105	565.265
242	4.74928	-3.62485	435.983	571.767
241	4.95543	-4.25607	436.42	577.545
240	5.07542	-4.03592	437.67	583.44
239	5.27669	-4.97075	439.905	588.698
238	5.37457	-5.71739	442.498	592.592
237	5.29163	-6.13851	445.602	596.102
236	5.2247	-5.46933	449.547	599.306
235	5.28853	-5.59267	454.291	602.143
234	5.33015	-5.19471	459.792	604.738
233	5.40692	-6.54795	465.841	607.092
232	5.44799	-6.78792	471.096	608.76
231	5.46189	-6.57701	477.676	610.276
230	5.41441	-6.41958	484.659	611.727
229	5.45533	-6.24454	491.733	613.365
228	5.67271	-6.36231	499.123	615.59
227	5.91454	-7.85999	506.341	618.406
226	5.67204	-8.8866	514.029	622.253
225	5.72339	-7.40937	521.286	626.65
224	5.84317	-7.30157	529.872	632.649
223	6.19245	-7.75685	538.637	639.298
222	6.08754	-8.38409	548.269	647.032
221	6.42384	-9.10371	558.459	655.69

wavelength (nm)	*L*-homoseongomycin θ (mdeg)	*D*-homoseongomycin θ (mdeg)	*L*-homoseongomycin HT voltage (V)	*D*-homoseongomycin HT voltage (V)
220	6.59826	-7.86591	569.588	666.267

Table S17. KK Transform data of *L*-homoseongomycin.

Experimental ECD (mdeg)	wavelength (μ)	molar Ellipticity (θ(μ))	λ for ORD	h	(2/π)(2h)(1/2)	Σ[(θ(μ)/(λ-μ))-(θ(μ)/(λ+μ))]	Molar Rotation (φ(λ))	Specific Rotation [α(λ)]
-0.575655	600	-3.86	599	1.98	1.26	-5.21	-6.60	-141.20
-0.562433	598	-3.77	597			-3.74	-4.73	-101.28
-0.526138	596	-3.53	595			-3.28	-4.15	-88.84
-0.541537	594	-3.63	593			-2.71	-3.44	-73.56
-0.559801	592	-3.76	591			-1.97	-2.50	-53.43
-0.548297	590	-3.68	589			-1.37	-1.74	-37.18
-0.513382	588	-3.45	587			-1.19	-1.50	-32.12
-0.495863	586	-3.33	585			-1.12	-1.42	-30.48
-0.494058	584	-3.32	583			-1.10	-1.40	-29.91
-0.51269	582	-3.44	581			-0.89	-1.13	-24.17
-0.529255	580	-3.55	579			-0.53	-0.67	-14.27
-0.520158	578	-3.49	577			-0.48	-0.61	-13.11
-0.534853	576	-3.59	575			-0.48	-0.61	-13.06
-0.595277	574	-4.00	573			0.27	0.34	7.35
-0.599961	572	-4.03	571			0.85	1.08	23.13
-0.600955	570	-4.03	569			1.54	1.96	41.85
-0.576129	568	-3.87	567			2.07	2.62	56.02
-0.549904	566	-3.69	565			2.60	3.30	70.57
-0.514271	564	-3.45	563			3.24	4.10	87.66
-0.43948	562	-2.95	561			3.54	4.48	95.93
-0.34379	560	-2.31	559			3.28	4.16	88.96
-0.268381	558	-1.80	557			2.62	3.32	70.93
-0.244239	556	-1.64	555			2.06	2.61	55.86
-0.245707	554	-1.65	553			1.71	2.17	46.43
-0.2519	552	-1.69	551			1.56	1.97	42.14
-0.229352	550	-1.54	549			0.87	1.10	23.55

Experimental ECD (mdeg)	wavelength (μ)	molar Ellipticity (θ(μ))	λ for ORD	h	(2/π)(2h)(1/2)	Σ[(θ(μ)/(λ-μ))-(θ(μ)/(λ+μ))]	Molar Rotation (φ(λ))	Specific Rotation [α(λ)]
-0.264041	548	-1.77	547			0.39	0.50	10.64
-0.33717	546	-2.26	545			0.48	0.61	13.13
-0.407842	544	-2.74	543			1.27	1.61	34.35
-0.395209	542	-2.65	541			1.61	2.04	43.61
-0.372472	540	-2.50	539			1.66	2.10	44.92
-0.361294	538	-2.42	537			1.61	2.04	43.73
-0.359399	536	-2.41	535			1.44	1.82	39.00
-0.401472	534	-2.69	533			1.96	2.48	53.04
-0.387666	532	-2.60	531			2.42	3.06	65.47
-0.311341	530	-2.09	529			1.82	2.31	49.35
-0.307502	528	-2.06	527			1.23	1.56	33.44
-0.354674	526	-2.38	525			1.07	1.35	28.86
-0.41172	524	-2.76	523			1.26	1.59	34.10
-0.462185	522	-3.10	521			1.85	2.34	50.09
-0.463747	520	-3.11	519			2.22	2.81	60.13
-0.450099	518	-3.02	517			2.22	2.82	60.23
-0.473371	516	-3.18	515			2.51	3.18	68.04
-0.486369	514	-3.26	513			2.77	3.51	75.06
-0.519573	512	-3.49	511			3.51	4.45	95.19
-0.516924	510	-3.47	509			4.50	5.70	122.03
-0.43063	508	-2.89	507			4.59	5.81	124.23
-0.38169	506	-2.56	505			4.65	5.89	126.07
-0.324077	504	-2.18	503			4.29	5.43	116.25
-0.316686	502	-2.13	501			4.24	5.36	114.75
-0.308282	500	-2.07	499			4.39	5.55	118.81
-0.265484	498	-1.78	497			4.07	5.15	110.24
-0.26799	496	-1.80	495			4.19	5.30	113.42
-0.23399	494	-1.57	493			4.06	5.14	109.99
-0.194109	492	-1.30	491			3.60	4.56	97.56
-0.17752	490	-1.19	489			2.99	3.78	80.88
-0.200661	488	-1.35	487			2.60	3.29	70.42
-0.238242	486	-1.60	485			2.44	3.08	65.98
-0.291314	484	-1.96	483			2.95	3.74	79.98
-0.257636	482	-1.73	481			2.81	3.55	76.03
-0.222773	480	-1.50	479			1.89	2.39	51.16
-0.29113	478	-1.95	477			1.60	2.03	43.33
-0.391254	476	-2.63	475			2.38	3.02	64.50
-0.392086	474	-2.63	473			2.78	3.52	75.35
-0.358507	472	-2.41	471			2.50	3.17	67.79

Experimental ECD (mdeg)	wavelength (μ)	molar Ellipticity (θ(μ))	λ for ORD	h	(2/π)(2h)(1/2)	Σ[(θ(μ)/(λ-μ))-(θ(μ)/(λ+μ))]	Molar Rotation (φ(λ))	Specific Rotation [α(λ)]
-0.365836	470	-2.46	469			2.10	2.66	56.98
-0.418176	468	-2.81	467			1.97	2.50	53.38
-0.50325	466	-3.38	465			2.59	3.29	70.29
-0.535363	464	-3.59	463			3.23	4.09	87.44
-0.533335	462	-3.58	461			3.60	4.57	97.67
-0.527442	460	-3.54	459			3.75	4.75	101.55
-0.553917	458	-3.72	457			4.36	5.52	118.11
-0.532994	456	-3.58	455			4.70	5.96	127.46
-0.504088	454	-3.38	453			4.89	6.20	132.56
-0.459128	452	-3.08	451			4.57	5.79	123.75
-0.45722	450	-3.07	449			4.33	5.49	117.35
-0.465836	448	-3.13	447			4.07	5.16	110.32
-0.488424	446	-3.28	445			3.78	4.79	102.50
-0.535585	444	-3.59	443			3.63	4.60	98.47
-0.604454	442	-4.06	441			3.85	4.88	104.44
-0.659098	440	-4.42	439			4.12	5.22	111.65
-0.718236	438	-4.82	437			4.58	5.81	124.20
-0.76486	436	-5.13	435			5.06	6.41	137.11
-0.811226	434	-5.44	433			5.59	7.08	151.39
-0.864805	432	-5.80	431			6.37	8.07	172.68
-0.897624	430	-6.02	429			7.14	9.05	193.50
-0.925227	428	-6.21	427			7.92	10.04	214.69
-0.953586	426	-6.40	425			8.81	11.16	238.70
-0.979973	424	-6.58	423			9.90	12.54	268.31
-0.985018	422	-6.61	421			11.04	13.98	298.97
-0.970652	420	-6.51	419			12.11	15.34	328.18
-0.944309	418	-6.34	417			13.16	16.67	356.55
-0.900249	416	-6.04	415			13.97	17.69	378.38
-0.871233	414	-5.85	413			14.88	18.85	403.21
-0.835309	412	-5.61	411			15.84	20.06	429.12
-0.787209	410	-5.28	409			16.70	21.15	452.35
-0.745169	408	-5.00	407			17.66	22.36	478.37
-0.695027	406	-4.66	405			18.65	23.62	505.20
-0.642986	404	-4.32	403			19.80	25.07	536.33
-0.576898	402	-3.87	401			21.16	26.80	573.16
-0.467783	400	-3.14	399			22.33	28.28	604.95
-0.336459	398	-2.26	397			23.13	29.30	626.75
-0.266192	396	-1.79	395			25.63	32.47	694.45
0.0878788	394	0.59	393			24.90	31.54	674.68

Experimental ECD (mdeg)	wavelength (μ)	molar Ellipticity (θ(μ))	λ for ORD	h	(2/π)(2h)(1/2)	Σ[(θ(μ)/(λ-μ))-(θ(μ)/(λ+μ))]	Molar Rotation (φ(λ))	Specific Rotation [α(λ)]
0.23434	392	1.57	391			25.32	32.07	686.05
0.480285	390	3.22	389			24.46	30.97	662.55
0.639297	388	4.29	387			23.70	30.02	642.11
0.765	386	5.13	385			23.34	29.56	632.25
0.932775	384	6.26	383			22.47	28.47	608.90
1.07945	382	7.24	381			21.41	27.12	580.14
1.20019	380	8.05	379			20.43	25.88	553.57
1.33234	378	8.94	377			19.11	24.20	517.74
1.44573	376	9.70	375			17.47	22.13	473.34
1.50865	374	10.13	373			16.01	20.28	433.75
1.54968	372	10.40	371			14.85	18.81	402.27
1.61984	370	10.87	369			13.20	16.72	357.75
1.64407	368	11.03	367			11.64	14.75	315.46
1.64138	366	11.02	365			10.26	13.00	278.03
1.63136	364	10.95	363			8.83	11.18	239.21
1.5806	362	10.61	361			7.91	10.02	214.25
1.55073	360	10.41	359			7.04	8.92	190.86
1.53695	358	10.32	357			5.92	7.50	160.40
1.49667	356	10.04	355			4.80	6.08	130.00
1.41653	354	9.51	353			4.21	5.34	114.13
1.36144	352	9.14	351			3.61	4.58	97.89
1.30279	350	8.74	349			3.15	3.99	85.26
1.25506	348	8.42	347			2.56	3.25	69.49
1.18547	346	7.96	345			2.24	2.84	60.70
1.12239	344	7.53	343			2.03	2.57	54.87
1.06357	342	7.14	341			1.94	2.45	52.42
1.01844	340	6.84	339			1.80	2.28	48.77
0.970578	338	6.51	337			1.79	2.27	48.45
0.942137	336	6.32	335			1.52	1.93	41.19
0.8814	334	5.92	333			1.67	2.11	45.16
0.85809	332	5.76	331			1.49	1.89	40.40
0.807944	330	5.42	329			1.53	1.94	41.55
0.761787	328	5.11	327			1.70	2.16	46.12
0.723366	326	4.85	325			2.00	2.54	54.30
0.70688	324	4.74	323			2.24	2.83	60.59
0.705274	322	4.73	321			2.27	2.87	61.48
0.696766	320	4.68	319			2.28	2.88	61.68
0.686683	318	4.61	317			2.29	2.89	61.91
0.679488	316	4.56	315			2.30	2.92	62.42

Experimental ECD (mdeg)	wavelength (μ)	molar Ellipticity (θ(μ))	λ for ORD	h	(2/π)(2h)(1/2)	Σ[(θ(μ)/(λ-μ))-(θ(μ)/(λ+μ))]	Molar Rotation (φ(λ))	Specific Rotation [α(λ)]
0.688636	314	4.62	313			2.09	2.64	56.51
0.700849	312	4.70	311			1.43	1.81	38.77
0.66929	310	4.49	309			0.77	0.98	20.88
0.599241	308	4.02	307			0.37	0.47	9.96
0.519135	306	3.48	305			0.03	0.03	0.69
0.414895	304	2.78	303			-0.25	-0.32	-6.85
0.233324	302	1.57	301			1.08	1.36	29.14
0.211598	300	1.42	299			1.04	1.32	28.25
0.0842881	298	0.57	297			1.73	2.19	46.78
-0.0574113	296	-0.39	295			3.30	4.18	89.51
-0.154931	294	-1.04	293			5.99	7.59	162.37
-0.0389056	292	-0.26	291			7.87	9.97	213.21
0.49602	290	3.33	289			-2.65	-3.36	-71.88
-0.790358	288	-5.30	287			6.41	8.11	173.55
-1.01163	286	-6.79	285			17.51	22.18	474.51
-0.0421396	284	-0.28	283			13.63	17.27	369.34
-0.0559306	282	-0.38	281			13.61	17.23	368.62
-0.0657768	280	-0.44	279			13.23	16.75	358.37
-0.396456	278	-2.66	277			19.14	24.24	518.48
-0.0972887	276	-0.65	275			20.67	26.18	559.92
0.0168264	274	0.11	273			24.05	30.46	651.51
0.541374	272	3.63	271			19.64	24.87	531.99
0.0636311	270	0.43	269			26.54	33.62	719.11
0.430741	268	2.89	267			29.99	37.99	812.57
1.0522	266	7.06	265			26.97	34.16	730.74
1.0421	264	6.99	263			27.82	35.24	753.74
0.940873	262	6.31	261			35.21	44.59	953.85
1.80818	260	12.14	259			33.03	41.83	894.78
2.15118	258	14.44	257			32.63	41.33	884.02
2.5284	256	16.97	255			32.47	41.13	879.77
3.05273	254	20.49	253			30.27	38.33	820.00
3.62427	252	24.32	251			22.93	29.04	621.13
3.40147	250	22.83	249			23.71	30.04	642.48
3.68249	248	24.71	247			22.33	28.28	604.94
3.90581	246	26.21	245			22.36	28.33	605.92
4.46595	244	29.97	243			17.94	22.72	485.98
4.74928	242	31.87	241			14.30	18.11	387.31
5.07542	240	34.06	239			9.47	12.00	256.58
5.37457	238	36.07	237			2.07	2.63	56.19

Experimental ECD (mdeg)	wavelength (μ)	molar Ellipticity (θ(μ))	λ for ORD	h	(2/π)(2h)(1/2)	Σ[(θ(μ)/(λ-μ))-(θ(μ)/(λ+μ))]	Molar Rotation (φ(λ))	Specific Rotation [α(λ)]
5.2247	236	35.07	235			-0.90	-1.14	-24.40
5.33015	234	35.77	233			-4.99	-6.32	-135.11
5.44799	232	36.56	231			-10.20	-12.92	-276.47
5.41441	230	36.34	229			-13.43	-17.01	-363.94
5.67271	228	38.07	227			-20.52	-25.99	-555.97
5.67204	226	38.07	225			-26.51	-33.57	-718.14
5.84317	224	39.22	223			-35.41	-44.85	-959.39
6.08754	222	40.86	221			-50.25	-63.64	-1361.28
6.59826	220	44.28						

ORD

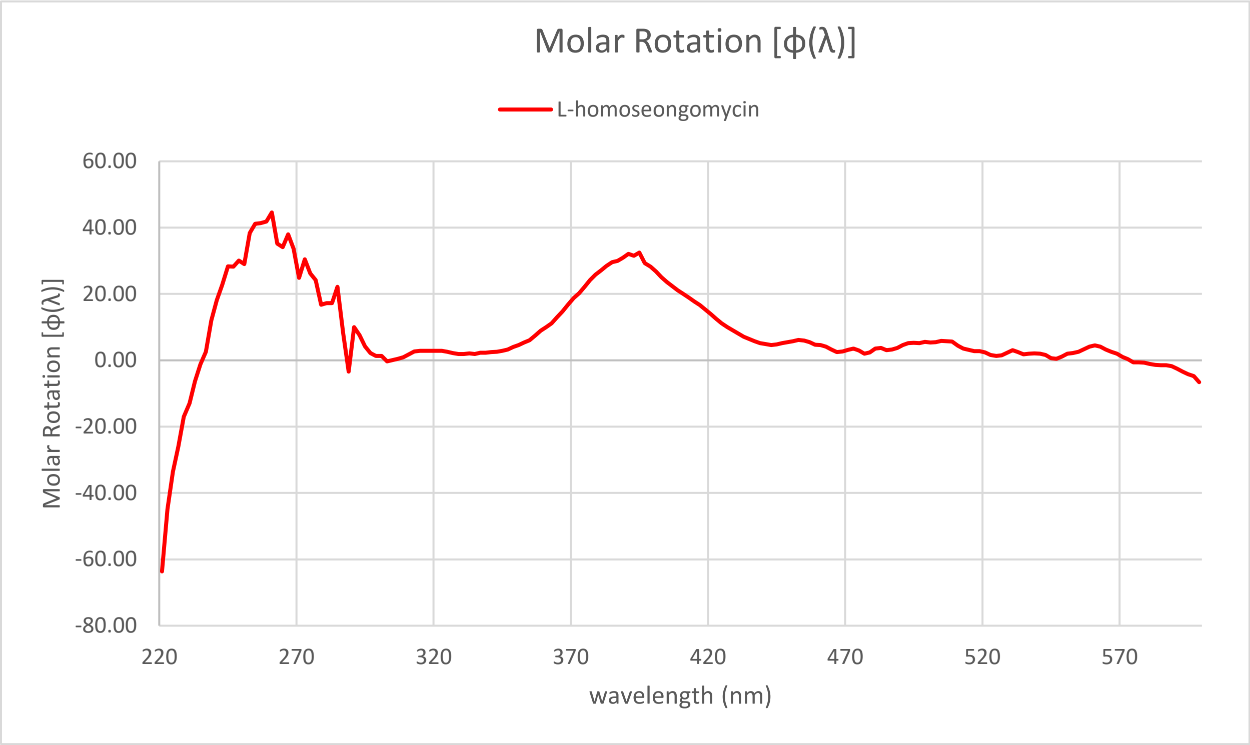

Figure S114. Transformed ECD spectra of *L*-**1** into corresponding ORD spectra (in units of molar rotation) using manual KK transform function.

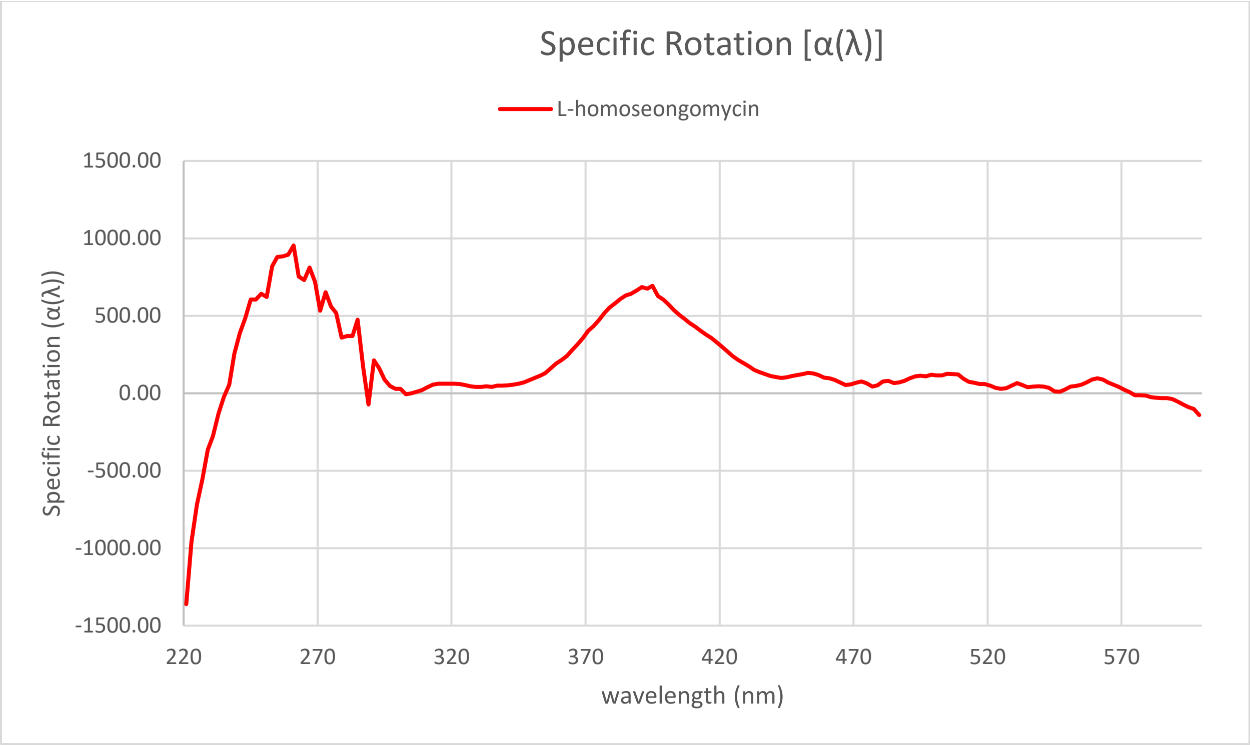

Figure S115. Transformed ECD spectra of *L*-**1** into corresponding ORD spectra (in units of specific rotation) using manual KK transform function.

Table S18. KK Transform data of *D*-homoseongomycin.

Experimental ECD (mdeg)	wavelength (μ)	molar Ellipticity (θ(μ))	λ for ORD	h	(2/π)(2h)(1/2)	Σ[(θ(μ)/(λ-μ))-(θ(μ)/(λ+μ))]	Molar Rotation [φ(λ)]	Specific Rotation (α(λ))
0.684509	600	4.59	599	1.98953	1.26657	2.76	3.49	74.64
0.616047	598	4.13	597			0.97	1.23	26.26
0.556178	596	3.73	595			-0.04	-0.06	-1.21
0.491688	594	3.30	593			-0.43	-0.55	-11.76
0.465136	592	3.12	591			-0.95	-1.20	-25.73
0.409613	590	2.75	589			-0.91	-1.15	-24.61
0.392437	588	2.63	587			-0.86	-1.09	-23.34
0.392449	586	2.63	585			-0.77	-0.98	-20.88
0.427857	584	2.87	583			-1.14	-1.45	-31.00
0.433974	582	2.91	581			-1.36	-1.73	-36.94
0.452983	580	3.04	579			-1.69	-2.14	-45.68
0.482235	578	3.24	577			-2.35	-2.97	-63.57
0.487047	576	3.27	575			-3.14	-3.97	-84.97
0.454462	574	3.05	573			-3.70	-4.69	-100.31
0.405776	572	2.72	571			-3.91	-4.95	-105.93
0.380475	570	2.55	569			-4.17	-5.29	-113.05
0.361503	568	2.43	567			-4.57	-5.78	-123.73
0.334391	566	2.24	565			-5.10	-6.46	-138.26
0.271781	564	1.82	563			-5.34	-6.76	-144.68
0.20436	562	1.37	561			-5.32	-6.74	-144.23
0.14341	560	0.96	559			-5.07	-6.42	-137.38
0.113081	558	0.76	557			-5.10	-6.46	-138.11
0.0466064	556	0.31	555			-4.57	-5.79	-123.89
0.0231385	554	0.16	553			-4.09	-5.18	-110.71
0.0298498	552	0.20	551			-3.91	-4.96	-106.00
0.0311831	550	0.21	549			-3.91	-4.95	-105.89
0.00631708	548	0.04	547			-3.57	-4.52	-96.76
0.0138733	546	0.09	545			-3.47	-4.40	-94.10
0.0173816	544	0.12	543			-3.49	-4.43	-94.66
0.0070906	542	0.05	541			-3.47	-4.39	-93.98
-0.0110771	540	-0.07	539			-3.31	-4.19	-89.71
-0.0211373	538	-0.14	537			-3.22	-4.08	-87.27
-0.0503186	536	-0.34	535			-2.74	-3.47	-74.21
-0.0437791	534	-0.29	533			-2.35	-2.97	-63.59
-0.0214722	532	-0.14	531			-2.03	-2.58	-55.12
0.0226423	530	0.15	529			-2.07	-2.62	-56.00
0.0558793	528	0.38	527			-2.25	-2.85	-61.07

Experimental ECD (mdeg)	wavelength (μ)	molar Ellipticity (θ(μ))	λ for ORD	h	(2/π)(2h)(1/2)	Σ[(θ(μ)/(λ-μ))-(θ(μ)/(λ+μ))]	Molar Rotation [φ(λ)]	Specific Rotation (α(λ))
0.075851	526	0.51	525			-2.52	-3.19	-68.19
0.0776698	524	0.52	523			-2.65	-3.36	-71.81
0.0855169	522	0.57	521			-2.86	-3.62	-77.35
0.0905541	520	0.61	519			-3.16	-4.00	-85.56
0.0841881	518	0.57	517			-3.56	-4.51	-96.39
0.0441205	516	0.30	515			-3.59	-4.55	-97.33
0.0188606	514	0.13	513			-3.68	-4.66	-99.68
-0.0268817	512	-0.18	511			-3.32	-4.21	-89.99
-0.0271987	510	-0.18	509			-3.30	-4.18	-89.39
-0.0466569	508	-0.31	507			-3.14	-3.97	-85.00
-0.0599748	506	-0.40	505			-2.92	-3.70	-79.11
-0.0593772	504	-0.40	503			-2.86	-3.63	-77.58
-0.0686241	502	-0.46	501			-2.87	-3.63	-77.69
-0.112502	500	-0.76	499			-2.19	-2.78	-59.45
-0.0858712	498	-0.58	497			-2.01	-2.54	-54.36
-0.0718331	496	-0.48	495			-1.88	-2.38	-50.88
-0.0677393	494	-0.45	493			-1.56	-1.98	-42.39
-0.0334509	492	-0.22	491			-1.57	-1.99	-42.61
-0.00743894	490	-0.05	489			-1.81	-2.30	-49.12
-0.0185718	488	-0.12	487			-1.74	-2.20	-47.04
-0.0152782	486	-0.10	485			-1.73	-2.19	-46.89
-0.0236425	484	-0.16	483			-1.51	-1.91	-40.78
-0.0071127	482	-0.05	481			-1.50	-1.90	-40.71
-0.00350682	480	-0.02	479			-1.47	-1.86	-39.72
-0.00525188	478	-0.04	477			-1.35	-1.70	-36.44
-0.00544845	476	-0.04	475			-1.15	-1.46	-31.13
-0.00049813	474	0.00	473			-0.86	-1.09	-23.30
0.0227926	472	0.15	471			-0.66	-0.84	-17.97
0.0587498	470	0.39	469			-0.72	-0.92	-19.61
0.0788186	468	0.53	467			-0.82	-1.04	-22.20
0.0808428	466	0.54	465			-0.68	-0.86	-18.46
0.102921	464	0.69	463			-0.76	-0.96	-20.49
0.0974412	462	0.65	461			-0.45	-0.57	-12.21
0.124304	460	0.83	459			-0.28	-0.35	-7.51
0.157537	458	1.06	457			-0.15	-0.19	-4.00
0.207759	456	1.39	455			-0.39	-0.50	-10.61
0.22168	454	1.49	453			-0.39	-0.49	-10.55
0.240765	452	1.62	451			-0.24	-0.30	-6.42
0.287748	450	1.93	449			-0.35	-0.44	-9.44

Experimental ECD (mdeg)	wavelength (μ)	molar Ellipticity (θ(μ))	λ for ORD	h	(2/π)(2h)(1/2)	Σ[(θ(μ)/(λ-μ))-(θ(μ)/(λ+μ))]	Molar Rotation [φ(λ)]	Specific Rotation (α(λ))
0.328413	448	2.20	447			-0.58	-0.74	-15.84
0.352969	446	2.37	445			-0.72	-0.91	-19.45
0.37998	444	2.55	443			-0.82	-1.04	-22.23
0.41201	442	2.77	441			-0.91	-1.15	-24.66
0.458647	440	3.08	439			-1.23	-1.56	-33.31
0.49275	438	3.31	437			-1.61	-2.03	-43.51
0.511512	436	3.43	435			-1.89	-2.39	-51.11
0.520619	434	3.49	433			-1.84	-2.33	-49.76
0.572541	432	3.84	431			-2.16	-2.73	-58.50
0.608044	430	4.08	429			-2.44	-3.09	-66.13
0.645881	428	4.33	427			-2.73	-3.45	-73.90
0.686972	426	4.61	425			-2.98	-3.77	-80.64
0.74532	424	5.00	423			-3.34	-4.23	-90.43
0.824387	422	5.53	421			-4.09	-5.18	-110.83
0.89405	420	6.00	419			-5.14	-6.51	-139.29
0.941061	418	6.32	417			-6.39	-8.09	-173.01
0.947745	416	6.36	415			-7.36	-9.32	-199.32
0.966176	414	6.48	413			-8.52	-10.79	-230.85
0.964578	412	6.47	411			-9.66	-12.24	-261.84
0.95361	410	6.40	409			-10.85	-13.74	-294.01
0.928207	408	6.23	407			-12.09	-15.31	-327.45
0.873455	406	5.86	405			-13.05	-16.53	-353.67
0.817979	404	5.49	403			-13.83	-17.52	-374.66
0.783147	402	5.26	401			-14.77	-18.71	-400.21
0.747935	400	5.02	399			-15.98	-20.23	-432.82
0.680726	398	4.57	397			-17.10	-21.66	-463.27
0.599211	396	4.02	395			-18.23	-23.09	-493.99
0.490864	394	3.29	393			-19.20	-24.32	-520.30
0.360472	392	2.42	391			-19.82	-25.11	-537.02
0.243862	390	1.64	389			-20.50	-25.97	-555.52
0.108206	388	0.73	387			-21.07	-26.69	-570.92
-0.0544634	386	-0.37	385			-21.18	-26.82	-573.70
-0.200482	384	-1.35	383			-21.18	-26.83	-573.84
-0.351033	382	-2.36	381			-20.94	-26.52	-567.22
-0.49511	380	-3.32	379			-20.48	-25.94	-554.85
-0.631168	378	-4.24	377			-19.74	-25.00	-534.76
-0.732592	376	-4.92	375			-19.17	-24.28	-519.41
-0.852275	374	-5.72	373			-18.14	-22.97	-491.37
-0.919596	372	-6.17	371			-17.40	-22.04	-471.40

Experimental ECD (mdeg)	wavelength (μ)	molar Ellipticity (θ(μ))	λ for ORD	h	(2/π)(2h)(1/2)	Σ[(θ(μ)/(λ-μ))-(θ(μ)/(λ+μ))]	Molar Rotation [φ(λ)]	Specific Rotation (α(λ))
-0.995874	370	-6.68	369			-16.50	-20.90	-447.05
-1.05228	368	-7.06	367			-15.61	-19.77	-422.97
-1.09055	366	-7.32	365			-14.83	-18.78	-401.72
-1.11696	364	-7.50	363			-14.22	-18.02	-385.39
-1.14671	362	-7.70	361			-13.72	-17.38	-371.77
-1.18512	360	-7.95	359			-13.27	-16.81	-359.48
-1.24548	358	-8.36	357			-12.52	-15.86	-339.28
-1.29657	356	-8.70	355			-11.56	-14.64	-313.23
-1.31655	354	-8.84	353			-10.76	-13.63	-291.58
-1.33814	352	-8.98	351			-9.86	-12.49	-267.20
-1.33952	350	-8.99	349			-9.06	-11.48	-245.50
-1.33027	348	-8.93	347			-8.37	-10.60	-226.64
-1.30981	346	-8.79	345			-7.92	-10.03	-214.52
-1.3011	344	-8.73	343			-7.56	-9.57	-204.81
-1.30793	342	-8.78	341			-7.13	-9.03	-193.06
-1.32041	340	-8.86	339			-6.54	-8.28	-177.17
-1.31296	338	-8.81	337			-6.23	-7.89	-168.76
-1.3371	336	-8.97	335			-5.62	-7.12	-152.39
-1.34765	334	-9.04	333			-5.06	-6.40	-136.96
-1.36675	332	-9.17	331			-4.33	-5.48	-117.21
-1.38533	330	-9.30	329			-3.33	-4.22	-90.25
-1.37659	328	-9.24	327			-2.33	-2.95	-63.01
-1.34344	326	-9.02	325			-1.53	-1.94	-41.46
-1.31835	324	-8.85	323			-0.62	-0.78	-16.75
-1.2775	322	-8.57	321			0.23	0.29	6.31
-1.23252	320	-8.27	319			1.06	1.35	28.83
-1.19628	318	-8.03	317			2.27	2.88	61.51
-1.11656	316	-7.49	315			3.45	4.38	93.60
-0.98996	314	-6.64	313			4.17	5.29	113.09
-0.868151	312	-5.83	311			4.72	5.98	127.92
-0.767536	310	-5.15	309			5.77	7.31	156.37
-0.595044	308	-3.99	307			6.42	8.13	173.95
-0.402209	306	-2.70	305			6.97	8.83	188.79
-0.151261	304	-1.02	303			6.87	8.70	186.05
0.122997	302	0.83	301			6.15	7.79	166.70
0.433294	300	2.91	299			4.02	5.09	108.89
0.639198	298	4.29	297			2.02	2.56	54.76
0.831639	296	5.58	295			0.29	0.36	7.76
1.16503	294	7.82	293			-4.42	-5.59	-119.64

Experimental ECD (mdeg)	wavelength (μ)	molar Ellipticity (θ(μ))	λ for ORD	h	(2/π)(2h)(1/2)	Σ[(θ(μ)/(λ-μ))-(θ(μ)/(λ+μ))]	Molar Rotation [φ(λ)]	Specific Rotation (α(λ))
1.25361	292	8.41	291			-8.71	-11.04	-236.06
1.23112	290	8.26	289			-11.88	-15.05	-321.94
1.29638	288	8.70	287			-16.20	-20.52	-438.89
1.3704	286	9.20	285			-24.06	-30.47	-651.85
0.862366	284	5.79	283			-27.31	-34.59	-739.95
0.391194	282	2.63	281			-28.50	-36.10	-772.21
0.0233276	280	0.16	279			-28.93	-36.65	-783.92
-0.239446	278	-1.61	277			-30.08	-38.10	-814.95
-0.631926	276	-4.24	275			-28.11	-35.61	-761.68
-0.587081	274	-3.94	273			-30.83	-39.05	-835.32
-0.794306	272	-5.33	271			-34.76	-44.03	-941.72
-1.44556	270	-9.70	269			-34.02	-43.09	-921.70
-2.01669	268	-13.53	267			-30.17	-38.21	-817.43
-2.25259	266	-15.12	265			-27.43	-34.75	-743.25
-2.44395	264	-16.40	263			-24.16	-30.60	-654.48
-2.41917	262	-16.24	261			-23.37	-29.60	-633.08
-2.52259	260	-16.93	259			-22.11	-28.01	-599.05
-2.5903	258	-17.38	257			-21.21	-26.87	-574.73
-2.65692	256	-17.83	255			-20.60	-26.09	-558.00
-2.73878	254	-18.38	253			-19.75	-25.01	-534.96
-2.72712	252	-18.30	251			-20.43	-25.87	-553.37
-2.73535	250	-18.36	249			-24.21	-30.67	-656.03
-3.31974	248	-22.28	247			-22.06	-27.94	-597.70
-3.67897	246	-24.69	245			-17.40	-22.03	-471.34
-3.44838	244	-23.14	243			-19.78	-25.06	-536.02
-3.62485	242	-24.33	241			-22.66	-28.69	-613.79
-4.03592	240	-27.09	239			-28.29	-35.83	-766.36
-5.71739	238	-38.37	237			-12.53	-15.87	-339.37
-5.46933	236	-36.71	235			-8.11	-10.27	-219.60
-5.19471	234	-34.86	233			-14.79	-18.73	-400.68
-6.78792	232	-45.56	231			2.05	2.60	55.67
-6.41958	230	-43.08	229			5.87	7.43	158.91
-6.36231	228	-42.70	227			-0.15	-0.19	-4.10
-8.8866	226	-59.64	225			35.54	45.02	962.97
-7.30157	224	-49.00	223			37.04	46.91	1003.50
-8.38409	222	-56.27	221			67.04	84.91	1816.30
-7.86591	220	-52.79						

ORD

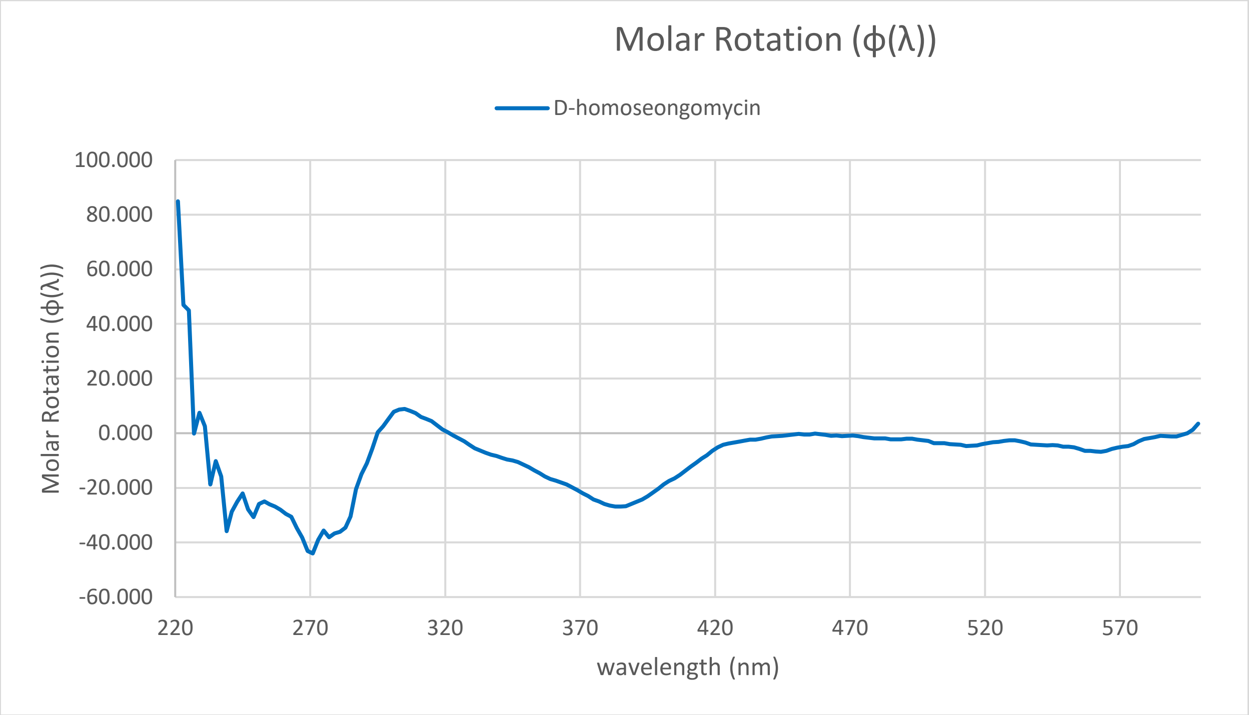

Figure S116. Transformed ECD spectra of *D*-**1** into corresponding ORD spectra (in units of molar rotation) using manual KK transform function.

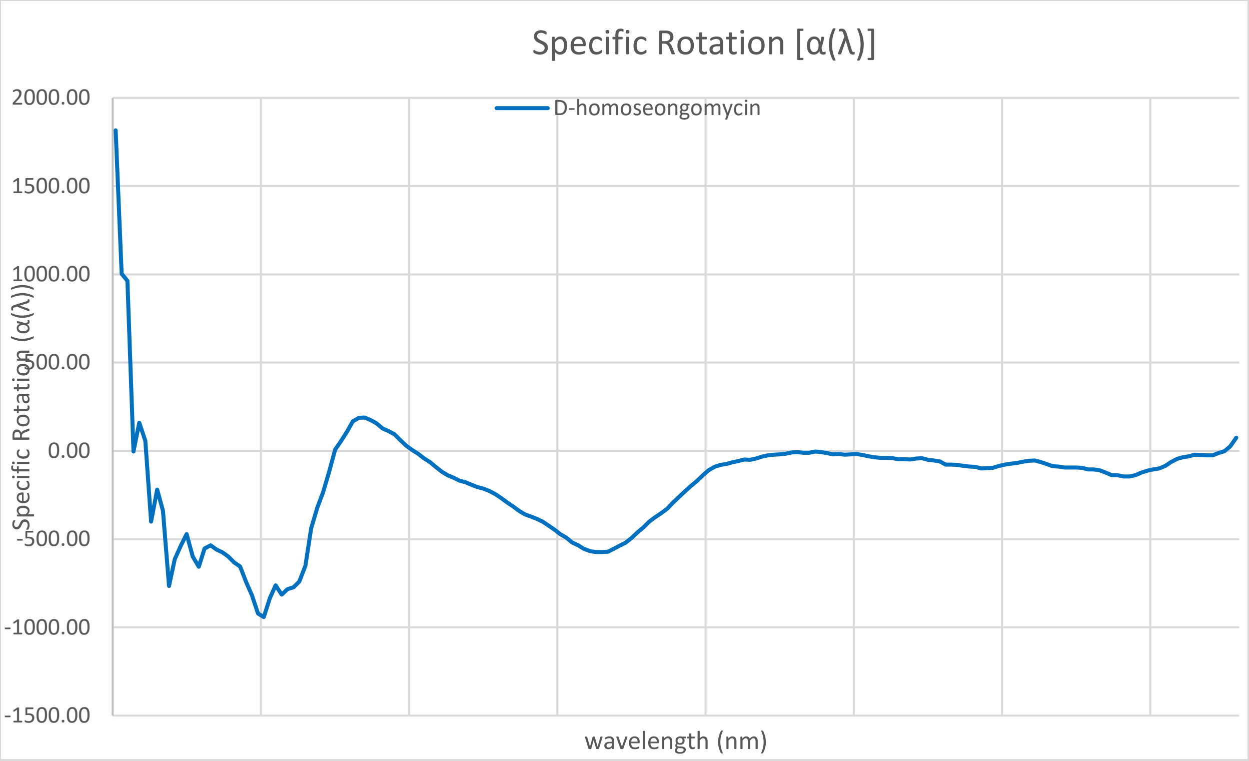

Figure S117. Transformed ECD spectra of *D*-**1** into corresponding ORD spectra (in units of specific rotation) using manual KK transform function.

References

(1) Wang, X.; Guo, J.; Qie, F.; Yan, Y. Enantioselective Addition of Diethylzinc to Aromatic Aldehydes Catalyzed by Chiral BINOL-Functionalized Nanoporous Graphene Oxides. *J. Mater. Sci.* **2019**, *54* (9), 6908–6916. https://doi.org/10.1007/s10853-018-03230-9.

(2) Sun, H.; DiMagno, S. G. Anhydrous Tetrabutylammonium Fluoride. *J. Am. Chem. Soc.* **2005**, *127* (7), 2050–2051. https://doi.org/10.1021/ja0440497.

(3) Engle, K. M.; Pfeifer, L.; Pidgeon, G. W.; Giuffredi, G. T.; Thompson, A. L.; Paton, R. S.; Brown, J. M.; Gouverneur, V. Coordination Diversity in Hydrogen-Bonded Homoleptic Fluoride–Alcohol Complexes Modulates Reactivity. *Chem. Sci.* **2015**, *6* (9), 5293–5302. https://doi.org/10.1039/C5SC01812A.

(4) Dong, Y.; Yang, J.; He, S.; Shi, Z.-C.; Wang, Y.; Zhang, X.-M.; Wang, J.-Y. Metal-Free Oxidative Cross-Dehydrogenative Coupling of Quinones with Benzylic C(Sp3)–H Bonds. *RSC Adv.* **2019**, *9* (47), 27588–27592. https://doi.org/10.1039/C9RA05678E.

(5) Urgin, K.; Jida, M.; Ehrhardt, K.; Müller, T.; Lanzer, M.; Maes, L.; Elhabiri, M.; Davioud-Charvet, E. Pharmacomodulation of the Antimalarial Plasmodione: Synthesis of Biaryl- and N-Arylalkylamine Analogues, Antimalarial Activities and Physicochemical Properties. *Molecules* **2017**, *22* (1), 161. https://doi.org/10.3390/molecules22010161.

(6) Sutherland, D. R.; Veguillas, M.; Oates, C. L.; Lee, A.-L. Metal-, Photocatalyst-, and Light-Free, Late-Stage C–H Alkylation of Heteroarenes and 1,4-Quinones Using Carboxylic Acids. *Org. Lett.* **2018**, *20* (21), 6863–6867. https://doi.org/10.1021/acs.orglett.8b02988.

(7) Galloway, J. D.; Mai, D. N.; Baxter, R. D. Silver-Catalyzed Minisci Reactions Using Selectfluor as a Mild Oxidant. *Org. Lett.* **2017**, *19* (21), 5772–5775. https://doi.org/10.1021/acs.orglett.7b02706.

(8) Namsa-aid, A.; Ruchirawat, S. Efficient Synthesis of Ningalin C. *Org. Lett.* **2002**, *4* (16), 2633–2635. https://doi.org/10.1021/ol026074s.

(9) Jiang, J.-H.; Boominathan, S. S. K.; Hu, W.-P.; Chen, C.-Y.; Vandavasi, J. K.; Lin, Y.-T.; Wang, J.-J. Sequential, One-Pot Access to Arylated Benzoquinones/Naphthoquinones from Phenols/Naphthols. *Eur. J. Org. Chem.* **2016**, *2016* (13), 2284–2289. https://doi.org/10.1002/ejoc.201600119.

(10) Roy, B. N.; Singh, G. P.; Lathi, P. S.; Agrawal, M. K.; Mitra, R.; Trivedi, A. A Novel Process for Synthesis of Atovaquone. *INDIAN J CHEM* **2013**.

(11) Franck, R. W.; Gupta, R. B. Baeyer-Villiger Oxidation of Naphthaldehydes: Easy Access to Naphthoquinones. *J. Org. Chem.* **1985**, *50* (23), 4632–4635. https://doi.org/10.1021/jo00223a041.

(12) Yin, J.; Landward, M. B.; Rainier, J. D. Photoelectrocyclization Reactions of Amidonaphthoquinones. *J. Org. Chem.* **2020**, *85* (6), 4298–4311. https://doi.org/10.1021/acs.joc.9b03417.

(13) Williams, W.; Sun, X.; Jebaratnam, D. Synthetic Studies on the Kinamycin Family of Antibiotics: Synthesis of 2-(Diazobenzyl)- *p* -Naphthoquinone, 1,7-Dideoxy-3-Demethylprekinamycin, and 1-Deoxy-3-Demethylprekinamycin. *J. Org. Chem.* **1997**, *62* (13), 4364–4369. https://doi.org/10.1021/jo9700484.

(14) Naber, J. R.; Buchwald, S. L. Palladium-Catalyzed Stille Cross-Coupling Reaction of Aryl Chlorides Using a Pre-Milled Palladium Acetate and XPhos Catalyst System. *Adv. Synth. Catal.* **2008**, *350* (7–8), 957–961. https://doi.org/10.1002/adsc.200800032.

(15) Mee, S. P. H.; Lee, V.; Baldwin, J. E. Stille Coupling Made Easier—The Synergic Effect of Copper(I) Salts and the Fluoride Ion. *Angew. Chem. Int. Ed.* **2004**, *43* (9), 1132–1136. https://doi.org/10.1002/anie.200352979.

(16) Barder, T. E.; Walker, S. D.; Martinelli, J. R.; Buchwald, S. L. Catalysts for Suzuki−Miyaura Coupling Processes: Scope and Studies of the Effect of Ligand Structure. *J. Am. Chem. Soc.* **2005**, *127* (13), 4685–4696. https://doi.org/10.1021/ja042491j.

(17) Knapp, D. M.; Gillis, E. P.; Burke, M. D. A General Solution for Unstable Boronic Acids: Slow-Release Cross-Coupling from Air-Stable MIDA Boronates. *J. Am. Chem. Soc.* **2009**, *131* (20), 6961–6963. https://doi.org/10.1021/ja901416p.

(18) Pradeep, P.; Ngwira, K. J.; Reynolds, C.; Rousseau, A. L.; Lemmerer, A.; Fernandes, M. A.; Johnson, M. M.; de Koning, C. B. Novel Methodology for the Synthesis of the Benzo[b]Phenanthridine and 6H-Dibenzo[c,h]Chromen-6-One Skeletons. Reactions of 2-Naphthylbenzylamines and 2-Naphthylbenzyl Alcohols. *Tetrahedron* **2016**, *72* (51), 8417–8427. https://doi.org/10.1016/j.tet.2016.10.071.

(19) Scully, S. S.; Porco Jr., J. A. Asymmetric Total Synthesis of the Epoxykinamycin FL-120 B′. *Angew. Chem. Int. Ed.* **2011**, *50* (41), 9722–9726. https://doi.org/10.1002/anie.201104504.

(20) Koyama, H.; Kamikawa, T. Total Syntheses of O4,O9-Dimethylstealthins A and C 1. *J. Chem. Soc. Perkin 1* **1998**, No. 2, 203–210. https://doi.org/10.1039/a706470e.

(21) Wehlan, H.; Jezek, E.; Lebrasseur, N.; Pavé, G.; Roulland, E.; White, A. J. P.; Burrows, J. N.; Barrett, A. G. M. Studies on the Total Synthesis of Lactonamycin: Synthesis of the CDEF Ring System. *J. Org. Chem.* **2006**, *71* (21), 8151–8158. https://doi.org/10.1021/jo0613378.

(22) Hajipour, A. R.; Zarei, A.; Khazdooz, L.; Ruoho, A. E. Simple and Efficient Procedure for the Friedel–Crafts Acylation of Aromatic Compounds with Carboxylic Acids in the Presence of P_2O_5 /AL_2O_3 Under Heterogeneous Conditions. *Synth. Commun.* **2009**, *39* (15), 2702–2722. https://doi.org/10.1080/00397910802663436.

(23) Nicolaou, K. C.; Chen, Q.; Li, R.; Anami, Y.; Tsuchikama, K. Total Synthesis of the Monomeric Unit of Lomaiviticin A. *J. Am. Chem. Soc.* **2020**, *142* (47), 20201–20207. https://doi.org/10.1021/jacs.0c10660.

(24) Kimura, S.; Kobayashi, S.; Kumamoto, T.; Akagi, A.; Sato, N.; Ishikawa, T. Syntheses of Prekinamycin and a Tetracyclic Quinone from Common Synthetic Intermediates. *Helv. Chim. Acta* **2011**, *94* (4), 578–591. https://doi.org/10.1002/hlca.201000296.

(25) Wang, B.; Sun, H.-X.; Sun, Z.-H.; Lin, G.-Q. Direct B-Alkyl Suzuki–Miyaura Cross-Coupling of Trialkyl- Boranes with Aryl Bromides in the Presence of Unmasked Acidic or Basic Functions and Base-Labile Protections under Mild Non-Aqueous Conditions. *Adv. Synth. Catal.* **2009**, *351* (3), 415–422. https://doi.org/10.1002/adsc.200800630.

(26) Grob, J. E.; Nunez, J.; Dechantsreiter, M. A.; Hamann, L. G. One-Pot Reductive Amination and Suzuki–Miyaura Cross-Coupling of Formyl Aryl and Heteroaryl MIDA Boronates in Array Format. *J. Org. Chem.* **2011**, *76* (12), 4930–4940. https://doi.org/10.1021/jo2005928.

(27) Comins, D. L.; Brown, J. D. Ortho Metalation Directed by .Alpha.-Amino Alkoxides. *J. Org. Chem.* **1984**, *49* (6), 1078–1083. https://doi.org/10.1021/jo00180a024.

(28) Mori, K.; Ichikawa, Y.; Kobayashi, M.; Shibata, Y.; Yamanaka, M.; Akiyama, T. Enantioselective Synthesis of Multisubstituted Biaryl Skeleton by Chiral Phosphoric Acid Catalyzed Desymmetrization/Kinetic Resolution Sequence. *J. Am. Chem. Soc.* **2013**, *135* (10), 3964–3970. https://doi.org/10.1021/ja311902f.

(29) Jagot, F.; Ntsimango, S.; Ngwira, K. J.; Fernandes, M. A.; de Koning, C. B. Synthesis of Angucycline/Tetrangulol Derivatives Using Suzuki-Miyaura Cross-Coupling and Ring-Closing Carbonyl-Olefin Metathesis Reactions. *Eur. J. Org. Chem.* **2022**, *2022* (24), e202200348. https://doi.org/10.1002/ejoc.202200348.

(30) Xu, X.; Kim, H.-S.; Chen, W.-M.; Ma, X.; Correy, G. J.; Banwell, M. G.; Jackson, C. J.; Willis, A. C.; Carr, P. D. Total Syntheses of the Amaryllidaceae Alkaloids Zephycandidine III and Lycosinine A and Their Evaluation as Inhibitors of Acetylcholinesterase. *Eur. J. Org. Chem.* **2017**, *2017* (27), 4044–4053. https://doi.org/10.1002/ejoc.201700705.

(31) Laha, J. K.; Gulati, U.; Saima; Schulte, T.; Breugst, M. pH-Controlled Intramolecular Decarboxylative Cyclization of Biarylacetic Acids: Implication on Umpolung Reactivity of Aroyl Radicals. *J. Org. Chem.* **2022**, *87* (10), 6638–6656. https://doi.org/10.1021/acs.joc.2c00295.

(32) Kangani, C. O.; Day, B. W. Mild, Efficient Friedel−Crafts Acylations from Carboxylic Acids Using Cyanuric Chloride and AlCl3. *Org. Lett.* **2008**, *10* (13), 2645–2648. https://doi.org/10.1021/ol800752v.
(33) Javed, M. I.; Brewer, M. Diazo Preparation via Dehydrogenation of Hydrazones with “Activated” DMSO. *Org. Lett.* **2007**, *9* (9), 1789–1792. https://doi.org/10.1021/ol070515w.
(34) Woo, C. M.; Gholap, S. L.; Herzon, S. B. Insights into Lomaiviticin Biosynthesis. Isolation and Structure Elucidation of (−)-Homoseongomycin. *J. Nat. Prod.* **2013**, *76* (7), 1238–1241. https://doi.org/10.1021/np400355h.
(35) Lin, S.-C.; Lehman, C. W.; Stewart, A. K.; Panny, L.; Bracci, N.; Wright, J. L. C.; Paige, M.; Strangman, W. K.; Kehn-Hall, K. Homoseongomycin, a Compound Isolated from Marine Actinomycete Bacteria K3-1, Is a Potent Inhibitor of Encephalitic Alphaviruses. *Antiviral Res.* **2021**, *191*, 105087. https://doi.org/10.1016/j.antiviral.2021.105087.
(36) Carney, J. R.; Hong, S.-T.; Gould, S. J. Seongomycin: A New Sulfur-Containing Benzo[b]Fluorene Derived from Genes Clustered with Those for Kinamycin Biosynthesis. *Tetrahedron Lett.* **1997**, *38* (18), 3139–3142. https://doi.org/10.1016/S0040-4039(97)00616-3.
(37) Polavarapu, P. L. Kramers−Kronig Transformation for Optical Rotatory Dispersion Studies. *J. Phys. Chem. A* **2005**, *109* (32), 7013–7023. https://doi.org/10.1021/jp0524328.
